# Cardiac cystic echinococcosis—A systematic review and analysis of the literature

**DOI:** 10.1371/journal.pntd.0012183

**Published:** 2024-05-30

**Authors:** Simone Bumann, Esther Kuenzli, Raffaella Lissandrin, Enrico Brunetti, Sam Goblirsch, Lars Henning, Francesca Tamarozzi, Andreas Neumayr

**Affiliations:** 1 Swiss Tropical and Public Health Institute, Basel, Switzerland; 2 University of Basel, Basel, Switzerland; 3 Division of Infectious and Tropical Diseases, University of Pavia, IRCCS S. Matteo Hospital Foundation, WHO Collaborating Centre on Clinical Management of Cystic Echinococcosis, Pavia, Italy; 4 Department of Medicine and Pediatrics, University of Minnesota, Minneapolis, Minnesota, United States of America; 5 Department of Public Health and Tropical Medicine, College of Public Health, Medical and Veterinary Sciences, James Cook University, Queensland, Australia; 6 Department of Infectious-Tropical Diseases and Microbiology, WHO Collaborating Centre on Strongyloidiasis and other Neglected Tropical Diseases, IRCCS Sacro Cuore Don Calabria Hospital, Negrar di Valpolicella, Verona, Italy; Istituto Superiore Di Sanita, ITALY

## Abstract

Human cystic echinococcosis (CE) is a parasitic infection caused by the larval stage of the tapeworm *Echinococcus granulosus sensu lato*, primarily affecting the liver and lungs. Although the heart is affected in only 0.02–2% of all CE cases, a considerable number of cases have been, and continue to be, published. However, due to the rare occurrence of cardiac CE and the resulting lack of clinical trials, knowledge about various aspects of the disease remains limited. To obtain a clearer picture of anatomical, clinical, diagnostic as well as therapeutic aspects of cardiac CE, we systematically reviewed the literature published between 1965 and 2022.

The anatomical pattern of the affected cardiac structures follows the extension of the supplying capillary bed. The majority of patients (82.7%) are symptomatic and present with prolonged non-specific symptoms such as dyspnoea, chest pain and palpitations. Acute complications generally derive from cyst rupture, occur in 18.3% of cases and manifest as embolism, pericardial tamponade, or anaphylactic reaction in 83.2%, 17.8% and 10.9% of these cases, respectively. As for CE cysts localized in other organs, the diagnosis of cardiac CE is made by imaging. Serology plays a minor role due to its limited sensitivity. Unlike abdominal CE cysts, cardiac CE cysts are usually resected independent of their stage (active/inactive), because their presence impairs cardiac performance and carries the risk of long-term sequelae. More than 80% of patients are treated with a single surgical intervention. We found a disease-related case fatality rate of 11.1%. Since local recurrence was reported up to 108 months and secondary CE up to 72 months after surgery, patients should be followed up for a minimum of 10 years.

## Author summary

Human cystic echinococcosis (CE) is a parasitic infection caused by the larva of the dog tapeworm *Echinococcus granulosus*, which primarily affects the liver and lungs. Although the heart is rarely affected (≤2% of cases), a considerable number of case reports continue to be published. While the diagnostic and clinical management of cardiac CE has greatly benefited from the increasingly widespread availability of diagnostic imaging techniques as well as from advances in heart surgery, the overall picture of cardiac CE remains patchy and several aspects remain poorly understood. To overcome this, we systematically reviewed the last five decades of published literature on cardiac CE, and analyzed and summarized the existing data.

## Introduction

Cystic echinococcosis (CE) is a zoonotic parasitosis caused by the larval stage (metacestode) of the tapeworm *Echinococcus granulosus sensu lato*. The parasite occurs worldwide, and is primarily endemic in pastoral regions. The parasite’s life cycle involves two hosts. The definitive host is a canid, usually the domestic dog, which harbours the adult parasite in the small bowel. The intermediate ungulate host, most commonly a sheep, becomes infected perorally when grazing on ground contaminated by parasite’s eggs shed with the feces of an infected dog. After ingestion, the embryo (oncosphere) hatches, penetrates the intestinal mucosa, enters into the host’s circulatory system, and, if not destroyed by the host’s immune response, develops into the characteristic vesicular fluid-filled metacestode (‘hydatid cyst’), when reaching a suitable anatomical site. This grows expansively and develops internally the protoscoleces. When the definitive host feeds on viscera with cysts containing protoscoleces, the cycle is complete [1].

Humans, which are accidental, dead-end hosts, become infected by the accidental ingestion of parasite eggs. In ~80% of the cases, a single organ is involved, primarily the liver (73.4% of the cases [2]) and the lungs (19.6% of the cases [2]), but virtually any part of the body may be affected, including the heart [3]. The earliest reference to the frequency of cardiac CE in the literature is from 1894 by Davies Thomas, who estimated that this would occur in 1.8% of all CE cases (cited in [4]). This corresponds to the figure of ≤2%, most frequently quoted in the literature [5].

**Fig 1:**
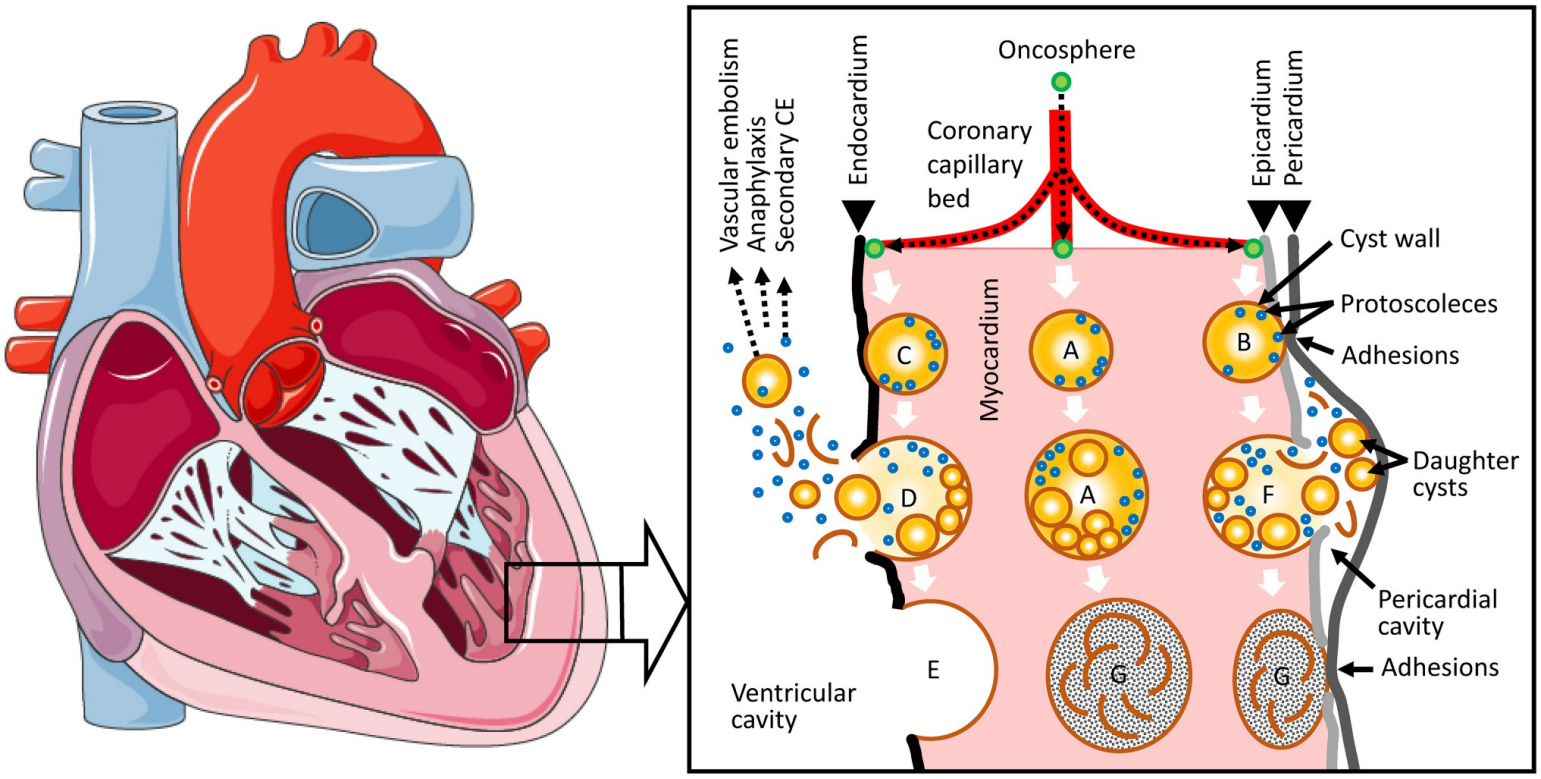
Anatomical model of the evolution of cardiac CE cysts according to Birincioglu and colleagues (modified from [5]). Depending on the localization of the oncosphere’s implantation, CE cysts develop embedded in the myocardium (A), close to the epicardium growing towards the epicardium (B), close to the endocardium growing towards the endocardium (C), rupture into the ventricular cavity (D), rupture into the pericardial cavity (F), or inactivate over time (G). Remnants of ruptured cysts (E) and inactive cysts (G) are exemplarily shown as sequelae of subendocardial and subpericardial cysts, respectively, but may occur equally in both sites. [human heart graphic adapted from Servier Medical Art (https://smart.servier.com/smart_image/smart-heart-sagittal-overview/) under CC BY 4.0 licensing].

As in other anatomic locations, cardiac CE cysts undergo natural evolution (or transition) from active/viable to inactive/non-viable and the changing morphology over time defines six cysts stages: CE1, CE2, CE3b (active/viable), CE3a (transitional), and CE4 and CE5 (inactive/non-viable) [6,7]. Cysts may grow either within the myocardium or towards the endocardium or the epicardium. Depending on their location, cyst growth may also cause subaortic or subpulmonary infundibular obstruction, coronary artery compression, or pericardiac tamponade. Fig 1 shows the putative anatomic development options of cardiac CE cysts according to the model proposed by Birincioglu and colleagues [5]. If the cyst grows close to the endocardium or the epicardium, the constant mechanical shear stress tends to lead to cyst rupture and spillage of cyst content (protoscoleces or daughter cysts) into the bloodstream or the pericardial sack, respectively, causing vascular embolism, secondary CE with the development of cysts in other organs or tissues (e.g., lungs), and potentially fatal anaphylactic reactions. Perforation of the cyst(s) during surgery is also a feared complication [6].

Diagnosis of CE is primarily made by imaging (ultrasonography [US], computed tomography [CT], magnetic resonance imaging [MRI]), with serology being used as an adjunct confirmatory diagnostic method [6]. Surgical removal of the cyst(s) is the treatment of choice in cardiac CE. The only other available treatment option for cardiac CE in active stages is antiparasitic treatment with a benzimidazole compound. Since local recurrence or secondary CE may present with a delay of many years or even decades, assessing the long-term treatment outcome of cardiac CE, as well as for other localizations, remains difficult.

The first traceable report of cardiac CE was published by Williams in 1836 [8]. The first surgical attempt to treat cardiac CE was made by Marten and de Crespigny in 1921 [9] and the first successful surgical intervention for cardiac CE was reported by Long in 1932 [10]. As with any rare disease manifestation, reaching scientific evidence on different medical aspects is difficult, since the vast majority of publications are case reports or case series, the larger ones, even from referral centres in endemic areas, describing only a few dozen of cases [5,11–13].

To obtain a clearer picture of cardiac CE, we systematically searched the literature for case reports and case series of cardiac CE published from 1965 to 2022 and analyzed the reported data on epidemiology, anatomy, symptoms, diagnosis, treatment, complications, and outcome. The restriction to the last five decades was deliberate, because neither appropriate imaging nor drug treatment was available before that time, and cardiac surgical techniques were still in their infancy. Furthermore, by 1964, only 43 successfully treated cases of cardiac CE had been described in the literature [14].

## Methods

We searched PubMed (MEDLINE; NCBI) using the string "(Cardiac OR Heart) AND (Echinococcosis OR Hydatidosis OR Hydatid Disease OR Echinococcal Cyst OR Hydatid Cyst) AND ("1965/01/01"[Date—Publication]: "2022/12/31"[Date—Publication])" and reviewed all retrieved references published in English, German, French, Italian, and Spanish (Fig 1 and S1 Text file). After screening by title and abstract, any publication that contained data on one or more cardiac CE cases was considered eligible for inclusion and full-text review. From these publications any retrievable data on epidemiology (patients’ age, sex and country of origin), symptoms, diagnostic methods, anatomical localization, cysts’ size and stage, medical history, localization and complications of concomitantly or previously diagnosed and treated extracardiac cysts, treatment modality, treatment outcome, and follow-up were extracted into a Microsoft Excel spreadsheet (Version 2010) for analysis. From each publication, data were extracted by two authors and discrepancies as well as the inclusion or exclusion of cases with unclear or contradictory data were clarified by consultation and discussion with a third author. If the patient’s country of origin was not reported, the country where the case was managed was used as a surrogate if it corresponded to a known CE endemic area. Continuous data were summarized as medians and ranges. Nominal and ordinal data were summarized as frequencies and percentages. Chi-square test and logistic regression were applied to evaluate correlations between patient- and cyst-specific parameters and the presence of eosinophilia or positive *Echinococcus* serology. Parameters demonstrating a p-value <0.1 in univariate logistic regression were also evaluated by multivariate logistic regression. Statistical analysis was conducted using Stata statistical software (Stata 16, StataCorp LLC 2019, Texas, USA).

## Results

**Fig 2:**
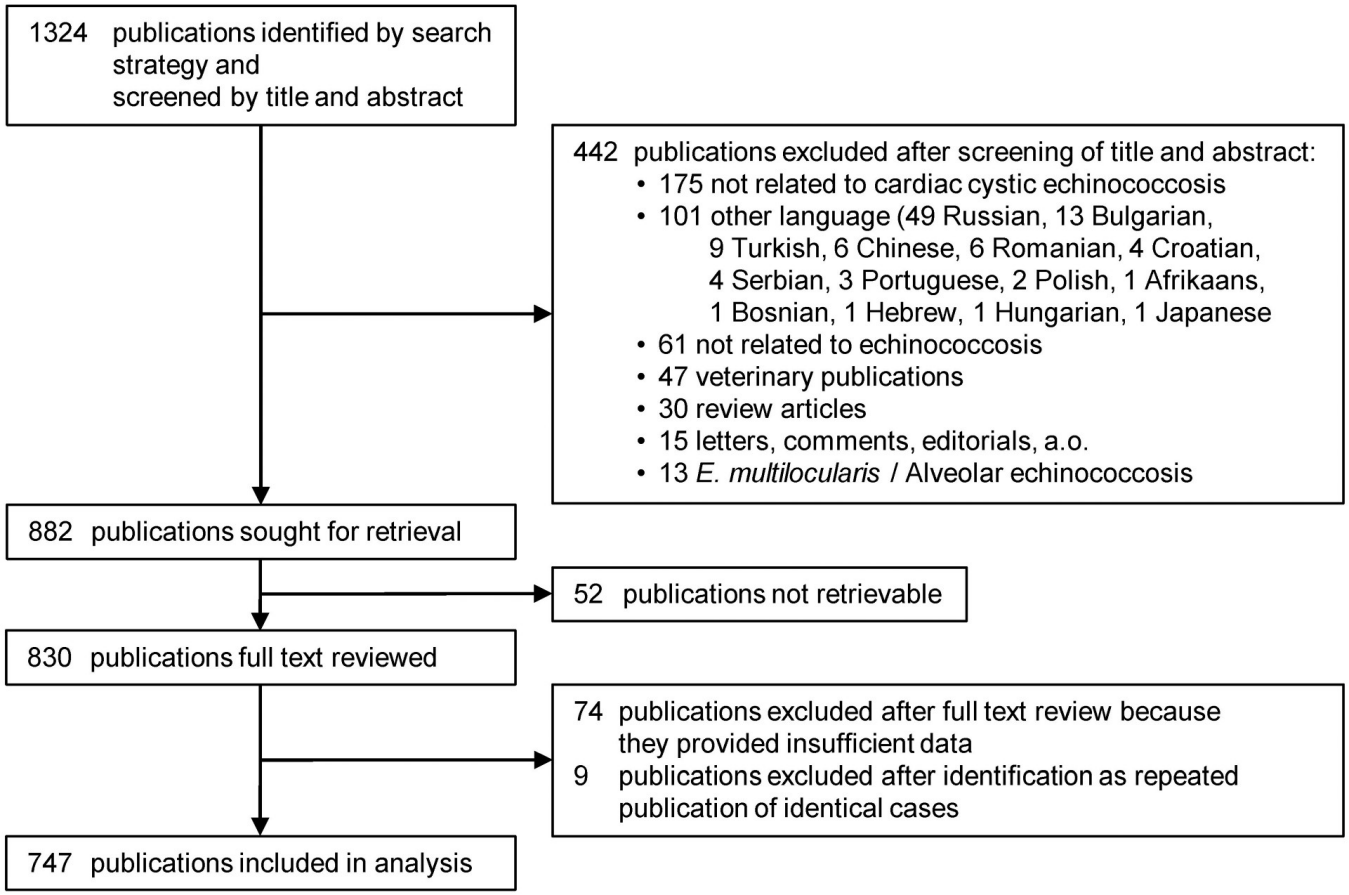
Flow diagram of search and selection of eligible publications on human cardiac cystic echinococcosis.

The search strategy identified 1324 publications (Fig 2). Of these, 442 were excluded after review of the title and abstract because either (i) not related to CE, (ii) not related to cardic CE, (iii) published in a language other than specified above, (iv) concerned veterinary cases, (v) were review articles, letters, comments, editorials, or (vi) were on alveolar echinococcosis. 52 publications were not retrievable. Of the 830 publications undergoing full text review, 74 were excluded because they provided insufficient data and 9 because they were repeated publications of identical cases. Finally, data from 747 publications on a total of 1210 human cardiac CE cases were included in the analysis (Fig 2).

### Epidemiology

**Fig 3:**
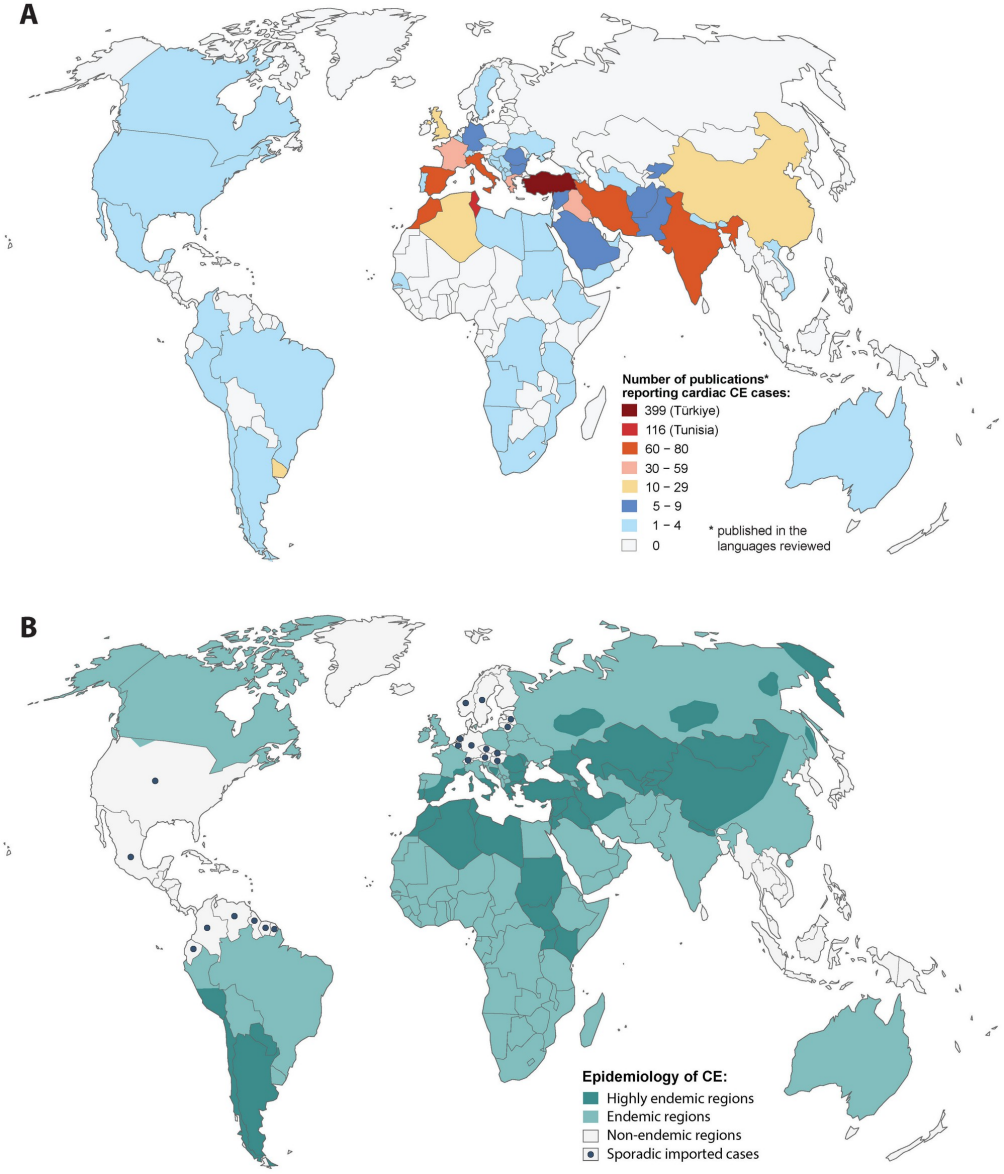
Geographical origin of published cases of cardiac cystic echinococcosis and global endemicity of cystic echinococcosis. (A) Number of publications reporting cardiac cystic echinococcosis cases published from 1965 to 2023 included in the review; (B) Global epidemiology of cystic echinococcosis [map created by Rosalie Zimmermann, adapted from Neumayr A.: Antiparasitic Treatment Recommendations. 2018, 2^nd^ edition. Tredition, Hamburg]

**Fig 4:**
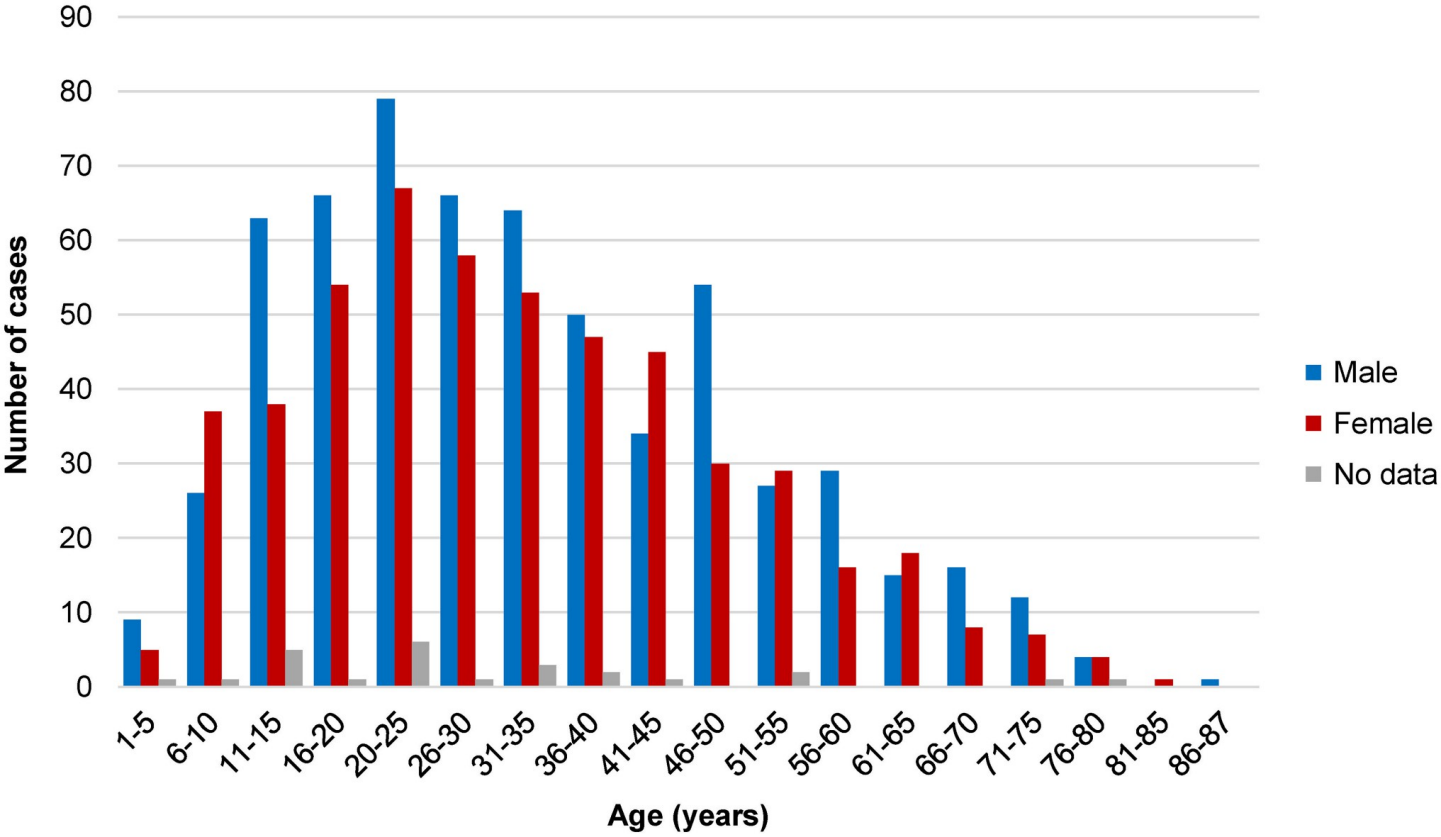
Age and sex distribution of published cases of cardiac cystic echinococcosis (n_age_ = 1157; n_sex_ = 1132).

Data on the geographic origin of human cardiac CE were available for 1172 (96.9%) cases (Fig 3). Data on the patients’ sex was available for 1132 (93.6%) cases (615 male, 517 female). Data on the age of the patients were available for 1157 (95.6%) cases; median age was 30 years (range 2–87), with no significant difference between male (median 30, range 3–87) and female patients (median 30, range 2–85). The age and sex distribution of the retrieved cases is shown in Fig 4.

### Anatomy

**Fig 5:**
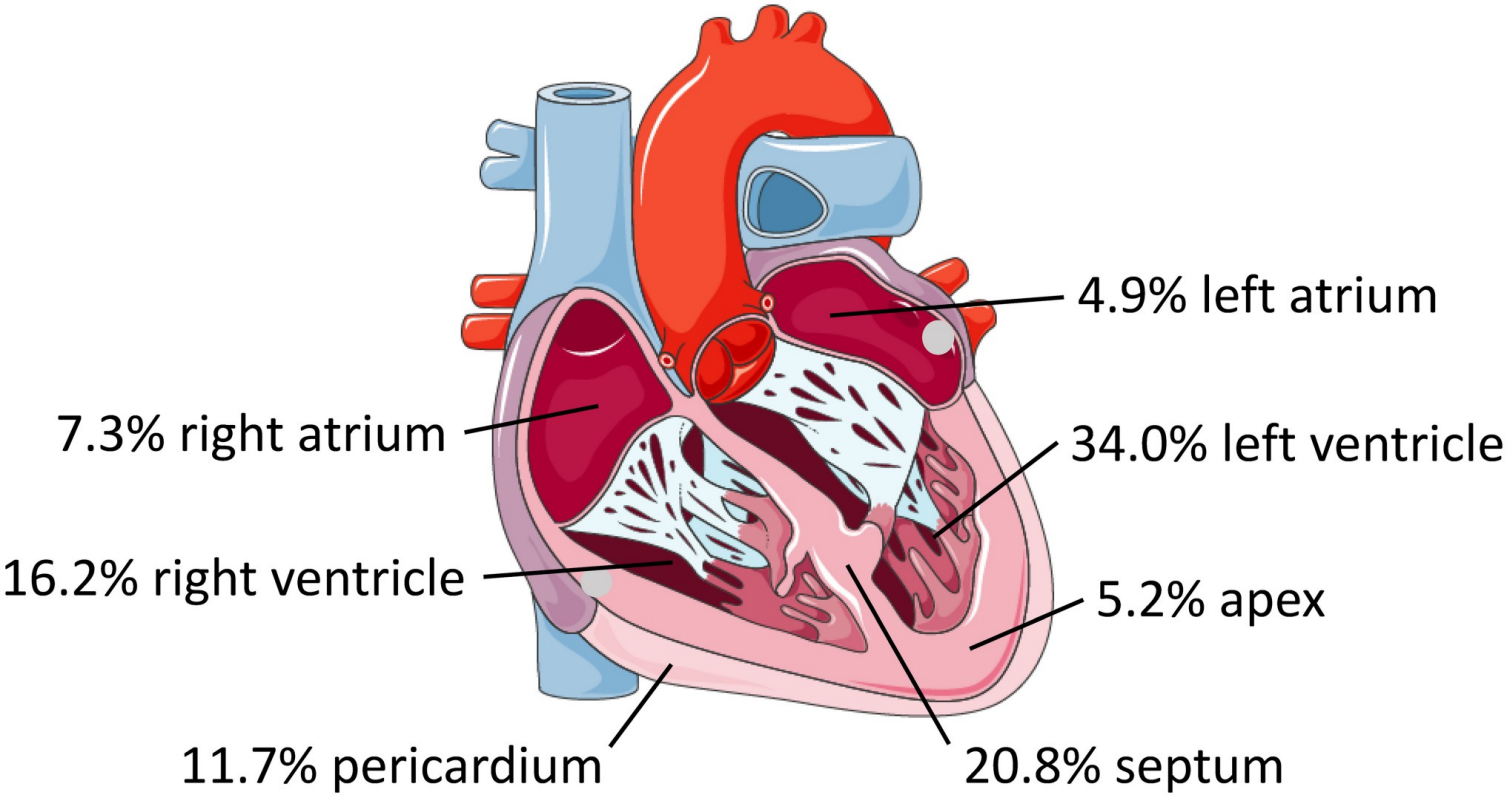
Percentage distribution of cardiac echinococcal cysts (n = 1397) according to the affected heart structure. [human heart graphic adapted from Servier Medical Art (https://smart.servier.com/smart_image/smart-heart-sagittal-overview/) under CC BY 4.0 licensing].

Data on the number of cardiac CE cysts were reported for 1125 (93%) patients. Of these, 932 (82.8%) had unilocular cysts and 193 (17.2%) had multiloculated (i.e. with daughter cysts) cysts. The exact anatomic localization was specified for 1397 (93.8%) of the 1489 reported cysts. Fig 5 shows the percentage distribution according to the affected cardiac structures.

Data on the cyst size was available for 974 (69.7%) of the cysts with specified anatomical localization (Table 1).

**Table 1:** Data on the size of cardiac cystic echinococcosis cysts with specified anatomical localization (n = 974).

Cardiac structure affected by CE	Cardiac CE cysts with data on localization and size N (%)	Median cyst size cm (range)
**Left ventricle**	362 (37.2)	5 (0.8–20)
**Septum**	248 (25.4)	4.8 (0.5–20)
**Right ventricle**	156 (16.0)	4.6 (0.5–12)
**Pericardium**	105 (10.8)	6.6 (1.5–13)
**Right atrium**	60 (6.2)	4 (1.5–8)
**Left atrium**	24 (2.5)	4 (2–10)
**Apex**	19 (2.0)	6 (2–10)

CE, cystic echinococcosis.

Data on the integrity of the cyst(s) were available for 1008 (83.3%) cases. Cyst rupture was reported in 158 (15.7%) of the cases. Data on the cyst stage, according to WHO classification, were available for 153 (10.3%) cysts: CE1, n = 48; CE2, n = 69; CE3A, n = 22; CE3B, n = 2; CE4, n = 4; CE5, n = 8. Of the 163 cases presenting with pericardial cysts, 94 cases (57.7%) did not have concomitantly present myocardial cysts while 69 cases (42.3%) had concomitantly present myocardial cysts.

**Fig 6:**
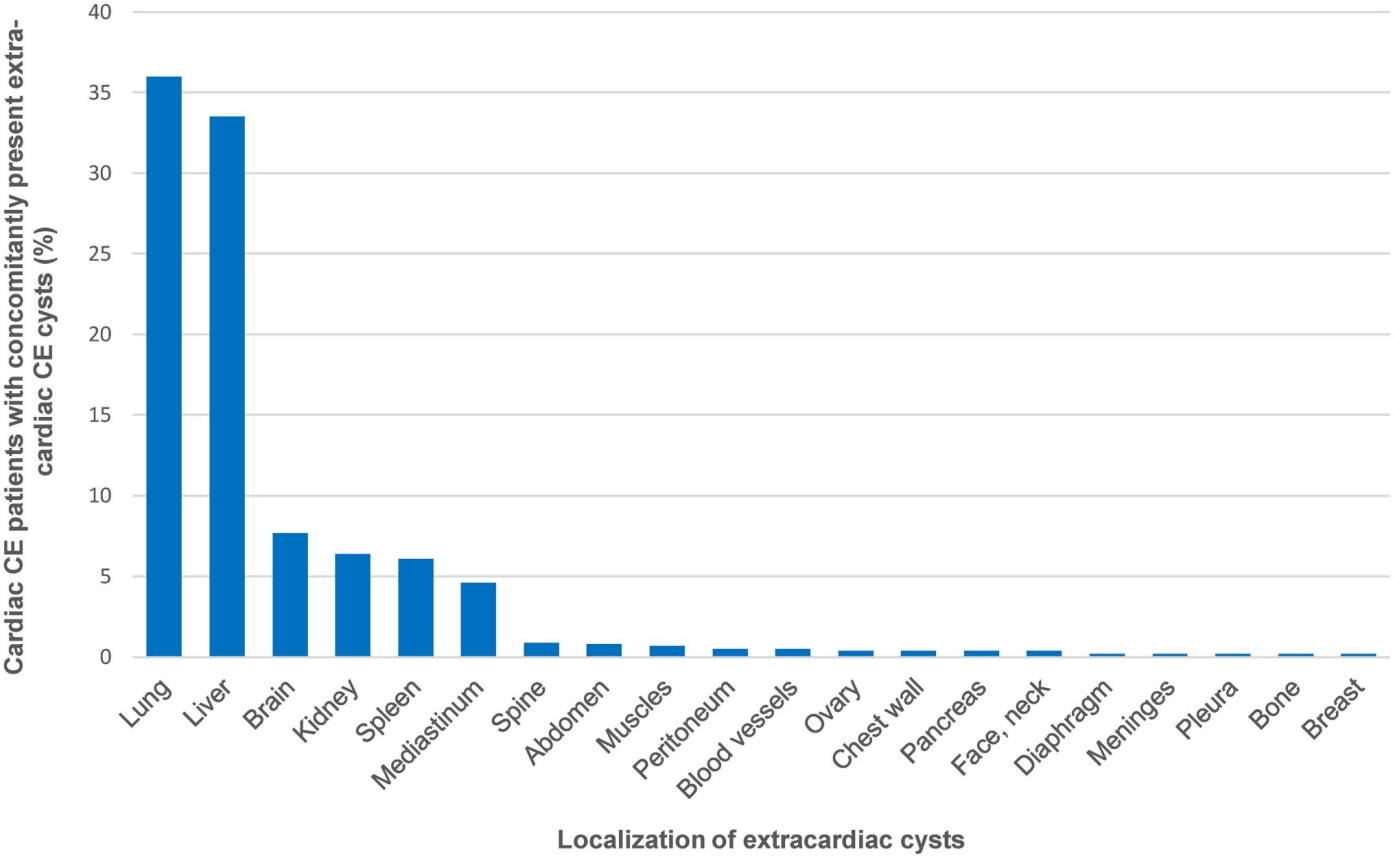
Anatomical distribution of extracardiac cysts in the cardiac echinococcosis cases with concomitantly present extracardiac involvement (n = 1095).

Data on the concomitant presence of extracardiac CE cysts was available for 1095 (90.5%) of the 1210 analysed cases. In 685 (62.6%) cases, the heart was the sole localization. Extracardiac cysts were present in 410 cases (37.4%). Fig 6 shows the anatomical distribution of these extracardiac cysts.

**Fig 7:**
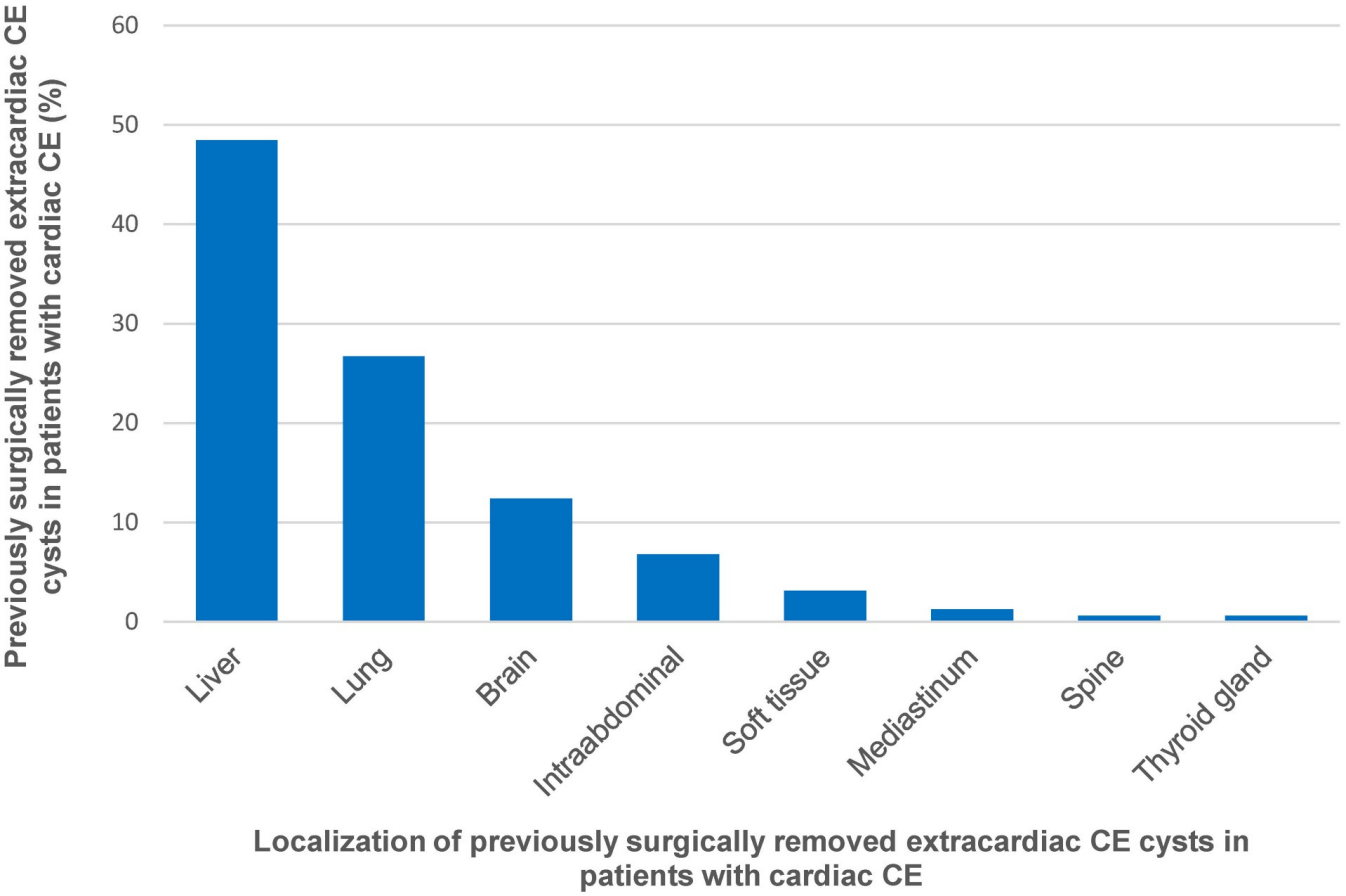
Anatomic localization of previously surgically removed extracardiac cystic echinococcal cysts in patients subsequently diagnosed with cardiac CE (n = 161).

Information on previous operations for extracardiac CE was available for 1008 (83.3%) of the 1210 analysed cases; 161 (16%) of these 1008 cases had a history of previous surgery for extracardiac CE. Data on the anatomical localization of the surgically removed extracardiac CE cysts pre-dating the diagnosis of cardiac CE was available for 161 cysts (Fig 7). Data on the time window between surgery for extracardiac CE and diagnosis of cardiac CE was available for 149 cases. The median time window was 5 years (range 0–55 years).

### Symptoms

**Fig 8:**
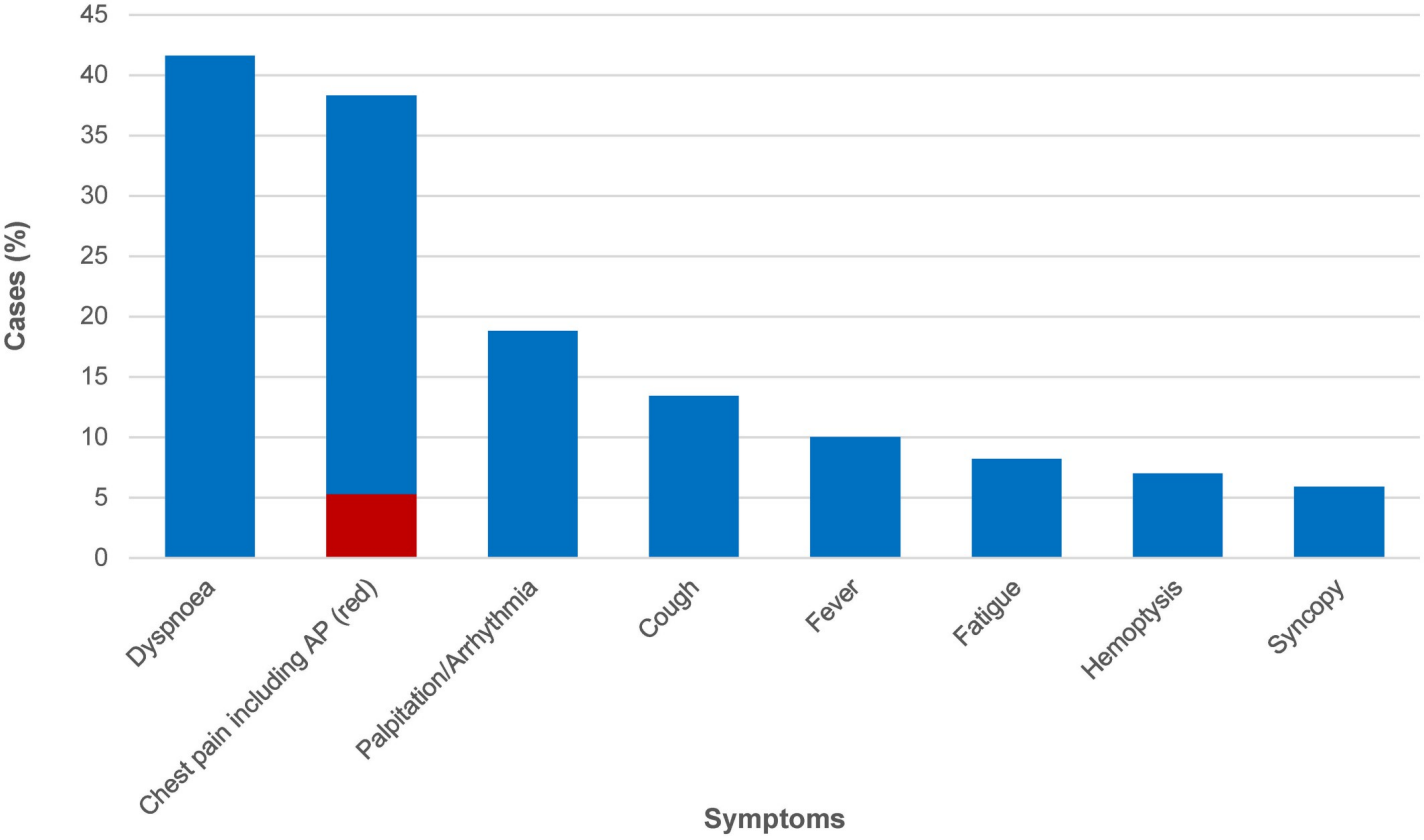
Frequency of symptoms in patients with unruptured cardiac echinococcal cysts at the time of diagnosis (n = 1010). AP, Angina pectoris (red bar segment); Note: due to the concomitant presence of more than one symptom per patient, the frequency of reported symptoms exceeds the number of patients.

Data on symptoms linked to unruptured cardiac CE at the time of diagnosis was available for 1010 (83.5%) cases. Symptoms were present in 835 (82.7%) of these cases (Fig 8).

### Acute complications

**Fig 9:**
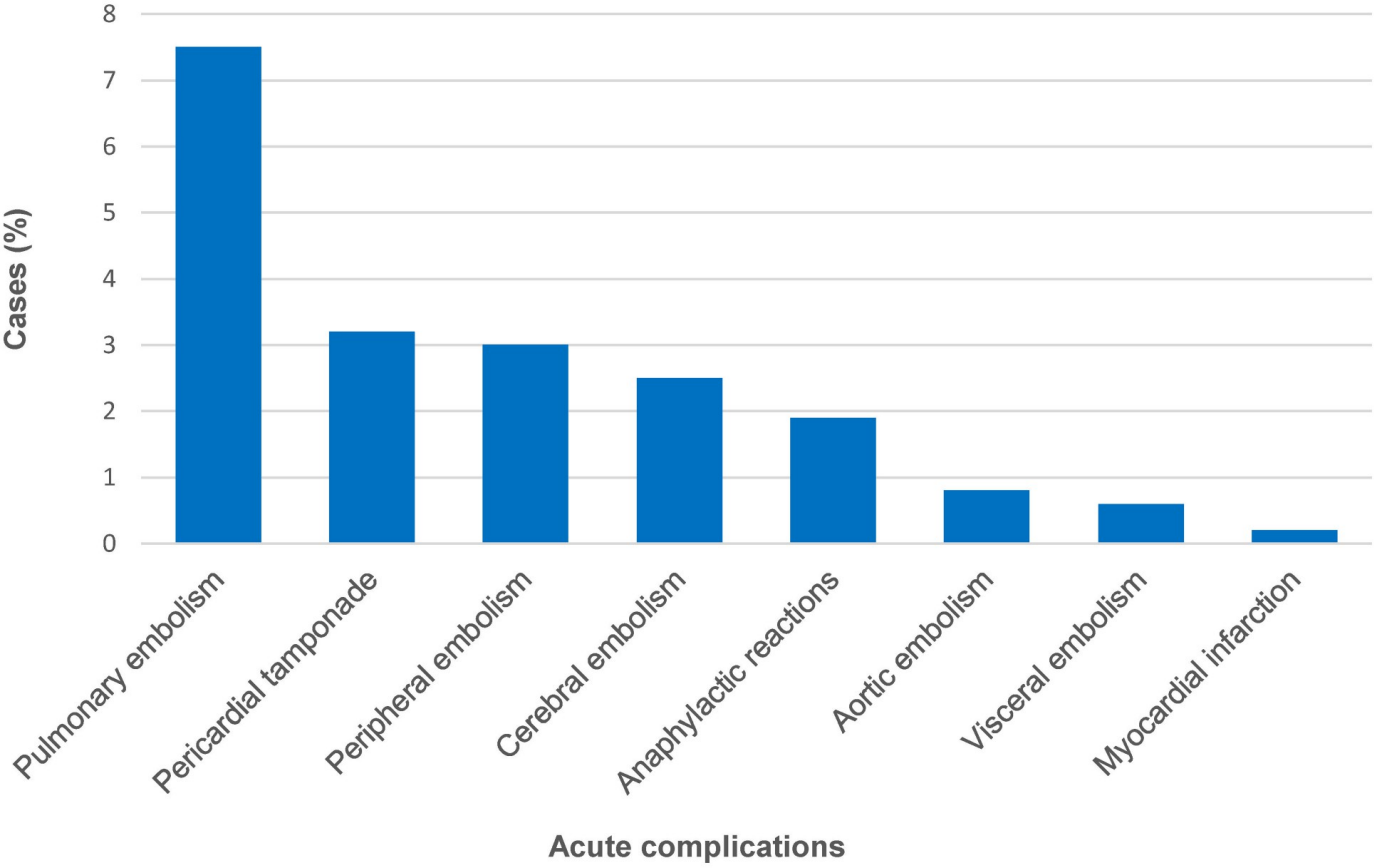
Spectrum of acute complications reported in patients with cardiac cystic echinococcosis (n = 191). Note: the number of reported acute complications exceeds the number of patients as in some patients more than one acute complication was reported.

Data on acute complications linked to cardiac CE was available for 1042 cases (86.1%). Acute complications occurred in 191 (18.3%) of these cases, were exclusively rupture-related, and consisted of three main pathologies: embolic events (n = 159), pericardial tamponade (n = 34), and anaphylactic reactions (n = 21) (Fig 9). In some cases, more than one complication was reported and, thus overall 83.2%, 17.8%, and 10.9% of cases suffered from embolic events, pericardial tamponade, and anaphylactic reactions, repectively.

The route of rupture was specified in 123 (64.4%) of the cases with reported cyst rupture. In 86 (69.9%) of the cases the cyst ruptured into the ventricular or atrial cavity, and in 37 (30.1%) into the pericardial cavitity. The exact rupture localization was reported in 58 of the cases: 27 cysts (46.6%) ruptured into the left ventricle, 16 (27.6%) into the right ventricle, 9 (15.5%) into the right atrium, 5 (8.6%) into the left atrium, and 1 septal cyst (1.7%) ruptured into both ventricles, causing a ventricular septal defect [15].

Symptoms caused by rupture-related emboli were the first leading to the diagnosis of cardiac CE in all cases with peripheral embolism, in 82.6% of cases with cerebral embolism/infarction, in 77.4% of cases with pulmonary embolism, and in 60% of cases with aortic embolism. In 88% of the patients with rupture-related pericardial tamponade, this was the first symptom leading to the diagnosis of cardiac CE. In 93.3% (14/15) of the cases reporting rupture-related anaphylactic reactions, the anaphylactic reaction was the first symptom leading to the diagnosis of cardiac CE. The sole other reported case of rupture-related anaphylaxis occurred during surgery for cardiac CE [16]. The type of anaphylactic reaction was specified in 14 of the 21 cases: 8 patients (57.1%) presented with hypotension or shock, 5 (35.7%) with pruritus, and 1 (7.1%) developed bronchospasm. Mirijello and colleagues reported the case of a 23-year-old man with Kounis syndrome, a rare form of acute coronary syndrome caused by an allergic reaction, which in this case was attributed to the rupture of a left ventricular echinococcal cyst [17].

### Diagnostics

Table 2 shows data on echinococcal serology, presence of eosinophilia (as reported by the authors) and reported use of electrocardiography (ECG) and imaging.

**Table 2:** Results of serology, eosinophilia, ECG and imaging techniques applied in patients with cardiac cystic echinococcosis.

**Performed diagnostics investigations**	**Positive/abnormal** **n (%)**	**Negative/normal** **n (%)**	**No data** **n**
**Serology**	495 (79.8)	125 (20.2)	590
**Blood eosinophilia**	172 (55.7)	137 (44.3)	901
**ECG**	518 (74.9)	174 (25.1)	518
	**Performed** **n (%)**	**Not performed** **n (%)**	**No data** **n**
**TTE**	875 (84.0)	167 (16.0)	168
**TEE**	123 (14.0)	753 (86.0)	334
**Cardiac CT**	524 (56.4)	405 (43.6)	281
**Cardiac MRI**	303 (34.0)	587 (66.0)	320
**Coronary angiography**	204 (22.3)	711 (77.7)	295

CE, cystic echinococcosis; ECG, electrocardiography; TTE, transthoracic echocardiography; TEE, transesophageal echocardiography; CT, computed tomography; MRI, magnet resonance imaging.

Tables 3 and 4 show the association between patient- and cyst-specific parameters and presence of eosinophilia by chi square testing and logistic regression analysis, respectively.

**Table 3:** Correlation of blood eosinophilia with patient and cyst specific parameters.

		Total^a^ n (%)	Eosinophilia present n (%)	Eosinophilia absent n (%)	p-value^c^
**All cases**		309 (100)	172 (55.7)	137 (44.3)	-
**Age**	<60 years	279 (90.3)	156 (90.7)	123 (89.8)	0.787
	≥60 years	30 (9.7)	16 (9.3)	14 (10.2)	
**Sex**	female	137 (44.8)	76 (44.7)	61 (44.9)	0.979
	male	169 (55.2)	94 (55.3)	75 (55.1)	
**Serology**	negative	49 (20.1)	17 (13.4)	32 (27.4)	**0.007**
	positive	195 (79.9)	110 (86.6)	85 (72.6)	
**Maximum cyst diameter**	<7 cm	161 (80.1)	94 (81.0)	67 (78.8)	0.698
	≥7 cm	40 (19.9)	22 (19.0)	18 (21.2)	
**Symptomatic disease**	no	40 (13.7)	18 (11.1)	22 (16.9)	0.151
	yes	252 (86.3)	144 (88.9)	108 (83.1)	
**Cyst rupture related complications** ^b^	no	240 (81.4)	128 (77.6)	112 (86.2)	**0.060**
	yes	55 (18.6)	37 (22.4)	18 (13.8)	

CE, cystic echinococcosis.

^a^ numbers may not add up to the total number due to missing data of the variables.

^b^ embolic events, pericardial tamponade, and anaphylactic reactions.

^c^ chi-square test.

**Table 4:** Factors associated with the presence of blood eosinophilia: Univariate and multivariate logistic regression model.

		Presence of blood eosinophilia in cardiac CE			
		OR (95% CI)	p-value	adjusted OR^a^ (95% CI)	p-value
**Age**	<60 years	1	0.787	-	
	≥60 years	0.90 (0.42–1.92)		-	
**Sex**	female	1	0.979	-	
	male	1.00 (0.64–1.58)		-	
**Serology**	negative	1	**0.008**	1	**0.009**
	positive	2.44 (1.27–4.68)		2.43 (1.25–4.71)	
**Maximum cyst diameter**	<7 cm	1	0.698	-	
	≥7 cm	0.87 (0.43–1.75)		-	
**Symptomatic disease**	no	1	0.154	-	
	yes	1.63 (0.83–3.19)		-	
**Cyst rupture related complications** ^b^	no	1	**0.063**	1	0.181
	yes	1.80 (0.97–3.34)		1.65 (0.79–3.44)	

CE, cystic echinococcosis; CI, confidence interval; OR, odds ratio.

^a^ adjusted for serology and presence of complications (the adjusted OR was only calculated if the OR showed a p-value <0.1)

^b^ embolic events, pericardial tamponade, and anaphylactic reactions.

Tables 5 and 6 show data on cyst stage and rupture-related complications in relation to the result of echinococcal serology and the presence of blood eosinophilia, respectively.

**Table 5:** Result of echinococcal serology and presence of blood eosinophilia according to cyst stage.

Cyst stage	Number of cases with data on cyst stage and CE-specific serology	Serology		Number of cases with data on cyst stage and blood eosinophilia	Blood eosinophilia	
		Positive n (%)	Negative n (%)		Present n (%)	Absent n (%)
**CE1**	24	16 (66.7)	8 (33.3)	16	7 (43.8)	9 (56.3)
**CE2**	39	32 (82.1)	7 (17.9)	25	15 (60)	10 (40)
**CE3A**	8	7 (87.5)	1 (12.5)	9	5 (55.6)	4 (44.4)
**CE3B**	1	1 (100)	0	2	1 (50)	1 (50)
**CE4**	2	2 (100)	0	0	–	–
**CE5**	7	6 (85.7)	1 (14.3)	3	2 (66.7)	1 (33.3)

CE, cystic echinococcosis.

**Table 6:** Correlation of rupture related complications with result of echinococcal serology and presence of blood eosinophilia.

Complication	Number of cases with data on complication and CE-specific serology	Serology		Number of cases with data on complication and blood eosinophilia	Blood eosinophilia	
		Positive n (%)	Negative n (%)		Present n (%)	Absent n (%)
**Embolic event**	61	53 (86.9)	8 (13.1)	36	23 (63.9)	13 (36.1)
**Cardiac tamponade**	15	11 (73.3)	4 (26.7)	14	11 (78.6)	3 (21.4)
**Anaphylactic reaction**	8	7 (87.5)	1 (12.5)	6	5 (83.3)	1 (16.7)

CE, cystic echinococcosis.

### Treatment

Data on the treatment modality and the used drug(s) were available for 1120 and 990 cases, respectively (Table 7).

**Table 7:** Treatment modalities (n_total_ = 1120 cases) and drugs (n_total_ = 990 cases) used in cardiac CE.

Treatment modality and drugs used	All cases with available data n (%)	Breakdown of cases by time period^a^ n (%)	
		1965–2010	2011–2022
**Surgery only**	463 (41.3)	378 (50.4)	85 (23.0)
**Surgery + medical treatment**	503 (44.9)	274 (36.5)	229 (61.9)
**Medical treatment only**	85 (7.6)	49 (6.5)	36 (9.7)
**No treatment**	69 (6.2)^b^	49 (6.5)	20 (5.4)
**Albendazole alone**	558 (56.4)	378 (50.4)	180 (57.7)
**Mebendazole alone**	360 (36.3)	274 (36.5)	86 (27.6)
**Praziquantel alone**	66 (6.7)	49 (6.5)	17 (5.4)
**Albendazole + Praziquantel**	6 (0.6)	0	6 (1.9)

^a^ data is shown for two time periods (before and after 2010) because, after an initial indication in 1996 [16], the most recent recommendations for the concomitant use of albendazole as a prophylaxis for any invasive procedure for CE were published in 2010 [6].

^b^ the reason for not giving medical treatment was stated in 16 cases: 4 patients were diagnosed with cardiac CE only after their death [18–21], 4 patients refused therapy [22–25], 3 patients died before therapy could be started [26–28], 2 patients died during surgery [16,23], 2 patients were diagnosed with inactive cysts and a watch and wait strategy was chosen [29,30], and in 1 patient albendazole had to be stopped because of hepatotoxicity [31].

Data on the number of cardiac surgical interventions were available for 1054 (87.1%) cases. In 175 of these cases (16.6%) no surgery was performed, in 862 (81.8%) a single operation was performed, in 15 (1.4%) 2 operations, and in 2 cases (0.2%) 3 operations were performed. The indication for re-operation was stated for 12 cases: bleeding complication (n = 5), constrictive pericarditis (n = 3), local recurrence (n = 3), and incomplete resection (n = 1).

The intraoperative use of a protoscolecidal solution was reported in 394 cases. The different protoscolecidal solutions used are listed in Table 8.

**Table 8:** Scolecidal solutions used during surgery of cardiac CE (n = 394).

Protoscolecidal solution used	Number of cases n (%)
Hypertonic saline	276 (70.1)
Hypertonic saline + iodine	11 (2.8)
Hypertonic saline + ethanol	8 (2.0)
Hypertonic saline + formalin	2 (0.5)
Hypertonic saline + ethanol + iodine	1 (0.3)
Iodine	50 (12.7)
Formalin (formaldehyde)	13 (3.3)
Silver nitrate	9 (2.3)
Hydrogen peroxide	6 (1.5)
Hypertonic glucose	6 (1.5)
Cetrimide	5 (1.3)
Ethanol	3 (0.7)
EthanoI + iodine	2 (0.5)
Formic acid^a^	1 (0.3)
Ethacridine lactate^a^	1 (0.3)

^a^ protoscolecidal property unclear

Whether complete or incomplete resection of the parasitic lesion(s) was performed was reported for 257 cases (22.7%). Complete resection was performed in 137 cases (53.3%) and incomplete resection in 120 cases (46.7%).

### Outcome

Data on the outcome was reported for 770 patients (63.6%): 659 patients (84.9%) were reported to have survived, 86 (11.1%) to have died due to complications directly related or attributable to cardiac CE, and 31 (4.0%) to have died due to (primarily postoperative) complications not directly related or attributable to cardiac CE.

Data on follow-up length was available for 568 cases, median 9 months (range 0–288). Local recurrence of cardiac CE after surgery was reported in 9 cases, median 12 months (range 1–108) after surgery. The previous resection was described as complete in one case and incomplete in another case, while in the remaining 7 cases no information was provided on the completeness of cyst resection.

Putatative secondary CE after surgery was reported in 19 cases, which was diagnosed median 18 months (range 1–72) after surgery. The anatomical location of the secondary CE lesions was reported for 18 patients (Table 9).

**Table 9:** Anatomical location of metastatic/secondary CE lesions (n = 25) after cardiac CE surgery of 18 patients.

Anatomic location of metastatic/secondary CE lesions following surgery for cardiac CE	Patients n (%)
Lung	11 (42.3)
Brain	4 (15.4)
Thoracic wall	3 (11.5)
Kidney	2 (7.7)
Liver	2 (7.7)
Abdominal wall	1 (3.8)
Mediastinum	1 (3.8)
Pulmonary artery	1 (3.8)
Spleen	1 (3.8)

CE, cystic echinococcosis.

Note: the total number of anatomic locations exceeds the number of patients because some patients had more than one metastatic CE lesion.

## Discussion

There is a clear disparity between the global pattern of CE distribution and the pattern of publications on cardiac CE (Fig 3). Since this pattern cannot be explained by the local prevalence of the disease alone, the most likely explanation for this discrepancy is different academic traditions of medical publishing. Whether different *E*. *granulosus s*.*l*. genotypes may possibly show differences in organ tropism and, thus influence the incidence of cardiac CE in certain regions, is not inferable from the data of this review, since no data about parasite genotyping were available in the reviewed publications.

Cardiac CE appears to be most commonly diagnosed in adulthood, with a slight predominance of males, similar to what is seen in spinal CE [32]. However, the identified age and sex distribution may not necessarily be generalizable or applicable to specific local situations, where age or sex distributions may differ according to local exposure patterns, access to medical care or other factors.

Our understanding of the anatomical distribution pattern and organ-specific frequency of CE is largely coming from reported cohort data. However, these data are skewed by publication bias, with unusal cases more likely to be published. Overall, the observed anatomic distribution pattern of cardiac CE cysts suggests that cyst location is primarily determined by the proportional coronary blood flow supplying the different heart regions. An interesting observation is that cardiac CE patients with concomitantly present extracardiac cysts have slightly more pulmonary than hepatic cysts (Fig 6) which is markedly different from the general distribution pattern of liver and lung cysts in CE, where the liver is affected in 73.4% and the lungs in 19.6% of CE cases [2]. A similar observation in patients with spinal CE [32] as well as our finding that CE solely affecting the heart is a rather frequent presentation (62.6% [685/1095] of the reviewed cardiac CE cases) raises the question whether cardiac (as well as pulmonary) CE may possibly be the result of an inhalative route of infection [5]. Such a hypothesis was attempted to be tested in 1965 in a study in sheep in which pulmonary echinococcal cysts were reported following iatrogenic infection with *E*. *granulosus* via the tracheal route [33]. However, considering that coronary blood flow accounts for only about 4–5% of cardiac output [34], one would generally expect the likelihood of oncospheres from the pulmonary circulation passing the heart and embolizing in the coronary capillary bed to be relatively low and the proportion of extracardiac manifestations resulting from this transmission route to be much higher. To date, an inhalative route of infection remains speculative. It will be interesting to see whether future research will find explanations for these observations as well as biological clues on which factors determine the implantation of onchospheres in different tissues.

We found that 16% (161/1008) of cardiac CE cases had a history of previous surgery for extracardiac CE. Whether or to what extent these cases may represent secondary cardiac CE due to iatrogenic seeding remains unclear. None of the publications reviewed contained data on the genomic fingerprinting of cysts and, to our knowledge, no such studies have been conducted to date.

Unless acute complications occur, symptoms of cardiac CE are nonspecific (Fig 8) and, due to the slow development of CE cysts, rarely acute. At the time of diagnosis, most patients had a prolonged history of symptoms that have been present for weeks or months. The most prominent of these symptoms are dyspnea and chest pain, which appear to be due to impaired cardiac muscle function and decreased cardiac output due to the mechanical disturbance caused by the cysts [13]. After dyspnoea and chest pain, palpitations are the third most frequently mentioned symptom. Palpitations are caused by ventricular extrasystoles, paroxysmal ventricular tachycardia episodes, and atrioventricular conduction disorders. Among the latter, atrioventricular blocks leading to bradycardia and Adam-Stokes seizures are particularly notable and typically caused by CE cysts in the interventricular septum [13].

Acute complications are generally cyst rupture-related (Fig 9). Abishek and Avinash postulated that because of the lower intramyocardial pressure conditions, cysts of the right ventricle are more likely to grow subendocardially, are more likely to project into the ventricular system, and consequently more likely to rupture intraventricularly [14]. Our data show a ratio of left ventricular to right ventricular cysts of 2.1:1 (475/226) and a ratio of ruptured left ventricular to right ventricular cysts of 1.7:1 (27/16). This suggests that if a difference in the likelihood of rupture between right and left ventricular cysts actually exists, the difference would probably be rather small. Abishek and Avinash also postulated that cases of pericardiac CE usually results from ruptured cardiac cysts [14]; however, we found that in 57.7% (94/163) of pericardial CE cases no concomitant myocardial involvement was traceable.

The rupture of a cyst into the pericardial cavity may be asymptomatic or cause acute pericarditis, which may progress into tamponade or constrictive pericarditis. The tamponade in this case is not primarily caused by the amount of fluid contained in the ruptured cyst, but by the prominent exudative reaction of the serous membrane of the pericardium [13,35]. Chest pain in pericardial CE may be related to immune-mediated pericarditis or result from compression of coronary arteries by growing CE cysts within the pericardium. The latter manifests with ischaemia-typical angina pectoris-like symptoms [14]. Other mechanisms by which cardiac CE may present with acute coronary syndrome are exceptionally rare. Myocardial infarction secondary to rupture of a cyst into the left ventricle and embolisation of cyst material into the coronary arteries was reported in only 2 of 1095 cases. Also, the rare entity of Kounis syndrome, a rare form of acute coronary syndrome caused by an allergic reaction, was described in only one case, in which the allergic reaction was attributed to rupture of a left ventricular echinococcal cyst [17].

As in CE in all other localizations, the diagnosis of cardiac CE is made using imaging techniques, with echocardiography being the central method in almost all published cases (Table 2). The value of other imaging modalities can only be roughly extrapolated, as the availability of these modalities has changed over time and often continues to vary geographically. The additive use of CT, transthoracic echocardiography (TTE) and transoesophageal echocardiography (TEE) has been reported to incresase sensitivity [5,36]. Due to the high spatial resolution and the characteristic morphology of CE cysts, cross-sectional imaging by CT and MRI may provide valuable additional information and be helpful with regard to surgical planning [37–39]. Intraoperatively performed TEE may be a valuable tool to guide surgery and preoperatively performed coronary angiography may also be indicated [40]. Due to the high frequency of concomitant pulmonary and hepatic cysts, all cardiac CE cases should be investigated accordingly.

Electrocardiography (ECG) shows a high rate of abnormal, albeit unspecific, findings in cardiac CE. The ECG changes vary depending on the location of the cysts with intramyocardial cysts depicting inert electrical windows causing respective ECG alterations [13,41]. Overall, three quarters of reviewed cases were reported to have abnormal ECG findings (Table 2).

The differential diagnoses of cardiac CE cysts are primarily cardiac tumors. There have been repeated reports of cases where the diagnosis of myxoma or cardiac CE cyst have been confused [42–47]. Although usually solid, cardiac tumours may have cystic or multicystic components, favouring further confusion with cardiac CE cysts [48,49]. Considering that cardiac myxoma and other more rare primary cardiac tumors and cardiac metastases are extremely uncommon (various postmortem studies report rates between 0.001 and 0.28%) [48], it may be speculated that in CE high endemic areas, the frequency of cardiac CE may be higher than the incidence of cardiac malignancy. Other rare differential diagnoses include congenital pericardial cysts [50], intracardiac thrombosis [51], and aneurysm [52].

Blood eosinophilia is a classically observed laboratory abnormality in tissue-invasive parasitic diseases, although its presence and extent vary widely. We found a statistically significant association of eosinophilia with positive serology and a tendency towards statistical significance of eosinophilia and cyst-rupture related complications (Tables 3 and 4). This is in accordance with previous data [53] and is compatible with eosinophilic reaction and antibody production being stimulated upon loss of cyst wall integrity [54]. However, even in the case of cyst rupture, blood eosinophila and positive serology are absent in a considerable number of patients (Table 6). Since blood eosinophilia also largely lacks specificity, the presence or absence of eosinophilia has no relevant predictive diagnostic value in CE [55]. Similarly, the predictive diagnostic value of serology is limited: a negative serology does not exclude the diagnosis of CE, while a positive serology, applied after the visualization of a CE-compatible lesion, may support the diagnosis of CE [56].

With regard to the therapeutic approach, the management of cardiac CE differs from the stage-specific treatment approach established for hepatic CE. While in abdominal CE, asymptomatic, uncomplicated, inactive cysts (CE4, CE5) are managed by a watch-and-wait approach, any cardiac CE cyst is generally considered an indication for surgical removal. This is because the mere presence of a cyst is considered to impair cardiac performance, predisposing to long-term sequelae [5]. The sole exception in this respect has been formulated by Thamer and colleagues: "*if the cyst is small*, *completely calcified*, *asymptomatic and without adverse effects on haemodynamics or blood supply…*" [13]. In line with the generally advocated surgical approach, all cases reviewed that did not undergo surgery either had contraindications or did not consent to surgery.

The vast majority of cardiac CE cases appear to be sufficiently treated by a single surgical procedure.

Since the spectrum of cardiac CE cases is broad, the differences in availability and the evolution of surgical techniques over time and at different centres vary considerably, and details regarding the applied surgical techniques were often poorly reported, we did not attempt a further analysis of these aspects. In the absence of clinical trials, the best available recommendations regarding the surgical management of cardiac CE come from centres reporting their experiences in larger case series (e.g. n = 41 [5]; n = 45 [13]). These publications also specify and summarize the surgical principles applying to cardiac CE [5,13].

52.5% of the cases reviewed received antiparasitic treatment (Table 7), but reported data on specific peri- or postoperative intake, dosage, and duration were largely insufficient to allow a more detailed evaluation. Although data are scarce, it seems reasonable to recommend, as for lung CE, that prolonged preoperative medical treatment should be avoided, to reduce the periinterventional risk of cyst rupture as the consequence of drug-induced damage of the cyst wall.

Hypertonic saline was the protoscolecidal solution used most commonly to protect the surgical field and sterilise the cyst prior to surgical removal (Table 8). This is in line with the current treatment recommendations for CE primarily recommending the use of 20% hypertonic saline [6]. Although the effectiveness of hypertonic saline is established [57], its use is not free of risk, as documented by reports on chemical sclerosing cholangitis [58] and even fatal cases of hypernatremia [59,60]. In addition, the number of publications reporting such complications is likely to be biased, as clinicians are generally not too keen to publish such results. The use of 95% ethanol, a protoscolicidal solution frequently used in percutaneous treatment (PAIR procedure) of hepatic CE [6], was rarely reported. This might be due to the experience of cardiac surgeons with alcohol septal ablation for the treatment of hypertrophic obstructive cardiomyopathy, in which a controlled local reduction of myocardial tissue by ethanol-induced necrosis is sought [61]. In the treatment of cardiac CE, myocardial necrosis is not desired, therefore the use of ethanol, a myotoxic substance, should be avoided here. The rarely reported use of formalin (formaldehyde) is considered obsolete due to its tissue toxicity. In one case each, the use of formic acid and ethacridine lactate as protoscolecidal solutions was reported. Both compounds have antiseptic properties, but it is unclear whether they have a protosocolecidal effect, which is why their use cannot be recommended. In the future, the intraoperative use of topical scolicidal solutions may possibly be replaced by preoperatively administered peroral praziquantel, a potent protoscolicidal agent, as recently shown by Richter and colleagues [62,63].

Data on the outcome of cardiac CE is scarce and derive from few case series. Overall, we found a mortality rate of ~15% among the cardiac CE cases reviewed. However, this figure should be interpreted with caution because these cases were reported over a period of seven decades from a variety of facilities with very different expertise and resources, not to mention publication bias. Thameur and colleagues, who published one of the largest cardiac CE cohorts (n = 45), reported that the case fatality rate of cardiac CE in their centre dropped from 67% in the 1970s to 5.5% in 2001 [13]. Thus, it could be speculated that the overall case fatality rate of cardiac CE may be as low as 5.5% in centres with considerable expertise and resources but may be considerably higher in settings with limited expertise and resources.

The median time span between surgery of cardiac CE and the occurrence of local recurrence or putative secondary CE were 12 months (range 1–108) and 18 months (range 1–72), respectively. As the median time of follow up across all reviewed cases was only 9 months (0–288), it is very likely that the actual number of cases with local recurrence or secondary CE is higher than the numbers we obtained.

In summary, data on cardiac CE remain limited and, due to its overall rarity, prospective data are unlikely to become available any time soon. As is the case with other rare and neglected diseases, it is to be hoped that in the future data from multicentric registries will allow systematically and prospectively filling the current knowledge gaps and defining best practice recommendation for the management of cardiac CE. The establishment of the European Registry of Cystic Echinococcosis [64] documents that efforts in this direction are feasible and underway. Box 1 summarizes the main conclusions we have drawn from our review and analysis of cardiac CE, and Box 2 contains our selection of publications that we recommend clinicians encountering cardiac CE to read.

**Box 1**

- the frequency of primary cardiac CE gives rise to speculation about the possible role of an inhalation route of infection.
- symptoms of cardiac CE are non-specific and acute complications are often the first clinical manifestation. Complications are generally rupture-related and include embolic events, pericardial tamponade, and anaphylactic reactions.
- as with other organ localizations, the diagnosis of cardiac CE is made by imaging. Serology plays a minor, although supportive, role due to its limited sensitivity.
- unlike in other anatomic localizations, cardiac CE cysts in all stages should be surgically removed (with possibly selected exceptions) as their presence is considered to impair cardiac performance leading to longterm sequelae.
- surgically treated cardiac CE patients should be followed up for at least 10 years for the occurrence of local recurrence or iatrogenic secondary CE.

**Box 2**

1. Birincioglu CL, Kervan U, Tufekcioglu O, Ozen A, Bardakci H, Kucuker SA, Saritas A. Cardiac echinococcosis. Asian Cardiovasc Thorac Ann. 2013;21(5):558–65.
2. Thameur H et al. Cardiopericardial Hydatid Cysts. World J. Surg. 2001;25:58–67.
3. Brunetti E, Kern P, Vuitton DA. Expert consensus for the diagnosis and treatment of cystic and alveolar echinococcosis in humans. Acta Trop. 2010;114(1):1–16.

## Supporting information

S1 Text Reference list of all included and excluded publications. (DOCX)

S1 Table Raw data master table. (XLSX)
