# Supplementary material for: Cardiac cystic echinococcosis—A systematic review and analysis of the literature
Source: PLoS Negl Trop Dis. 2024 May 30;18(5):e0012183. doi: 10.1371/journal.pntd.0012183 (PMC11139302; doi:10.1371/journal.pntd.0012183)
Supplement: S1 Text — (DOCX) [file pntd.0012183.s001.docx]

**Supplementary file S1**

**Reference list of all included and excluded publications (Referring to Fig 2)**


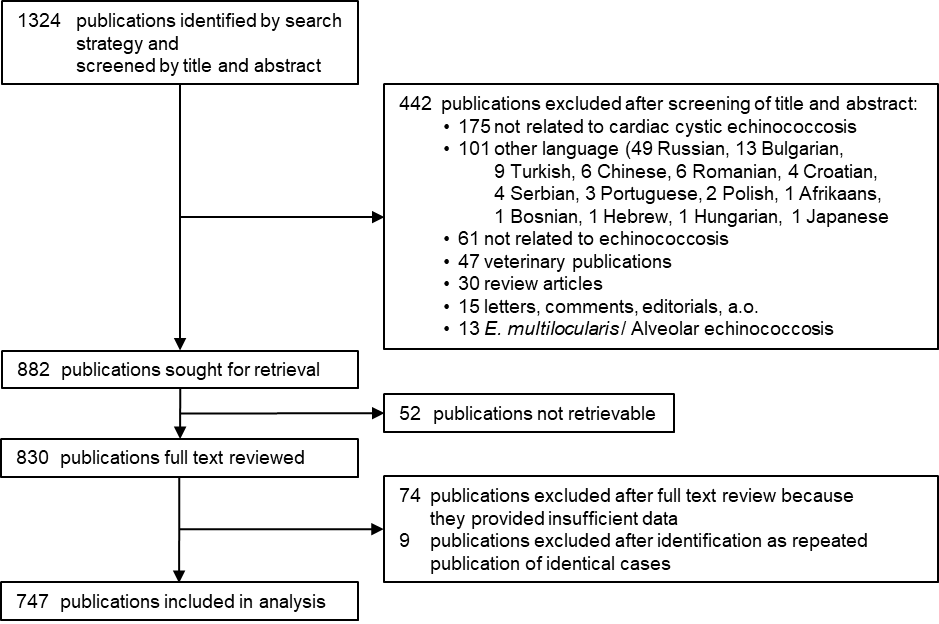


**Included publications (N) Reference N°**

747 [1-747]

**Excluded publications (N / reason for exclusion)**

175 Publications not related to cardiac cystic echinococcosis [748-922]
101 Publications in other languages [923-1023]
61 Publications not related to echinococcosis [1024-1084]
47 Veterinary publications [1085-1131]
30 Review articles [1132-1161]
15 Letters, comments editorials, a.o. [1162-1176]
13 Publications concerning *E. multilocularis* / alveolar echinococcosis [1177-1189]
52 Publications not retrievable [1190-1241]
74 Publications excluded after full text review because they provided

insufficient data [1242-1315]

## 9 Publications excluded after identification as repeated publication

## of identical cases [1316-1324]

**References**

1. Acikel S, Kiziltepe U, Turkvatan A, Cakici M, Koroglu DB, Sahpaz A, et al. Intracavitary cardiac hydatid cysts with a high risk of thromboemboli. Invasive nature of cardiac echinococcosis. Herz. 2014;39(7):882-6. Epub 2013/08/29. doi: 10.1007/s00059-013-3932-x. PubMed PMID: 23982833.

2. Aggeli C, Koudounis P, Felekos I, Zisimos K, Angelis A, Alexopoulos N, et al. A multimodality approach of a cardiac echinococcus cyst in an asymptomatic woman. Int J Cardiol. 2016;202:874-6. Epub 2015/10/18. doi: 10.1016/j.ijcard.2015.10.030. PubMed PMID: 26476046.

3. Aghajani H, Yazdani S, Forouzan Nia SK, Vahidi H, Aghajani F. Dog Footprint in the Heart. J Tehran Heart Cent. 2016;11(4):198-202. Epub 2017/05/13. PubMed PMID: 28496513; PubMed Central PMCID: PMCPMC5424844.

4. Agnino A, Lanzone AM, Spira G, Anselmi A. Surgical treatment of left ventricular echinococcosis through the HeartPort technique. Interact Cardiovasc Thorac Surg. 2018;26(2):357-9. Epub 2017/10/20. doi: 10.1093/icvts/ivx290. PubMed PMID: 29049663.

5. Aksu U, Kalkan K. Pericardial cyst hydatid rupture complicated by pericardial tamponade. Int J Cardiol. 2015;193:62-3. Epub 2015/05/26. doi: 10.1016/j.ijcard.2015.05.066. PubMed PMID: 26005180.

6. Alshehri HZ, Lawand S, Omar AH, Koudieh MS. Surgical approach to huge cardiac hydatid cyst of the interventricular septum. Asian Cardiovasc Thorac Ann. 2014;22(9):1093-5. Epub 2014/06/03. doi: 10.1177/0218492313492745. PubMed PMID: 24887847.

7. Altas O, Sarikaya S, Saci H, Yerlikhan O, Kirali K. Cardiac cystic echinococcosis: Report of three cases. Asian Pac J Trop Med. 2014;7(11):922-4. Epub 2014/12/03. doi: 10.1016/s1995-7645(14)60163-2. PubMed PMID: 25441996.

8. Aslan AN, Bastug S, Turinay ZS, Bozkurt E. Case images: a demonstrative cardiac hydatid cyst in the interventricular septum. Turk Kardiyol Dern Ars. 2015;43(3):318. Epub 2015/04/24. doi: 10.5543/tkda.2015.46529. PubMed PMID: 25906009.

9. Bayramov F, Emet S, Dadashov M, Umman B, Bugra Z. A case of an atypically located cardiac hydatid cyst. Anatol J Cardiol. 2015;15(9):E24-5. Epub 2015/10/02. doi: 10.5152/AnatolJCardiol.2015.6548. PubMed PMID: 26424637; PubMed Central PMCID: PMCPMC5368507.

10. Beedkar A, Parikh R, Deshmukh P. Asymptomatic Presentation of Large Cardiac Hydatid. J Assoc Physicians India. 2017;65(2):98-9. Epub 2017/05/01. PubMed PMID: 28457048.

11. Beheshtirouy S, Kakaei F, Oliaei-Motlagh M, Teimouri-Dereshki A, Jalilzadeh-Binazar M. Syncope in a patient with a large left ventricular hydatid cyst: an unusual presentation. Int J Cardiol. 2014;172(3):e385-6. Epub 2014/01/28. doi: 10.1016/j.ijcard.2013.12.283. PubMed PMID: 24461965.

12. Ben Khalfallah A, Ben Slima H. [Cardiac hydatid cyst. Which imaging modality for an accurate diagnosis?]. Ann Cardiol Angeiol (Paris). 2017;66(2):102-8. Epub 2017/03/28. doi: 10.1016/j.ancard.2016.12.008. PubMed PMID: 28342525.

13. Blanco M, Echevarria JR, Fernandez-Gutierrez M, Laguna G. Heart Failure for Superinfected Giant Pericardial Hydatid Cyst. Ann Thorac Surg. 2017;103(2):e197. Epub 2017/01/23. doi: 10.1016/j.athoracsur.2016.09.039. PubMed PMID: 28109390.

14. Bogdanovic A, Radojkovic M, Tomasevic RJ, Pesic I, Petkovic TR, Kovacevic P, et al. Presentation of pericardial hydatid cyst as acute cardiac tamponade. Asian J Surg. 2017;40(2):175-7. Epub 2014/01/08. doi: 10.1016/j.asjsur.2013.10.001. PubMed PMID: 24393813.

15. Bouassida I, Pricopi C, Mangiameli G, Arame A, Auliac JB, Gorbatai B, et al. [Cardiac compression of hydatid origin]. Rev Pneumol Clin. 2018;74(4):248-52. Epub 2018/05/22. doi: 10.1016/j.pneumo.2018.03.009. PubMed PMID: 29779892.

16. Carita P, Verdecchia M, Ferro G, Buccheri D, Corrado E, Novo S, et al. Multimodality imaging in cardiac echinococcosis for diagnosis and follow-up of an untreatable cyst. Int J Cardiol. 2016;221:468-70. Epub 2016/07/15. doi: 10.1016/j.ijcard.2016.06.317. PubMed PMID: 27414724.

17. Carmona P, Alonso J, Aparicio S, Zarragoikoetxea I, Ibanez F, Argente P. Cardiac Hydatid Disease: An Uncommon Cause of Cardiac Tumors. J Cardiothorac Vasc Anesth. 2017;31(2):675-7. Epub 2016/08/25. doi: 10.1053/j.jvca.2016.04.016. PubMed PMID: 27554223.

18. Celik M, Yildirim E, Yuksel UC, Bozlar U, Karabacak K, Erol G. The Multimodality Imaging of Primary Cardiac Hydatid Cyst Located in the Right Ventricular Free Wall. Echocardiography. 2016;33(8):1269-71. Epub 2016/05/10. doi: 10.1111/echo.13252. PubMed PMID: 27159905.

19. Charfeddine S, Mallek S, Gueldiche M, Triki F, Jmaa HB, Frikha I, et al. A huge cardiac hydatid cyst: An unusual cause of chest pain revealing multivisceral hydatidosis in a young woman. J Saudi Heart Assoc. 2015;27(4):286-91. Epub 2015/11/12. doi: 10.1016/j.jsha.2015.04.003. PubMed PMID: 26557748; PubMed Central PMCID: PMCPMC4614895.

20. Chatzidakis E, Zogopoulos P, Paleologos TS, Papageorgiou N. Surgical Planning for the Treatment of a Patient with Multiple, Secondary, Intracranial Echinococcal Cysts. Surg J (N Y). 2016;2(1):e7-e10. Epub 2015/12/18. doi: 10.1055/s-0035-1570317. PubMed PMID: 28824975; PubMed Central PMCID: PMCPMC5553456.

21. Das T, Ozer M, Yagmur G, Yildirim M, Ozgun A, Demirel H. Hydatid Disease Involved in the Heart, Liver, and Kidney That Caused Sudden Death: Case Report. Am J Forensic Med Pathol. 2015;36(4):265-7. Epub 2015/09/12. doi: 10.1097/paf.0000000000000200. PubMed PMID: 26355853.

22. Dasbaksi K, Haldar S, Mukherjee K, Mukherjee P. A rare combination of hepatic and pericardial hydatid cyst and review of literature. Int J Surg Case Rep. 2015;10:52-5. Epub 2015/03/26. doi: 10.1016/j.ijscr.2015.02.052. PubMed PMID: 25805610; PubMed Central PMCID: PMCPMC4429948.

23. Demirel M, Toprak C, Tabakci MM, Ocal L. Case images: Cardiac hydatid cyst presenting as double-chambered left ventricle. Turk Kardiyol Dern Ars. 2016;44(4):350. Epub 2016/07/04. doi: 10.5543/tkda.2016.36797. PubMed PMID: 27372624.

24. El Kharras A, Atmane M, El Fenni J, Chaouir S, Amil T. [Cardiac and renal hydatid disease--contribution of imaging: report of a case]. Pan Afr Med J. 2014;18:153. Epub 2014/11/25. doi: 10.11604/pamj.2014.18.153.2410. PubMed PMID: 25419291; PubMed Central PMCID: PMCPMC4236841.

25. El Malki H, Chetoui A, Fenane H, Benyoussef H, Rhissassi J, Sayah R, et al. Intracavitary hydatid cyst of the left ventricle in child: an emergency surgery case report. Pan Afr Med J. 2014;19:401. Epub 2014/01/01. doi: 10.11604/pamj.2014.19.401.5770. PubMed PMID: 25995797; PubMed Central PMCID: PMCPMC4430140.

26. Elkarimi S, Ouldelgadia N, Gacem H, Zouizra Z, Boumzebra D, Blelaabidia B, et al. [Tamponade reveals an intra-pericardial hydatid cyst - a case report]. Ann Cardiol Angeiol (Paris). 2014;63(4):267-70. Epub 2012/03/23. doi: 10.1016/j.ancard.2012.01.003. PubMed PMID: 22436634.

27. Epcacan S, Ramoglu MG, Ozdemir F. Isolated cardiac hydatid cyst mimicking myocardial ischaemia in a female patient. Cardiol Young. 2018;28(10):1160-1. Epub 2018/08/08. doi: 10.1017/s1047951118000653. PubMed PMID: 30081969.

28. Fennira S, Sarray H, Kammoun S, Zoubli A, Kammoun Y, Kraiem S, et al. A large cardiac hydatid cyst in the interventricular septum: A case report. Int J Infect Dis. 2019;78:31-3. Epub 2018/09/16. doi: 10.1016/j.ijid.2018.09.004. PubMed PMID: 30218815.

29. Fiengo L, Bucci F, Giannotti D, Patrizi G, Redler A, Kucukaksu DS. Giant cardiac hydatid cyst in children: case report and review of the literature. Clin Med Insights Case Rep. 2014;7:111-6. Epub 2014/09/25. doi: 10.4137/CCRep.S15862. PubMed PMID: 25249763; PubMed Central PMCID: PMCPMC4167224.

30. Gencheva DG, Menchev DN, Penchev DK, Tokmakova MP. An Incidental Finding of Heart Echinococcosis in a Patient with Infective Endocarditis: a Case Report. Folia Med (Plovdiv). 2017;59(1):110-3. Epub 2017/04/07. doi: 10.1515/folmed-2017-0017. PubMed PMID: 28384109.

31. Gocen U, Atalay A, Basturk Y, Topcuoglu MS, Yaliniz H, Salih OK. Urgent surgery for cardiac hydatid cyst located in interventricular septum. Asian Cardiovasc Thorac Ann. 2014;22(8):965-7. Epub 2014/06/03. doi: 10.1177/0218492313489562. PubMed PMID: 24887829.

32. Gunay N, Ocal L, Aksoy S, Baktir AO, Keser N. A Giant Isolated Cardiac Hydatid Cyst in the Interventricular Septum. Korean Circ J. 2017;47(3):418-9. Epub 2017/06/02. doi: 10.4070/kcj.2016.0248. PubMed PMID: 28567094; PubMed Central PMCID: PMCPMC5449538.

33. Gurzu S, Beleaua MA, Egyed-Zsigmond E, Jung I. Unusual Location of Hydatid Cysts: Report of Two Cases in the Heart and Hip Joint of Romanian Patients. Korean J Parasitol. 2017;55(4):429-31. Epub 2017/09/08. doi: 10.3347/kjp.2017.55.4.429. PubMed PMID: 28877576; PubMed Central PMCID: PMCPMC5594723.

34. Hela BJ, Abir B, Majdi G, Aiman D, Iheb S, Nizar E, et al. Interventricular septum hydatid cyst presenting with acute lower limb ischemia: a case report. Libyan J Med. 2015;10:28634. Epub 2015/07/02. doi: 10.3402/ljm.v10.28634. PubMed PMID: 26130458; PubMed Central PMCID: PMCPMC4486353.

35. Huerta-Obando AV, Olivera-Baca EY, Silva-Diaz J, Salazar-Diaz A. [Cardiac Hydatid Cyst in a Child: A Case Report]. Rev Peru Med Exp Salud Publica. 2018;35(2):338-43. Epub 2018/09/06. doi: 10.17843/rpmesp.2018.352.3258. PubMed PMID: 30183910.

36. Inzirillo F, Giorgetta C, Ravalli E. Pericardial echinococcosis: unusual presentation in a non-endemic region. Asian Cardiovasc Thorac Ann. 2014;22(9):1126-7. Epub 2014/06/03. doi: 10.1177/0218492313507936. PubMed PMID: 24887900.

37. Jain N, Sethi S, Gupta N, Goel V, Puri SK. Comprehensive Evaluation of Cardiac Hydatid Using 256 Slice Dual Source CT: One Stop Shop. J Clin Diagn Res. 2015;9(10):Td01-3. Epub 2015/11/12. doi: 10.7860/jcdr/2015/13679.6550. PubMed PMID: 26557591; PubMed Central PMCID: PMCPMC4625310.

38. Jain PK, Malik V, Divya A, Narula J, Hote M. Interventricular septal hydatid cyst: Transesophageal echocardiography as a therapeutic tool during bypass. Ann Card Anaesth. 2015;18(3):421-4. Epub 2015/07/04. doi: 10.4103/0971-9784.159817. PubMed PMID: 26139754; PubMed Central PMCID: PMCPMC4881693.

39. Kara SS, Gullu UU. A case of intracardiac echinococcosis. Cardiol Young. 2017;27(7):1392-3. Epub 2017/06/21. doi: 10.1017/s1047951117001196. PubMed PMID: 28631585.

40. Kastengren M, Liska J, Svenarud P, Dalen M. Intraventricular Septal Echinococcosis. Ann Thorac Surg. 2017;104(1):e89. Epub 2017/06/22. doi: 10.1016/j.athoracsur.2017.02.049. PubMed PMID: 28633274.

41. Khan JK, Khan S, Fatimi S. An unexpected case of a hydatid cyst on the mitral valve mimicking infective endocarditis. Eur J Cardiothorac Surg. 2016;49(4):1290. Epub 2015/05/20. doi: 10.1093/ejcts/ezv187. PubMed PMID: 25983081.

42. Khosravi A, Taghipour H, Fanaei SA, Assar O, Ghyasy MS, Mirlohi SM. Heart hydatid cyst close to the left descending artery in a thirteen-year-old boy. Iran Red Crescent Med J. 2014;16(9):e15164. Epub 2015/01/17. doi: 10.5812/ircmj.15164. PubMed PMID: 25593721; PubMed Central PMCID: PMCPMC4270658.

43. Kitai T, Ito S, Koyama T, Furukawa Y. Echinococcosis of the heart. Eur Heart J. 2014;35(25):1682. Epub 2014/04/10. doi: 10.1093/eurheartj/ehu136. PubMed PMID: 24713646.

44. Klein K, Kehmeier E. [52-year-old female with dyspnea and decreased physical capacity]. Dtsch Med Wochenschr. 2014;139(40):2023-4. Epub 2014/09/26. doi: 10.1055/s-0034-1387229. PubMed PMID: 25254400.

45. Kocabay G, Yilmazer S, Korkmaz Y, Karadeniz A, Duran NE. Primary cardiac hydatid cyst presenting as noncompaction cardiomyopathy. Echocardiography. 2017;34(1):141-2. Epub 2016/10/01. doi: 10.1111/echo.13391. PubMed PMID: 27688059.

46. Kohlmaier B, Trobisch A, Pfurtscheller K, Knez I, Klepetko W, Pilhatsch A, et al. Cardiac and Pulmonary Cystic Echinococcosis With Massive Obstruction of the Pulmonary Vessel System in a 16-Year-Old Girl. Pediatr Infect Dis J. 2018;37(11):e273-e5. Epub 2018/04/11. doi: 10.1097/inf.0000000000002066. PubMed PMID: 29634619.

47. Kothari J, Lakhia K, Solanki P, Bansal S, Boraniya H, Pandya H, et al. Invasive pericardial hydatid cyst: Excision of multiple huge cysts. J Saudi Heart Assoc. 2017;29(1):53-6. Epub 2017/01/28. doi: 10.1016/j.jsha.2016.06.005. PubMed PMID: 28127219; PubMed Central PMCID: PMCPMC5247296.

48. L'Aarje A, Lyazidi S, Kitane Y, Alami A, Habbal R. Cardiac hydatid cyst of the right ventricle: Severe localization. J Cardiol Cases. 2017;16(4):138-40. Epub 2017/08/02. doi: 10.1016/j.jccase.2017.06.009. PubMed PMID: 30279818; PubMed Central PMCID: PMCPMC6149277.

49. Levent F, Edem E, Emren SV, Altay S. Coronary compression by supposed cardiac hydatid cyst: an unusual cardiac mass. Postepy Kardiol Interwencyjnej. 2018;14(2):208-9. Epub 2018/07/17. doi: 10.5114/aic.2018.76418. PubMed PMID: 30008779; PubMed Central PMCID: PMCPMC6041829.

50. Mantini C, Capparuccia C, Cademartiri F, Messalli G, Mastrodicasa D, Cinalli S, et al. Uncommon Isolated Unilocular Myocardial Cyst in a Dog-Friendly Young Female Patient- Multimodality Imaging. Circ J. 2017;81(7):1056-8. Epub 2017/01/31. doi: 10.1253/circj.CJ-16-1215. PubMed PMID: 28132983.

51. Martin-Izquierdo M, Martin-Trenor A. [Hydatidosis simulating a cardiac tumour with pulmonary metastases]. Cir Cir. 2016;84(4):318-23. Epub 2016/01/08. doi: 10.1016/j.circir.2015.03.003. PubMed PMID: 26738651.

52. Mirijello A, Pepe G, Zampiello P, Criconia GM, Mendola A, Manfrini A. A Male Patient with Syncope, Anaphylaxis, and ST-Elevation: Hepatic and Cardiac Echinococcosis Presenting with Kounis Syndrome. J Emerg Med. 2016;51(4):e73-e7. Epub 2016/08/16. doi: 10.1016/j.jemermed.2016.06.011. PubMed PMID: 27516403.

53. Mirzaie A, Erfanian-Taghvaei MR, Mirzaie M, Sharifi-Noghabi R. Interventricular Septum Hydatid Cyst: Successful Seven-Year Follow Up- Case Report. Iran J Public Health. 2014;43(9):1295-8. Epub 2015/07/16. PubMed PMID: 26175985; PubMed Central PMCID: PMCPMC4500433.

54. Modani S, Karthik DK, Heda S, Deshpande A. Atypical chest pain in a patient with hydatid cyst of the interventricular septum. BMJ Case Rep. 2018;2018. Epub 2018/06/18. doi: 10.1136/bcr-2018-224833. PubMed PMID: 29909390.

55. Moghul D, Hamidi H. Incidental finding of cardiac hydatid cysts, report of two cases. BMC Med Imaging. 2018;18(1):22. Epub 2018/08/15. doi: 10.1186/s12880-018-0268-2. PubMed PMID: 30103678; PubMed Central PMCID: PMCPMC6090605.

56. Mouhsine A, Belkouch A, Athmane el M, Roukhssi R, El Fikri A, Belyamani L, et al. Hydatid cyst of the pericardium: a case report. Pan Afr Med J. 2014;19:330. Epub 2014/01/01. doi: 10.11604/pamj.2014.19.330.5542. PubMed PMID: 25918570; PubMed Central PMCID: PMCPMC4405061.

57. Musleh M, Abuhussein N, Musleh G, Waterworth P. Innovative use of the octopus stabilizer in the excision of a cardiac hydatid cyst. J Surg Case Rep. 2016;2016(2). Epub 2016/02/28. doi: 10.1093/jscr/rjw019. PubMed PMID: 26921611; PubMed Central PMCID: PMCPMC4769357.

58. Naeem SN, Burhan H, Khan G. Hydatid cyst of the cardiac interventricular septum. Asian Cardiovasc Thorac Ann. 2015;23(3):320-2. Epub 2014/06/03. doi: 10.1177/0218492313506125. PubMed PMID: 24887895.

59. Ohri S, Sachdeva A, Bhatia M, Shrivastava S. Cardiac hydatid cyst in left ventricular free wall. Echo Res Pract. 2015;2(1):K17-9. Epub 2015/12/23. doi: 10.1530/erp-14-0112. PubMed PMID: 26693324; PubMed Central PMCID: PMCPMC4676447.

60. Oraha AY, Faqe DA, Kadoura M, Kakamad FH, Yaldo FF, Aziz SQ. Cardiac Hydatid cysts; presentation and management. A case series. Ann Med Surg (Lond). 2018;30:18-21. Epub 2018/06/28. doi: 10.1016/j.amsu.2018.04.001. PubMed PMID: 29946454; PubMed Central PMCID: PMCPMC6016321.

61. Ozbudak E, Durmaz D, Arikan AA, Halici U, Yavuz S, Emre E. Incidentally detected cardiac cyst hydatid after blunt thoracic trauma. Korean J Thorac Cardiovasc Surg. 2014;47(2):160-2. Epub 2014/05/02. doi: 10.5090/kjtcs.2014.47.2.160. PubMed PMID: 24782970; PubMed Central PMCID: PMCPMC4000877.

62. Ozturk C, Yildirim AO, Unlu M, Aparci M, Demirkol S, Sari S, et al. The association of intracardiac hydatid cyst and muscle bridge cause electrocardiographic abnormality detected by multimodality imaging. Anadolu Kardiyol Derg. 2014;14(7):E17-8. Epub 2014/08/28. doi: 10.5152/akd.2014.5664. PubMed PMID: 25163091.

63. Ozturk M, Sigirci A, Dagli AF. A rare cause of embolism in the popliteal artery of an adolescent: ruptured cardiac hydatid cyst. Anatol J Cardiol. 2015;15(6):E20. Epub 2015/05/27. doi: 10.5152/akd.2015.6341. PubMed PMID: 26006151; PubMed Central PMCID: PMCPMC5779162.

64. Padmanabhan TNC, Kumar KVK, Sadiq Azam M, Kumar Bilolikar A. Primary echinococcus infection of the heart: a rare type of cystic echinococcosis. Eur Heart J. 2017;38(29):2255. Epub 2017/05/13. doi: 10.1093/eurheartj/ehx269. PubMed PMID: 28499031.

65. Parakh N, Jagia P, Hote M, Arava S. Giant Hydatid Cyst of the Interventricular Septum. Echocardiography. 2016;33(3):488-90. Epub 2015/10/27. doi: 10.1111/echo.13090. PubMed PMID: 26498475.

66. Peker RO, Sabuncu T, Kumbasar U, Guvener M, Demircin M, Pasaoglu I. A giant hydatid cyst atypically located in the left ventricle. Turk J Pediatr. 2015;57(3):308-10. Epub 2015/12/25. PubMed PMID: 26701955.

67. Petik B, Hazirolan T, Uysal G, Erturk SM. Cardiac Hydatid Cysts: Computed Tomography and Magnetic Resonance Imaging Findings of the 5 Cases. J Comput Assist Tomogr. 2015;39(5):816-9. Epub 2015/07/22. doi: 10.1097/rct.0000000000000284. PubMed PMID: 26196344.

68. Poorzand H, Teshnizi MA, Baghini VS, Gifani M, Gholoobi A, Zirak N. Giant cardiac hydatid cyst with rare adhesions. Hellenic J Cardiol. 2014;55(3):260-4. Epub 2014/05/28. PubMed PMID: 24862620.

69. Prati G, Gatti G, Belgrano M, Pinamonti B, Rauber E, Gripshi F, et al. Disseminated echinococcosis: follow your heart. J Cardiovasc Med (Hagerstown). 2016;17 Suppl 2:e146-e8. Epub 2017/05/10. doi: 10.2459/jcm.0000000000000389. PubMed PMID: 28482352.

70. Rossetti E, Boto A, Gonzalez Cambaceres C, Ruvinsky S, Sagray E. Acute arterial embolism as the clinical presentation of a disseminated hydatidosis: case report. Arch Argent Pediatr. 2018;116(4):e616-e20. Epub 2018/07/18. doi: 10.5546/aap.2018.eng.e616. PubMed PMID: 30016043.

71. Sabzi F, Faraji R. Hydatid cyst of the interventricular septum causing complete heart block and postoperative ventricular septal defect. Indian J Crit Care Med. 2014;18(7):473-5. Epub 2014/08/07. doi: 10.4103/0972-5229.136080. PubMed PMID: 25097364; PubMed Central PMCID: PMCPMC4118517.

72. Sabzi F, Madani H, Dabiri S, Pormotabed A, Faraji R. Thrombotic cardiac apex hydatid cyst. Indian Heart J. 2015;67(6):577-80. Epub 2015/12/26. doi: 10.1016/j.ihj.2015.09.022. PubMed PMID: 26702690; PubMed Central PMCID: PMCPMC4699956.

73. Sabzi F, Madani H, Khosravi D, Hosseini MS, Faraji R. Large cardiac hydatidosis bulging as a mass to pericardium-case report. Iran J Parasitol. 2015;10(1):128-31. Epub 2015/04/24. PubMed PMID: 25904956; PubMed Central PMCID: PMCPMC4403532.

74. Sabzi F, Vaziri S, Faraji R. Right ventricular hydatid cyst ruptured to pericardium. Ann Card Anaesth. 2015;18(3):445-8. Epub 2015/07/04. doi: 10.4103/0971-9784.159825. PubMed PMID: 26139761; PubMed Central PMCID: PMCPMC4881708.

75. Saglican Y, Yalcin O, Kaygusuz E. Cystic Echinococcosis: One Entity, Two Unusual Locations. Turkiye Parazitol Derg. 2016;40(1):51-3. Epub 2016/05/26. doi: 10.5152/tpd.2016.4378. PubMed PMID: 27222337.

76. Sahin I, Ozkaynak B, Ayca B, Okuyan E. An uncommon localization of a giant hydatid cyst presenting with cardiac tamponade. Turk Kardiyol Dern Ars. 2015;43(1):86-8. Epub 2015/02/07. doi: 10.5543/tkda.2015.59207. PubMed PMID: 25655857.

77. Sahin I, Ozkaynak B, Gungor B, Ayca B, Sigirci S, Mert B, et al. Sustained ventricular tachycardia as the first manifestation of hydatid cyst located in the interventricular septum. Echocardiography. 2015;32(7):1196-8. Epub 2015/01/15. doi: 10.1111/echo.12894. PubMed PMID: 25586822.

78. Sahin ST, Cengiz B, Yurdakul S, Aytekin S. A huge multilocular hydatid cyst with an unusual localization. Acta Cardiol. 2015;70(6):746. Epub 2015/12/31. doi: 10.2143/ac.70.6.3120196. PubMed PMID: 26717232.

79. Salehi R, Parvizi R, Pourafkari L, Nader ND. Large isolated hydatid cyst of the interventricular septum. Echo Res Pract. 2015;2(1):I5-i6. Epub 2015/12/23. doi: 10.1530/erp-14-0075. PubMed PMID: 26693319; PubMed Central PMCID: PMCPMC4676427.

80. Salido L, Mestre JL, Moya JL, Hernandez-Antolin R, Zamorano JL. Chronic giant hydatid cyst fistulized to the left ventricle: long-term survival without surgery. Eur Heart J Cardiovasc Imaging. 2015;16(7):798. Epub 2015/03/10. doi: 10.1093/ehjci/jev042. PubMed PMID: 25750200.

81. Sarli B, Ugurlu M, Baktir AO, Tekin AI, Tok A, Yagmur B. Lone, Mobile Left Atrial Hydatid Cyst. Tex Heart Inst J. 2016;43(3):261-3. Epub 2016/06/16. doi: 10.14503/thij-15-5261. PubMed PMID: 27303247; PubMed Central PMCID: PMCPMC4894710.

82. Savas G, Sahin O, Ozmen S, Oguzhan A. Case images: coexistence of the heart and liver hydatid cyst. Turk Kardiyol Dern Ars. 2014;42(6):588. Epub 2014/11/05. doi: 10.5543/tkda.2014.60955. PubMed PMID: 25362956.

83. Senhaji L, Karhate M, Amara B, El Biaze M, Benjelloun MC, Tizniti S, et al. [Multiple lung nodules and mediastinal widening of unusual etiology]. Rev Mal Respir. 2015;32(3):291-5. Epub 2015/04/08. doi: 10.1016/j.rmr.2014.03.013. PubMed PMID: 25847208.

84. Seth HS, Mishra P, Khandekar JV, Raut C, Mohapatra CKR, Ammannaya GKK. A Concomitant Intramyocardial and Pulmonary Hydatid Cyst: A Rare Case Report. Braz J Cardiovasc Surg. 2017;32(2):138-40. Epub 2017/05/12. doi: 10.21470/1678-9741-2016-0046. PubMed PMID: 28492796; PubMed Central PMCID: PMCPMC5409250.

85. Shakil U, Rehman AU, Shahid R. Isolated cardiac hydatid cyst. J Coll Physicians Surg Pak. 2015;25(5):374-5. Epub 2015/05/27. doi: 05.2015/jcpsp.374375. PubMed PMID: 26008667.

86. Sharma P, Lakhia K, Malhotra A, Garg P. Ruptured intracardiac hydatid cyst presenting as acute coronary syndrome. Asian Cardiovasc Thorac Ann. 2016;24(6):587-9. Epub 2015/06/06. doi: 10.1177/0218492315589196. PubMed PMID: 26045489.

87. Shojaei E, Yassin Z, Rezahosseini O. Cardiac Hydatid Cyst: A Case Report. Iran J Public Health. 2016;45(11):1507-10. Epub 2016/12/29. PubMed PMID: 28028503; PubMed Central PMCID: PMCPMC5182260.

88. Shojaeifard M, Hosseini S, Hosseini S, Pouraliakbar H, Dehghani H, Noohi F. Cardiac Hydatid Cyst without Liver Involvement: A Case Report. Iran J Parasitol. 2016;11(2):274-8. Epub 2017/01/18. PubMed PMID: 28096865; PubMed Central PMCID: PMCPMC5236108.

89. Singh A, Sharma R, Garg A, Nanda NC, Elsayed M, Taher A, et al. Usefulness of bubble study in echocardiographic diagnosis of contained rupture of hydatid cyst in the right ventricular outflow tract. Echocardiography. 2016;33(9):1402-8. Epub 2016/09/22. doi: 10.1111/echo.13317. PubMed PMID: 27650224.

90. Suner A, Koroglu S, Cakici M, Polat M, Cetin M, Petik B, et al. Mediastinal hydatid cyst compressing the pulmonary artery and atrial septal defect: a rare association. Rev Port Cardiol. 2014;33(5):317-8. Epub 2014/06/04. doi: 10.1016/j.repc.2013.11.011. PubMed PMID: 24890769.

91. Svrckova P, Nabarro L, Chiodini PL, Jager HR. Disseminated cerebral hydatid disease (multiple intracranial echinococcosis). Pract Neurol. 2019;19(2):156-63. Epub 2018/10/12. doi: 10.1136/practneurol-2018-001954. PubMed PMID: 30305379.

92. Tabesh H, Ahmadi Tafti H, Ameri S. Unusual Presentation of Interventricular Hydatid Cyst: A Case Report. Iran J Public Health. 2015;44(1):130-3. Epub 2015/06/11. PubMed PMID: 26060784; PubMed Central PMCID: PMCPMC4450000.

93. Tefera E, Knapp J, Teodori M. Hydatid cyst of the interventricular septum. Glob Cardiol Sci Pract. 2017;2017(1):e201709. Epub 2017/10/04. doi: 10.21542/gcsp.2017.9. PubMed PMID: 28971108; PubMed Central PMCID: PMCPMC5621715.

94. Tekin AF, Durmaz MS, Dagli M, Akbayrak S, Akbayrak P, Turgut B. Left ventricular hydatid cyst mimicking acute coronary syndrome. Radiol Case Rep. 2018;13(3):697-701. Epub 2018/04/24. doi: 10.1016/j.radcr.2018.03.019. PubMed PMID: 29682142; PubMed Central PMCID: PMCPMC5909026.

95. Uygur B, Ustabasioglu FE, Karakurt H, Akinci O, Celik O. An unusual cause of chest pain: An isolated huge cardiac hydatid cyst. J Clin Ultrasound. 2018;46(4):262-4. Epub 2017/06/29. doi: 10.1002/jcu.22518. PubMed PMID: 28656619.

96. Vazhev ZG, Stoev HA. Cardiac Echinococcosis Involving Left Ventricular Myocardium in an 18-Year-Old Patient. Folia Med (Plovdiv). 2018;60(2):308-13. Epub 2018/10/26. doi: 10.1515/folmed-2017-0081. PubMed PMID: 30355821.

97. Wadhawa V, Shah J, Doshi C, Ramani J, Lakhia K, Rathod D, et al. Surgical overview of cardiac echinococcosis: a rare entity. Interact Cardiovasc Thorac Surg. 2018;27(2):191-7. Epub 2018/03/10. doi: 10.1093/icvts/ivy053. PubMed PMID: 29522097.

98. Warraich HJ, Rymer JA, Schroder JN, Kim HW, Leithe ME, Harrison JK. Intracardiac Echinococcal Cyst Causing Biventricular Cavity Obliteration. Circ Heart Fail. 2018;11(2):e004830. Epub 2018/02/18. doi: 10.1161/circheartfailure.117.004830. PubMed PMID: 29453289.

99. Xiong F, Liu C, Wang S, Feng K. Huge right ventricular outflow echinococcosis presenting as acute coronary syndrome. J Clin Ultrasound. 2016;44(8):518-21. Epub 2016/03/01. doi: 10.1002/jcu.22348. PubMed PMID: 26923677.

100. Yaman M, Ates AH, Arslan U, Ozturk H, Aksakal A. A giant cardiac hydatid cyst presenting with chest pain and ventricular tachycardia in a pregnant woman undergoing cesarean section. Indian Heart J. 2016;68 Suppl 2:S118-s20. Epub 2016/10/19. doi: 10.1016/j.ihj.2016.04.011. PubMed PMID: 27751261; PubMed Central PMCID: PMCPMC5067732.

101. Yasim A, Ustunsoy H, Gokaslan G, Hafiz E, Arslanoglu Y. Cardiac Echinococcosis: A Single-Centre Study with 25 Patients. Heart Lung Circ. 2017;26(2):157-63. Epub 2016/08/17. doi: 10.1016/j.hlc.2016.05.122. PubMed PMID: 27526976.

102. Yildiz CE, Sinan UY, Yildiz A, Cetin G, Kucukoglu S. A Case of Isolated Cardiac Hydatid Cyst that Mimics Lymphoproliferative Malignancy. Echocardiography. 2015;32(6):1036-9. Epub 2014/12/04. doi: 10.1111/echo.12856. PubMed PMID: 25470654.

103. Yilmaz R, Akpinar YE, Bayramoglu Z, Ozyavuz HI, Unal OF, Dursun M. Magnetic resonance imaging characteristics of cardiac hydatid cyst. Clin Imaging. 2018;51:202-8. Epub 2018/06/04. doi: 10.1016/j.clinimag.2018.05.016. PubMed PMID: 29860193.

104. Zaghba N, Maiouak S, Benjelloun H, Yassine N, Bakhatar A, Bahlaoui A. [Chronic pulmonary heart disease complicating hydatic embolism]. Med Mal Infect. 2015;45(1-2):52-4. Epub 2015/01/03. doi: 10.1016/j.medmal.2014.11.006. PubMed PMID: 25553612.

105. Akpinar I, Tekeli S, Sen T, Sen N, Basar N, Cagli KE, et al. Extremely rare cardiac involvement: recurrent pericardial hydatid cyst. Intern Med. 2012;51(4):391-3. Epub 2012/02/16. doi: 10.2169/internalmedicine.51.6370. PubMed PMID: 22333375.

106. Aksakal E, Degirmenci H, Bakirci EM, Sevimli S. Symptomatic isolated giant hydatid cyst in the posterolateral region of the left ventricle. Turk Kardiyol Dern Ars. 2011;39(8):740. Epub 2012/01/20. doi: 10.5543/tkda.2011.01683. PubMed PMID: 22257819.

107. Akshay S, Abid GM, Ajeya J, Kumar SD. Left ventricular hydatid cyst. J Card Surg. 2012;27(6):710-1. Epub 2012/07/07. doi: 10.1111/j.1540-8191.2012.01497.x. PubMed PMID: 22765347.

108. Alizadeh-Ghavidel A, Kyavar M, Sadeghpour A, Totonchi Z, Mirmesdagh Y, Almassi N, et al. Unusual clinical presentation of a giant left ventricle hydatid cyst. J Cardiovasc Thorac Res. 2013;5(4):175-8. Epub 2014/01/10. doi: 10.5681/jcvtr.2013.038. PubMed PMID: 24404351; PubMed Central PMCID: PMCPMC3883543.

109. Allouch M, Ben Ahmed H, Gloulou F, Moncef H. Sudden death due to an unrecognized cardiac hydatid cyst. Intern Med. 2011;50(18):2051-2. Epub 2011/09/17. doi: 10.2169/internalmedicine.50.5813. PubMed PMID: 21921397.

110. Atalay A, Salih OK, Gezer S, Gocen U, Yaliniz H, Keklik V, et al. Simultaneous heart and bilateral lung hydatid cyst operated in a single session. Heart Lung Circ. 2013;22(8):682-4. Epub 2012/12/26. doi: 10.1016/j.hlc.2012.11.014. PubMed PMID: 23265691.

111. Bakirci EM, Kalkan K, Duman H, Tanboga IH, Degirmenci H. Pancardiac hydatid cyst. Anadolu Kardiyol Derg. 2013;13(7):E39. Epub 2013/11/01. doi: 10.5152/akd.2013.254. PubMed PMID: 24172812.

112. Bayraktaroglu S, Ceylan N, Savas R, Nalbantgil S, Alper H. Hydatid disease of right ventricle and pulmonary arteries: a rare cause of pulmonary embolism--computed tomography and magnetic resonance imaging findings (2009: 5b). Eur Radiol. 2009;19(8):2083-6. Epub 2009/07/07. doi: 10.1007/s00330-008-1165-9. PubMed PMID: 19578922.

113. Behzadnia N, Hossein-Ahmadi Z, Sharif-Kashani B, Sheybani-Afshar F, Naghash-Zadeh F, Ansari-Aval Z, et al. Pericardial hydatid cyst in oblique sinus, obstructing all pulmonary veins: a rare presentation. Tanaffos. 2013;12(1):78-80. Epub 2013/01/01. PubMed PMID: 25191454; PubMed Central PMCID: PMCPMC4153237.

114. Ben Khelil M, Allouche M, Banasr A, Gloulou F, Benzarti A, Zhioua M, et al. Sudden death due to hydatid disease: a six-year study in the northern part of Tunisia. J Forensic Sci. 2013;58(5):1163-70. Epub 2013/07/05. doi: 10.1111/1556-4029.12172. PubMed PMID: 23822140.

115. Beselia K, Nachkepia M, Gigilashvili K, Keshelava G, Janashia G, Imnadze G. Surgical treatment of an Echinococcus cyst of the interventricular septum complicated by septic endocarditis, complete atrioventricular block, and rupture into the Valsalva sinus. Gen Thorac Cardiovasc Surg. 2010;58(5):248-50. Epub 2010/05/08. doi: 10.1007/s11748-009-0516-z. PubMed PMID: 20449717.

116. Besir Y, Gucu A, Surer S, Rodoplu O, Melek M, Tetik O. Giant cardiac hydatid cyst in the interventricular septum protruding to right ventricular epicardium. Indian Heart J. 2013;65(1):81-3. Epub 2013/02/27. doi: 10.1016/j.ihj.2012.12.014. PubMed PMID: 23438618; PubMed Central PMCID: PMCPMC3861300.

117. Bonardi M, Dellabianca C, Della Valle V, Valentini A, Raineri C, Dore R. Hydatid cyst of the cardiac interventricular septum. Int J Cardiol. 2012;158(3):e45-6. Epub 2011/11/15. doi: 10.1016/j.ijcard.2011.10.022. PubMed PMID: 22075409.

118. Bozkurt H, Aydin M, Talas Z, Demir Y, Surucu E. Rest myocardial perfusion scintigraphy showing the invasion of the hydatid cyst in the myocardium. Clin Nucl Med. 2013;38(12):1006-8. Epub 2013/11/12. doi: 10.1097/rlu.0000000000000229. PubMed PMID: 24212446.

119. Braggion-Santos MF, Abdel-Aty H, Hofmann N, Katus HA, Steen H. Cardiac hydatidosis: a follow-up with cardiovascular magnetic resonance. Circulation. 2012;126(18):2257-60. Epub 2012/10/31. doi: 10.1161/circulationaha.112.117176. PubMed PMID: 23109516.

120. Braggion-Santos MF, Abdel-Aty H, Hofmann N, Katus HA, Steen H. Cardiac cystic echinococcosis: a long-term follow-up case report. Clin Res Cardiol. 2013;102(1):85-8. Epub 2012/08/08. doi: 10.1007/s00392-012-0497-2. PubMed PMID: 22868693.

121. Caglayan K, Celik A, Koc A, Kutluk AC, Altinli E, Celik AS, et al. Unusual locations of hydatid disease: diagnostic and surgical management of a case series. Surg Infect (Larchmt). 2010;11(4):349-53. Epub 2010/08/11. doi: 10.1089/sur.2009.017. PubMed PMID: 20695827.

122. Cakici M, Cetin M, Ercan S, Davutoglu V. Isolated multiple invasive cardiac hydatid cyst. BMJ Case Rep. 2013;2013. Epub 2013/06/12. doi: 10.1136/bcr-2013-010106. PubMed PMID: 23749835; PubMed Central PMCID: PMCPMC3702852.

123. Canpolat U, Yorgun H, Sahiner L, Aytemir K. Hydatid cyst of the interventricular septum presenting as supraventricular tachycardia. Anadolu Kardiyol Derg. 2012;12(6):E30-1. Epub 2012/06/26. doi: 10.5152/akd.2012.178. PubMed PMID: 22728744.

124. Canpolat U, Yorgun H, Sunman H, Aytemir K. Cardiac hydatid cyst mimicking left ventricular aneurysm and diagnosed by magnetic resonance imaging. Turk Kardiyol Dern Ars. 2011;39(1):47-51. Epub 2011/03/02. PubMed PMID: 21358231.

125. Cansu A, Cakir M, Dilber E, Gedikli O, Cansu A, Kul S, et al. Cardiac echinococcosis associated with cerebrovascular occlusive disease and subcutaneous bullous eruptions and ulcers. Ann Trop Paediatr. 2011;31(3):251-4. Epub 2011/07/26. doi: 10.1179/1465328111y.0000000018. PubMed PMID: 21781421.

126. Carrascal Y, Arroyo J, Valenzuela H, Fernandez M. Cardiac echinococcosis: surviving 40 years later? Eur J Cardiothorac Surg. 2012;41(1):231. Epub 2011/06/07. doi: 10.1016/j.ejcts.2011.04.030. PubMed PMID: 21640604; PubMed Central PMCID: PMCPMC3241118.

127. Catano J. Cardiac echinococcosis, an unusual echocardiographic finding. Am J Trop Med Hyg. 2010;82(2):173. Epub 2010/02/06. doi: 10.4269/ajtmh.2010.09-0557. PubMed PMID: 20133987; PubMed Central PMCID: PMCPMC2813152.

128. Cecconi A, Maroto L, Vilacosta I, Luaces M, Ortega L, Escribano N, et al. Acute pericarditis secondary to hydatid cyst rupture: diagnosis by multimodality imaging. Circulation. 2013;128(18):2073-4. Epub 2013/10/30. doi: 10.1161/circulationaha.112.000392. PubMed PMID: 24166417.

129. Cece H, Yildiz S, Sogut O, Hazar A, Sezen Y. Isolated Pericardial Hydatid Cyst: A Case Report. Cardiol Res. 2011;2(5):253-5. Epub 2011/10/01. doi: 10.4021/cr96w. PubMed PMID: 28357016; PubMed Central PMCID: PMCPMC5358288.

130. Chaurasia AS, Nawale JM, Patil SN, Yemul MA, Mukhedkar S, Sharma SK, et al. Cystic hydatidosis of the heart and brain. Echocardiography. 2012;29(8):E208-9. Epub 2012/06/15. doi: 10.1111/j.1540-8175.2012.01743.x. PubMed PMID: 22694125.

131. Darwazah AK, Zaghari M, Eida M, Batrawy M. Left ventricular endocardial ecchinococcosis associated with multiple intracranial hydatid cysts. J Cardiothorac Surg. 2013;8:104. Epub 2013/04/23. doi: 10.1186/1749-8090-8-104. PubMed PMID: 23601473; PubMed Central PMCID: PMCPMC3639073.

132. Diaz-Menendez M, Perez-Molina JA, Norman FF, Perez-Ayala A, Monge-Maillo B, Fuertes PZ, et al. Management and outcome of cardiac and endovascular cystic echinococcosis. PLoS Negl Trop Dis. 2012;6(1):e1437. Epub 2012/01/12. doi: 10.1371/journal.pntd.0001437. PubMed PMID: 22235354; PubMed Central PMCID: PMCPMC3250507.

133. Dogra N, Puri GD, Kumar B. Isolated pericardial echinococcosis: Perioperative transesophageal echocardiographic evaluation. J Cardiovasc Dis Res. 2013;4(2):149-51. Epub 2013/09/13. doi: 10.1016/j.jcdr.2012.11.004. PubMed PMID: 24027375; PubMed Central PMCID: PMCPMC3770108.

134. Donegani E, Pisani P, Radaelli S, Pula G, Portella G. Right ventricular hydatid cyst: a case report. J Cardiovasc Surg (Torino). 2009;50(3):417-8. Epub 2009/04/03. PubMed PMID: 19339960.

135. Eckle T, Peter J, Ruttimann S. [A very uncommon reason for chest pain]. Ther Umsch. 2012;69(12):679-82. Epub 2012/11/29. doi: 10.1024/0040-5930/a000348. PubMed PMID: 23188778.

136. El Majhad A, Lachhab A, Cherradi R, Srairi J, Srairi N, Doghmi N, et al. [The contribution of magnetic resonance imaging (MRI) in the diagnosis of cardiac hydatid cyst]. East Mediterr Health J. 2011;17(12):996-1000. Epub 2012/02/24. PubMed PMID: 22355956.

137. Ellouze S, Bahri M, Abdennadher M, Kossentini M, Abid N, Chaabouni S, et al. [Rare cause of pulmonary embolism in a child]. Arch Pediatr. 2011;18(9):987-9. Epub 2011/08/02. doi: 10.1016/j.arcped.2011.06.008. PubMed PMID: 21803553.

138. Ercan S, Bosnak V, Yuce M, Davutoglu V, Yavuz F. Isolated invasive endomyocardial cystic echinococcosis presenting with heart failure. Case Rep Med. 2012;2012:603087. Epub 2012/07/26. doi: 10.1155/2012/603087. PubMed PMID: 22829839; PubMed Central PMCID: PMCPMC3398593.

139. Ercan S, Gokaslan G, Davutoglu V, Inanc IH, Bosnak V, Deniz H. Isolated ruptured inter-atrial septal cystic echinococcosis during pregnancy. Echocardiography. 2013;30(8):E236-8. Epub 2013/05/29. doi: 10.1111/echo.12260. PubMed PMID: 23710802.

140. Erol T, Altay H, Tarim E. A pericardial hydatid cyst and pregnancy. Acta Cardiol. 2011;66(3):387-9. Epub 2011/07/13. doi: 10.2143/ac.66.3.2114143. PubMed PMID: 21744713.

141. Ertas F, Duygu H, Acet H, Nazli C, Ergene O. Isolated pericardial hydatidosis as a cause of chest pain. Int J Cardiol. 2011;150(1):e28-9. Epub 2009/09/22. doi: 10.1016/j.ijcard.2009.08.033. PubMed PMID: 19765837.

142. Espinoza C, Palma P, Soto D, Rozas V, Ramirez A, Gonzalez M. [Cardiac hydatidosis: Report of one case]. Rev Med Chil. 2012;140(11):1449-52. Epub 2013/05/17. doi: 10.4067/s0034-98872012001100011. PubMed PMID: 23677192.

143. Fabijanic D, Bulat C, Letica D, Nenadic D, Pesutic-Pisac V, Carevic V. Echocardiographic appearance of a hydatid cyst of the papillary muscle and chordae tendineae. J Clin Ultrasound. 2011;39(7):431-3. Epub 2011/04/15. doi: 10.1002/jcu.20818. PubMed PMID: 21491459.

144. Fazlinezhad A, Moohebati M, Azari A, Bigdeloo L. Acute pericardial tamponade due to ruptured multiloculated myocardial hydatid cyst. Eur J Echocardiogr. 2009;10(3):459-61. Epub 2008/09/27. doi: 10.1093/ejechocard/jen249. PubMed PMID: 18818301.

145. Fendri AH, Boulacel A, Brahami A. [Hydatic cyst of the heart at the Constantine Erriadh teaching hospital (Algeria). A retrospective study from 2008 to 2009]. Bull Soc Pathol Exot. 2010;103(5):305-8. Epub 2010/11/06. doi: 10.1007/s13149-010-0083-7. PubMed PMID: 21052887.

146. Fernandez-Berges D, Fernandez Sarabia MT, Zabala Morales MS, Felix Redondo FJ. [Cardiac hydatidic cyst]. Med Clin (Barc). 2009;132(19):762-3. Epub 2009/03/31. doi: 10.1016/j.medcli.2009.01.007. PubMed PMID: 19327788.

147. Gecmen C, Candan O, Karabay CY, Guler A, Elveran A, Baskan O, et al. Hydatid cyst in the right atrium and pulmonary hypertension secondary to cystic embolism. Hellenic J Cardiol. 2011;52(2):160-2. Epub 2011/04/12. PubMed PMID: 21478127.

148. Gerber BL, Pasquet A, El Khoury G, Verhelst R, Vanoverschelde JL, Watremez C, et al. Echinococcosis of the heart and ascending aorta. Circulation. 2012;125(1):185-7. Epub 2012/01/05. doi: 10.1161/circulationaha.111.043893. PubMed PMID: 22215893.

149. Goz M, Hazar A, Demirbag R. Multivesicular intrapericardial hydatidosis. Eur J Cardiothorac Surg. 2011;39(4):605. Epub 2010/08/31. doi: 10.1016/j.ejcts.2010.07.018. PubMed PMID: 20801668.

150. Gruttadauria S, D'Ancona G, Pagano D, Panarello G, Follis F, Gridelli B. Complex hydatid cyst of the liver with intra-cardiac involvement. Am Surg. 2010;76(12):E241-2. Epub 2011/03/01. PubMed PMID: 21352681.

151. Haggui A, Saidi I, Dahmani R, Hajlaoui N, Lahidheb D, Filali T, et al. [Intra myocardial hydatid cyst fortuitously discovered]. Tunis Med. 2012;90(11):829-30. Epub 2012/12/01. PubMed PMID: 23197065.

152. Hosseinian A, Mohammadzadeh A, Shahmohammadi G, Hasanpour M, Maleki N, Doustkami H, et al. Rupture of a giant cardiac hydatid cyst in the left ventricular free wall: successful surgical management of a rare entity. Am J Cardiovasc Dis. 2013;3(2):103-6. Epub 2013/06/21. PubMed PMID: 23785588; PubMed Central PMCID: PMCPMC3683408.

153. Ibn Elhadj Z, Boukhris M, Kammoun I, Halima AB, Addad F, Kachboura S. Cardiac hydatid cyst revealed by ventricular tachycardia. J Saudi Heart Assoc. 2014;26(1):47-50. Epub 2014/03/01. doi: 10.1016/j.jsha.2013.08.001. PubMed PMID: 24578600; PubMed Central PMCID: PMCPMC3936243.

154. Ipek G, Omeroglu SN, Goksedef D, Balkanay OO, Kanbur E, Engin E, et al. Large cardiac hydatid cyst in the interventricular septum. Tex Heart Inst J. 2011;38(6):719-22. Epub 2011/12/27. PubMed PMID: 22199447; PubMed Central PMCID: PMCPMC3233322.

155. Juttner K, McKenzie L, Razzak N, Zehyle E, Belliere M, Anderson D, et al. Cardiac hydatid disease in the third world. BMJ Case Rep. 2011;2011. Epub 2011/01/01. doi: 10.1136/bcr.12.2010.3609. PubMed PMID: 22700616; PubMed Central PMCID: PMCPMC3079493.

156. Karam N, Azarine A, Menasche P, Hagege AA. An egg-shaped cyst of the right interventricular septum. Circulation. 2013;127(23):2351-2. Epub 2013/06/12. doi: 10.1161/circulationaha.113.001630. PubMed PMID: 23753846.

157. Kaya Z, Bicer A, Boyaci N, Sezen Y, Yildiz A, Sumbul S, et al. Eggshell-like appearance around the heart mimicking left ventricular mass. Circulation. 2013;127(23):e660-2. Epub 2013/06/12. doi: 10.1161/circulationaha.112.000759. PubMed PMID: 23753847.

158. Khattabi WE, Afif H, Berrada Z, Rhissassi J, Aichane A, Bouayad Z. [Multiple pulmonary hydatid disease with cardiovascular localisation]. Rev Mal Respir. 2011;28(5):686-90. Epub 2011/06/08. doi: 10.1016/j.rmr.2010.10.037. PubMed PMID: 21645843.

159. Koneti NR, Jagannath BR, Sreeram N. Cardiac hydatid cyst. Cardiol Young. 2013;23(4):606-7. Epub 2013/08/06. doi: 10.1017/s1047951112001801. PubMed PMID: 23910021.

160. Koubaa M, Lahiani D, Abid L, Maaloul I, Kahla SB, Bradii M, et al. Can albendazole be the only treatment for cardiac echinococcosis with multiple organ involvement? Int J Cardiol. 2012;161(3):e58-60. Epub 2012/05/09. doi: 10.1016/j.ijcard.2012.04.082. PubMed PMID: 22560938.

161. Kumar Paswan A, Prakash S, Dubey RK. Cardiac tamponade by hydatid pericardial cyst: a rare case report. Anesth Pain Med. 2014;4(1):e9137. Epub 2014/03/25. doi: 10.5812/aapm.9137. PubMed PMID: 24660148; PubMed Central PMCID: PMCPMC3961027.

162. Kurdal AT, Kahraman N, Iskesen I, Sirin BH. Unusual location of hydatid cyst: the posterior leaflet of tricuspid valve. Ann Ital Chir. 2010;81(3):211-4. Epub 2010/11/26. PubMed PMID: 21090559.

163. Kuyumcu ME, Yesil Y, Etgul S, Halacli B, Sunman H, Yavuz BB, et al. Inoperable isolated cardiac hydatid cyst controlled with albendazole in an older adult with dementia. Age Ageing. 2013;42(6):812-4. Epub 2013/08/07. doi: 10.1093/ageing/aft090. PubMed PMID: 23917482.

164. Leila A, Laroussi L, Abdennadher M, Msaad S, Frikha I, Kammoun S. A cardiac hydatid cyst underlying pulmonary embolism: a case report. Pan Afr Med J. 2011;8:12. Epub 2011/11/29. PubMed PMID: 22121421; PubMed Central PMCID: PMCPMC3201579.

165. Martinez Rodriguez R, Campello Ros D, Sopena Garcia R. [Anaesthetic treatment in the removal of a cardiac hydatid cyst]. Rev Esp Anestesiol Reanim. 2012;59(7):390-3. Epub 2012/07/14. doi: 10.1016/j.redar.2012.05.012. PubMed PMID: 22789614.

166. Merad Z, Belkralladi H, Zahzeh T, Kara Slimane D, Aoumer K, Mechouar B, et al. [Hydatid cyst of the heart: fatal outcome]. Med Trop (Mars). 2011;71(2):122. Epub 2011/06/24. PubMed PMID: 21695866.

167. Mestres CA, Toshani A, Hemdan A, Alewa AM, Bernal JM. Hydatid pericardial tamponade: a grape soup. Lancet. 2011;377(9780):1862. Epub 2011/05/10. doi: 10.1016/s0140-6736(10)61154-3. PubMed PMID: 21550108.

168. Mocumbi AO. Intracardiac masses in young Africans: case reports and a brief review of the literature. Cardiol Young. 2012;22(4):368-71. Epub 2012/03/10. doi: 10.1017/s1047951112000285. PubMed PMID: 22400896.

169. Mohosen S, Mirhosseini, Parsaiyan Z, Fakhri M. A case of cardiac hydatidosis: role for transesophageal echocardiography in evaluating bilateral pulmonary nodules. Pneumologia. 2013;62(2):110-2. Epub 2013/07/31. PubMed PMID: 23894793.

170. Molavipour A, Javan H, Moghaddam AA, Dastani M, Abbasi M, Ghahramani S. Combined medical and surgical treatment of intracardiac hydatid cysts in 11 patients. J Card Surg. 2010;25(2):143-6. Epub 2010/05/25. doi: 10.1111/j.1540-8191.2009.00973.x. PubMed PMID: 20492027.

171. Moorthy N, Ananthakrishna R, Rajendran R, Girish Gowda SL, Bhat SP, Nanjappa MC. Giant cardiac hydatid cyst: an uncommon cause of cardiomegaly. J Am Coll Cardiol. 2013;62(16):e145. Epub 2013/08/27. doi: 10.1016/j.jacc.2013.04.103. PubMed PMID: 23973685.

172. Moorthy N, Kumar S, Lal H, Goel PK. Incidental detection of asymptomatic intrapericardial calcified hydatid cyst. Heart Views. 2013;14(1):36-7. Epub 2013/04/13. doi: 10.4103/1995-705x.107120. PubMed PMID: 23580925; PubMed Central PMCID: PMCPMC3621224.

173. Mustafa B, Ahmed W, Akbar MT. Hydatid cyst of the cardiac interventricular septum with complete atrioventricular block: a case report from Pakistan. J Pak Med Assoc. 2012;62(3):291-2. Epub 2012/07/07. PubMed PMID: 22764470.

174. Neuville M, Grisoli D, Nicoud A, Jacquier A, Lagier JC, Collart F, et al. Cardiac hydatidosis. Am J Trop Med Hyg. 2010;83(1):102-3. Epub 2010/07/03. doi: 10.4269/ajtmh.2010.10-0026. PubMed PMID: 20595485; PubMed Central PMCID: PMCPMC2912583.

175. Oc M, Ulular O, Oc B. Surgical treatment of right ventricular hydatid cyst. Heart Surg Forum. 2012;15(3):E167-9. Epub 2012/06/16. doi: 10.1532/hsf98.20111160. PubMed PMID: 22698608.

176. Ozbek IC, Sever K, Mansuroglu D, Kurtoglu N. Giant left ventricular hydatid cyst and its surgical treatment. Asian Cardiovasc Thorac Ann. 2012;20(6):743. Epub 2013/01/04. doi: 10.1177/0218492312439482. PubMed PMID: 23284130.

177. Ozkan M, Astarcioglu MA, Tuncer A, Gursoy O, Aykan AC. Cardiac cyst hydatid: a multimodality approach. Rev Esp Cardiol (Engl Ed). 2012;65(2):187-8. Epub 2011/07/26. doi: 10.1016/j.recesp.2011.04.009. PubMed PMID: 21784572.

178. Palmerini E, Federici D, Ballo P, Mondillo S, Chiavarelli M. Surgical management of cardiac hydatidosis. J Card Surg. 2011;26(3):292-3. Epub 2010/11/04. doi: 10.1111/j.1540-8191.2010.01140.x. PubMed PMID: 21044154.

179. Parvizi R, Namdar H, Bilehjani E, Bayat A, Sheikhalizadeh MA. Simultaneous operation of hydatid cyst of the heart and liver: a case report. J Cardiovasc Thorac Res. 2013;5(3):127-8. Epub 2013/11/21. doi: 10.5681/jcvtr.2013.027. PubMed PMID: 24252990; PubMed Central PMCID: PMCPMC3825392.

180. Rekik S, Krichene S, Sahnoun M, Trabelsi I, Kharrat I, Charfeddine H, et al. Unusual cause of syncope in a 17 year-old young woman: left ventricular hydatid cyst. Int J Cardiol. 2009;136(1):e21-3. Epub 2008/07/29. doi: 10.1016/j.ijcard.2008.04.077. PubMed PMID: 18656270.

181. Rowell A, Figtree M, Dimmick S, Kotsiou G, Grieve SM, Figtree GA. Recurrent right ventricular echinococcosis characterized by cardiac magnetic resonance. Int J Cardiol. 2012;158(2):293-4. Epub 2012/05/26. doi: 10.1016/j.ijcard.2012.04.127. PubMed PMID: 22626837.

182. Sabouni F, Ferdosian F, Mamishi S, Nejat F, Monnajemzadeh M, Rezaei N. Multiple organ involvement with hydatid cysts. Iran J Parasitol. 2010;5(2):65-70. Epub 2010/06/01. PubMed PMID: 22347246; PubMed Central PMCID: PMCPMC3279838.

183. Sabzi F, Faraji R. A giant hydatid cyst in the interventricular septum with papillary muscle involvement. Korean J Parasitol. 2013;51(3):349-52. Epub 2013/07/19. doi: 10.3347/kjp.2013.51.3.349. PubMed PMID: 23864747; PubMed Central PMCID: PMCPMC3712110.

184. Sai Chandran BV, Durga Prasad R, Sudarsan Reddy P, Subbarao VK. Intramyocardial hydatid cyst: a mistaken identity and its successful removal on a beating heart. Interact Cardiovasc Thorac Surg. 2011;13(2):208-10. Epub 2011/05/21. doi: 10.1510/icvts.2011.267518. PubMed PMID: 21596759.

185. Selmi K, Bergaoui H, Tlili R, Khayati A, Boujnah MR. Live three-dimensional transthoracic echocardiography of left ventricular intramyocardial hydatid cyst. Eur Heart J Cardiovasc Imaging. 2012;13(1):120. Epub 2011/09/22. doi: 10.1093/ejechocard/jer168. PubMed PMID: 21933793; PubMed Central PMCID: PMCPMC3366597.

186. Shankarappa RK, Moorthy N, Bhat P, Nanjappa MC. Left ventricular hydatid cyst: an uncommon cause of chest pain in young adults. Cardiol Young. 2013;23(3):460-2. Epub 2012/09/26. doi: 10.1017/s1047951112001230. PubMed PMID: 23006608.

187. Shetty R, Vivek G, Nayak S, Dias L. A mysterious visitor to the heart. BMJ Case Rep. 2012;2012. Epub 2012/08/04. doi: 10.1136/bcr-03-2012-6005. PubMed PMID: 22859381; PubMed Central PMCID: PMCPMC4542618.

188. Singh H, Mohite P. Solitary pericardial hydatid cyst encompassing the heart. Eur J Cardiothorac Surg. 2012;42(5):901. Epub 2012/10/23. doi: 10.1093/ejcts/ezs249. PubMed PMID: 23087093.

189. Sogunuru G, Murty DS, Chinta VR, Vuddanda K, Moka N. Cardiac hydatidosis presenting as an acute coronary syndrome. BMJ Case Rep. 2010;2010. Epub 2010/01/01. doi: 10.1136/bcr.02.2010.2752. PubMed PMID: 22752832; PubMed Central PMCID: PMCPMC3034206.

190. Tekbas EO, Tekbas G, Atilgan ZA, Islamoglu Y, Cil H, Yazici M. Left ventricle hydatid cyst mimicking acute coronary syndrome. J Infect Dev Ctries. 2012;6(7):579-83. Epub 2012/07/31. PubMed PMID: 22842945.

191. Tekin R, Kara AF, Tekin RC, Cimen D. Cardiac hydatid cyst case recovered with medical treatment. Anadolu Kardiyol Derg. 2011;11(7):650-1. Epub 2011/10/05. doi: 10.5152/akd.2011.172. PubMed PMID: 21967788.

192. Tokgoz HC, Tanboga IH, Uslu ZA, Tuncer A, Kaymaz C. Huge intramyocardial echinococcal cyst resulting in a significant left ventricular cavity obliteration evaluated by real-time 3-dimensional transesophageal echocardiography and multidetector computed tomography before and after complete excision. Circulation. 2011;124(15):1692-3. Epub 2011/10/12. doi: 10.1161/circulationaha.110.985432. PubMed PMID: 21986776.

193. Tomar AS, Ganjoo P, Satsangi DK, Trehan V, Tempe DK. Interatrial septum hydatid cyst removal under cardiopulmonary bypass. Indian Heart J. 2010;62(5):456-7. Epub 2010/09/01. PubMed PMID: 23189891.

194. Toufan M, Afrasiabi A, Pourafkari L. Isolated cardiac hydatidosis--a case report. Kardiol Pol. 2010;68(2):223-5. Epub 2010/03/20. PubMed PMID: 20301035.

195. Toufan M, Pourafkari L. Isolated cardiac hydatid cyst. Am J Med Sci. 2010;340(5):412. Epub 2010/06/22. doi: 10.1097/MAJ.0b013e3181c3b1ab. PubMed PMID: 20562692.

196. Tsigkas G, Chouchoulis K, Apostolakis E, Kalogeropoulou C, Koutsogiannis N, Koumoundourou D, et al. Heart echinococcus cyst as an incidental finding: early detection might be life-saving. J Cardiothorac Surg. 2010;5:124. Epub 2010/12/15. doi: 10.1186/1749-8090-5-124. PubMed PMID: 21143844; PubMed Central PMCID: PMCPMC3004882.

197. Tulay CM. Primary mediastinal hydatid cysts. Ann Thorac Cardiovasc Surg. 2014;20(4):316-9. Epub 2013/06/27. doi: 10.5761/atcs.oa.13.02273. PubMed PMID: 23801183.

198. Tuncer E, Tas SG, Mataraci I, Tuncer A, Donmez AA, Aksut M, et al. Surgical treatment of cardiac hydatid disease in 13 patients. Tex Heart Inst J. 2010;37(2):189-93. Epub 2010/04/20. PubMed PMID: 20401292; PubMed Central PMCID: PMCPMC2851433.

199. Tuncer E, Turk U, Alioglu E. Cardiac hydatid cyst: an unusual cause of chest pain. Int Cardiovasc Res J. 2013;7(4):150-1. Epub 2014/04/24. PubMed PMID: 24757641; PubMed Central PMCID: PMCPMC3987446.

200. Turak O, Ozcan F, Sokmen E, Isleyen A, Okten RS, Tufekcioglu O, et al. Syncope as the primary manifestation of hydatid cyst. Report of two cases with different etiologies. Herz. 2014;39(2):287-90. Epub 2013/06/12. doi: 10.1007/s00059-013-3827-x. PubMed PMID: 23749195.

201. Turan T, Cakmak A, Capraz M, Aykan AC. Interventricular septal cardiac hydatid cyst mimicking hypertrophic cardiomyopathy. Anadolu Kardiyol Derg. 2013;13(3):E19-20. Epub 2013/02/28. doi: 10.5152/akd.2013.096. PubMed PMID: 23443872.

202. Ucar O, Cicekcioglu H, Demircelik B, Aydogdu S. Mitral regurgitation due to a hydatid cyst located in the left ventricular papillary muscle. Anadolu Kardiyol Derg. 2010;10(3):E10. Epub 2010/06/12. doi: 10.5152/akd.2010.080. PubMed PMID: 20538547.

203. Vaidyanathan KR, Vaijyanath P, Betigeri A, Cherian KM. Left ventricular epicardial hydatid cyst compressing the left circumflex artery. J Card Surg. 2009;24(4):483-4. Epub 2009/07/09. doi: 10.1111/j.1540-8191.2008.00742.x. PubMed PMID: 19583628.

204. Wang HB, Li M, Wen H. Isolated cardiac hydatid cyst. J Card Surg. 2010;25(6):702-3. Epub 2010/10/01. doi: 10.1111/j.1540-8191.2010.01121.x. PubMed PMID: 20880080.

205. Ward TJ, Jacobi AH, Mendelson DS, Lento PA. AIRP best cases in radiologic-pathologic correlation: cardiac echinococcus infection. Radiographics. 2013;33(5):1413-8. Epub 2013/09/13. doi: 10.1148/rg.335125209. PubMed PMID: 24025932.

206. Xing Y, Bawudong D, Zhang WB, Liu WY, Pan CX, Wen H, et al. Multidetector CT and MR imaging cardiac hydatidosis: case report and review of the literature. Int J Cardiovasc Imaging. 2011;27 Suppl 1:97-102. Epub 2011/10/21. doi: 10.1007/s10554-011-9958-3. PubMed PMID: 22012491.

207. Yalcin M, Isilak Z, Atalay M, Incedayi M. Symptomatic giant cardiac cyst hydatid in an adult woman. BMJ Case Rep. 2013;2013. Epub 2013/03/26. doi: 10.1136/bcr-2013-009148. PubMed PMID: 23524347; PubMed Central PMCID: PMCPMC3618702.

208. Yilmaz S, Ozhan M, Sager S, Halac M, Sonmezoglu K, Uslu I. Cardiac hydatid cyst mimicking malignancy. Clin Nucl Med. 2011;36(7):580-1. Epub 2011/06/04. doi: 10.1097/RLU.0b013e3182177232. PubMed PMID: 21637065.

209. Younis SN, Faraj AA. Cardiac hydatid disease, case report, and review of literature. Acta Clin Belg. 2014;69(1):66-8. Epub 2014/03/19. doi: 10.1179/0001551213z.0000000003. PubMed PMID: 24635402.

210. Yuksel S, Gulel O, Elmali M, Kale A, Sahin M. Huge cardiac cyst hydatid causing cardiac symptoms and electrocardiographic changes. Eur J Echocardiogr. 2009;10(8):991-2. Epub 2009/09/15. doi: 10.1093/ejechocard/jep109. PubMed PMID: 19749197.

211. Zaghba N, Yassine N, Bakhatar A, Bahlaoui A. [Multiple lung hydatidosis with cardiac, pulmonary artery and aortic localizations]. Rev Pneumol Clin. 2010;66(3):197-200. Epub 2010/06/22. doi: 10.1016/j.pneumo.2009.08.005. PubMed PMID: 20561486.

212. Zawaideh C, Aste M, Morelli MC, Rosa GM, Bezante GP. Recurrent hydatid cyst: ultrasound detection of an unusual cardiac embolic source. Eur Heart J Cardiovasc Imaging. 2013;14(9):934. Epub 2013/04/06. doi: 10.1093/ehjci/jet046. PubMed PMID: 23558023.

213. Zheng F, Wang X, Ma SF, Qiao J, Sheyhidin I. Surgical treatment of pericardial echinococcosis: report of eight cases. Chin Med J (Engl). 2013;126(3):591-2. Epub 2013/02/21. PubMed PMID: 23422133.

214. Abid A, Ben Omrane S, Kaouel K, Marghli A, Dhiab M, Abid N, et al. Intracavitary cardiac hydatid cyst. Cardiovasc Surg. 2003;11(6):521-5. Epub 2003/11/25. doi: 10.1016/s0967-2109(03)00116-9. PubMed PMID: 14627976.

215. Abounadi R, El Meziane A, El Biaze M, Bakhatar A, Yassine N, Alaoui-Yazidi A, et al. [Cardiac hydatidosis with dissemination to the pulmonary parenchyma and the pulmonary artery]. Rev Pneumol Clin. 2006;62(4):247-51. Epub 2006/11/01. PubMed PMID: 17075550.

216. Acarturk E, oZeren A, Koc M, Yaliniz H, Bicakci S, Demir M. Left ventricular hydatid cyst presenting with acute ischemic stroke: case report. J Am Soc Echocardiogr. 2004;17(9):1009-10. Epub 2004/09/01. doi: 10.1016/j.echo.2004.04.031. PubMed PMID: 15337971.

217. Achouh P, Grinda JM, Yvorra S, Fabiani JN. Hydatid cyst of the cardiac interventricular septum. Eur J Cardiothorac Surg. 2004;26(1):217-8. Epub 2004/06/18. doi: 10.1016/j.ejcts.2004.03.037. PubMed PMID: 15201008.

218. Al-Mahroos HM, Garadah TS, Aref MH, Al-Bannay RA. Cardiac echinococcosis: echocardiographic diagnosis with a fatal clinical outcome. Saudi Med J. 2005;26(11):1803-5. Epub 2005/11/29. PubMed PMID: 16311670.

219. Aleksic-Shihabi A, Vidolin EP. Cystic echinococcosis of the heart and brain: a case report. Acta Med Okayama. 2008;62(5):341-4. Epub 2008/11/06. doi: 10.18926/amo/30968. PubMed PMID: 18985095.

220. Altozano Gomez JC, Nogales Asensio JM, Arrobas Vaca J, Carretero Gomez J. [A 33-year-old male with abdominal pain and right heart failure]. Rev Clin Esp. 2005;205(7):353, 5-6. Epub 2005/07/21. PubMed PMID: 16029764.

221. Altun O, Akalin F, Ayabakan C, Karadag B, Berrak SG, Bilal MS, et al. Cardiac echinococcosis with intra-atrial localization. Turk J Pediatr. 2006;48(1):76-9. Epub 2006/03/28. PubMed PMID: 16562792.

222. Apaydin AZ, Oguz E, Ayik F, Nalbantgil S, Ceylan N. Hydatid cyst confined to the papillary muscle: a very rare cause of mitral regurgitation. Tex Heart Inst J. 2009;36(6):598-600. Epub 2010/01/14. PubMed PMID: 20069089; PubMed Central PMCID: PMCPMC2801946.

223. Arslan C, Canturk E, Duygu E, Bozkurt AK. Simultaneous hydatid cysts of both the right atrium and right ventricle. Acta Medica (Hradec Kralove). 2007;50(3):217-9. Epub 2008/02/08. PubMed PMID: 18254277.

224. Asri F, Tazi I, Maaroufi K, El Moudden A, Ghannane H, Ait Benali S. [Cerebral hydatic cyst and psychiatric disorders. Two cases]. Encephale. 2007;33(2):216-9. Epub 2007/08/07. PubMed PMID: 17675918.

225. Aydin NB, Celik S, Suzer A, Coruh T, Okay T, Gercekoglu H. Hydatid cyst in the wall of the ascending aorta. Asian Cardiovasc Thorac Ann. 2006;14(2):153-4. Epub 2006/03/23. doi: 10.1177/021849230601400215. PubMed PMID: 16551825.

226. Bakir I, Enc Y, Cicek S. Hydatid Cyst in the Pulmonary Artery: An Uncommon Localization. Heart Surg Forum. 2004;7(1):13-5. Epub 2004/02/26. PubMed PMID: 14980840.

227. Baksh SI, Alsaad KO, Chan NH. Pathologic quiz case: large cardiac mass in a 24-year-old woman. Cardiac hydatid cyst. Arch Pathol Lab Med. 2005;129(1):e23-4. Epub 2005/01/05. doi: 10.1043/1543-2165(2005)129<e23:PQCLCM>2.0.CO;2. PubMed PMID: 15628923.

228. Bal N, Kocer NE, Arpaci R, Ezer A, Kayaselcuk F. Uncommon locations of hydatid cyst. Saudi Med J. 2008;29(7):1004-8. Epub 2008/07/16. PubMed PMID: 18626530.

229. Barbetseas J, Lambrou S, Aggeli C, Vyssoulis G, Frogoudaki A, Tsiamis E, et al. Cardiac hydatid cysts: echocardiographic findings. J Clin Ultrasound. 2005;33(4):201-5. Epub 2005/04/28. doi: 10.1002/jcu.20108. PubMed PMID: 15856512.

230. Basavanagowdappa H, Prakash N, Srinivas A, Babu MS, Shenoy U, Nanaiah A, et al. Hydatid cyst of the right ventricle, liver and lungs. Indian Heart J. 2009;61(1):97-101. Epub 2009/09/05. PubMed PMID: 19729699.

231. Bell C, Yorgason J, Jessen ME, Josephs S, Jain T, Jeyarajah DR. Management of hepatic and intracardiac echinococcal cysts: case report and review of the literature. Surg Infect (Larchmt). 2006;7(3):309-13. Epub 2006/08/01. doi: 10.1089/sur.2006.7.309. PubMed PMID: 16875463.

232. Ben-Hamda K, Maatouk F, Ben-Farhat M, Betbout F, Gamra H, Addad F, et al. Eighteen-year experience with echinococcosus of the heart: clinical and echocardiographic features in 14 patients. Int J Cardiol. 2003;91(2-3):145-51. Epub 2003/10/16. doi: 10.1016/s0167-5273(03)00032-9. PubMed PMID: 14559124.

233. Biyik I, Acar S, Ergene O. Left atrial mobile hydatid cyst mimicking left atrial myxoma and mitral stenosis and causing heart failure and arrhythmia. Int J Cardiovasc Imaging. 2007;23(2):193-5. Epub 2006/07/27. doi: 10.1007/s10554-006-9132-5. PubMed PMID: 16868856.

234. Bouraoui H, Trimech B, Madhaoui A, Ernez-Hajri S, Jeridi G, Ammar H. [Hydatid cyst of the right atrium]. Rev Med Liege. 2006;61(1):8-10. Epub 2006/02/24. PubMed PMID: 16491541.

235. Bozbuga N, Erentug V, Akinci E, Yakut C. Is surgical therapy the only treatment of choice for cardiac echinococcosis with multiple organ involvement? Interact Cardiovasc Thorac Surg. 2003;2(3):367-8. Epub 2007/08/03. doi: 10.1016/s1569-9293(03)00072-0. PubMed PMID: 17670072.

236. Bozkurt AK, Koksal C. Cardiac echinococcosis in a 3-year-old child. J Cardiovasc Surg (Torino). 2004;45(2):125-7. Epub 2004/06/05. PubMed PMID: 15179347.

237. Brecker SJ, Mandal K, Harrison T, Griffin G, Varghese A, Pennell DJ, et al. Hydatid disease of the heart. Ann R Coll Surg Engl. 2005;87(2):W1-4. Epub 2006/06/23. doi: 10.1308/147870805x28064. PubMed PMID: 16790125; PubMed Central PMCID: PMCPMC1963872.

238. Celik T, Iyisoy A, Kursaklioglu H, Unlu M, Turhan H, Kose S. Intracavitary left ventricular hydatid cysts ruptured during cardiopulmonary resuscitation in a patient with acute myocardial infarction. Int J Cardiol. 2006;111(1):155-7. Epub 2005/07/06. doi: 10.1016/j.ijcard.2005.05.059. PubMed PMID: 15996775.

239. Cevirme D, Yerebakan C, Bayraktar S, Sunar H. Cardiac hydatid cyst of the interatrial septum. Wien Med Wochenschr. 2009;159(17-18):470-2. Epub 2009/10/14. doi: 10.1007/s10354-009-0707-3. PubMed PMID: 19823794.

240. Chadly A, Krimi S, Mghirbi T. Cardiac hydatid cyst rupture as cause of death. Am J Forensic Med Pathol. 2004;25(3):262-4. Epub 2004/08/24. PubMed PMID: 15322472.

241. Cruz-Gonzalez I, Martin-Herrero F, Gonzalez-Santos JM, Gutierrez-Diez JA, Sanchez-Ledesma M, Maree AO. Images in cardiovascular medicine. Anaphylaxis and recurrent hydatid disease. Circulation. 2007;115(25):e643-5. Epub 2007/06/27. doi: 10.1161/circulationaha.106.684126. PubMed PMID: 17592084.

242. Darcin OT, Kazaz H, Celkan A. Hydatid disease of the interventricular septum causing pulmonary dissemination. Acta Cardiol. 2003;58(5):431-3. Epub 2003/11/12. doi: 10.2143/ac.58.5.2005309. PubMed PMID: 14609310.

243. David EP, Fernandez MA, Fernandez-Aviles F. Isolated cardiac hydatidosis. Eur Heart J. 2007;28(23):2829. Epub 2007/07/20. doi: 10.1093/eurheartj/ehm249. PubMed PMID: 17636310.

244. Dell'Amore A, Botta L, Camurri N. Intramyocardial hydatic cyst in a young woman: Successful excision without cardiopulmonary by-pass. Heart Lung Circ. 2006;15(4):266. Epub 2006/09/28. PubMed PMID: 17004311.

245. Demirci S, Gunaydin G, Dogan KH, Toy H. Sudden death due to hydatid cyst rupture located in right ventricle. Am J Forensic Med Pathol. 2008;29(4):346-8. Epub 2009/03/05. doi: 10.1097/PAF.0b013e3181847e69. PubMed PMID: 19259023.

246. Demircin M, Dogan OF, Kanbak M. An unusual localization of cyst hydatidosis associated with cardiac hydatid disease. Anadolu Kardiyol Derg. 2007;7(1):103-4. Epub 2007/03/10. PubMed PMID: 17347095.

247. Derntl M, Bergler-Klein J, Lang IM. Images in cardiology: echinococcal cyst mimicking myocardial infarction. Heart. 2005;91(12):1536. Epub 2005/11/17. doi: 10.1136/hrt.2005.062919. PubMed PMID: 16287737; PubMed Central PMCID: PMCPMC1769211.

248. Djoshibaev S, Kudaiberdiev T, Maralov A, Shabraliev S, Djooshev K, Halikov UM, et al. Surgical treatment of isolated cardiac echinococciasis: report of five cases. Anadolu Kardiyol Derg. 2003;3(2):137-43. Epub 2003/06/27. PubMed PMID: 12826509.

249. Elangovan S, Harshavardan K, Meenakshi K, Swaminathan TS, Murthy P. Left ventricular hydatid cyst with myocardial infarction in a patient with severe rheumatic mitral stenosis. Indian Heart J. 2004;56(6):664-7. Epub 2005/03/09. PubMed PMID: 15751525.

250. Elhattaoui M, Charei N, Bennis A, Tahiri A, Chraibi N, Haddani J, et al. [Cardiac hydatid cysts: report of 10 cases]. Arch Mal Coeur Vaiss. 2006;99(1):19-25. Epub 2006/02/17. PubMed PMID: 16479885.

251. Erentug V, Bozbuga N, Kirali K, Mataraci I, Kaymaz C, Balkanay M, et al. Cardiac hydatid cysts: surgical treatment and results. J Card Surg. 2004;19(4):358-60. Epub 2004/07/13. doi: 10.1111/j.0886-0440.2004.4094_11.x. PubMed PMID: 15245471.

252. Erkut B, Unlu Y, Ozden K, Acikel M. Cardiac echinococcosis: recurrent intramyocardial-extracardiac hydatid cysts with pericardial protrusion. Circ J. 2008;72(10):1718-20. Epub 2008/08/30. doi: 10.1253/circj.cj-07-1063. PubMed PMID: 18728338.

253. Eroglu E, Gemici G, Ergenoglu M, Yildiz C, Kucukaksu S, Degertekin M. Giant hydatid cyst of the interventricular septum mimicking acute myocardial infarction on ECG: an unusual cause of ST segment elevation. J Cardiovasc Med (Hagerstown). 2009;10(5):425-7. Epub 2009/03/21. doi: 10.2459/JCM.0b013e328329335e. PubMed PMID: 19300277.

254. Fertin M, Mouquet F, Lallemant R, Gaxotte V, Decoene C, Larrue B, et al. Diagnosis, imaging, and treatment of an unusual cardiac hydatid cyst. Cardiovasc Pathol. 2006;15(6):356-8. Epub 2006/11/23. doi: 10.1016/j.carpath.2006.08.004. PubMed PMID: 17113016.

255. Garot J, Unterseeh T, Morice MC. Comprehensive cardiovascular magnetic resonance for diagnosis of cardiac hydatid cyst. Eur Heart J. 2008;29(17):2069. Epub 2008/03/14. doi: 10.1093/eurheartj/ehn090. PubMed PMID: 18337235.

256. Ghrairi H, Khouadja MA, Abouda M, Ammar J, Hantous S, Kilani T, et al. [Hydatid cyst of the heart and vessels, four cases]. Presse Med. 2005;34(2 Pt 1):101-4. Epub 2005/02/03. PubMed PMID: 15687977.

257. Goksel OS, Tanju S, Surmen B, El H, Tireli E, Dayioglu E. Recurrent apical cardiac hydatid cyst presenting with angina. Acta Chir Belg. 2008;108(6):783-5. Epub 2009/02/27. PubMed PMID: 19241943.

258. Gormus N, Durgut K, Ozergin U, Solak H. Suppurated mediastinal and cardiac echinococcosis: report of a case. Surg Today. 2005;35(8):668-70. Epub 2005/07/22. doi: 10.1007/s00595-002-2979-8. PubMed PMID: 16034548.

259. Gormus N, Yeniterzi M, Telli HH, Solak H. The clinical and surgical features of right-sided intracardiac masses due to echinococcosis. Heart Vessels. 2004;19(3):121-4. Epub 2004/05/29. doi: 10.1007/s00380-003-0732-x. PubMed PMID: 15168059.

260. Gulcan O, Turkoz R, Oguzkurt L, Tercan F, Sezgin A. Unusual clinical presentation of isolated cardiac hydatid cyst. Anadolu Kardiyol Derg. 2006;6(1):111. Epub 2006/03/10. PubMed PMID: 16524825.

261. Gundogdu F, Arslan S, Kantarci AM. Intramyocardial echinococcal cyst demonstrated by multislice computed tomography. Heart. 2006;92(10):1479. Epub 2006/09/16. doi: 10.1136/hrt.2005.082826. PubMed PMID: 16973802; PubMed Central PMCID: PMCPMC1861030.

262. Guney MR, Ketenci B, Cimen S, Ozay B, Aksoy S, Cinar B, et al. Management of cardiac hydatid cyst disease. Saudi Med J. 2008;29(1):150-2. Epub 2008/01/08. PubMed PMID: 18176696.

263. Gurbuz A, Tetik O, Yilik L, Emrecan B, Ozsoyler I, Ozbek C. Cardiac involvement of hydatid disease. Jpn J Thorac Cardiovasc Surg. 2003;51(11):594-8. Epub 2003/12/03. doi: 10.1007/bf02736699. PubMed PMID: 14650589.

264. Guven A, Sokmen G, Yuksel M, Kokoglu OF, Koksal N, Cetinkaya A. A case of asymptomatic cardiopericardial hydatid cyst. Jpn Heart J. 2004;45(3):541-5. Epub 2004/07/09. doi: 10.1536/jhj.45.541. PubMed PMID: 15240975.

265. Hassine E, Kraoua S, Marniche K, Bousnina S, Lefi A, Kachboura S, et al. [A dead and calcified right ventricular hydatic cyst. The limits of imaging]. Presse Med. 2003;32(38):1802-4. Epub 2003/12/10. PubMed PMID: 14663381.

266. Henaine R, Mathevet JL, Rouviere H, Di-Filippo S, Cannesson M, Obadia JF, et al. Coronary artery bypass in myocardial ischemia of the young due to hydatid cyst. J Card Surg. 2008;23(5):573-5. Epub 2008/07/16. doi: 10.1111/j.1540-8191.2008.00626.x. PubMed PMID: 18624986.

267. Heye T, Lichtenberg A, Junghanss T, Hosch W. Cardiac manifestation of cystic echinococcosis: comparison of dual-source cardio-computed tomography and cardiac magnetic resonance imaging and their impact on disease management. Am J Trop Med Hyg. 2007;77(5):875-7. Epub 2007/11/07. PubMed PMID: 17984345.

268. Ileri M, Hisar I, Atak R, Senen K, Aras D, Buyukasik N. A pericardial hydatid cyst masquerading as acute inferolateral myocardial infarction--a case report. Angiology. 2005;56(5):637-40. Epub 2005/09/30. doi: 10.1177/000331970505600518. PubMed PMID: 16193206.

269. Ilic S, Parezanovic V, Djukic M, Kalangos A. Ruptured hydatid cyst of the interventricular septum with acute embolic pulmonary artery complications. Pediatr Cardiol. 2008;29(4):855-7. Epub 2008/01/05. doi: 10.1007/s00246-007-9176-x. PubMed PMID: 18175164.

270. Iltumur K, Karabulut A, Toprak N. Recurrent multiple cardiac hydatidosis. Eur J Echocardiogr. 2005;6(4):294-6. Epub 2005/07/05. doi: 10.1016/j.euje.2004.10.011. PubMed PMID: 15992715.

271. Jaafari A, Nedia F, Boukhriss B, Ehlem B, Moez T, Habib BM. [Fatal hydatid pulmonary embolism. Report of two cases]. Ann Cardiol Angeiol (Paris). 2009;58(2):125-8. Epub 2008/07/19. doi: 10.1016/j.ancard.2008.05.009. PubMed PMID: 18635154.

272. Jimenez Casso S, Puente Sanchez MC, Fernandez Pineda L, Palmeiro Uriach A. [Solid cardiac mass due to hydatidosis]. An Pediatr (Barc). 2005;62(4):383-5. Epub 2005/04/14. PubMed PMID: 15826571.

273. Jouhadi Z, Ailal F, Dreoua N, Eddine AZ, Abid A, Skalli A, et al. [Cardiac hydatid cyst. Two cases in children]. Presse Med. 2004;33(18):1260-3. Epub 2004/12/22. PubMed PMID: 15611705.

274. Kakouros S, Kakouros N, Alchanatis M, Karydes K. Cardiac echinococcosis. Echocardiography. 2006;23(9):807-9. Epub 2006/09/27. doi: 10.1111/j.1540-8175.2006.00317.x. PubMed PMID: 16999705.

275. Kammoun I, Ben Halima A, Ammar J, Chine S, Chaabane O, Zouaoui W, et al. [Hydatic pulmonary embolism complicating a cardiac hydatic cyst. A case report]. Tunis Med. 2004;82(8):777-80. Epub 2004/11/10. PubMed PMID: 15532775.

276. Karabay O, Onen A, Yildiz F, Yilmaz E, Erdal CA, Sanli A, et al. The case of a cyst hydatid localized within the interatrial septum. Jpn Heart J. 2004;45(4):703-7. Epub 2004/09/09. doi: 10.1536/jhj.45.703. PubMed PMID: 15353883.

277. Karadede A, Alyan O, Sucu M, Karahan Z. Coronary narrowing secondary to compression by pericardial hydatid cyst. Int J Cardiol. 2008;123(2):204-7. Epub 2007/03/14. doi: 10.1016/j.ijcard.2006.11.174. PubMed PMID: 17350701.

278. Katewa A, Vaideeswar P, Khandekar JV, Sajid S, Jawale RM, Agrawal NB, et al. Isolated pericardial and intracardiac hydatidosis: presentation as congestive cardiac failure and fatal pulmonary embolism. Cardiovasc Pathol. 2009;18(2):114-8. Epub 2008/04/12. doi: 10.1016/j.carpath.2007.11.001. PubMed PMID: 18402821.

279. Kelle S, Kohler U, Thouet T, Fleck E, Nagel E. Cardiac involvement of Echinococcus granulosus evaluated by multi-contrast CMR imaging. Int J Cardiol. 2009;131(2):e59-60. Epub 2007/08/21. doi: 10.1016/j.ijcard.2007.05.114. PubMed PMID: 17707527.

280. Kervan U, Bardakci H, Altintas G, Tufekcioglu O, Birincioglu CL. A case of intraventricular septum dissection presenting with aneurysmal dilatation through the outflow track of the left ventricle. J Card Surg. 2008;23(2):173-6. Epub 2008/02/29. doi: 10.1111/j.1540-8191.2007.00509.x. PubMed PMID: 18304139.

281. Kolsi M, Frikha I, Triki N, Ayadi H, Siala I, Ayoub A, et al. [Cardiac multifocal hydatid cyst]. Arch Mal Coeur Vaiss. 2005;98(1):75-7. Epub 2005/02/24. PubMed PMID: 15724425.

282. Kosar F, Aksoy Y, Sahin I, Erdil N. Pericardial hydatid cyst mimicking acute coronary syndrome. Tex Heart Inst J. 2005;32(4):570-2. Epub 2006/01/25. PubMed PMID: 16429906; PubMed Central PMCID: PMCPMC1351833.

283. Kosecik M, Karaoglanoglu M, Yamak B. Pericardial hydatid cyst presenting with cardiac tamponade. Can J Cardiol. 2006;22(2):145-7. Epub 2006/02/18. doi: 10.1016/s0828-282x(06)70254-9. PubMed PMID: 16485050; PubMed Central PMCID: PMCPMC2538998.

284. Mahdhaoui A, Bouraoui H, Khelif A, Majdoub MA, Jeridi G, Hajri SE, et al. Hydatid cyst of the right atrium and rheumatic mitral disease: a fortuitous association. J Heart Valve Dis. 2003;12(4):535-7. Epub 2003/08/16. PubMed PMID: 12918860.

285. Mahdhaoui A, Bouraoui H, Souissi J, Mabrouk KH, Bahri F, Amara H, et al. [Double location of cardiac hydatid cyst: left ventricle and pulmonary artery]. Rev Med Interne. 2004;25(1):94-6. Epub 2004/01/23. doi: 10.1016/j.revmed.2003.09.011. PubMed PMID: 14736568.

286. Makaryus AN, Hametz C, Mieres J, Kort S, Carneglia J, Mangion J. Diagnosis of suspected cardiac echinococcosis with negative serologies: role of transthoracic, transesophageal, and contrast echocardiography. Eur J Echocardiogr. 2004;5(3):223-7. Epub 2004/05/19. doi: 10.1016/j.euje.2003.08.001. PubMed PMID: 15147665.

287. Malagnino V, Falzarano SM. [Primary cardiac echinococcosis]. Pathologica. 2006;98(4):232-4. Epub 2006/12/21. PubMed PMID: 17175792.

288. Mansuroglu D, Omeroglu SN, Akdemir R, Omeroglu A, Uzun K, Ipek G, et al. Right atrial hydatid cyst prolapsing into the tricuspid valve. Tex Heart Inst J. 2004;31(4):452-3. Epub 2005/03/05. PubMed PMID: 15745306; PubMed Central PMCID: PMCPMC548256.

289. Mitrev ZK, Anguseva TN. Complex reconstruction of hydatid cyst-destructed left ventricle: a case report. Heart Surg Forum. 2006;9(1):E490-2. Epub 2005/12/02. doi: 10.1532/hsf98.20051131. PubMed PMID: 16318934.

290. Mohsen T, El Beharry N, Maree T, Akl ES. Cardiac echinococcosis of the interventricular septum in early childhood: report of two cases. J Thorac Cardiovasc Surg. 2009;137(1):e14-6. Epub 2009/01/22. doi: 10.1016/j.jtcvs.2008.04.008. PubMed PMID: 19154866.

291. Muller MF. Images in cardiovascular medicine. Complicated case of left ventricular echinococcosis. Circulation. 2004;110(2):e13-4. Epub 2004/07/14. doi: 10.1161/01.Cir.0000135584.93927.B6. PubMed PMID: 15249518.

292. Muthu SK, Thiagarajan A, Govindarajan S, Coutinho CM, Gunawardena WJ, Swaminathan TS. Hydatid cyst of the cardiac interventricular septum: report of two cases. Br J Radiol. 2007;80(959):e278-82. Epub 2007/11/09. doi: 10.1259/bjr/78279821. PubMed PMID: 17989328.

293. Natarajan D, Bera M. Hydatid cyst of the heart. Heart. 2007;93(7):847. Epub 2007/06/16. doi: 10.1136/hrt.2006.095869. PubMed PMID: 17569807; PubMed Central PMCID: PMCPMC1994458.

294. Nemes A, Geleijnse ML, van Geuns RJ, Caliskan K, Michels M, Soliman OI, et al. Evaluation of pericardial hydatid cysts by different echocardiographic imaging modalities. Int J Cardiovasc Imaging. 2006;22(5):647-51. Epub 2006/04/21. doi: 10.1007/s10554-006-9089-4. PubMed PMID: 16625312.

295. Niarchos C, Kounis GN, Frangides CR, Koutsojannis CM, Batsolaki M, Gouvelou-Deligianni GV, et al. Large hydatic cyst of the left ventricle associated with syncopal attacks. Int J Cardiol. 2007;118(1):e24-6. Epub 2007/03/21. doi: 10.1016/j.ijcard.2006.11.251. PubMed PMID: 17368584.

296. Nimeri NA, Ali SK. Diagnosis and management of pediatric cardiac hydatid cyst. Saudi Med J. 2007;28(3):469. Epub 2007/03/06. PubMed PMID: 17334485.

297. Nisanoglu V, Erdil N, Isik B, Battaloglu B, Alat I. Acute abdominal aorta embolism caused by rupture of a cardiac hydatid cyst. Ann Vasc Surg. 2004;18(4):484-6. Epub 2004/05/25. doi: 10.1007/s10016-004-0061-2. PubMed PMID: 15156365.

298. Nurkalem Z, Atmaca H, Kayacioglu I, Uslu N, Gorgulu S, Eren M. Hydatid disease involving the left ventricle: a case of unusual combination. Int J Cardiol. 2006;112(2):e30-2. Epub 2006/07/25. doi: 10.1016/j.ijcard.2006.03.068. PubMed PMID: 16859775.

299. Olgun H, Karacan M, Ceviz N, Kocak H. Cardiac hydatid cyst. Eurasian J Med. 2009;41(1):73. Epub 2009/04/01. PubMed PMID: 25610070; PubMed Central PMCID: PMCPMC4261649.

300. Omay O, Celebi S, Kumbasar U, Babaoglu K, Gunay I. Interventricular hydatid cyst imitating pulmonary stenosis. Heart Surg Forum. 2008;11(1):E30-1. Epub 2008/02/14. doi: 10.1532/hsf98.20071168. PubMed PMID: 18270135.

301. Omrani GR, Kargar F, Aazami MH. Intra-pericardial hydatid cyst seen by dynamic computed tomography. Eur J Cardiothorac Surg. 2006;30(5):805. Epub 2006/09/19. doi: 10.1016/j.ejcts.2006.07.031. PubMed PMID: 16979344.

302. Ozturk E, Ozturk A, Zeyrek F, Demirbag R, Temamogullari AV. Recurrent pulmonary microemboli secondary to primary cardiac hydatidosis. Heart Lung Circ. 2007;16(6):457-9. Epub 2007/02/23. doi: 10.1016/j.hlc.2006.10.002. PubMed PMID: 17314071.

303. Ozyurtkan MO, Kocyigit S, Cakmak M, Ozsoy IE, Balci AE. Case report: mediastinal hydatid cysts. Turkiye Parazitol Derg. 2009;33(2):179-81. Epub 2009/07/15. PubMed PMID: 19598101.

304. Pakis I, Akyildiz EU, Karayel F, Turan AA, Senel B, Ozbay M, et al. Sudden death due to an unrecognized cardiac hydatid cyst: three medicolegal autopsy cases. J Forensic Sci. 2006;51(2):400-2. Epub 2006/03/29. doi: 10.1111/j.1556-4029.2006.00056.x. PubMed PMID: 16566779.

305. Prakash EB. An isolated cardiac hydatid cyst. J Assoc Physicians India. 2005;53:543. Epub 2005/08/27. PubMed PMID: 16121809.

306. Ren H, Zhang H, Zhang CJ, Du ZZ. Cardiac echinococcosis. Chin Med Sci J. 2005;20(4):276-8. Epub 2006/01/21. PubMed PMID: 16422260.

307. Rena O, Garavoglia M, Francini M, Bellora P, Oliaro A, Casadio C. Solitary pericardial hydatid cyst. J Cardiovasc Surg (Torino). 2004;45(1):77-80. Epub 2004/03/26. PubMed PMID: 15041943.

308. Rezaian GR, Aslani A. Endocardial hydatid cyst: a rare presentation of echinococcal infection. Eur J Echocardiogr. 2008;9(2):342-3. Epub 2008/05/21. doi: 10.1093/ejechocard/jen020. PubMed PMID: 18490334.

309. Saad RA, Amer KM, Migliore M, Aziz T, Azzu A. Right intraventricular hydatid cyst of the heart. Asian Cardiovasc Thorac Ann. 2003;11(2):160-2. Epub 2003/07/25. doi: 10.1177/021849230301100216. PubMed PMID: 12878567.

310. Salehi M, Soleimani A. Cardiac echinococcosis with negative serologies: a report of two cases. Heart Lung Circ. 2009;18(1):59-61. Epub 2007/12/18. doi: 10.1016/j.hlc.2007.08.006. PubMed PMID: 18082448.

311. Savas L, Onlen Y, Akcali C, Aslan B, Pourbagher A, Tunc T, et al. Hydatid disease with atypical localization: 4 cases report. Scand J Infect Dis. 2004;36(8):613-5. Epub 2004/09/17. doi: 10.1080/00365540410017581. PubMed PMID: 15370676.

312. Selcuk MT, Selcuk H, Tufekcioglu O, Baser K, Ozbulbul NI, Birincioglu L, et al. A hydatid cyst of the interventricular septum diagnosed incidentally by multislice computed tomography. Heart Lung Circ. 2008;17(4):347-9. Epub 2007/03/06. doi: 10.1016/j.hlc.2006.12.011. PubMed PMID: 17336156.

313. Senkaya I, Bostan OM, Celebi S, Cil E. Multiple hydatid cysts of pericardium and epicardium. Anadolu Kardiyol Derg. 2004;4(1):82-4. Epub 2004/03/23. PubMed PMID: 15033625.

314. Sensoz Y, Ozkokeli M, Ates M, Akcar M. Right ventricle hydatid cyst requiring tricuspid valve excision. Int J Cardiol. 2005;101(2):339-41. Epub 2005/05/11. doi: 10.1016/j.ijcard.2004.01.043. PubMed PMID: 15882692.

315. Sevimli S, Aksakal E, Erkut B, Ceviz M, Turkyilmaz A, Karakelleoglu S, et al. Three-dimensional imaging of a comma-shaped right ventricular hydatid cyst. Echocardiography. 2009;26(4):481-4. Epub 2009/04/23. doi: 10.1111/j.1540-8175.2008.00880.x. PubMed PMID: 19382948.

316. Shah JR, Joshi A, Patkar D. A young woman with multiple cardiac mass lesions. Br J Radiol. 2009;82(976):344-7. Epub 2009/03/28. doi: 10.1259/bjr/64927200. PubMed PMID: 19325048.

317. Shehatha J, Alward M, Saxena P, Konstantinov IE. Surgical management of cardiac hydatidosis. Tex Heart Inst J. 2009;36(1):72-3. Epub 2009/05/14. PubMed PMID: 19436793; PubMed Central PMCID: PMCPMC2676529.

318. Sinha A, Nanda NC, Panwar RB, Kasliwal RR, Chauhan N, Beniwal S, et al. Live three-dimensional transthoracic echocardiographic assessment of left ventricular hydatid cyst. Echocardiography. 2004;21(8):699-705. Epub 2004/11/18. doi: 10.1111/j.0742-2822.2004.04005.x. PubMed PMID: 15546370.

319. Sirlak M, Ozcinar E, Eren NT, Eryilmaz S, Uysalel A, Enneli D, et al. Multiple hydatid cystectomy of the heart necessitating LIMA to LAD anastomosis in a young patient. Cardiovasc Pathol. 2009;18(1):53-6. Epub 2008/04/12. doi: 10.1016/j.carpath.2007.08.003. PubMed PMID: 18402806.

320. Soleimani A, Sahebjam M, Marzban M, Shirani S, Abbasi A. Hydatid cyst of the right ventricle in early pregnancy. Echocardiography. 2008;25(7):778-80. Epub 2008/08/30. doi: 10.1111/j.1540-8175.2008.00668.x. PubMed PMID: 18754937.

321. Soydinc S, Davutoglu V, Oylumlu M. Progress of untreated massive cardiac echinococcosis--echocardiographic follow-up. Echocardiography. 2006;23(9):780-3. Epub 2006/09/27. doi: 10.1111/j.1540-8175.2006.00310.x. PubMed PMID: 16999698.

322. Tandon S, Darbari A. Hydatid cyst of the right atrium: a rare presentation. Asian Cardiovasc Thorac Ann. 2006;14(3):e43-4. Epub 2006/05/23. doi: 10.1177/021849230601400324. PubMed PMID: 16714680.

323. Tasdemir K, Akcali Y, Gunebakmaz O, Kaya MG, Mavili E, Sarli B, et al. Surgical approach to the management of cardiovascular echinococcosis. J Card Surg. 2009;24(3):281-4. Epub 2009/05/15. doi: 10.1111/j.1540-8191.2008.00727.x. PubMed PMID: 19438781.

324. Tercan F, Kacar N, Kilic D, Oguzkurt L, Turkoz R, Habesoglu MA. Hydatid cysts of the bilateral pulmonary arteries and left ventricle wall: computed tomography and magnetic resonance imaging findings. J Comput Assist Tomogr. 2005;29(1):31-3. Epub 2005/01/25. doi: 10.1097/01.rct.0000153405.09602.c5. PubMed PMID: 15665679.

325. Tiryakioglu O, Vural H, Ozyazicioglu AF. The curative excision of left ventricular hydatic cyst. Heart Lung Circ. 2009;18(1):57-8. Epub 2007/12/18. doi: 10.1016/j.hlc.2007.08.007. PubMed PMID: 18082449.

326. Tiseo D, Borrelli F, Gentile I, Benassai G, Quarto G, Borgia G. [Cystic echinococcosis in humans: our clinic experience]. Parassitologia. 2004;46(1-2):45-51. Epub 2004/08/13. PubMed PMID: 15305685.

327. Tufekcioglu O, Birincioglu CL, Arda K, Fansa I, Saritas A, Karahan M. Echocardiography findings in 16 cases of cardiac echinococcosis: proposal for a new classification system. J Am Soc Echocardiogr. 2007;20(7):895-904. Epub 2007/07/10. doi: 10.1016/j.echo.2006.12.012. PubMed PMID: 17617317.

328. Tuğcu B, Günaldi O, Güneş M, Güler AK, Adilay U, Eseoğlu M, et al. Hydatid cysts in uncommon locations in the same patient: simultaneous cardiac and spinal involvement. Minim Invasive Neurosurg. 2008;51(4):234-6. doi: 10.1055/s-2008-1080904. PubMed PMID: 18683117.

329. Ugurlucan M, Sayin OA, Surmen B, Cinar T, Yekeler E, Dursun M, et al. Images in cardiovascular medicine. Hydatid cyst of the interventricular septum. Circulation. 2006;113(24):e869-70. Epub 2006/06/21. doi: 10.1161/circulationaha.105.595298. PubMed PMID: 16785345.

330. Ulas MM, Kocabeyoglu SS, Yener AU, Pac M. Myocardial cyst of the left ventricle with two heads invasing pericardium. J Card Surg. 2009;24(2):200. Epub 2009/03/10. doi: 10.1111/j.1540-8191.2008.00733.x. PubMed PMID: 19267833.

331. Ulgen MS, Yazici M, Kayrak M, Duzenli MA, Koc F. Three-year follow up of recurrent cardiac echinococcosis simulating myxoma: report of a rare case. Anadolu Kardiyol Derg. 2007;7(4):442-3. Epub 2007/12/11. PubMed PMID: 18065346.

332. Vural M, Sayin B, Pasaoglu L, Koparal S, Elverici E, Dede D. Isolated pericardial hydatid cyst in an asymptomatic patient: a remark on its radiologic diagnosis. Clin Imaging. 2007;31(1):37-9. Epub 2006/12/27. doi: 10.1016/j.clinimag.2006.10.002. PubMed PMID: 17189845.

333. Yaliniz H, Tokcan A, Salih OK, Ulus T. Surgical treatment of cardiac hydatid disease: A report of 7 cases. Tex Heart Inst J. 2006;33(3):333-9. Epub 2006/10/17. PubMed PMID: 17041691; PubMed Central PMCID: PMCPMC1592285.

334. Yaliniz H, Tokcan A, Ulus T, Kisacikoglu B, Salih OK, Topcuoglu MS, et al. A rare presentation of cardiac hydatid cyst: stroke and acute aortic occlusion. Heart Surg Forum. 2004;7(5):E364-6. Epub 2005/04/01. doi: 10.1532/hsf98.20041056. PubMed PMID: 15799904.

335. Yilik L, Ergunes K, Yetkin U, Ali G. Synchronized coronary revascularization and multiple cardiac cysthydatid operation. Heart Lung. 2004;33(1):42-5. Epub 2004/02/26. doi: 10.1016/j.hrtlng.2003.10.002. PubMed PMID: 14983138.

336. Yilmaz M, Senkaya I, Kaderli A, Ener S. Complete atrioventricular block due to a hydatid cyst located in the interventricular septum: a case report. Heart Surg Forum. 2007;10(1):E9-e11. Epub 2006/12/13. doi: 10.1532/hsf98.20061126. PubMed PMID: 17162409.

337. Yilmazkaya B, Yondem OZ, Gurkahraman S, Yukselen MA, Circi R, Tasdemir O. Direct approach to hydatid cyst of the interventricular septum. Tex Heart Inst J. 2009;36(2):174-6. Epub 2009/05/14. PubMed PMID: 19436819; PubMed Central PMCID: PMCPMC2676597.

338. Zobel C, Kuhn-Regnier F, Kruger K, Gerharz M, Schneider CA, Muller-Ehmsen J, et al. Echinococcus cyst located in the interventricular septum. Clin Res Cardiol. 2006;95(11):600-4. Epub 2006/08/10. doi: 10.1007/s00392-006-0428-1. PubMed PMID: 16897142.

339. Afif H, Aichane A, Trombati N, Bahlaoui A, Bouayad Z, Boumzebra M, et al. [Multiple pulmonary hydatidosis with floating balloon appearance and cardiac localization]. Rev Mal Respir. 2000;17(3):697-9. Epub 2000/08/22. PubMed PMID: 10951967.

340. Agarwal DK, Agarwal R, Barthwal SP. Interventricular septal hydatid cyst presenting as complete heart block. Heart. 1996;75(3):266. Epub 1996/03/01. doi: 10.1136/hrt.75.3.266. PubMed PMID: 8800990; PubMed Central PMCID: PMCPMC484284.

341. Ahmed T, Al-Zaibag M, Allan A, Gabriel C, Widaa H, Hulaimi N, et al. Cardiac echinococcosis causing acute dissection of the left ventricular free wall. Echocardiography. 2002;19(4):333-6. Epub 2002/06/06. PubMed PMID: 12047786.

342. Ait Ben Ali S, Hilmani S, Choukri M, Sami A, El Azhari A, Achouri M, et al. [Multiple cerebral hydatic cysts of cardiac origin. A case report]. Neurochirurgie. 1999;45(5):426-9. Epub 2000/03/16. PubMed PMID: 10717596.

343. Alehan D, Celiker A, Aydingoz U. Cardiac hydatid cyst in a child: diagnostic value of echocardiography and magnetic resonance imaging. Acta Paediatr Jpn. 1995;37(5):645-7. Epub 1995/10/01. doi: 10.1111/j.1442-200x.1995.tb03395.x. PubMed PMID: 8533597.

344. Ali SM, McLaughlin JS. Intramyocardial echinococcus cyst. Ann Thorac Surg. 2002;73(5):1656. Epub 2002/05/23. doi: 10.1016/s0003-4975(01)02735-7. PubMed PMID: 12022580.

345. Atilgan D, Demirel S, Akkaya V, Korkut F. Left ventricular hydatid cyst: an unusual location of Echinococcus granulosus with multiple organ involvement. J Am Soc Echocardiogr. 1996;9(2):212-5. Epub 1996/03/01. doi: 10.1016/s0894-7317(96)90034-6. PubMed PMID: 8849622.

346. Atilgan D, Kudat H, Tukek T, Ozcan M, Yildirim OB, Elmaci TT, et al. Role of transesophageal echocardiography in diagnosis and management of cardiac hydatid cyst: report of three cases and review of the literature. J Am Soc Echocardiogr. 2002;15(3):271-4. Epub 2002/03/05. doi: 10.1067/mje.2002.120507. PubMed PMID: 11875393.

347. Aupetit JF, Ritz B, Ferrini M, Coppin M, Champsaur G. Images in cardiovascular medicine. Hydatid cyst of the interventricular septum. Circulation. 1997;95(9):2325-6. Epub 1997/05/06. doi: 10.1161/01.cir.95.9.2325. PubMed PMID: 9142012.

348. Aydin NE, Ege E, Selcuk MA, Erguvan R. Echinococcal hydatid cyst at the right ventricle outlet with leakage to the pulmonary artery outflow causing follicular airway disease and sudden death. Am J Forensic Med Pathol. 2001;22(2):165-8. Epub 2001/06/08. doi: 10.1097/00000433-200106000-00010. PubMed PMID: 11394751.

349. Aydogdu T, Sahin N, Ulusan V, Gurpinar F, Turkay C, Bayezid O. Right atrial hydatid cyst associated with multiple organ involvement: case report. J Thorac Cardiovasc Surg. 2001;121(5):1009-11. Epub 2001/04/28. doi: 10.1067/mtc.2001.112628. PubMed PMID: 11326256.

350. Baque J, Huart V, Pierrot JM, Louail B, Grinda JM, Sapoval M, et al. [Hydatid cyst of the heart interventricular septum: multidetector CT scan and MRI findings]. J Radiol. 2003;84(5):614-6. Epub 2003/09/19. PubMed PMID: 13677828.

351. Basset D, Frapier JM, Dedet JP, Grolleau R. [Autochthonous cardiac hydatid cyst of favorable outcome]. Presse Med. 2000;29(21):1175. Epub 2000/07/25. PubMed PMID: 10906937.

352. Ben M'Rad S, Mathlouthi A, Merai S, Mestiri I, Ben Miled-Mrad K, Djenayah F. [Pulmonary embolism of hydatic origin]. Presse Med. 1998;27(5):205-7. Epub 1998/10/13. PubMed PMID: 9768011.

353. Bennani S, Ait Bolbarod A, el Mrini M, Benjelloun S. [Multiple bilateral renal hydatidosis. A case report]. Ann Urol (Paris). 1995;29(3):150-3. Epub 1995/01/01. PubMed PMID: 7486851.

354. Bennis A, Bennani-Smires C, Chraibi N. Imaging in Cardiac Echinococcosis. Echocardiography. 1997;14(5):455-8. Epub 2001/02/15. PubMed PMID: 11174981.

355. Bennis A, Bennani-Smires C, Chraibi N. Magnetic resonance imaging study of a cardiac hydatid cyst. Eur Heart J. 1997;18(3):525-6. Epub 1997/03/01. doi: 10.1093/oxfordjournals.eurheartj.a015277. PubMed PMID: 9076394.

356. Bennis A, Chraibi S, Noureddine M, Bennani-Smires C, Soulami S, Chraibi N. [Imaging in cardiac hydatid cyst. Apropos of a case]. Ann Cardiol Angeiol (Paris). 1996;45(3):132-5. Epub 1996/03/01. PubMed PMID: 8762916.

357. Bennis A, Darif A, Mehadji BE, Chraibi N. Cardiac hydatid cyst revealed by complete heart block. Heart Vessels. 2000;15(1):46-8. Epub 2000/09/23. PubMed PMID: 11001486.

358. Beshlyaga VM, Demyanchuk VB, Glagola MD, Lazorishinets VV. Echinococcus cyst of the left ventricle in 10-year-old patient. Eur J Cardiothorac Surg. 2002;21(1):87. Epub 2002/01/15. doi: 10.1016/s1010-7940(01)01073-9. PubMed PMID: 11788266.

359. Birincioglu CL, Bardakci H, Kucuker SA, Ulus AT, Arda K, Yamak B, et al. A clinical dilemma: cardiac and pericardiac echinococcosis. Ann Thorac Surg. 1999;68(4):1290-4. Epub 1999/10/30. doi: 10.1016/s0003-4975(99)00692-x. PubMed PMID: 10543495.

360. Birincioglu CL, Tarcan O, Bardakci H, Saritas A, Tasdemir O. Off-pump technique for the treatment of ventricular myocardial echinococcosis. Ann Thorac Surg. 2003;75(4):1232-7. Epub 2003/04/10. doi: 10.1016/s0003-4975(02)04709-4. PubMed PMID: 12683569.

361. Birincioglu CL, Tarcan O, Nisanoglu V, Bardakci H, Tasdemir O. Is it cardiac tumor or echinococcosis? Tex Heart Inst J. 2001;28(3):230-1. Epub 2001/10/27. PubMed PMID: 11678264; PubMed Central PMCID: PMCPMC101188.

362. Borner H, Demertzis S, Heisel A, Berg G, Schneider G, Schafers HJ. [Acute pericardial tamponade in cardiac echinococcosis]. Z Kardiol. 1999;88(12):1028-33. Epub 2000/02/02. PubMed PMID: 10654395.

363. Brechignac X, Durieu I, Perinetti M, Geriniere L, Richalet C, Vitol Durand D. [Hydatid cyst of the heart]. Presse Med. 1997;26(14):663-5. Epub 1997/04/26. PubMed PMID: 9180881.

364. Busacca G, Puzzo A, Fiamma G. [Cardiac echinococcosis. Report of a case]. Minerva Cardioangiol. 1998;46(1-2):35-8. Epub 1998/10/22. PubMed PMID: 9780620.

365. Caballero J, Arana R, Calle G, Caballero FJ, Berruezo A, de Zayas R, et al. [A hydatid cyst in the vena cava inferior and right atrium with venous flow obstruction and pulmonary dissemination]. Rev Esp Cardiol. 1999;52(4):281-4. Epub 1999/04/28. doi: 10.1016/s0300-8932(99)74914-6. PubMed PMID: 10217973.

366. Cakir O, Eren N, Kilinc N. Cardiac hydatic cyst causing cerebral emboli in a child. Pediatr Cardiol. 2002;23(5):555-6. Epub 2002/09/05. PubMed PMID: 12211204.

367. Ceviz M, Becit N, Kocak H. Infected cardiac hydatid cyst. Heart. 2001;86(5):E13. Epub 2001/10/17. doi: 10.1136/heart.86.5.e13. PubMed PMID: 11602564; PubMed Central PMCID: PMCPMC1729986.

368. Ceyran H, Tasdemir K, Tezcaner T, Asgun F, Karahan OI, Emirogullari ON, et al. A rare cause of peripheral arterial embolism: ruptured cardiac hydatid cyst. Vasa. 2002;31(2):129-31. Epub 2002/07/09. doi: 10.1024/0301-1526.31.2.129. PubMed PMID: 12099145.

369. Charet E, Roudaut R, Lafitte S, Laffort P, Madonna F, de Mascarel A. Echocardiographic demonstration of rupture of intraseptal hydatid cyst. J Am Soc Echocardiogr. 2000;13(10):955-8. Epub 2000/10/13. doi: 10.1067/mje.2000.106824. PubMed PMID: 11029723.

370. Chellaoui M, Bouhouch R, Akjouj M, Chat L, Achaabane F, Alami D, et al. [Pericardial hydatid disease: three case reports]. J Radiol. 2003;84(3):329-31. Epub 2003/05/09. PubMed PMID: 12736595.

371. Davlouros PA, Ikonomidis I, Frimas K, Manolis AS. Silent cardiac echinococcosis. Lancet Infect Dis. 2002;2(6):367. Epub 2002/07/30. PubMed PMID: 12144899.

372. De Nardo A, Monea P, Adornato EM. [Multiple intracardiac hydatid cysts with clinical picture simulating mitral stenosis and inferior myocardial necrosis]. G Ital Cardiol. 1999;29(10):1218-21. Epub 1999/11/05. PubMed PMID: 10546138.

373. De Paulis R, Seddio F, Colagrande L, Polisca P, Chiariello L. Cardiac echinococcosis causing coronary artery disease. Ann Thorac Surg. 1999;67(6):1791-3. Epub 1999/07/03. doi: 10.1016/s0003-4975(99)00341-0. PubMed PMID: 10391299.

374. Demirkol MO, Kurtoglu N, Keles C, Sismanoglu M, Turan F. Hydatid cyst of the interventricular septum: an unusual cause of a fixed myocardial perfusion defect. Clin Nucl Med. 2001;26(8):716-7. Epub 2001/07/14. doi: 10.1097/00003072-200108000-00016. PubMed PMID: 11452186.

375. Demirtas M, Usal A, San M, Birand A. Hydatid disease presenting as cardiac tamponade. A case history. Angiology. 1996;47(6):601-7. Epub 1996/06/01. doi: 10.1177/000331979604700609. PubMed PMID: 8678335.

376. Drira I, Fennira H, Hantous S, El Mokhtar E, Rekhis O, Hadoussa J, et al. [Hydatid pulmonary embolism. Two case reports]. Rev Pneumol Clin. 2000;56(1):41-4. Epub 2000/03/31. PubMed PMID: 10740114.

377. Ege E, Soysal O, Gulculer M, Ozdemir H, Pac M. Cardiac hydatid cyst causing massive pulmonary embolism. Thorac Cardiovasc Surg. 1997;45(5):249-50. Epub 1997/12/24. doi: 10.1055/s-2007-1013738. PubMed PMID: 9402667.

378. El Abbassi Skalli A, El Amraoui F, Chikhaoui N, Kadiri R. [Hydatid cyst of the mediastinum: 2 cases]. J Radiol. 2000;81(2):154-7. Epub 2000/03/08. PubMed PMID: 10705147.

379. El Fortia M, Bendaoud M, Maghur H, Ben Musa AA, Ettir A, Dirar A, et al. Intracavitary cardiac hydatid cyst and the wall sign criteria. Eur J Ultrasound. 1998;8(2):115-7. Epub 1998/12/10. PubMed PMID: 9845791.

380. el Quessar A, Benabdejlil M, Mansouri A, el Hassani MR, Chakir N, Jiddane M, et al. [Cerebral vascular accidents due to hydatid embolisms. Apropos of 2 cases]. J Neuroradiol. 1996;23(2):74-8. Epub 1996/09/01. PubMed PMID: 8991963.

381. Emirogullari N, Uzum K, Ustunbas HB, Andac H, Tasdemir K. Primary cardiac echinococcosis in childhood. Case report. Scand J Thorac Cardiovasc Surg. 1995;29(3):153-6. Epub 1995/01/01. PubMed PMID: 8614785.

382. Erenturk S, Kocazeybek B, Oner A, Sonmez B. Cardiac hydatid cyst in left ventricle. Acta Chir Belg. 1998;98(4):164-5. Epub 1998/10/21. PubMed PMID: 9779240.

383. Firatli II, Ozder A, Incesoy N, Turko, gbreve, lu C, et al. Asymptomatic Pericardial Hydatid Cyst. Int J Angiol. 1999;8(3):161-4. Epub 1999/11/30. PubMed PMID: 10387125.

384. Giorgadze, Nadareishivili A, Goziridze M, Zodelava E, Nachkepia M, Grigolia G, et al. Unusual recurrence of hydatid cysts of the heart: report of two cases and review of the clinical and surgical aspects of the disease. J Card Surg. 2000;15(3):223-8. Epub 2001/06/21. PubMed PMID: 11414609.

385. Gomez-Aldaravi Gutierrez R, Otero Coto E, Chorro Gasco FJ, Munoz Gil J, Losada Casares A, Lopez Merino V. [Cardiac cysts. A case of isolated cardiac hydatidosis]. Rev Esp Cardiol. 1999;52(5):355-8. Epub 1999/06/16. doi: 10.1016/s0300-8932(99)74928-6. PubMed PMID: 10368589.

386. Gulati G, Goyal NK, Kothari SS, Sharma S, Bisoi AK. Pericardial hydatid cyst. Indian Heart J. 2002;54(4):437-8. Epub 2002/12/05. PubMed PMID: 12462678.

387. Gurgun C, Nalbantgil S, Cinar CS. Two cases of cardiac cyst hydatid with right and left ventricular involvement. Int J Cardiol. 2001;78(2):193-5. Epub 2001/06/12. doi: 10.1016/s0167-5273(00)00467-8. PubMed PMID: 11398768.

388. Iglesias LF, Zabala y Morales M, Marcos G, Gonzalez Eguaras M, Vega J, Vaccari O, et al. [Pericarditis secondary to the rupture of a hydatid cyst]. Rev Esp Cardiol. 1999;52(2):135-8. Epub 1999/03/12. PubMed PMID: 10073097.

389. Jamil F, Nanda NC, Thakur AC, Malhotra S, Agrawal DI, Reddy VV, et al. Echocardiographic Detection of Intramyocardial Coronary Obstruction Produced by Pericardial Hydatid Cyst. Echocardiography. 1997;14(5):459-60. Epub 2001/02/15. PubMed PMID: 11174982.

390. Jeridi G, Boughzala E, Hajri S, Hediji A, Ammar H. [Complicated hydatid cyst of the right atrium simulating myxoma of the tricuspid valve]. Ann Cardiol Angeiol (Paris). 1997;46(3):159-62. Epub 1997/03/01. PubMed PMID: 9183397.

391. Johnstone MT, Notarianni M, Charlamb M, Rasmussen C, Quist W, Levitsky S. Images in cardiovascular medicine. Ventricular tachycardia: a complication of an intramyocardial echinococcal cyst. Circulation. 2000;102(1):123-5. Epub 2000/07/06. doi: 10.1161/01.cir.102.1.123. PubMed PMID: 10880425.

392. Kabbaj N, Chat L, Dafiri R, Imani F. [Rare cause of ischemic cerebrovascular infarct in a young patient: cardiac hydatic cyst]. J Radiol. 1998;79(1):53-6. Epub 1998/10/03. PubMed PMID: 9757222.

393. Kammoun S, Frikha I, Fourati K, Fendri S, Benyoussef S, Sahnoun Y, et al. Hydatid cyst of the heart located in the interventricular septum. Can J Cardiol. 2000;16(7):921-4. Epub 2000/08/10. PubMed PMID: 10934311.

394. Kanadasi M, Demirtas M, San M, Ozer C, Soyupak SK, Kisacikoglu B. Mobile right atrial hydatid cyst with multiorgan involvement. Catheter Cardiovasc Interv. 2000;49(2):204-7. Epub 2000/01/22. PubMed PMID: 10642775.

395. Kaplan M, Demirtas M, Cimen S, Ozler A. Cardiac hydatid cysts with intracavitary expansion. Ann Thorac Surg. 2001;71(5):1587-90. Epub 2001/06/01. doi: 10.1016/s0003-4975(01)02443-2. PubMed PMID: 11383804.

396. Kara G, Aras G, Kucuk ON. Radionuclide imaging of cardiac hydatid cyst. Clin Nucl Med. 2003;28(3):254-5. Epub 2003/02/20. doi: 10.1097/01.Rlu.0000053545.39171.11. PubMed PMID: 12592148.

397. Karadede A, Ulgen MS, Temamogullari AV, Toprak N. A complicated case of pericardial hydatid cyst manifesting as constrictive pericarditis. Can J Cardiol. 2000;16(5):673-6. Epub 2000/06/02. PubMed PMID: 10833546.

398. Kardaras F, Kardara D, Tselikos D, Tsoukas A, Exadactylos N, Anagnostopoulou M, et al. Fifteen year surveillance of echinococcal heart disease from a referral hospital in Greece. Eur Heart J. 1996;17(8):1265-70. Epub 1996/08/01. doi: 10.1093/oxfordjournals.eurheartj.a015045. PubMed PMID: 8869869.

399. Keil W, Pankratz H, Szabados A, Baur C. [Sudden death caused by an arterial hydatid embolism]. Dtsch Med Wochenschr. 1997;122(10):293-6. Epub 1997/03/07. doi: 10.1055/s-2008-1047611. PubMed PMID: 9102272.

400. Keles C, Sismanoglu M, Bozbuga N, Erdogan HB, Akinci E, Ipek G, et al. A cardiac hydatid cyst involving the basal interventricular septum causing biventricular outflow tract obstruction. Thorac Cardiovasc Surg. 2000;48(6):377-9. Epub 2001/01/06. PubMed PMID: 11145411.

401. Kemaloglu S, Ozkan U, Bukte Y, Acar M, Ceviz A. Growth rate of cerebral hydatid cyst, with a review of the literature. Childs Nerv Syst. 2001;17(12):743-5. Epub 2002/02/28. doi: 10.1007/s003810100498. PubMed PMID: 11862441.

402. Klodas E, Roger VL, Miller FA, Jr., Utz JP, Danielson GK, Edwards WD. Cardiac echinococcosis: case report of unusual echocardiographic appearance. Mayo Clin Proc. 1995;70(7):657-61. Epub 1995/07/01. doi: 10.4065/70.7.657. PubMed PMID: 7791388.

403. Kontopoulos AG, Avramides MJ, Athyros VG. Diagnosis, treatment, and long-term follow up of a patient with a hydatid cyst of the left ventricle. Br Heart J. 1994;72(6):592. Epub 1994/12/01. doi: 10.1136/hrt.72.6.592. PubMed PMID: 7857746; PubMed Central PMCID: PMCPMC1025652.

404. Kopp CW, Binder T, Grimm M, Merl O, Thalhammer F, Ullrich R, et al. Images in cardiovascular medicine. Left ventricular echinococcosis with peripheral embolization. Circulation. 2002;106(13):1741-2. Epub 2002/09/25. doi: 10.1161/01.cir.0000035274.48425.92. PubMed PMID: 12270872.

405. Kotoulas GK, Magoufis GL, Gouliamos AD, Athanassopoulou AK, Roussakis AC, Koulocheri DP, et al. Evaluation of hydatid disease of the heart with magnetic resonance imaging. Cardiovasc Intervent Radiol. 1996;19(3):187-9. Epub 1996/05/01. doi: 10.1007/bf02577618. PubMed PMID: 8661648.

406. Kudaiberdiev T, Djoshibaev S, Yankovskaya L, Djumanazarov A. Multiple hydatid cysts of epicardium and pericardium. Int J Cardiol. 2001;81(2-3):265-7. Epub 2001/12/18. doi: 10.1016/s0167-5273(01)00554-x. PubMed PMID: 11744146.

407. Kulan K, Tuncer C, Kulan C, Serce K, Goldeli O, Irhan S, et al. Hydatid cyst of the interventricular septum and contribution of magnetic resonance imaging. Acta Cardiol. 1995;50(6):477-81. Epub 1995/01/01. PubMed PMID: 8932568.

408. Kurtoglu N, Ermeydan C, Akdemir R, Basaran Y, Dindar I. Interventricular septal hydatid cyst. Echocardiography. 2000;17(7):693-5. Epub 2000/12/07. PubMed PMID: 11107208.

409. Kurugoglu S, Kizilkilic O, Ogut G, Mihmanli I, Akman C, Tanrikulu H. Primary cardiac hydatid disease: cross-sectional imaging features. South Med J. 2002;95(10):1140-4. Epub 2002/11/12. PubMed PMID: 12425497.

410. Kutay V, Ekim H, Yakut C. Infected myocardial hydatid cyst imitating left ventricular aneurysm. Cardiovasc Surg. 2003;11(3):239-41. Epub 2003/04/22. PubMed PMID: 12704337.

411. Laglera S, Garcia-Enguita MA, Martinez-Gutierrez F, Ortega JP, Gutierrez-Rodriguez A, Urieta A. A case of cardiac hydatidosis. Br J Anaesth. 1997;79(5):671-3. Epub 1998/01/10. doi: 10.1093/bja/79.5.671. PubMed PMID: 9422912.

412. Lahdhili H, Hachicha S, Ziadi M, Thameur H. Acute pulmonary embolism due to the rupture of a right ventricle hydatic cyst. Eur J Cardiothorac Surg. 2002;22(3):462-4. Epub 2002/09/03. doi: 10.1016/s1010-7940(02)00360-3. PubMed PMID: 12204747.

413. Lioulias AG, Kokotsakis JN, Foroulis CN, Skouteli ET. Images in cardiovascular medicine. Multiple cardiac hydatid cysts: consistency of echocardiographic and surgical findings. Tex Heart Inst J. 2002;29(3):226-7. Epub 2002/09/13. PubMed PMID: 12224733; PubMed Central PMCID: PMCPMC124769.

414. Lopez-Rios F, Perez-Barrios A, de Agustin PP. Primary cardiac hydatid cyst in a child. Cytologic diagnosis of a case. Acta Cytol. 1997;41(4 Suppl):1387-90. Epub 1997/07/01. PubMed PMID: 9990281.

415. Macedo AJ, Magalhaes MP, Tavares NJ, Bento L, Sampayo F, Lima M. Cardiac hydatid cyst in a child. Pediatr Cardiol. 1997;18(3):226-8. Epub 1997/05/01. doi: 10.1007/s002469900158. PubMed PMID: 9142717.

416. Maden O, Atak R, Yetgin E, Senen K, Aras D, Kutuk E. An unusual primary left ventricular hydatid cyst complicated by cardiac tamponade and mitral regurgitation--a case report. Angiology. 2002;53(4):487-91. Epub 2002/07/30. doi: 10.1177/000331970205300419. PubMed PMID: 12143959.

417. Maffeis GR, Petrucci O, Carandina R, Leme CA, Jr., Truffa M, Vieira R, et al. Cardiac echinococcosis. Circulation. 2000;101(11):1352-4. Epub 2000/03/22. doi: 10.1161/01.cir.101.11.1352. PubMed PMID: 10725298.

418. Malamou-Mitsi V, Pappa L, Vougiouklakis T, Peschos D, Kazakos N, Grekas G, et al. Sudden death due to an unrecognized cardiac hydatid cyst. J Forensic Sci. 2002;47(5):1062-4. Epub 2002/10/02. PubMed PMID: 12353547.

419. Mangano S, Carerj S, Micari A, Cerrito M, Di Rosa S, Grassi R. Echinococcosis of the heart: echocardiographic features in a child. Ital Heart J. 2003;4(5):354. Epub 2003/07/10. PubMed PMID: 12848096.

420. Manisali M, Ozaksoy D, Kovanlikaya I. The role of MR imaging in cardiac echinococcosis. AJR Am J Roentgenol. 1997;168(1):282-3. Epub 1997/01/01. doi: 10.2214/ajr.168.1.8976967. PubMed PMID: 8976967.

421. Marci M, Ajello A, Finazzo F, Violante F, Pizzuto A, Battaglia A, et al. Cardiac echinococcus complicated by ventricular tachycardia. Echocardiography. 2001;18(7):613-5. Epub 2001/12/12. PubMed PMID: 11737973.

422. Marci M, Ponari A, Finazzo F, Battaglia A. Echocardiographically diagnosed cardiac echinococcus complicated by embolic intraventricular thrombus. J Am Soc Echocardiogr. 1998;11(12):1158-60. Epub 1999/01/29. doi: 10.1016/s0894-7317(98)80013-8. PubMed PMID: 9923997.

423. Maroto LC, Carrascal Y, Lopez MJ, Forteza A, Perez A, Zavanella C. Hydatid cyst of the interventricular septum in a 3.5-year-old child. Ann Thorac Surg. 1998;66(6):2110-1. Epub 1999/02/04. doi: 10.1016/s0003-4975(98)01079-0. PubMed PMID: 9930510.

424. Mecozzi G, Verunelli F, Mariani MA, Zucchelli G, Grandjean JG. Interventricular hydatid cyst with atrioventricular block: a case report. Ital Heart J. 2003;4(5):347-9. Epub 2003/07/10. PubMed PMID: 12848094.

425. Moutiris JA, Mavrommatis P, Zambartas C, Henein M. Isolated cardiac echinococcosis in Cyprus. Int J Cardiol. 2000;75(1):99-101. Epub 2001/02/24. doi: 10.1016/s0167-5273(00)00265-5. PubMed PMID: 11203329.

426. Narayan R, Mathew JT, Shetty PK. Cardiac echinococcosis. J Assoc Physicians India. 2001;49:292. Epub 2001/02/28. PubMed PMID: 11225153.

427. Narin N, Mese T, Unal N, Pinarli S, Cangar S. Pericardial hydatid cyst with a fatal course. Acta Paediatr Jpn. 1996;38(1):61-2. Epub 1996/02/01. doi: 10.1111/j.1442-200x.1996.tb03437.x. PubMed PMID: 8992862.

428. Nour-Eddine M, Habbal R, Haddani J, Mehadji BZ, Chraibi N. [Hydatic disease of the heart presenting with pericardial effusion. A case report]. Arch Mal Coeur Vaiss. 2000;93(1):95-9. Epub 2001/03/03. PubMed PMID: 11227725.

429. Odev K, Acikgozoglu S, Gormus N, Aribas OK, Kiresi DA, Solak H. Pulmonary embolism due to cardiac hydatid disease: imaging findings of unusual complication of hydatid cyst. Eur Radiol. 2002;12(3):627-33. Epub 2002/03/01. doi: 10.1007/s003300100988. PubMed PMID: 11870478.

430. Oliviero U, Scordino F, Scherillo G, Tosone G, Orlando R, Fazio S. Myocardial ischemia caused by an hydatid cyst of the interventricular septum successfully treated with albendazole. Ital Heart J. 2000;1(6):431-4. Epub 2000/08/10. PubMed PMID: 10929745.

431. Onursal E, Elmaci TT, Tireli E, Dindar A, Atilgan D, Ozcan M. Surgical treatment of cardiac echinococcosis: report of eight cases. Surg Today. 2001;31(4):325-30. Epub 2001/04/26. doi: 10.1007/s005950170153. PubMed PMID: 11321342.

432. Ozates M, Sari I. A pericardial hydatid cyst extending to the left lobe of the liver: the diagnostic value of MRI. Heart Vessels. 2000;15(1):44-5. Epub 2000/09/23. PubMed PMID: 11001485.

433. Ozdemir M, Diker E, Aydogdu S, Goksel S. Complete heart block caused by cardiac echinococcosis and successfully treated with albendazole. Heart. 1997;77(1):84-5. Epub 1997/01/01. doi: 10.1136/hrt.77.1.84. PubMed PMID: 9038702; PubMed Central PMCID: PMCPMC484642.

434. Ozer N, Aytemir K, Kuru G, Atalar E, Ozer Y, Ovunc K, et al. Hydatid cyst of the heart as a rare cause of embolization: report of 5 cases and review of published reports. J Am Soc Echocardiogr. 2001;14(4):299-302. Epub 2001/04/05. doi: 10.1067/mje.2001.108474. PubMed PMID: 11287893.

435. Ozyazicioglu A, Kocak H, Ceviz M, Balci AY. Surgical treatment of echinococcal cysts of the heart: report of 3 cases. Asian Cardiovasc Thorac Ann. 2002;10(1):66-8. Epub 2002/06/25. doi: 10.1177/021849230201000118. PubMed PMID: 12079977.

436. Parisi F, Gagliardotto P, Zattera G, Pansini S, Di Summa M, Orzan F, et al. Complicated removal of a hydatid cyst of the interventricular septum. J Cardiovasc Surg (Torino). 1995;36(3):269-71. Epub 1995/06/01. PubMed PMID: 7629213.

437. Perez Martinez A, Velasco Bayon JM, Gutierrez-Larraya F, Urquia Marti L, Fernandez-Epifanio JL, Merino Batres G. [Cardiac hydatid cyst in a child]. Rev Esp Cardiol. 1999;52(8):625-7. Epub 1999/08/10. doi: 10.1016/s0300-8932(99)74980-8. PubMed PMID: 10439664.

438. Porcu A, Dessanti A, Feo CF, Ballore L, Scanu AM, Fancellu A, et al. Three cases of cardiac hydatidosis: diagnosis, surgical treatment, and complications. Int Surg. 2001;86(2):127-31. Epub 2002/03/29. PubMed PMID: 11918238.

439. Posacioglu H, Nalbantgil S, Ozbakkaloglu M, Halil H, Buket S. Cardiac hydatid cyst located in the interventricular septum. Ann Thorac Surg. 2002;74(6):2199. Epub 2003/03/20. doi: 10.1016/s0003-4975(02)03489-6. PubMed PMID: 12643429.

440. Rein R, Niggemann B, Runge M. [Echinococcosis of the heart]. Herz. 1996;21(3):192-7. Epub 1996/06/01. PubMed PMID: 8767863.

441. Richter D, Bosse A, Weber A, Farah I, Laczkovics A. [Echinococcus cyst of the right ventricle. Diagnosis and therapy of a rare disease picture]. Zentralbl Chir. 2001;126(1):65-7. Epub 2001/03/03. doi: 10.1055/s-2001-11723. PubMed PMID: 11227298.

442. Riebel T, Bassir C, Luck W, Maurer J, Schmitz L. [Sonographic course of systemic echinococcosis in a 10 year old girl (with cardiac, hepatic, renal and muscular involvement)]. Ultraschall Med. 1999;20(5):201-6. Epub 1999/12/14. doi: 10.1055/s-1999-8909. PubMed PMID: 10595389.

443. Sabah I, Yalcin F, Okay T. Rupture of a presumed hydatid cyst of the interventricular septum diagnosed by transoesophageal echocardiography. Heart. 1998;79(4):420-1. Epub 1998/06/09. doi: 10.1136/hrt.79.4.420. PubMed PMID: 9616357; PubMed Central PMCID: PMCPMC1728658.

444. Sagkan O, Kossus A, Demirag MK, Dursun Y, Bahadir H, Yazici M, et al. Paroxysmal ventricular tachycardia due to interventricular hydatid cyst. Echocardiography. 2002;19(8):683-5. Epub 2002/12/19. PubMed PMID: 12487638.

445. Sakarya ME, Etlik O, Sakarya N, Ozen S, Temizoz O, Evirgen O, et al. MR findings in cardiac hydatid cyst. Clin Imaging. 2002;26(3):170-2. Epub 2002/05/02. PubMed PMID: 11983468.

446. Salih OK, Celik SK, Topcuoglu MS, Kisacikoglu B, Tokcan A. Surgical treatment of hydatid cysts of the heart: a report of 3 cases and a review of the literature. Can J Surg. 1998;41(4):321-7. Epub 1998/08/26. PubMed PMID: 9711167; PubMed Central PMCID: PMCPMC3950090.

447. Sarkis A, Ashoush R, Alawi A, Haddad A, Jebara V, Checrallah E. [Hydatid cyst of the heart simulating coronary ischemia]. Ann Cardiol Angeiol (Paris). 2001;50(4):206-10. Epub 2003/01/31. PubMed PMID: 12555594.

448. Shalaby RI, Rajendran U, Majeed OA, Shuhaiber H. Polyvisceral echinococcosis with involvement of the heart and chest wall: follow-up and review of literature. Ann Thorac Cardiovasc Surg. 1999;5(4):248-53. Epub 1999/10/06. PubMed PMID: 10508951.

449. Simic O, Strathausen S, Attarbaschi M, Bolte J. [Echinococcal hydatid cyst in the right ventricle]. Dtsch Med Wochenschr. 1996;121(43):1325-8. Epub 1996/10/25. doi: 10.1055/s-2008-1043147. PubMed PMID: 8964213.

450. Sinci V, Ozdogan ME, Tunaoglu FS, Kula S, Aydin H, Ozdemir H, et al. Hydatid disease and massive cardiac involvement. Ann Thorac Cardiovasc Surg. 1999;5(5):336-9. Epub 1999/11/07. PubMed PMID: 10550721.

451. Singh NP, Arora SK, Gupta A, Anuradha S, Sridhara G, Agarwal SK, et al. Stroke: a rare presentation of cardiac hydatidosis. Neurol India. 2003;51(1):120-1. Epub 2003/07/17. PubMed PMID: 12865545.

452. Sinha PR, Jaipuria N, Avasthey P. Intracardiac hydatid cyst and sudden death in a child. Int J Cardiol. 1995;51(3):293-5. Epub 1995/10/01. doi: 10.1016/0167-5273(95)02408-o. PubMed PMID: 8586478.

453. Siwach SB, Katyal VK, Jagdish. Cardiac echinococcosis--a rare echocardiographic diagnosis. Heart. 1997;77(4):378-9. Epub 1997/04/01. doi: 10.1136/hrt.77.4.378. PubMed PMID: 9155623; PubMed Central PMCID: PMCPMC484738.

454. Siwach SB, Katyal VK, Jagdish. Intracardiac mass lesions: experience of 14 cases. Indian Heart J. 1999;51(4):414-7. Epub 1999/11/05. PubMed PMID: 10547940.

455. Snodgrass D, Blome S. Cardiac hydatid disease: report of two cases. Australas Radiol. 2002;46(2):194-6. Epub 2002/06/13. doi: 10.1046/j.1440-1673.2001.01036.x. PubMed PMID: 12060162.

456. Struillou L, Rabaud C, Bischoff N, Preiss MA, May T, Canton P. [Complications of cardiac hydatid cyst. 2 cases]. Presse Med. 1997;26(25):1192-4. Epub 1997/11/05. PubMed PMID: 9380610.

457. Tedy G, Maamari S, Khoury J, Heraoui E, Karam G, Haddad A, et al. [Pericardial hydatid cysts. Value of magnetic resonance imaging. Apropos of a clinical case]. Ann Cardiol Angeiol (Paris). 1995;44(6):280-3. Epub 1995/06/01. PubMed PMID: 7574359.

458. Tejada JG, Saavedra J, Molina L, Forteza A, Gomez C. Hydatid disease of the interventricular septum causing pericardial effusion. Ann Thorac Surg. 2001;71(6):2034-5; discussion 5-6. Epub 2001/06/28. doi: 10.1016/s0003-4975(00)02269-4. PubMed PMID: 11426797.

459. Telli HH, Durgut K, Temizhan A, Gormus N. Ruptured cardiac hydatid cyst masquerading as acute coronary syndrome: report of a case. Surg Today. 2001;31(10):908-11. Epub 2002/01/05. PubMed PMID: 11759888.

460. Toquero J, Castedo E, Oteo JF, Rubio A, Canas A. Hydatid cyst in the interventricular septum: a rare cause of dizziness? Tex Heart Inst J. 2000;27(4):414-5. Epub 2001/02/24. PubMed PMID: 11198320; PubMed Central PMCID: PMCPMC101116.

461. Trehan V, Shah P, Yusuf J, Mukhopadhyay S, Nair GM, Arora R. Thromboembolism: a rare complication of cardiac hydatidosis. Indian Heart J. 2002;54(2):199-201. Epub 2002/06/28. PubMed PMID: 12086387.

462. Tukek T, Demirel S, Atilgan D, Akkaya V, Onursal E. Transesophageal echocardiography in the treatment of right atrial hydatid cyst. Its impact on diagnosis and surgical management. J Cardiovasc Surg (Torino). 2000;41(4):651-2. Epub 2000/10/29. PubMed PMID: 11052304.

463. Turgut M, Benli K, Eryilmaz M. Secondary multiple intracranial hydatid cysts caused by intracerebral embolism of cardiac echinococcosis: an exceptional case of hydatidosis. Case report. J Neurosurg. 1997;86(4):714-8. Epub 1997/04/01. doi: 10.3171/jns.1997.86.4.0714. PubMed PMID: 9120638.

464. Turkvatan A, Yelgec NS, Calikoglu U, Olcer T. Primary left ventricular hydatid cyst in a child: case report. Can Assoc Radiol J. 2000;51(6):346-8. Epub 2001/01/13. PubMed PMID: 11155397.

465. Uddin MJ, Sanyal SC, Salama AL, Othman B, Haque E, Cherian G, et al. Surgical management of echinococcosis of the heart. J Cardiovasc Surg (Torino). 2000;41(4):571-4. Epub 2000/10/29. PubMed PMID: 11052285.

466. Ugur HC, Attar A, Bagdatoglu C, Erdogan A, Egemen N. Secondary multiple intracranial hydatid cysts caused by intracerebral embolism of cardiac echinococcosis. Acta Neurochir (Wien). 1998;140(8):833-4. Epub 1998/11/12. doi: 10.1007/s007010050186. PubMed PMID: 9810451.

467. Ulgen MS, Alan S, Karadede A, Aydinalp O, Toprak N. Cardiac hydatid cysts located in both the left ventricular apex and the intraventricular septum: case report. Heart Vessels. 2000;15(5):243-4. Epub 2001/09/19. PubMed PMID: 11560362.

468. Umesan CV, Kurian VM, Verghese S, Sivaraman A, Cherian KM. Hydatid cyst of the left ventricle of the heart. Indian J Med Microbiol. 2003;21(2):139-40. Epub 2007/07/24. PubMed PMID: 17643003.

469. Unlu Y, Ceviz M, Karaoglanoglu N, Becit N, Kocak H. Arterial embolism caused by a ruptured hydatid cyst in the heart: report of a case. Surg Today. 2002;32(11):989-91. Epub 2002/11/22. doi: 10.1007/s005950200198. PubMed PMID: 12444437.

470. Ustunsoy H, Akdemir I, Sivrikoz MC, Tahtaci N, Aksoy M, Tuncozgur B. Cardiac hydatid cyst: report of two cases. Heart Lung Circ. 2002;11(2):117-20. Epub 2005/12/15. doi: 10.1046/j.1443-9506.2001.00112.x. PubMed PMID: 16352080.

471. Ustunsoy H, Akdemir I, Tahtaci N. Images in cardiology: Cardiac hydatid cyst. Heart. 2000;84(5):464. Epub 2000/10/20. doi: 10.1136/heart.84.5.464. PubMed PMID: 11039997; PubMed Central PMCID: PMCPMC1729486.

472. Uysalel A, Aral A, Atalay S, Akalin H. Cardiac echinococcsis with multivisceral involvement. Pediatr Cardiol. 1996;17(4):268-70. Epub 1996/07/01. PubMed PMID: 8662054.

473. Uysalel A, Yazicioglu L, Aral A, Akalin H. A multivesicular cardiac hydatid cyst with hepatic involvement. Eur J Cardiothorac Surg. 1998;14(3):335-7. Epub 1998/10/07. doi: 10.1016/s1010-7940(98)00169-9. PubMed PMID: 9761448.

474. Vazan A, Awad J, Elami A, Rudis E, Gilon D, Singer JJ, et al. Hydatid cysts of the heart. Circulation. 1999;100(7):e42-4. Epub 1999/08/18. doi: 10.1161/01.cir.100.7.e42. PubMed PMID: 10449704.

475. Vicol C, Rupp G, Wagner T, Sumer C, Hopfner W, Struck E. [Surgical treatment of acute pericardial tamponade in an infestation of the heart by Echinococcus]. Dtsch Med Wochenschr. 1998;123(9):250-2. Epub 1998/04/03. doi: 10.1055/s-2007-1023945. PubMed PMID: 9524535.

476. Yadav BS, Garg N, Raj D. Hydatid cyst of heart presenting as ventricular tachycardia. J Assoc Physicians India. 1999;47(3):342-3. Epub 2000/09/22. PubMed PMID: 10999135.

477. Yalcin E, Dogru D, Ozcelik U, Kiper N, Gocmen A, Akhan O. Cardiac hydatid cyst and pulmonary hydatidosis in a child. Pediatr Infect Dis J. 2002;21(12):1178-80. Epub 2003/01/02. doi: 10.1097/00006454-200212000-00024. PubMed PMID: 12508795.

478. Zamani A, Aydemir Y, Gormus N, Odev K, Solak H. Cardiac hydatid cyst in a patient with pulmonary tuberculosis. Int J Tuberc Lung Dis. 2002;6(11):1033-4. Epub 2002/12/12. PubMed PMID: 12475153.

479. Akcakaya N, Soylemez Y, Cokugras H, Aytac A, Akalin F. A case of hydatid cyst with intramural cardiac localization. Scand J Infect Dis. 1994;26(6):765-66. Epub 1994/01/01. doi: 10.3109/00365549409008650. PubMed PMID: 7747105.

480. Akhtar MJ. Hydatid disease of the right ventricle and role of tomographic scanning in its diagnosis. Int J Cardiol. 1991;33(3):432-4. Epub 1991/12/01. doi: 10.1016/0167-5273(91)90075-z. PubMed PMID: 1761340.

481. Alcini E, Tattoni V, Gabrielli F, Arturi E. [Echocardiography in the diagnosis of the intracardiac mass]. Ann Ital Chir. 1987;59(1-6):21-30. Epub 1987/01/01. PubMed PMID: 3503612.

482. Alfonso F, Rey M, Balaguer J, Artiz V, Rabago G. Hydatid cyst of the right atrium diagnosed by echocardiography. Am J Cardiol. 1987;60(10):931-2. Epub 1987/10/01. doi: 10.1016/0002-9149(87)91057-5. PubMed PMID: 3661416.

483. Ambrosi P, Mesana T, Habib G, Boulain L, Lambert M, Simeoni JB, et al. [Right intra-atrial extension of hydatid cyst mimicking cardiac thrombosis. Apropos of a case]. Arch Mal Coeur Vaiss. 1992;85(6):909-12. Epub 1992/06/01. PubMed PMID: 1417411.

484. Ameli M, Mobarhan HA, Nouraii SS. Surgical treatment of hydatid cysts of the heart: report of six cases. J Thorac Cardiovasc Surg. 1989;98(5 Pt 2):892-901. Epub 1989/11/01. PubMed PMID: 2682022.

485. Antonelli G, Chiddo A, Bortone A, Iliceto S, Rizzon P. Hydatid cyst of the interventricular septum causing obstruction of the right ventricular outflow tract: cross-sectional echocardiographic, angiographic and pathological findings. Eur Heart J. 1986;7(12):1083-5. Epub 1986/12/01. doi: 10.1093/oxfordjournals.eurheartj.a062019. PubMed PMID: 3830203.

486. Bardi I, Ben Cheikh M, Fourati M, Mechmeche R, Abdelmoumen S, Bousnina A, et al. [Computerized tomography of hydatid cyst of the heart in children]. J Belge Radiol. 1987;70(3):223-7. Epub 1987/01/01. PubMed PMID: 3654574.

487. Bartolo M, Artignani PL, Di Folca A. [Femoral embolism caused by rupture of a cardiac hydatid cyst. Presentation of a case and review of the literature]. Angiologia. 1981;33(6):341-4. Epub 1981/11/01. PubMed PMID: 7325402.

488. Baud F, Gandjbakhch I, Pavie A, Marchand P, Cabrol C. [Surgical treatment of a hydatid cyst of the interventricular septum. Apropos of a case]. Ann Chir. 1983;37(7):569-71. Epub 1983/10/01. PubMed PMID: 6680828.

489. Bayezid O, Ocal A, Isik O, Okay T, Yakut C. A case of cardiac hydatid cyst localized on the interventricular septum and causing pulmonary emboli. J Cardiovasc Surg (Torino). 1991;32(3):324-6. Epub 1991/05/01. PubMed PMID: 2055928.

490. Bazelly B, Donzeau-Gouge GP, Vanetti A, Daumet P. [Metastatic pulmonary echinococcosis secondary to a primary cardiac lesion situated in the interventricular septum. One case successfully treated surgically (author's transl)]. J Chir (Paris). 1982;119(4):271-3. Epub 1982/04/01. PubMed PMID: 7085813.

491. Ben-Musa AA, Singh H, Shembesh AH, Chugh JC. Cardiac hydatid cyst in a child. Clin Pediatr (Phila). 1990;29(7):409-11. Epub 1990/07/01. doi: 10.1177/000992289002900710. PubMed PMID: 2376099.

492. Benomar A, Yahyaoui M, Birouk N, Vidailhet M, Chkili T. Middle cerebral artery occlusion due to hydatid cysts of myocardial and intraventricular cavity cardiac origin. Two cases. Stroke. 1994;25(4):886-8. Epub 1994/04/01. doi: 10.1161/01.str.25.4.886. PubMed PMID: 8160236.

493. Boujnan MR, Langar J, Rokbani L, Kchir MM, Charrad A. [Hydatid cyst of the heart. Diagnostic characteristics and course]. Tunis Med. 1990;68(3):221-3. Epub 1990/03/01. PubMed PMID: 2356582.

494. Buris L, Takacs P, Varga M. Sudden death caused by hydatid embolism. Z Rechtsmed. 1987;98(2):125-8. Epub 1987/01/01. PubMed PMID: 3604467.

495. Byard RW, Bourne AJ. Cardiac echinococcosis with fatal intracerebral embolism. Arch Dis Child. 1991;66(1):155-6. Epub 1991/01/01. doi: 10.1136/adc.66.1.155. PubMed PMID: 1994846; PubMed Central PMCID: PMCPMC1793215.

496. Cantoni S, Frola C, Gatto R, Loria F, Terzi MI, Vallebona A. Hydatid cyst of the interventricular septum of the heart: MR findings. AJR Am J Roentgenol. 1993;161(4):753-4. Epub 1993/10/01. doi: 10.2214/ajr.161.4.8372751. PubMed PMID: 8372751.

497. Capella G, Zolezzi F, Villani R, Veniani M, Negro R, Rovelli EG, et al. [Right cardiac echinococcosis with coronary compression. Description of a clinical case]. G Ital Cardiol. 1986;16(8):696-701. Epub 1986/08/01. PubMed PMID: 3792735.

498. Cherif O, Thameur H, Chaouch H, Ben Ayed M, Fourati M, Rokbani L. [Hydatid heart cyst rupture into the aorta and hydatid pulmonary embolism]. Tunis Med. 1990;68(8-9):543-6. Epub 1990/08/01. PubMed PMID: 2281542.

499. Contamin C, Aubert M, Denis B, Goulier A, Hadjian A, Barrie J. [Hydatid cyst of the left ventricle associated with polyvisceral hydatidosis (author's transl)]. Chirurgie. 1980;106(9):731-6. Epub 1980/11/01. PubMed PMID: 7225190.

500. de Almeida CA, Peteiro J, Gonzalez A, Alzamora P, Gomez J, de Buruaga JD. [Echocardiographic diagnosis of a hydatid cyst localized at the intraventricular septum]. Rev Port Cardiol. 1989;8(12):859-61. Epub 1989/12/01. PubMed PMID: 2631833.

501. De Castro S, Santilli M, Brandimarte C, Gualdi G, Migliau G. [Role of noninvasive diagnosis in cardiac echinococcosis: pulmonary embolization from right atrial localization]. Cardiologia. 1991;36(10):817-21. Epub 1991/10/01. PubMed PMID: 1799894.

502. De Cristofaro M, Baldi C, Petrone M, Vecchio A, Di Mauro M, Ravera B. [Echinococcal cyst of the right ventricle: diagnostic role of 2-dimensional echocardiography]. G Ital Cardiol. 1985;15(10):995-7. Epub 1985/10/01. PubMed PMID: 4092920.

503. De Martini M, Nador F, Binda A, Arpesani A, Odero A, Lotto A. Myocardial hydatid cyst ruptured into the pericardium: cross-sectional echocardiographic study and surgical treatment. Eur Heart J. 1988;9(7):819-24. Epub 1988/07/01. doi: 10.1093/eurheartj/9.7.819. PubMed PMID: 3169050.

504. Desnos M, Brochet E, Cristofini P, Cosnard G, Keddari M, Mostefai M, et al. Polyvisceral echinococcosis with cardiac involvement imaged by two-dimensional echocardiography, computed tomography and nuclear magnetic resonance imaging. Am J Cardiol. 1987;59(4):383-4. Epub 1987/02/01. doi: 10.1016/0002-9149(87)90828-9. PubMed PMID: 3812300.

505. Di Bello R. Cardiac echinococcosis. Late sudden death after surgical treatment. Chest. 1981;79(1):110-1. Epub 1981/01/01. doi: 10.1378/chest.79.1.110. PubMed PMID: 7449486.

506. Di Biasi P, Gullace G, Fundaro P, Corallo S, Santoli C. [Cardiac echinococcosis: 2 cases treated surgically]. G Ital Cardiol. 1989;19(4):345-9. Epub 1989/04/01. PubMed PMID: 2753278.

507. Dutka DP, Morris GK. Intracardiac thrombus formation in association with hydatid disease. Eur Heart J. 1990;11(12):1120-3. Epub 1990/12/01. doi: 10.1093/oxfordjournals.eurheartj.a059653. PubMed PMID: 2292261.

508. Elias G, Khoury J, Hatem J. [Hydatid cyst of the right ventricle]. Arch Mal Coeur Vaiss. 1991;84(6):865-7. Epub 1991/06/01. PubMed PMID: 1898222.

509. Elkouby A, Vaillant A, Comet B, Malmejac C, Houel J. [Cardiac hydatidosis. Review of recent literature and report of 15 cases]. Ann Chir. 1990;44(8):603-10. Epub 1990/01/01. PubMed PMID: 2270894.

510. Eren EE, Aykut S, Kayihan A, Aydogan H, Dagsali S. Echinococcal cyst of the interventricular septum with right ventricular protrusion. Tex Heart Inst J. 1989;16(4):292-5. Epub 1989/01/01. PubMed PMID: 15227384; PubMed Central PMCID: PMCPMC326542.

511. Ernst A, Cikes I, Radovanovic N. Two-dimensional echocardiographic study of a cardiac hydatid cyst. Am J Cardiol. 1983;52(10):1361-3. Epub 1983/12/01. doi: 10.1016/0002-9149(83)90607-0. PubMed PMID: 6650426.

512. Erol C, Candan I, Akalin H, Sonel A, Kervancioglu C. Cardiac hydatid cyst simulating tricuspid stenosis. Am J Cardiol. 1985;56(12):833-4. Epub 1985/11/01. doi: 10.1016/0002-9149(85)91164-6. PubMed PMID: 4061322.

513. Exadactylos NI, Kouskos GP, Tsoukas A. Echinococcal disease with a cardiac hydatid cyst masquerading as coronary heart disease. Int J Cardiol. 1994;43(1):105-6. Epub 1994/01/01. doi: 10.1016/0167-5273(94)90101-5. PubMed PMID: 8175212.

514. Garcia-Fernandez F, Lopez De Sa E, Gamallo C, Pavon C, Sotillo J. Massive tricuspid regurgitation caused by intramyocardial hydatid cyst. Am J Cardiol. 1986;57(13):1199-200. Epub 1986/05/01. doi: 10.1016/0002-9149(86)90703-4. PubMed PMID: 3706178.

515. Gavrilescu S, Gavrilescu M, Streian C, Ene V. Hydatid cyst of the heart. A report of five cases. Med Interne. 1979;17(3):235-9. Epub 1979/07/01. PubMed PMID: 504927.

516. Geiger AW, Konertz W, Hindricks G, Hachenberg T, Fahrenkamp A, Scheld HH. Echinococcal cyst of the interventricular septum: a rare cause of myocardial ischemia. Thorac Cardiovasc Surg. 1992;40(1):42-4. Epub 1992/02/01. doi: 10.1055/s-2007-1020109. PubMed PMID: 1631865.

517. Goksel S, Kural T, Ergin A, Cengel A, Ozer C, Kutuk E. Hydatid cyst of the interventricular septum. Diagnosis by cross-sectional echocardiography and computed tomography, treatment with mebendazole. Jpn Heart J. 1991;32(5):741-4. Epub 1991/09/01. doi: 10.1536/ihj.32.741. PubMed PMID: 1774836.

518. Gomez-Arnau J, Criado A, Burgos R, Horno R, Agosti J. Iatrogenic myocardial dysfunction after formalization of the heart. Cardiovasc Dis. 1981;8(4):558-61. Epub 1981/12/01. PubMed PMID: 15216184; PubMed Central PMCID: PMCPMC288003.

519. Gonzalez M, Artaza M, Ortigosa J, Burgos R, de Miguel JM, de Teresa E, et al. [Diagnosis of cardiac hydatid cysts by bidimensional echocardiography]. Rev Esp Cardiol. 1982;35(6):557-60. Epub 1982/01/01. PubMed PMID: 7184091.

520. Guillot B, Andre-Fouet X, Chuzel M, Bozio A, Clermont A, Termet H, et al. [Hydatid cyst of the heart diagnosed with bidimensional echography. Apropos of a case]. Arch Mal Coeur Vaiss. 1982;75(12):1431-5. Epub 1982/12/01. PubMed PMID: 6820265.

521. Gurlek A, Dagalp Z, Ozyurda U. A case of multiple pericardial hydatid cysts. Int J Cardiol. 1992;36(3):366-8. Epub 1992/09/01. doi: 10.1016/0167-5273(92)90310-y. PubMed PMID: 1428273.

522. Hafid F, Maiza E, Hammoudi D, Derdar T, Keddari M. [Hydatid cyst of the pericardium and diaphragm. Apropos of a case]. Pediatrie. 1989;44(4):331-4. Epub 1989/01/01. PubMed PMID: 2677977.

523. Hamani A, Kerma A, Zbir E, Khatouri A, Nazzi M. [Hydatid cyst of the heart. Contribution of two-dimensional echocardiography. Apropos of 2 cases surgically treated]. Arch Mal Coeur Vaiss. 1992;85(1):95-8. Epub 1992/01/01. PubMed PMID: 1550440.

524. Haouala H, Rahal N, M'Henni H, Ben Hamadi F, Mechmech R, Guediche M. [Hydatid cyst of the heart of multivesicle type. Apropos of a case]. Ann Cardiol Angeiol (Paris). 1989;38(2):83-6. Epub 1989/02/01. PubMed PMID: 2650609.

525. Hernigou A, Plainfosse MC, Merran S, Guermonprez JL. [Role of echography in the diagnosis of 5 hydatid cysts of the heart]. Ann Radiol (Paris). 1983;26(8):648-54. Epub 1983/12/01. PubMed PMID: 6666976.

526. Hindricks G, Bocker D, Konertz W, Bongartz G, Borggrefe M. Polycystic disease of the kidneys complicating the diagnosis of myocardial echinococcosis. Eur Heart J. 1993;14(1):141-2. Epub 1993/01/01. doi: 10.1093/eurheartj/14.1.141. PubMed PMID: 8432283.

527. Houel J, Malmejac C, Vaillant A, Pinot JJ. [Surgical aspects of cardiac hydatidosis]. Chirurgie. 1984;110(3):240-8. Epub 1984/01/01. PubMed PMID: 6468070.

528. Jit I, Sahni D, Datta BN. Multiple hydatid cysts involving the heart, pulmonary trunk and aorta. Indian Heart J. 1993;45(1):65-7. Epub 1993/01/01. PubMed PMID: 8365745.

529. Journet M, Gay J, Benoit P, Marcantoni JP, Fontaliran P, Bourmayan C, et al. [Syncope with a rare etiology, cardiac hydatidosis]. Ann Med Interne (Paris). 1982;133(7):478-82. Epub 1982/01/01. PubMed PMID: 7158895.

530. Kabbani SS, Jokhadar M, Sundouk A, Nabhani F, Baba B, Shafik AI. Surgical management of cardiac echinococcosis. Report of four cases. J Cardiovasc Surg (Torino). 1992;33(4):505-10. Epub 1992/07/01. PubMed PMID: 1527160.

531. Kilani T, Mechemeche R, Daoues A, Horchani H, Malmeljac C, Houel J. [Endocavitary rupture of heart hydatid cysts--5 cases]. Tunis Med. 1991;69(3):139-42. Epub 1991/03/01. PubMed PMID: 1841466.

532. Kostucki W, van Kuyk M, Cornil A. Changing echocardiographic features of a hydatid cyst of the heart. Br Heart J. 1985;54(2):224-5. Epub 1985/08/01. doi: 10.1136/hrt.54.2.224. PubMed PMID: 4015934; PubMed Central PMCID: PMCPMC481884.

533. Krober SM, Wehner HD, Kaiserling E. [Rupture of an echinococcus cyst in the heart septum as a cause of sudden heart death]. Pathologe. 1993;14(4):223-6. Epub 1993/07/01. PubMed PMID: 8367391.

534. Lanzoni AM, Barrios V, Moya JL, Epeldegui A, Celemin D, Lafuente C, et al. Dynamic left ventricular outflow obstruction caused by cardiac echinococcosis. Am Heart J. 1992;124(4):1083-5. Epub 1992/10/01. doi: 10.1016/0002-8703(92)90999-c. PubMed PMID: 1529887.

535. Limacher MC, McEntee CW, Attar M, Nelson JG, DeBakey ME, Quinones MA. Cardiac echinococcal cyst: diagnosis by two-dimensional echocardiography. J Am Coll Cardiol. 1983;2(3):574-7. Epub 1983/09/01. doi: 10.1016/s0735-1097(83)80287-3. PubMed PMID: 6875121.

536. Louzir B, Beji M, Marsit N, Essaim I, Zaquali RM, Daghfous J. [Bilateral and multiple round pulmonary opacities]. Rev Pneumol Clin. 1994;50(4):178-9. Epub 1994/01/01. PubMed PMID: 7724988.

537. Lunardi P, Missori P, Di Lorenzo N, Fortuna A. Cerebral hydatidosis in childhood: a retrospective survey with emphasis on long-term follow-up. Neurosurgery. 1991;29(4):515-7; discussion 7-8. Epub 1991/10/01. PubMed PMID: 1944831.

538. M'Raihi ML, Djemel A, M'Zah N, Mechmeche R, Zegaya M, Ben Ismail M. [Multiple pulmonary hydatidosis associated with intracardiac cyst. Apropos of a case]. Rev Pneumol Clin. 1989;45(2):78-80. Epub 1989/01/01. PubMed PMID: 2799224.

539. Malouf J, Saksouk FA, Alam S, Rizk GK, Dagher I. Hydatid cyst of the heart: diagnosis by two-dimensional echocardiography and computed tomography. Am Heart J. 1985;109(3 Pt 1):605-7. Epub 1985/03/01. doi: 10.1016/0002-8703(85)90576-9. PubMed PMID: 3976487.

540. Mancuso L, Bondi F, Marchi S, Iacona MA, Guarnera S, Patane L. Cardiac hydatid disease with clinical features resembling tricuspid stenosis. Am Heart J. 1987;113(5):1234-6. Epub 1987/05/01. doi: 10.1016/0002-8703(87)90943-4. PubMed PMID: 3578019.

541. Mandke JV, Sanzgiri VP. Hydatid cyst of the interatrial and interventricular septum of the heart. Chest. 1991;99(4):1020. Epub 1991/04/01. doi: 10.1378/chest.99.4.1020. PubMed PMID: 2009753.

542. Martini B, Villanova C, Scattolin G, Grasso F, Mobilia G, Toffoluti T, et al. [Cardiac echinococcosis. Description of a case with cysts localized in the free wall of the left ventricle]. Minerva Cardioangiol. 1989;37(4):205-8. Epub 1989/04/01. PubMed PMID: 2771089.

543. Mechmeche R, Ben Cheikh M, Bousnina A, Gharbi HA, Ben Ismail M. [Imaging in cardiac echinococcosis. Apropos of 21 cases. Codification of a strategy for its study]. Ann Radiol (Paris). 1985;28(5):373-80. Epub 1985/01/01. PubMed PMID: 4051434.

544. Miralles A, Bracamonte L, Pavie A, Bors V, Rabago G, Gandjbakhch I, et al. Cardiac echinococcosis. Surgical treatment and results. J Thorac Cardiovasc Surg. 1994;107(1):184-90. Epub 1994/01/01. PubMed PMID: 8283882.

545. Morillo Perena F, Senador Gomez G, Paravisini Parra J. [Pulmonary hydatid embolism of cardiac origin. 2 case reports]. Rev Esp Cardiol. 1981;34(3):241-4. Epub 1981/01/01. PubMed PMID: 7302342.

546. Mucientes F, Zechel J, Burotto S, de la Fuente H. Hydatid massive pulmonary embolism due to primary cardiac echinococcosis. Rev Latinoam Microbiol. 1985;27(2):93-5. Epub 1985/04/01. PubMed PMID: 4070845.

547. Munoz Gil J, Chorro FJ, Martinez-Leon J, Losada A, Carbonell C, Llopis R, et al. [Echocardiography in the diagnosis of a pericardial cyst. Presentation of a case]. Rev Esp Cardiol. 1985;38(4):298-9. Epub 1985/07/01. PubMed PMID: 4048623.

548. Noah MS, el Din Hawas N, Joharjy I, Abdel-Hafez M. Primary cardiac echinococcosis: report of two cases with review of the literature. Ann Trop Med Parasitol. 1988;82(1):67-73. Epub 1988/02/01. doi: 10.1080/00034983.1988.11812211. PubMed PMID: 3041930.

549. O'Connor LF, Tellez G, Montero CG, Nunez L, Figuera D. [Cardiac hydatidosis: apropos of 10 cases surgically treated]. Rev Esp Cardiol. 1988;41(2):97-102. Epub 1988/02/01. PubMed PMID: 3368621.

550. Oliver JM, Benito LP, Ferrufino O, Sotillo JF, Nunez L. Cardiac hydatid cyst diagnosed by two-dimensional echocardiography. Am Heart J. 1982;104(1):164-5. Epub 1982/07/01. doi: 10.1016/0002-8703(82)90659-7. PubMed PMID: 7090975.

551. Oliver JM, Sotillo JF, Dominguez FJ, Lopez de Sa E, Calvo L, Salvador A, et al. Two-dimensional echocardiographic features of echinococcosis of the heart and great blood vessels. Clinical and surgical implications. Circulation. 1988;78(2):327-37. Epub 1988/08/01. doi: 10.1161/01.cir.78.2.327. PubMed PMID: 3396169.

552. Ottino G, Villani M, De Paulis R, Trucco G, Viara A. Restoration of atrioventricular conduction after surgical removal of a hydatid cyst of the interventricular septum. J Thorac Cardiovasc Surg. 1987;93(1):144-7. Epub 1987/01/01. PubMed PMID: 3796025.

553. Pansard Y, De Brux JL, Cohen-Solal A, Steg G, Himbert D, Popoff A, et al. [Hydatid cyst of the right heart and post-embolic pulmonary hypertension]. Arch Mal Coeur Vaiss. 1987;80(5):667-9. Epub 1987/05/01. PubMed PMID: 3113387.

554. Papagna D, Aloe MA, Spagnolo S. [Cardiopulmonary echinococcosis: a report of a clinical case]. G Ital Cardiol. 1991;21(10):1129-33. Epub 1991/10/01. PubMed PMID: 1804751.

555. Papo I, Ginsberg E, Albreht M, Martinovic N, Sokolic J. Surgical treatment of cardiac echinococcosis: report of nine cases. Tex Heart Inst J. 1982;9(1):3-9. Epub 1982/03/01. PubMed PMID: 15226804; PubMed Central PMCID: PMCPMC341464.

556. Pasaoglu I, Dogan R, Hazan E, Oram A, Bozer AY. Right ventricular hydatid cyst causing recurrent pulmonary emboli. Eur J Cardiothorac Surg. 1992;6(3):161-3. Epub 1992/01/01. doi: 10.1016/1010-7940(92)90124-g. PubMed PMID: 1567632.

557. Pasaoglu I, Dogan R, Pasaoglu E, Tokgozoglu L. Surgical treatment of giant hydatid cyst of the left ventricle and diagnostic value of magnetic resonance imaging. Cardiovasc Surg. 1994;2(1):114-6. Epub 1994/02/01. PubMed PMID: 8049915.

558. Patrikar DM, Mitra KR, Bhutada VR. Cerebral hydatid disease. Australas Radiol. 1993;37(2):226-7. Epub 1993/05/01. doi: 10.1111/j.1440-1673.1993.tb00059.x. PubMed PMID: 8512521.

559. Perna GP, Greco A, Valle G, Carughi S, Giannatempo GM, Lucentini L. [Recurrent cardiac echinococcosis: a report of a case with multiple intrapericardial cysts]. Cardiologia. 1992;37(9):627-30. Epub 1992/09/01. PubMed PMID: 1292867.

560. Picchio E, Giovannini E, Siclari F, Lotti A. [Cardiac echinococcosis. Two-dimensional echocardiographic diagnosis (author's transl)]. G Ital Cardiol. 1981;11(9):1327-31. Epub 1981/01/01. PubMed PMID: 7327340.

561. Porcu A, Conchedda C, Chironi G, Niolu P, Carta A, Ermini RL, et al. [Cardiac hydatidosis]. Ann Ital Chir. 1994;65(6):649-54. Epub 1994/11/01. PubMed PMID: 7598318.

562. Porcu M, Meloni G, Farci G, Loi B. [Hydatid localization in the proximal tract of the right branch of the pulmonary artery. Description of a case]. G Ital Cardiol. 1988;18(5):398-9. Epub 1988/05/01. PubMed PMID: 3192046.

563. Rehka V. [Human tapeworm findings in Gottwaldov]. Angew Parasitol. 1981;22(1):13-20. Epub 1981/02/01. PubMed PMID: 7235287.

564. Remadi JP, al Habash O, Hage A, Daillet E, Fisch M, Maho V, et al. [Hydatid cyst of the interventricular septum. Apropos of a case]. Arch Mal Coeur Vaiss. 1994;87(3):409-13. Epub 1994/03/01. PubMed PMID: 7832631.

565. Rey M, Alfonso F, Torrecilla EG, McKenna WJ, Balaguer J, Alvarez L, et al. Diagnostic value of two-dimensional echocardiography in cardiac hydatid disease. Eur Heart J. 1991;12(12):1300-7. Epub 1991/12/01. doi: 10.1093/eurheartj/12.12.1300. PubMed PMID: 1778196.

566. Richard JM, Suarez G, Perez Lorente F, Martinez Trabanco I, Moris C, Cortina A. [Heart hydatidosis diagnoses with bidimensional echocardiography]. Rev Med Univ Navarra. 1989;33(4):199-200. Epub 1989/10/01. PubMed PMID: 2490184.

567. Rivera R, Delcan JL. Surgical treatment of coronary insufficiency produced by cardiac echinococcosis. Chest. 1980;78(6):849-52. Epub 1980/12/01. doi: 10.1378/chest.78.6.849. PubMed PMID: 6969644.

568. Rossouw GJ, Knott-Craig CJ, Erasmus PE. Cardiac echinococcosis: cyst removal in a beating heart. Ann Thorac Surg. 1992;53(2):328-9. Epub 1992/02/01. doi: 10.1016/0003-4975(92)91343-8. PubMed PMID: 1731678.

569. Russo G, Tamburino C, Cuscuna S, Arcidiacono G, Foti R, Grimaldi DR, et al. Cardiac hydatid cyst with clinical features resembling subaortic stenosis. Am Heart J. 1989;117(6):1385-7. Epub 1989/06/01. doi: 10.1016/0002-8703(89)90424-9. PubMed PMID: 2729066.

570. Samarrai AA, Hussain WM, al-Shaarbaf HH, Ismail MA. Cardiac echinococcosis: the diagnostic value of computed tomography scanning. J Cardiovasc Surg (Torino). 1990;31(4):518-24. Epub 1990/07/01. PubMed PMID: 2211808.

571. Shields DA. Multiple emboli in hydatid disease. Bmj. 1990;301(6745):213-4. Epub 1990/07/28. doi: 10.1136/bmj.301.6745.213. PubMed PMID: 2393730; PubMed Central PMCID: PMCPMC1663583.

572. Shukri RS, Melhem RE. Intracardiac hydatid cyst: concise communication. J Thorac Imaging. 1993;8(1):79-80. Epub 1993/01/01. PubMed PMID: 8418321.

573. Sirinelli A, Le Guludec D, Laine JF, Sebag C, Bourguignon M, Slama M, et al. Ventricular tachycardia revealing a hydatid cyst. Am Heart J. 1987;114(3):656-9. Epub 1987/09/01. doi: 10.1016/0002-8703(87)90769-1. PubMed PMID: 3630907.

574. Spinelli C, Gori L, Berti P, Pierallini S, Durno M, Angeletti CA, et al. [Cardiac echinococcosis. Case report and review of the literature]. Minerva Chir. 1993;48(12):695-7. Epub 1993/06/30. PubMed PMID: 8414114.

575. Tuzuner A, Kuterdem E, Bac B, Oygur N, Uluoglu O, Aker H. An uncommon case of hydatid cyst localized on the endocardium causing arterial embolism. Vasa. 1980;9(4):277-80. Epub 1980/01/01. PubMed PMID: 7467809.

576. Urbanyi B, Rieckmann C, Hellberg K, Krakau M, Liebau G, Mayer A, et al. Myocardial echinococcosis with perforation into the pericardium. J Cardiovasc Surg (Torino). 1991;32(4):534-8. Epub 1991/07/01. PubMed PMID: 1864885.

577. Van Nooten G, Deuvaert F, De Paepe J, Primo G. Surgical treatment of echinococcal cyst of the right heart. Acta Chir Belg. 1985;85(4):227-30. Epub 1985/07/01. PubMed PMID: 4050254.

578. Vanjak D, Moutaoufik M, Leroy O, Beuscart C, Billiau V, Chidiac C, et al. [Cardiac hydatidosis: contribution of magnetic resonance imaging. Report of a case]. Arch Mal Coeur Vaiss. 1990;83(11):1739-42. Epub 1990/10/01. PubMed PMID: 2122852.

579. Varela-Duran J, Riede UN. Cardiac echinococcosis with pulmonary embolism. Pathol Res Pract. 1980;170(1-3):252-7. Epub 1980/12/01. doi: 10.1016/s0344-0338(80)80171-3. PubMed PMID: 18788168.

580. von Sinner WN, Linjawi T, al watban JA. Mediastinal hydatid disease: report of three cases. Can Assoc Radiol J. 1990;41(2):79-82. Epub 1990/04/01. PubMed PMID: 2328426.

581. Yekeler I, Kocak H, Aydin NE, Basoglu A, Okur A, Senocak H, et al. A case of cardiac hydatid cyst localized in the lungs bilaterally and on anterior wall of right ventricle. Thorac Cardiovasc Surg. 1993;41(4):261-3. Epub 1993/08/01. doi: 10.1055/s-2007-1013868. PubMed PMID: 8211934.

582. al-Naaman Y, Iskenderian AS, al-Omeri MM, al-Naama MR, Barlow D. Successful emergency operation for hydatid disease of the left ventricle. J Cardiovasc Surg (Torino). 1973;14(5):564-8. Epub 1973/09/01. PubMed PMID: 4767450.

583. al-Naaman YD, al-Omeri MM. Cardio-pericardial echinococcosis causing myocardial insufficiency, rightsided heart failure and constrictive pericarditis. Report of four cases and review of the literature. J Cardiovasc Surg (Torino). 1970;11(4):303-9. Epub 1970/07/01. PubMed PMID: 4250308.

584. al-Naaman YD, Samarrai AA, al-Omeri MM. Hydatid disease of the heart. A report of four cases. J Cardiovasc Surg (Torino). 1973;14(1):95-101. Epub 1973/01/01. PubMed PMID: 4690983.

585. Antonovic J, Rosch J. Angiographic approach to study of human cardiac echinococcosis. Report of a case. Radiology. 1972;103(2):281-2. Epub 1972/05/01. doi: 10.1148/103.2.281. PubMed PMID: 5024547.

586. Athanasiou DJ. [On echinococcal cysts in the heart]. Munch Med Wochenschr. 1966;108(13):707-11. Epub 1966/04/01. PubMed PMID: 6013878.

587. Beltran de Heredia J, Ramos G, Areal FL. Echinococcus disease of the heart. Int Surg. 1973;58(3):198-201. Epub 1973/03/01. PubMed PMID: 4693139.

588. Ben-Ismail M, Fourati M, Bousnina A, Kafsi N. [Complete syncopal auriculoventricular block by a hydatid cyst of the intraventricular septum]. Ann Med Interne (Paris). 1978;129(8-9):539-42. Epub 1978/08/01. PubMed PMID: 718007.

589. Biffani G, Lotti A, D'Alessandro L. [A case of primary echinococcal cyst of the heart]. Boll Soc Ital Cardiol. 1975;20(2):129-31. Epub 1975/01/01. PubMed PMID: 1231902.

590. Bleifeld W, Effert S. [On the diagnosis of cystic formations in various body regions using the ultrasonic echo sounding method]. Dtsch Med Wochenschr. 1967;92(39):1741-50. Epub 1967/09/29. PubMed PMID: 6054335.

591. Bozer AY, Saylam A. An unusual case of pericardial hydatid cyst simulating aortic aneurysm. J Cardiovasc Surg (Torino). 1973;14(1):90-4. Epub 1973/01/01. PubMed PMID: 4690982.

592. Bumin O. Pericarditis constrictiva due to hydatid disease. Am J Surg. 1966;112(3):450-4. Epub 1966/09/01. doi: 10.1016/0002-9610(66)90221-2. PubMed PMID: 5917316.

593. Calamai G, Perna AM, Venturini A. Hydatid disease of the heart. Report of five cases and review of the literature. Thorax. 1974;29(4):451-8. Epub 1974/07/01. doi: 10.1136/thx.29.4.451. PubMed PMID: 4277513; PubMed Central PMCID: PMCPMC470179.

594. Campioni N, Dalla Torre L. [Cardiac echinococcosis]. Cardiol Prat. 1973;24(3):289-95. Epub 1973/07/01. PubMed PMID: 4779827.

595. Comakov M. [Sudden death in a case of cardiac echinococcosis]. Folia Med (Plovdiv). 1965;7(5):378-80. Epub 1965/01/01. PubMed PMID: 4957793.

596. Conte G, Lauro S, Lise M, Nava A, Casarotto D. [Echinococcal cyst with unusual localizations. Case reports]. Minerva Med. 1971;62(69):3227-36. Epub 1971/09/15. PubMed PMID: 5111909.

597. De los Arcos E, Madurga MP, Perez Leon J, Martinez JL, Urquia M. Hydatid cyst of interventricular septum causing left anterior hemiblock. Br Heart J. 1971;33(4):623-5. Epub 1971/07/01. doi: 10.1136/hrt.33.4.623. PubMed PMID: 5557504; PubMed Central PMCID: PMCPMC487223.

598. Deutsch V, Kreisler B, Padeh B, Pausner YM. Echinococcosis of the heart diagnosed by cardioangiography. Br J Radiol. 1969;42(499):540-3. Epub 1969/07/01. doi: 10.1259/0007-1285-42-499-540. PubMed PMID: 5788065.

599. Di Bello R, Cat JM, Schmidt VZ. Chronic hydatid pericarditis with effusion. J Thorac Cardiovasc Surg. 1968;55(6):859-63. Epub 1968/06/01. PubMed PMID: 5651660.

600. Di Bello R, Mantero ME, Dubra J, Sanjines A. Hydatid cyst of the left ventricle of the heart. Acute hydatid pericarditis. Am J Cardiol. 1967;19(4):603-6. Epub 1967/04/01. doi: 10.1016/0002-9149(67)90439-0. PubMed PMID: 4225515.

601. Di Bello R, Sadi I, Esteves S. Abnormal precordial pulsation in cardiac echinococcosis. J Thorac Cardiovasc Surg. 1967;53(3):366-70. Epub 1967/03/01. PubMed PMID: 6019150.

602. Di Carlo D, Astolfi D, Capestro F, Ferrini L. [Cardiac echinococcosis simulating myocardial necrosis in a 9 year-old boy. Diagnosis and surgical treatment (author's transl)]. G Ital Cardiol. 1978;8(3):323-8. Epub 1978/01/01. PubMed PMID: 640315.

603. DiBello R, Abo JC, Borges UL. Hydatid constrictive pericarditis. A new case and review of the literature. J Thorac Cardiovasc Surg. 1970;59(4):530-2. Epub 1970/04/01. PubMed PMID: 5439362.

604. Dibello R, Abo JC, Cortes R, Zubiaurre L, Folle LE, Urioste HA, et al. HYDATID CYST OF THE LEFT VENTRICLE OF THE HEART: MITRAL INSUFFICIENCY AFTER SURGICAL TREATMENT. J Thorac Cardiovasc Surg. 1965;49:854-9. Epub 1965/05/01. PubMed PMID: 14277507.

605. Dodek A. A Cardiac mass in a young woman. Chest. 1972;62(3):317-8. Epub 1972/09/01. doi: 10.1378/chest.62.3.317. PubMed PMID: 5056590.

606. Fabian MS, Feher I, Reok A. [Echinococcus cyst of the heart]. Fortschr Geb Rontgenstr Nuklearmed. 1970;113(4):429-33. Epub 1970/10/01. PubMed PMID: 4252214.

607. Farooki QZ, Adelman S, Green EW. Echocardiographic differentiation of a cystic and a solid tumor of the heart. Am J Cardiol. 1977;39(1):107-11. Epub 1977/01/01. doi: 10.1016/s0002-9149(77)80019-2. PubMed PMID: 831418.

608. Fawzy ME. Hydatid disease of the heart. Br Heart J. 1976;38(3):307-8. Epub 1976/03/01. doi: 10.1136/hrt.38.3.307. PubMed PMID: 1259847; PubMed Central PMCID: PMCPMC485839.

609. Feichter GE, Bucking H, Moll A. [Unusual echinococcus disease attacking the heart and brain (author's transl)]. MMW Munch Med Wochenschr. 1974;116(47):2073-6. Epub 1974/11/22. PubMed PMID: 4216813.

610. Ganau A, Andreoni G, Piga G, Cassisa L, Budroni M, Scalise S, et al. [Bidimensional echocardiography in the study of a myocardial and pericardial echinococcal cyst]. Boll Soc Ital Cardiol. 1978;23(1):202-8. Epub 1978/01/01. PubMed PMID: 754772.

611. Gilsanz V, Campo C, Cue R, Estella J, Estrada RV, Perez-oteiza C, et al. Recurrent pulmonary embolism due to hydatid disease of heart. Study of 3 cases, one with intermittent tricuspid valve obstruction (atrial pseudomyxoma). Br Heart J. 1977;39(5):553-8. Epub 1977/05/01. doi: 10.1136/hrt.39.5.553. PubMed PMID: 861098; PubMed Central PMCID: PMCPMC483274.

612. Godeau P, Herreman G, Marty JP, Bisseliche F, Parizot A, Guiraudon G, et al. [Hydatidosis involving the heart and liver simultaneously. A case report with successful surgery]. Coeur Med Interne. 1976;15(1):117-22. Epub 1976/01/01. PubMed PMID: 1000911.

613. Handjani AM, Farpour A, Mechanic K, Haghighat A, Dutz W. Cardiovascular echinococcosis. Am J Surg. 1969;117(5):667-70. Epub 1969/05/01. doi: 10.1016/0002-9610(69)90403-6. PubMed PMID: 5791037.

614. Heyat J, Mokhtari H, Hajaliloo J, Shakibi JG. Surgical treatment of echinococcal cyst of the heart. Report of a case and review of the world literature. J Thorac Cardiovasc Surg. 1971;61(5):755-64. Epub 1971/05/01. PubMed PMID: 4931085.

615. Houel, Dor V, Malmejac C, Paoli JM, Gosset C. [Subendocardial hydatid cyst of the left ventricle. Operation under extracorporeal circulation]. Mem Acad Chir (Paris). 1965;91(32):1026-31. Epub 1965/12/08. PubMed PMID: 5857977.

616. Iacoboni M, Tedeschi A, Testoni F. [Echinococcal cyst of the myocardium]. Boll Soc Ital Cardiol. 1974;19(1):107-12. Epub 1974/01/01. PubMed PMID: 4469779.

617. Imperati L, Perrotti E. [On a case of primary hydatidosis of the heart]. Osp Ital Chir. 1967;16(1):61-70. Epub 1967/01/01. PubMed PMID: 5596031.

618. Karageorgis B, Papanicolis I. Some remarks on two personal cases of cardiac echinococcosis. Dis Chest. 1967;51(2):199-204. Epub 1967/02/01. doi: 10.1378/chest.51.2.199. PubMed PMID: 6019124.

619. Konstantinov D. [Radiographic studies in pericardial cysts]. Folia Med (Plovdiv). 1970;12(1):46-50. Epub 1970/01/01. PubMed PMID: 4991559.

620. Lellouch A, Rulliere R. [Intramyocardial hydatid cysts (2 cases and review of the literature)]. Coeur Med Interne. 1976;15(4):599-606. Epub 1976/10/01. PubMed PMID: 1021338.

621. Loperfido F, Bellocci F, Bonomo L, Marano P. [Hydatid cyst of the interventricular septum: electro-vectorcardiographic and cineangiographic study]. Boll Soc Ital Cardiol. 1973;18(10):911-7. Epub 1973/01/01. PubMed PMID: 4807024.

622. Minetto E, Lavezzaro G, Dellepiane G. [Cardiac localizations of hydatidosis]. Cardiol Prat. 1966;17(5):459-71. Epub 1966/10/01. PubMed PMID: 5995553.

623. Murphy TE, Kean BH, Venturini A, Lillehei CW. Echinococcus cyst of the left ventricle. Report of a case with review of the pertinent literature. J Thorac Cardiovasc Surg. 1971;61(3):443-50. Epub 1971/03/01. PubMed PMID: 5545589.

624. Palant A, Deutsch V, Kishon Y, Lieberman Y, Yahini JH, Neufeld HN. Pulmonary hydatid embolization. Report on 2 operated cases and review of published reports. Br Heart J. 1976;38(10):1086-91. Epub 1976/10/01. doi: 10.1136/hrt.38.10.1086. PubMed PMID: 973883; PubMed Central PMCID: PMCPMC483136.

625. Papamichael E, Ikkos D, Milingos M, Yannacopoulos J. Echinococcosis of the heart. Chest. 1971;59(3):280-3. Epub 1971/03/01. doi: 10.1378/chest.59.3.280. PubMed PMID: 5101727.

626. Perez-Gomez F, Duran H, Tamames S, Perrote JL, Blanes A. Cardiac echinococcosis: clinical picture and complications. Br Heart J. 1973;35(12):1326-31. Epub 1973/12/01. doi: 10.1136/hrt.35.12.1326. PubMed PMID: 4759932; PubMed Central PMCID: PMCPMC458802.

627. Pinch LW, Sehat A. Constrictive pericarditis associated with echinococcus cyst. Chest. 1972;61(2):194-5. Epub 1972/02/01. doi: 10.1378/chest.61.2.194. PubMed PMID: 5058905.

628. Porte J, Touboul P, Delahaye JP, Cavallaro J, Clermont A, Mikaeloff P, et al. [Recurrent ventricular tachycardia due to hydatic cyst of the heart. Recovery after surgical resection. Report of one case]. Arch Mal Coeur Vaiss. 1975;68(8):893-8. Epub 1975/08/01. PubMed PMID: 812444.

629. Price HV. Hydatid disease of interatrial septum. Arch Dis Child. 1973;48(10):816-8. Epub 1973/10/01. doi: 10.1136/adc.48.10.816. PubMed PMID: 4749687; PubMed Central PMCID: PMCPMC1648563.

630. Romanoff H. Echinococcosis of the heart: report of three new cases. J Thorac Cardiovasc Surg. 1973;66(1):29-39. Epub 1973/07/01. PubMed PMID: 4715383.

631. Serradimigni A, Houel J, Trigano, Malmejac C, Bory M, Poggi L. [Hydatid cyst of the heart simulating a myocardial infarct]. Arch Mal Coeur Vaiss. 1969;62(8):1175-82. Epub 1969/08/01. PubMed PMID: 4983775.

632. Shakibi JG, Safavian MH, Azar H, Siassi B. Surgical treatment of echinococcal cyst of the heart. Report of two cases and review of the world literature. J Thorac Cardiovasc Surg. 1977;74(6):941-6. Epub 1977/12/01. PubMed PMID: 336985.

633. Soler-Soler J, Noguera L, Fito R. Recent multiple pulmonary nodules with progressive cardiomegaly. Chest. 1978;73(3):397-8. Epub 1978/03/01. doi: 10.1378/chest.73.3.397. PubMed PMID: 630937.

634. Urquia M, Perez Leon J, de los Arcos E, Madurga P. Surgical treatment of cardiac echinococcosis. Report of 3 cases. J Cardiovasc Surg (Torino). 1972;13(2):191-8. Epub 1972/03/01. PubMed PMID: 5034839.

635. Vaglio L, Guarini L. [On a case of echinococcosis of the heart]. Gazz Int Med Chir. 1965;69(19):1588-604. Epub 1965/10/15. PubMed PMID: 5852188.

636. Vara R, Thorbeck CV. [Echinococcal cysts of the heart. Report on 4 cases of heart echinococcosis]. Chirurg. 1974;45(4):190-2. Epub 1974/04/01. PubMed PMID: 4837349.

637. Vestri A, Nigri A, Massi L, Reale A. [Electrocardiographic picture of myocardial infarct during echinococcosis of the heart]. Boll Soc Ital Cardiol. 1972;17(9):752-4. Epub 1972/01/01. PubMed PMID: 4681377.

638. Viamonte M, Jr., LePage JR. Pitfalls in the radiologic evaluation of mediastinal abnormalities. Radiol Clin North Am. 1968;6(3):451-65. Epub 1968/12/01. PubMed PMID: 4235527.

639. Welew GP. [To the problem of primary echinococcosis of the heart with a contribution of 2 cases]. Zentralbl Allg Pathol. 1966;108(5):573-8. Epub 1966/03/10. PubMed PMID: 5300952.

640. Al-Hakkak SMM, Al-Faham FSM, Al-Awwady AN. Acute limb ischemia caused by ruptured cardiac hydatid cyst - A case report. Int J Surg Case Rep. 2019;55:18-22. Epub 20190114. doi: 10.1016/j.ijscr.2018.12.005. PubMed PMID: 30684813; PubMed Central PMCID: PMCPMC6351290.

641. Alis D, Turna O. Magnetic Resonance and Computed Tomography Findings of Isolated Hydatid Cyst of the Interventricular Septum. J Belg Soc Radiol. 2018;102(1):74. Epub 20181123. doi: 10.5334/jbsr.1670. PubMed PMID: 30515472; PubMed Central PMCID: PMCPMC6266963.

642. Altıntaş Taşlıçay C, Dervişoğlu E, Babaoglu A, Anık Y. Co-occurrence of cardiac hydatid cyst with cerebral embolisation and vascular hydatid cyst. J Paediatr Child Health. 2019;55(11):1391-4. Epub 20190613. doi: 10.1111/jpc.14540. PubMed PMID: 31192477.

643. Balani A, Kumar AD, Dey AK. Intramyocardial hydatid cyst: a case report. Eur Heart J Case Rep. 2018;2(2):yty053. Epub 20180604. doi: 10.1093/ehjcr/yty053. PubMed PMID: 31020132; PubMed Central PMCID: PMCPMC6177088.

644. Cai X, Cai H, Gan Q, Chang W, Yuan F, Luo W, et al. Case Report: Rare Presentation of Multivisceral Echinococcosis. Am J Trop Med Hyg. 2019;100(5):1204-7. doi: 10.4269/ajtmh.18-0673. PubMed PMID: 30860020; PubMed Central PMCID: PMCPMC6493931.

645. Çetin M, Karaman K, Özgökçe M, Geylan H, Yildizeli B. Hydatid cyst involvement of both pulmonary arteries in a 14-year-old girl. Paediatr Int Child Health. 2018;38(4):294-7. Epub 20171023. doi: 10.1080/20469047.2017.1389808. PubMed PMID: 29057706.

646. Firouzi A, Neshati Pir Borj M, Alizadeh Ghavidel A. Cardiac hydatid cyst: A rare presentation of echinococcal infection. J Cardiovasc Thorac Res. 2019;11(1):75-7. Epub 20190313. doi: 10.15171/jcvtr.2019.13. PubMed PMID: 31024677; PubMed Central PMCID: PMCPMC6477106.

647. Gupta Y, Priyadarshi M. Perioperative management of intramyocardial hydatid cyst with off-pump technique. Ann Card Anaesth. 2019;22(1):92-5. doi: 10.4103/aca.ACA_46_18. PubMed PMID: 30648688; PubMed Central PMCID: PMCPMC6350434.

648. Oner T, Korun O, Celebi A. A cardiac hydatid cyst mimicking a pericardial tumour in a paediatric case. Cardiol Young. 2019;29(2):244-6. Epub 20181204. doi: 10.1017/s1047951118002032. PubMed PMID: 30511599.

649. Orhan G, Bastopcu M, Aydemir B, Ersoz MS. Intracardiac and pulmonary artery hydatidosis causing thromboembolic pulmonary hypertension. Eur J Cardiothorac Surg. 2018;53(3):689-90. doi: 10.1093/ejcts/ezx330. PubMed PMID: 28958014.

650. Pant B, Ramesh A, Selvaraj R. Hydatid cyst of the interventricular septum - A rare cause of heart block. Indian Pacing Electrophysiol J. 2019;19(2):79-80. Epub 20190117. doi: 10.1016/j.ipej.2019.01.002. PubMed PMID: 30660635; PubMed Central PMCID: PMCPMC6450826.

651. Separovic Hanzevacki J, Gasparovic H, Reskovic Luksic V, Ostojic Z, Biocina B. Staged management of a giant cardiac hydatid cyst: a case report. BMC Infect Dis. 2018;18(1):694. Epub 20181227. doi: 10.1186/s12879-018-3599-2. PubMed PMID: 30587137; PubMed Central PMCID: PMCPMC6307286.

652. Singh SK, Singh V, Kumar S, Devenraj V, Bhandari M, Pandey AK. Right ventricular hydatid cyst presented as tachyarrhythmia. Asian Cardiovasc Thorac Ann. 2019;27(6):489-91. Epub 20181128. doi: 10.1177/0218492318817665. PubMed PMID: 30486662.

653. Su L, Yu J, Dai C, Liu Y, Peng L. Echinococcosis in left ventricle: a case report. Medicine (Baltimore). 2019;98(16):e15267. doi: 10.1097/md.0000000000015267. PubMed PMID: 31008970; PubMed Central PMCID: PMCPMC6494232.

654. Vural U, Aglar AA, Kayacioglu İ. Intracoronary Hydatid Cyst Resulted in Coronary Artery Disease in a Young Patient. Braz J Cardiovasc Surg. 2019;34(1):107-10. doi: 10.21470/1678-9741-2018-0033. PubMed PMID: 30810684; PubMed Central PMCID: PMCPMC6385834.

655. Abbas M, Shaghaleh MM, Sultan MN, Aljress A, Kashkash MF. A rare case of interventricular hydatid cyst in a 17-year-old male: A case report. Ann Med Surg (Lond). 2022;80:104058. Epub 20220625. doi: 10.1016/j.amsu.2022.104058. PubMed PMID: 36045850; PubMed Central PMCID: PMCPMC9422047.

656. Akrim Y, Babokh F, El Hakkouni A. Cardiac Echinococcosis With Hepatic Involvement in a Child: A Case Report. Cureus. 2022;14(10):e30390. Epub 20221017. doi: 10.7759/cureus.30390. PubMed PMID: 36407185; PubMed Central PMCID: PMCPMC9668324.

657. Al-Dairy A, Abo Kasem R. Surgical excision of a cardiac hydatid cyst from the right ventricle in a child. Clin Case Rep. 2021;9(8):e04714. Epub 20210825. doi: 10.1002/ccr3.4714. PubMed PMID: 34466264; PubMed Central PMCID: PMCPMC8385462.

658. Al-Hakkak SMM, Abed AN, Janabi AK, Ali MK, Naema AA, Mahdi AB. Acute common iliac arterial occlusion caused by ruptured primary cardiac hydrated cyst -A case report. Ann Med Surg (Lond). 2019;45:113-9. Epub 20190809. doi: 10.1016/j.amsu.2019.08.003. PubMed PMID: 31463047; PubMed Central PMCID: PMCPMC6706607.

659. Aljaber NN, Alshoabi SA, Qurashi AA, Daqqaq TS. Cardiac hydatid cyst in the right ventricle: An unusual case at a rare site. J Taibah Univ Med Sci. 2020;15(3):249-52. Epub 20200325. doi: 10.1016/j.jtumed.2020.02.004. PubMed PMID: 32647522; PubMed Central PMCID: PMCPMC7336018.

660. AlShamlan RA, Almousa AM, Al Saeed MJ, Al-Dera FH, Alobaydun MA. Cardiac Hydatid Cyst Successfully Managed with Albendazole: A Case Report. Cureus. 2019;11(12):e6405. Epub 20191217. doi: 10.7759/cureus.6405. PubMed PMID: 31970035; PubMed Central PMCID: PMCPMC6964963.

661. Alur N, Kikkeri MS, Marimuthu V, Khamitkar Shankar Rao S, Backer PH. Cardiac echinococcosis associated with severe biventricular dysfunction: diagnosis by multimodality imaging. Eur Heart J Cardiovasc Imaging. 2022;23(6):e268. doi: 10.1093/ehjci/jeac035. PubMed PMID: 35165708.

662. Ameen A, Hilal K, Shaikh A, Khan F, Fatimi S. Cardiac hydatid cyst presenting as ventricular arrhythmia: a case report. Egypt Heart J. 2021;73(1):105. Epub 20211207. doi: 10.1186/s43044-021-00231-z. PubMed PMID: 34874501; PubMed Central PMCID: PMCPMC8651847.

663. Arora N, Behera A, Naganur SH, Chhabra M, Singhal M, Dhibar DP. Hydatid disease and the heart. Qjm. 2021;114(5):342. doi: 10.1093/qjmed/hcab003. PubMed PMID: 33459794.

664. Assamti M, Rasras H, Ismaili N, Elouafi N. Right-Sided Cardiac Hydatid Cyst Complicated With Pulmonary Embolization: A Pediatric Case Report. Cureus. 2020;12(11):e11503. Epub 20201116. doi: 10.7759/cureus.11503. PubMed PMID: 33354448; PubMed Central PMCID: PMCPMC7744213.

665. Ay M, Ogul H, Kantarci M. Unusual Mimicker of Left Ventricular Aneurysm in Contrast Enhanced CT Imaging; Calcified Hydatid Cyst. Arch Bronconeumol (Engl Ed). 2020;56(10):669. Epub 20191024. doi: 10.1016/j.arbres.2019.08.006. PubMed PMID: 31668770.

666. Bahjat AS, Sharif Tahir AM, Mohammed AA. Hydatid cyst of the heart with mitral valve stenosis; Case report. Ann Med Surg (Lond). 2020;49:49-52. Epub 20191206. doi: 10.1016/j.amsu.2019.11.018. PubMed PMID: 31890197; PubMed Central PMCID: PMCPMC6926137.

667. Bajdechi M, Manolache D, Tudor A, Orghidan M, Gurghean A. Cardiac hydatid cysts in a young man: A case report and a literature review. Exp Ther Med. 2022;24(3):550. Epub 20220701. doi: 10.3892/etm.2022.11487. PubMed PMID: 35978922; PubMed Central PMCID: PMCPMC9366287.

668. Bakr L, Alnajar S. A huge right ventricular hydatid cyst related to tricuspid valve causing severe pulmonary hypertension. Clin Case Rep. 2021;9(4):1853-6. Epub 20201212. doi: 10.1002/ccr3.3636. PubMed PMID: 33936602; PubMed Central PMCID: PMCPMC8077309.

669. Baruah N, Saikia PP, Nath M. Off-pump excision of ventricular myocardial hydatid cyst: a case report and review of literature. Indian J Thorac Cardiovasc Surg. 2021;37(4):427-30. Epub 20210115. doi: 10.1007/s12055-020-01113-w. PubMed PMID: 34220025; PubMed Central PMCID: PMCPMC8218134.

670. Ben Abderrahim S, Turki E, Gammoudi B, Kort I, Hmila I. Sudden Death Due to Ruptured Cardiac Hydatid Cyst of the Right Ventricle. Am J Forensic Med Pathol. 2021;42(4):e64-e6. doi: 10.1097/paf.0000000000000707. PubMed PMID: 34510051.

671. Berarducci J, Armenta-Moreno JI, Garcia-Cardenas AM, Armendáriz-Ferrari JC, Espinola-Zavaleta N. Cardiac echinococcosis: a multimodality approach. Eur Heart J Case Rep. 2021;5(12):ytab475. Epub 20211125. doi: 10.1093/ehjcr/ytab475. PubMed PMID: 34888438; PubMed Central PMCID: PMCPMC8652102.

672. Biçer M, Kozan Ş, Altın HF, Aydemir NA. Surgical management of cardiac cystic echinococcosis in a paediatric patient: a case report. Interact Cardiovasc Thorac Surg. 2022;35(6). doi: 10.1093/icvts/ivac279. PubMed PMID: 36420984; PubMed Central PMCID: PMCPMC9987217.

673. Bougrine R, Aissaoui H, Elouafi N, Ismaili N. Incidental Asymptomatic Giant Hydatid Cyst of the Interventricular Septum Bulging Into the Right Ventricle. Cureus. 2021;13(2):e13532. Epub 20210224. doi: 10.7759/cureus.13532. PubMed PMID: 33786239; PubMed Central PMCID: PMCPMC7996477.

674. Cakir IM, Aslan S, Bekci T. Cardiac and hepatic hydatid cyst in a child with chest pain. Rev Soc Bras Med Trop. 2021;54:e0131 2021. Epub 20210428. doi: 10.1590/0037-8682-0131-2021. PubMed PMID: 33950125; PubMed Central PMCID: PMCPMC8083889.

675. Cakir O, Sade R, Alper F. Radiological imaging of pericardial hydatid cyst. Rev Soc Bras Med Trop. 2021;54. Epub 20210308. doi: 10.1590/0037-8682-0753-2020. PubMed PMID: 33681936; PubMed Central PMCID: PMCPMC8008908.

676. Çankaya BY, Çolak A. Cystic echinococcosis involving the cardiac interventricular septum. Rev Soc Bras Med Trop. 2021;54. Epub 20210308. doi: 10.1590/0037-8682-0499-2020. PubMed PMID: 33681920; PubMed Central PMCID: PMCPMC8008895.

677. Cheng Z, Fang T, Guo Y. Typical MRI of cardiac hydatid involvement: a case report. Acta Cardiol. 2021;76(3):324-5. Epub 20200923. doi: 10.1080/00015385.2020.1746497. PubMed PMID: 32964782.

678. de Gregorio C, Ferrazzo G, Ceresa F, De Donno BF, Patanè F. Dynamic right ventricular outflow tract obstruction by cardiac hydatic cysts: A multimodality imaging study. J Clin Ultrasound. 2021;49(7):690-2. Epub 20210226. doi: 10.1002/jcu.22993. PubMed PMID: 33634879.

679. de Matteis GM, Arcari L, Mustilli M, Fina P, Stingone AM, Preziosi P, et al. Multimodality Imaging for Diagnosis and Characterization of a Cardiac Hydatid Cyst. J Cardiovasc Echogr. 2020;30(2):119-20. Epub 20200817. doi: 10.4103/jcecho.jcecho_14_20. PubMed PMID: 33282653; PubMed Central PMCID: PMCPMC7706379.

680. De NV, Minh PN, Duyet LV, Bich NN, Son TN, Jung BK, et al. Two Human Cases of Echinococcus ortleppi Infection in the Lung and Heart in Vietnam. Korean J Parasitol. 2020;58(4):451-6. Epub 20200825. doi: 10.3347/kjp.2020.58.4.451. PubMed PMID: 32871639; PubMed Central PMCID: PMCPMC7462803.

681. Derbel B, Ziadi J, Besbes T, Dougaz W, Mleyhi S, Zairi I, et al. Intracardiac echinococcosis cyst mimicking a septal cardiac tumor with neurological symptoms. Int J Infect Dis. 2019;88:152-3. Epub 20190828. doi: 10.1016/j.ijid.2019.08.024. PubMed PMID: 31472236.

682. Dhahri R, Amri K, Slouma M, Metoui L, Gharsallah I, Chourabi C, et al. Cardiac and Multiple Vertebral Hydatid Cysts: An Unusual Association. J Clin Rheumatol. 2021;27(8s):S644-s6. doi: 10.1097/rhu.0000000000001661. PubMed PMID: 35073637.

683. Dind A, Harmer JA, Hansen PS, Harris B. Extensive pulmonary artery embolisation caused by cardiac hydatid cyst rupture. BMJ Case Rep. 2021;14(4). Epub 20210407. doi: 10.1136/bcr-2020-240521. PubMed PMID: 33827876; PubMed Central PMCID: PMCPMC8030676.

684. Dong Z, Yusup M, Lu Y, Tang B. Hydatid cyst of the heart as a rare cause of arrhythmia: A case report and review of published reports. HeartRhythm Case Rep. 2022;8(6):458-62. Epub 20220409. doi: 10.1016/j.hrcr.2022.04.004. PubMed PMID: 35774212; PubMed Central PMCID: PMCPMC9237349.

685. Durmaz A, Düzyol Ç, Gür S, İlkeli E, Omay O. Pediatric hydatid cyst with ventricular aneurysm and surgical treatment with dor procedure, case report. J Card Surg. 2022;37(12):5584-7. Epub 20221106. doi: 10.1111/jocs.17132. PubMed PMID: 36335619.

686. El Boussaadani B, Regragui H, Bouhdadi H, Wazaren H, Ajhoun I, Laaroussi M, et al. Primary cardiac hydatid cyst presenting with massive pericardial effusion: a case report. Egypt Heart J. 2020;72(1):51. Epub 20200817. doi: 10.1186/s43044-020-00085-x. PubMed PMID: 32804331; PubMed Central PMCID: PMCPMC7431496.

687. El Hadj Sidi C, Mgarrech I, Alimi F, Tarmiz A. Four pathways for hydatid pulmonary embolism. J Card Surg. 2020;35(8):1877-84. Epub 20200711. doi: 10.1111/jocs.14754. PubMed PMID: 32652654.

688. El Ouarradi A, Oualim S, Bensahi I, Elkouhen M, Abouloiafa I, Sabry M. Brain and Cardiac Concomitant Localization of the Hydatid Cyst. Case Rep Pediatr. 2020;2020:4829496. Epub 20200818. doi: 10.1155/2020/4829496. PubMed PMID: 32908763; PubMed Central PMCID: PMCPMC7450355.

689. Erdoğan KE, Uğuz E, Hıdıroğlu M, Erkılıç E, Güney MC, Şener E. Surgical treatment of hydatid cyst infiltrating into myocardium and causing mitral valve regurgitation. Turk Gogus Kalp Damar Cerrahisi Derg. 2019;27(3):395-7. Epub 20190614. doi: 10.5606/tgkdc.dergisi.2019.17526. PubMed PMID: 32082892; PubMed Central PMCID: PMCPMC7021430.

690. Foladi N, Farzam F, Shah Hoshang MM, Rastin MS, Aien MT. Incidental Left Ventricular Myocardial Hydatid Cyst - A case report. Radiol Case Rep. 2022;17(3):496-501. Epub 20211215. doi: 10.1016/j.radcr.2021.11.040. PubMed PMID: 34976252; PubMed Central PMCID: PMCPMC8685912.

691. Fortunato G, Battellini R, Marenchino R, Posatini R, Domenech A, Kotowicz V. How to remove multiple mediastinal and pericardial hydatid cysts. Multimed Man Cardiothorac Surg. 2019;2019. Epub 20190906. doi: 10.1510/mmcts.2019.026. PubMed PMID: 31497937.

692. Giri S, LeVine S, Watts MR. Ventricular Tachycardia and the Cystic Heart: A Case Report. J Emerg Med. 2020;58(6):e243-e6. Epub 20200416. doi: 10.1016/j.jemermed.2020.03.021. PubMed PMID: 32307217.

693. Girit S, Polatoğlu E, Şenol E, Ceyran H, Çoban Kökten Ş. Pericardial hydatid cyst and tuberculosis co-existence. Turk Gogus Kalp Damar Cerrahisi Derg. 2018;26(2):312-5. Epub 20180430. doi: 10.5606/tgkdc.dergisi.2018.14909. PubMed PMID: 32082754; PubMed Central PMCID: PMCPMC7024124.

694. Groves DW, Klion AD, Chen MY, Arai AE. Swiss cheese heart. Eur Heart J. 2018;39(3):255-6. doi: 10.1093/eurheartj/ehx626. PubMed PMID: 29236989; PubMed Central PMCID: PMCPMC5837325.

695. Guha A, Ranjan R, Saxena P, Mehta Y. A rare case of cardiac hydatid cyst. Ann Card Anaesth. 2021;24(4):470-2. doi: 10.4103/aca.ACA_42_20. PubMed PMID: 34747756; PubMed Central PMCID: PMCPMC8617402.

696. Gupta A, Mishra SC, Jaiswal S, Pande S. Intracardiac hydatid cyst located in right ventricular outflow tract: a rare site. Indian J Thorac Cardiovasc Surg. 2021;37(5):588-90. Epub 20210402. doi: 10.1007/s12055-021-01165-6. PubMed PMID: 34511771; PubMed Central PMCID: PMCPMC8387540.

697. Handran CB, Hurwitz Koweek LM, Mammarappallil JG. Case 274: Cardiac Echinococcus. Radiology. 2020;294(2):478-81. doi: 10.1148/radiol.2019171881. PubMed PMID: 31961783.

698. Harmouchi H, Kouache ME, Lakranbi M, Ouadnouni Y, Smahi M. Cardiac hydatid cyst interposed between the right atrium and right ventricle on the tricuspid valve. Asian Cardiovasc Thorac Ann. 2022;30(2):199-201. Epub 20210112. doi: 10.1177/0218492320988444. PubMed PMID: 33435692.

699. Ibrahim FF, Rubay D, Yi S, Barqawi Z, Abed AN. Surgical Management of Cardiac Hydatid Cyst and the Residual Intramural Ectocyst. Cureus. 2020;12(8):e9829. Epub 20200818. doi: 10.7759/cureus.9829. PubMed PMID: 32953338; PubMed Central PMCID: PMCPMC7495958.

700. Ijaz N, Hanif H, Rehman Z, Ali N, Iqbal T, Rehman IU. Hydatid Cyst In A Rare Site; The Left Ventricle. J Ayub Med Coll Abbottabad. 2022;34(2):381-4. doi: 10.55519/jamc-02-9222. PubMed PMID: 35576309.

701. İriz E, Yaylı S, Kula S. Cystic echinococcosis of the interventricular septum: a rare clinical presentation. Cardiol Young. 2020;30(10):1515-6. Epub 20200805. doi: 10.1017/s1047951120002383. PubMed PMID: 32753083.

702. İyigün T, Kyaruzi MM, Kutay V, Karakurt ST. Asymptomatic Huge Cardiac Hydatid Cyst Located in the Interventricular Septum. Braz J Cardiovasc Surg. 2020;35(2):235-8. Epub 20200401. doi: 10.21470/1678-9741-2018-0368. PubMed PMID: 32369307; PubMed Central PMCID: PMCPMC7199978.

703. Jain SK, Jha VK, Mani GK. Concurrent intrapericardial-pulmonary hydatidosis: an unusual multisystem echinococcosis. Indian J Thorac Cardiovasc Surg. 2021;37(4):438-41. Epub 20210104. doi: 10.1007/s12055-020-01109-6. PubMed PMID: 34220028; PubMed Central PMCID: PMCPMC8218120.

704. Jamli M, Cherif T, Ajmi N, Besbes T, Mgarrech I, Jerbi S, et al. Surgical Management and Outcomes of Cardiac and Great Vessels Echinococcosis: A 16-Year Experience. Ann Thorac Surg. 2020;110(4):1333-8. Epub 20200304. doi: 10.1016/j.athoracsur.2020.01.065. PubMed PMID: 32145201.

705. Joseph AG, Lahiri R, Joseph A, Sengupta G. Giant hydatid cyst of interventricular septum of heart. Indian J Thorac Cardiovasc Surg. 2020;36(1):81-4. Epub 20190910. doi: 10.1007/s12055-019-00867-2. PubMed PMID: 33061102; PubMed Central PMCID: PMCPMC7525870.

706. Jusabani AM, Kalambo CF, Jusabani M, Surani S. Myocardial Echinococcosis: Rare Manifestation of a Common Parasitic Disease in Northern Tanzania. Cureus. 2020;12(6):e8681. Epub 20200618. doi: 10.7759/cureus.8681. PubMed PMID: 32699681; PubMed Central PMCID: PMCPMC7370672.

707. Karami M, Sadatmadani SF, Kouhi H, Sadeghi B, Rostamiyan Z, Hashemzadeh M. A rare presentation of hydatid cyst, involvement of uncommon sites with sparing of typical locations. J Res Med Sci. 2021;26:1. Epub 20210128. doi: 10.4103/jrms.JRMS_127_20. PubMed PMID: 34084180; PubMed Central PMCID: PMCPMC8103959.

708. Karima T, Chenik S, Fehri W. A rare combination of cardiac and pulmonary cyst and review of the literature. IDCases. 2021;26:e01251. Epub 20210817. doi: 10.1016/j.idcr.2021.e01251. PubMed PMID: 34471602; PubMed Central PMCID: PMCPMC8390689.

709. Kaskar A, Shetty V, Shetty D. Chronic pulmonary thromboembolism due to intracardiac and pulmonary hydatidosis. Asian Cardiovasc Thorac Ann. 2020;28(9):610-2. Epub 20200909. doi: 10.1177/0218492320957918. PubMed PMID: 32903022.

710. Kasturi S, Kiritkumar M, Kumar C, Parachuri V. Surgical management of hydatid cyst of the interventricular septum. Multimed Man Cardiothorac Surg. 2021;2021. Epub 20210408. doi: 10.1510/mmcts.2021.019. PubMed PMID: 33844476.

711. Khalilian MR, Norouzi AR, Zamani H, Nia SKF, Teymoordash SN. A Rare Case of Cardiac Hydatid Disease without Liver and Lungs Involvement. Iran J Public Health. 2021;50(11):2332-6. doi: 10.18502/ijph.v50i11.7590. PubMed PMID: 35223609; PubMed Central PMCID: PMCPMC8826334.

712. Kort I, Hmandi O, Bekir O, Belhaj A, Jemail L, Allouche M, et al. Sudden death due to a massive hydatid pulmonary embolism secondary to a cardiac cyst rupture. J Forensic Sci. 2022;67(5):2101-5. Epub 20220610. doi: 10.1111/1556-4029.15075. PubMed PMID: 35686886.

713. Kuemmerli C, Sánchez-Velázquez P, Tschuor C, Oberkofler C, Lachat M, Müllhaupt B, et al. When Echinococcus granulosus transmigrates from the liver into the pericardium: a case report. J Surg Case Rep. 2021;2021(2):rjaa492. Epub 20210209. doi: 10.1093/jscr/rjaa492. PubMed PMID: 33598114; PubMed Central PMCID: PMCPMC7875092.

714. Kumar A, Ballal P, Nagamani AC, Sheriff SA. Surgical excision of an epicardial ventricular hydatid cyst. Asian Cardiovasc Thorac Ann. 2020;28(5):273-5. Epub 20200516. doi: 10.1177/0218492320927200. PubMed PMID: 32418436.

715. Lahdhili H, Lajmi M, Messaoudi H, Ragmoun W, Chenik S. One-stage surgery for both hepatic and left ventricular hydatid cysts using transthoracic route. Clin Case Rep. 2021;9(3):1115-7. Epub 20210121. doi: 10.1002/ccr3.3675. PubMed PMID: 33768793; PubMed Central PMCID: PMCPMC7981602.

716. Lahmidi I, Assoweh CD, Haddiya I, Bentata Y, Ouafi NE, Ismaili N. Clinicopathological features of adult right-sided cardiac masses: Analysis of 19 cases. Ann Med Surg (Lond). 2022;77:103613. Epub 20220410. doi: 10.1016/j.amsu.2022.103613. PubMed PMID: 35638074; PubMed Central PMCID: PMCPMC9142628.

717. Lahmidi I, Boutaybi M, El Ouazzani J, Elouafi N, Bazid Z. Isolated Cardiac Hydatid Cyst Causing Complete Heart Block. Cureus. 2020;12(12):e11945. Epub 20201207. doi: 10.7759/cureus.11945. PubMed PMID: 33425524; PubMed Central PMCID: PMCPMC7785471.

718. Liu L, Wu B, Li M, Guo Y. Case report: Right atrium-inferior vena cava bypass in a patient with unusual cardiac cystic echinococcosis. Front Cardiovasc Med. 2022;9:1001073. Epub 20221103. doi: 10.3389/fcvm.2022.1001073. PubMed PMID: 36407447; PubMed Central PMCID: PMCPMC9669341.

719. Lu YM, Zhang L, Xing Q, Zhou XH, Li YD, Zhang JH, et al. Ventricular tachycardia as the initial symptom of cardiac hydatidosis. Chin Med J (Engl). 2019;132(22):2765-6. doi: 10.1097/cm9.0000000000000520. PubMed PMID: 31725452; PubMed Central PMCID: PMCPMC6940097.

720. Lyazidi S, Abetti A, Abdellaoui A, El Adaoui A, Habbal R, Ettaoumi Y. Cardiac hydatid cyst in the right ventricle - A rare case report of echinococcosis presentation. Ann Med Surg (Lond). 2021;66:102427. Epub 20210525. doi: 10.1016/j.amsu.2021.102427. PubMed PMID: 34123377; PubMed Central PMCID: PMCPMC8175272.

721. Mahjoub Y, Boussaid M, Mesrati MA, Hadj MB, Limem H, Abdeljalil N, et al. Hydatid Disease, an Uncommon Etiology of Death in Forensic Practice. Am J Forensic Med Pathol. 2022;43(2):121-5. Epub 20220223. doi: 10.1097/paf.0000000000000750. PubMed PMID: 35213407.

722. Maliqari N, Teneqexhi L, Koja A, Veshti A, Baboci A, Haxhiu A, et al. Echinococcosis in both heart and lungs, the first case reported in Albania. Cardiol Young. 2021;31(11):1819-22. Epub 20210323. doi: 10.1017/s1047951121000998. PubMed PMID: 33752766.

723. Meimand SE, Sadeghpour A, Pakbaz M, Ghavidel AA, Pouraliakbar H, Kamali M, et al. Cardiac Echinococcosis Associated with Other Organ Involvement: Report of Two Challenging Cases. CASE (Phila). 2021;5(1):33-8. Epub 20201109. doi: 10.1016/j.case.2020.09.008. PubMed PMID: 33644511; PubMed Central PMCID: PMCPMC7887518.

724. Mesrati MA, Mahjoub Y, Ben Abdejlil N, Boussaid M, Belhaj M, Limem H, et al. Case Report: Sudden death related to unrecognized cardiac hydatid cyst. F1000Res. 2020;9:286. Epub 20200424. doi: 10.12688/f1000research.23277.3. PubMed PMID: 33500772; PubMed Central PMCID: PMCPMC7814283.

725. Mir H, McClure A, Thampinathan B, Chow C, Cusimano RJ, Bogoch, II, et al. Echocardiographic Features of Cardiac Echinococcal Infection. CASE (Phila). 2021;5(1):26-32. Epub 20201111. doi: 10.1016/j.case.2020.10.002. PubMed PMID: 33644510; PubMed Central PMCID: PMCPMC7887517.

726. Mohammad A, Sameer M, Vimala LR, Gnanamuthu BR, Benjamin SR, Shankar R. Pulmonary embolism from cardiac hydatids. Indian J Thorac Cardiovasc Surg. 2021;37(2):205-8. Epub 20201028. doi: 10.1007/s12055-020-01070-4. PubMed PMID: 33642720; PubMed Central PMCID: PMCPMC7876217.

727. Mutlu D, Raimoğlu U, Cimci M, Ömeroğlu SN, Durmaz E, İkitimur B, et al. Cardiac Hydatid Disease and Peritoneal Tuberculosis Coexistence. Turk Kardiyol Dern Ars. 2022;50(5):371-3. doi: 10.5543/tkda.2022.21244. PubMed PMID: 35860889.

728. Nasri S, Aichouni N, Lokman S, Ouafi NE, Kamaoui I, Skiker I. Cardiac hydatid cyst: 2 case reports. Radiol Case Rep. 2021;16(12):3829-33. Epub 20211010. doi: 10.1016/j.radcr.2021.09.013. PubMed PMID: 34659601; PubMed Central PMCID: PMCPMC8503848.

729. Nguyen HTT, Pham VT, Duong HD, Kirkpatrick JN, Taylor WR, Pham HM. Concomitant intramyocardial and hepatic hydatid cysts diagnosed by multi-modality imaging: A rare case report. Front Cardiovasc Med. 2022;9:1055000. Epub 20221214. doi: 10.3389/fcvm.2022.1055000. PubMed PMID: 36588570; PubMed Central PMCID: PMCPMC9795171.

730. Prasad K, Kumar R, Halder V, Raju M, Negi SL, Naganur S. Multimodality imaging of an interventricular septum hydatid cyst. Egypt Heart J. 2021;73(1):23. Epub 20210309. doi: 10.1186/s43044-021-00147-8. PubMed PMID: 33687569; PubMed Central PMCID: PMCPMC7943659.

731. Rhissassi J, Bouhdadi H, Wazaren H, El Aamadi W, Bouchikh M, Laaroussi M. Incidental finding of multiple pulmonary and cardiac hydatid cysts. Ann Cardiol Angeiol (Paris). 2021;70(2):122-4. Epub 20210223. doi: 10.1016/j.ancard.2021.01.004. PubMed PMID: 33637315.

732. Sarr SA, Aw F, Ndiaye M, Mingou J, Bodian M, Dioum M, et al. [Chronic Right Ventricular Failure Revealing a Large Compressive Hydatid Cyst at the Cardiology Department of the Aristide le Dantec Hospital (Dakar, Senegal)]. Bull Soc Pathol Exot. 2019;112(4):202-5. doi: 10.3166/bspe-2019-0090. PubMed PMID: 32003192.

733. Shakerian B, Mandegar MH. Huge Hydatid Cyst of the Right Ventricular Outflow Tract. Sultan Qaboos Univ Med J. 2021;21(3):485-7. Epub 20210829. doi: 10.18295/squmj.4.2021.017. PubMed PMID: 34522418; PubMed Central PMCID: PMCPMC8407898.

734. Singh A, Negi S, Kumar R, Toshkani D, Niyogi SG. Honeycomb in the heart: A rare case of hydatid cyst of the interventricular septum. J Clin Ultrasound. 2021;49(8):803-4. Epub 20210329. doi: 10.1002/jcu.22992. PubMed PMID: 33782966.

735. Singh S, Yadav MK. Extensive myocardial calcification mimicking giant honey-bee associated with cardiac hydatid cyst. Int J Cardiovasc Imaging. 2021;37(1):163-4. Epub 20200904. doi: 10.1007/s10554-020-01972-9. PubMed PMID: 32888106.

736. Sonsoz MR, Gunes SC. An intra-cardiac mass in a patient with Behçet's disease: Cardiac hydatid cyst. Echocardiography. 2020;37(4):646-8. Epub 20200316. doi: 10.1111/echo.14631. PubMed PMID: 32175642.

737. Tascanov M, Uğur M. Multiple hydatid cysts of the interventricular septum. Turk Gogus Kalp Damar Cerrahisi Derg. 2019;27(3):398-400. Epub 20190614. doi: 10.5606/tgkdc.dergisi.2019.17768. PubMed PMID: 32082893; PubMed Central PMCID: PMCPMC7021415.

738. Taşdemir A, Tuncay A, Karaman H, Canoz O, Aşık R, Özmen R, et al. Cardiac Masses: Pathological and Surgical Features - A Multicenter Study. Braz J Cardiovasc Surg. 2021;36(5):656-62. Epub 20211017. doi: 10.21470/1678-9741-2020-0225. PubMed PMID: 33355800; PubMed Central PMCID: PMCPMC8597599.

739. Vakilian F, Kamali A, Azari A, Poorzand H, Kamali A, Vakili Ahrari Roodi S. Isolated cardiac hydatid cyst presented as myopericarditis: A case report. J Cardiovasc Thorac Res. 2020;12(1):75-7. Epub 20200106. doi: 10.34172/jcvtr.2020.13. PubMed PMID: 32211143; PubMed Central PMCID: PMCPMC7080332.

740. Velho TR, Gonçalves JM, Pereira RM, da Cruz RM, Guerra NC, Ferreira R, et al. Primary cardiac hydatic cyst with multiple locations: an unusual case of chest pain. Gen Thorac Cardiovasc Surg. 2021;69(7):1147-50. Epub 20210412. doi: 10.1007/s11748-021-01631-3. PubMed PMID: 33846933.

741. Verma PK, Rohilla R, Natarajan V, Gupta PK. A rare case of coexisting tuberculosis with hydatid disease from North India with review of literature. BMJ Case Rep. 2020;13(9). Epub 20200907. doi: 10.1136/bcr-2020-235301. PubMed PMID: 32900726; PubMed Central PMCID: PMCPMC7478055.

742. Vlachakis PK, Dimitriadis K, Antonopoulos A, Tsioufis K. An 'uninvited guest' from the past: echinococcosis of the pericardium. Eur Heart J Case Rep. 2022;6(9):ytac372. Epub 20220907. doi: 10.1093/ehjcr/ytac372. PubMed PMID: 36131907; PubMed Central PMCID: PMCPMC9479878.

743. Wedin JO, Astudillo RM, Kurland S, Grinnemo KH, Astudillo R, Vikholm P, et al. A Rare Case of Cardiac Echinococcosis: The Role of Multimodality Imaging. CASE (Phila). 2021;5(4):230-4. Epub 20210428. doi: 10.1016/j.case.2021.03.002. PubMed PMID: 34430773; PubMed Central PMCID: PMCPMC8370852.

744. Wegner B, Meel R, Nell T, Nqwata L, Wong M. Hydatid disease of the interventricular septum: Echocardiographic and computed tomography findings. SA J Radiol. 2020;24(1):1986. Epub 20201215. doi: 10.4102/sajr.v24i1.1986. PubMed PMID: 33391841; PubMed Central PMCID: PMCPMC7756595.

745. Yimamu R, Qing-Qing L, Wei-Min Z. Primary pericardial hydatid cyst in an asymptomatic butcher. Cardiol Young. 2021;31(3):479-81. Epub 20201214. doi: 10.1017/s1047951120004114. PubMed PMID: 33308341.

746. Zghal FM, Boudiche S, Zongo T, Rekik B, Ouali S, Mourali MS. An unusual cause of stroke and sudden death: Intracavitary left ventricular echinococcosis. Int J Infect Dis. 2020;90:26-7. Epub 20191023. doi: 10.1016/j.ijid.2019.10.018. PubMed PMID: 31654817.

747. Zhang R, Shen C, Rao L. Cardiac echinococcosis secondary to hepatic echinococcosis: a rare case report. Cardiovasc Diagn Ther. 2022;12(1):147-52. doi: 10.21037/cdt-21-569. PubMed PMID: 35282668; PubMed Central PMCID: PMCPMC8898688.

748. Ozaydin I, Ozaydin C, Oksuz S, Yildirim M. Primary echinococcus cyst of the thyroid: a case report. Acta Med Iran. 2011;49(4):262-4. Epub 2011/06/30. PubMed PMID: 21713739.

749. Cemil B, Tun K, Gurcay AG, Uygur A, Kaptanoglu E. Cranial epidural hydatid cysts: clinical report and review of the literature. Acta Neurochir (Wien). 2009;151(6):659-62. Epub 2009/03/26. doi: 10.1007/s00701-009-0276-7. PubMed PMID: 19319472.

750. D'Alessandro A. Polycystic echinococcosis in tropical America: Echinococcus vogeli and E. oligarthrus. Acta Trop. 1997;67(1-2):43-65. Epub 1997/09/15. PubMed PMID: 9236939.

751. Kohansal MH, Nourian A, Rahimi MT, Daryani A, Spotin A, Ahmadpour E. Natural products applied against hydatid cyst protoscolices: A review of past to present. Acta Trop. 2017;176:385-94. Epub 2017/09/25. doi: 10.1016/j.actatropica.2017.09.013. PubMed PMID: 28935552.

752. Dibello R, Garciadarosa CM, Rubio R, Falconi LM, Urioste B. HYDATID CHRONIC COR PULMONALE. Am J Cardiol. 1965;15:708-14. Epub 1965/05/01. doi: 10.1016/0002-9149(65)90360-7. PubMed PMID: 14285155.

753. Rothlin MA. Fatal intraoperative pulmonary embolism from a hepatic hydatid cyst. Am J Gastroenterol. 1998;93(12):2606-7. Epub 1998/12/22. doi: 10.1111/j.1572-0241.1998.00562.x. PubMed PMID: 9860445.

754. Murray RH. Perispherical calcification at the site of old myocardial infarction. Am J Roentgenol Radium Ther Nucl Med. 1968;102(2):297-300. Epub 1968/02/01. doi: 10.2214/ajr.102.2.297. PubMed PMID: 5299883.

755. D'Alessandro A, Ramirez LE, Chapadeiro E, Lopes ER, de Mesquita PM. Second recorded case of human infection by Echinococcus oligarthrus. Am J Trop Med Hyg. 1995;52(1):29-33. Epub 1995/01/01. doi: 10.4269/ajtmh.1995.52.29. PubMed PMID: 7856823.

756. Manciulli T, Serraino R, D'Alessandro GL, Cattaneo L, Mariconti M, Vola A, et al. Evidence of Low Prevalence of Cystic Echinococcosis in the Catanzaro Province, Calabria Region, Italy. Am J Trop Med Hyg. 2020;103(5):1951-4. doi: 10.4269/ajtmh.20-0119. PubMed PMID: 32975181; PubMed Central PMCID: PMCPMC7646790.

757. Nakayama DK. The Surgical Operation that Led to the Declaration of Independence and the Bill of Rights. Am Surg. 2022;88(11):2609-11. Epub 20220503. doi: 10.1177/00031348221093528. PubMed PMID: 35506199.

758. Ghesquiere F, Mourot N, Olivero de Rubiana JP, Benichou A, Garen C, Chigot JP. [Surgery of hepatic hydatic cyst. Per-operative complications. Possibility of anaphylactic etiology (author's transl)]. Anesth Analg (Paris). 1979;36(11-12):561-3. Epub 1979/01/01. PubMed PMID: 95469.

759. Bajwa SJ, Panda A, Bajwa SK, Kaur J, Singh A. Anesthetic challenges in the simultaneous management of pulmonary and hepatic hydatid cyst. Anesth Essays Res. 2011;5(1):105-8. Epub 2011/01/01. doi: 10.4103/0259-1162.84189. PubMed PMID: 25885312; PubMed Central PMCID: PMCPMC4173378.

760. Kammoun S, Zayene M, Fendri S, Fourati S, Marouene M, Ben Youssef S, et al. [Chronic cor pulmonale caused by hydatid embolism complicating hepatic hydatid cyst]. Ann Cardiol Angeiol (Paris). 1997;46(5-6):317-20. Epub 1997/05/01. PubMed PMID: 9295892.

761. Ziadi A, Ejlaidi A, Hachimi A, Elkhayari A, Samkaoui MA. [Intraoperative cardiac arrest of a hepatic hydatid cyst surgery]. Ann Fr Anesth Reanim. 2014;33(3):195-6. Epub 2014/03/19. doi: 10.1016/j.annfar.2014.01.018. PubMed PMID: 24636789.

762. Mladenovic J, Videnovic N, Mladenovic K, Mladenovic S, Mladenovic R. Primary hydatid disease of the transverse abdominal muscle A case report. Ann Ital Chir. 2020;9. Epub 20200309. PubMed PMID: 32161184.

763. Sharif Tahir AM, Bahjat AS, Mohammed AA. Primary infected hydatid cyst of the thigh in a young lady; case report with literature review. Ann Med Surg (Lond). 2019;47:32-5. Epub 20190927. doi: 10.1016/j.amsu.2019.09.011. PubMed PMID: 31641500; PubMed Central PMCID: PMCPMC6796525.

764. Vecchio R, Vecchio V, Intagliata E. Transmission ways of Echinococcus granulosus in rare muscular locations of hydatid disease. Ann Med Surg (Lond). 2020;55:332-3. Epub 20200522. doi: 10.1016/j.amsu.2020.04.047. PubMed PMID: 32566219; PubMed Central PMCID: PMCPMC7296182.

765. Zakariaei Z, Fakhar M, Sharifpour A, Banimostafavi ES, Soleymani M, Zakariaei A. Anaphylactic shock due to ruptured pulmonary hydatid cyst in a young patient from Iran. Ann Med Surg (Lond). 2021;68:102675. Epub 20210806. doi: 10.1016/j.amsu.2021.102675. PubMed PMID: 34401139; PubMed Central PMCID: PMCPMC8358647.

766. Shalabi RI, Ayed AK, Amin M. 15 Years in surgical management of pulmonary hydatidosis. Ann Thorac Cardiovasc Surg. 2002;8(3):131-4. Epub 2002/12/11. PubMed PMID: 12472393.

767. Biglioli P, Spirito R, Roberto M, Parolari A, Agrifoglio M, Pompilio G, et al. False hydatic aneurysm of the thoracic aorta. Ann Thorac Surg. 1995;59(2):524-5. Epub 1995/02/01. doi: 10.1016/0003-4975(94)00574-q. PubMed PMID: 7847983.

768. Oguzkaya F, Akcali Y, Kahraman C, Emirogullari N, Bilgin M, Sahin A. Unusually located hydatid cysts: intrathoracic but extrapulmonary. Ann Thorac Surg. 1997;64(2):334-7. Epub 1997/08/01. doi: 10.1016/s0003-4975(97)00521-3. PubMed PMID: 9262570.

769. Tanveer F, Bhargava A. A Cyst Close to the Heart. Ann Thorac Surg. 2019;108(6):e413. Epub 20190726. doi: 10.1016/j.athoracsur.2019.05.084. PubMed PMID: 31356798.

770. Tong G, Lin X, Ma T, Wang X, Zhang W. Simultaneous Removal of Right Lung Hydatid Cyst and Repair of Atrial Septal Defect in a Single Session. Ann Thorac Surg. 2016;101(1):335-6. Epub 2015/12/24. doi: 10.1016/j.athoracsur.2015.01.074. PubMed PMID: 26694269.

771. Salem R, Zrig A, Joober S, Trimech T, Harzallah W, Jellali MA, et al. Pulmonary embolism in echinococcosis: two case reports and literature review. Ann Trop Med Parasitol. 2011;105(1):85-9. Epub 2011/02/08. doi: 10.1179/136485911x12899838413466. PubMed PMID: 21294952; PubMed Central PMCID: PMCPMC4089793.

772. Debi U, Bhatia V, Sandhu MS. Abdominal Aortic Occlusion by Hydatid Cysts. Aorta (Stamford). 2020;8(1):21-2. Epub 20200629. doi: 10.1055/s-0039-3401994. PubMed PMID: 32599630; PubMed Central PMCID: PMCPMC7324249.

773. Yuste P, Torres Carballada MA, Miguel Alonso JL. [Mechanism of electric alternance in pericardial effusion. Study with ultrasonics]. Arch Inst Cardiol Mex. 1975;45(2):197-202. Epub 1975/03/01. PubMed PMID: 1138658.

774. Norouzi R, Hejazy M, Azizi D, Ataei A. Effect of Taxus baccata L. Extract on Hydatid Cyst Protoscolices In vitro. Arch Razi Inst. 2021;75(4):473-80. Epub 20210101. doi: 10.22092/ari.2019.125573.1310. PubMed PMID: 33403842; PubMed Central PMCID: PMCPMC8410152.

775. Nanjappan S, RajagopalaReddy J, Velayutham V, Rajagopal S, DoraiRajan G. A rare cause of pericardial tamponade. Asian Cardiovasc Thorac Ann. 2008;16(4):348. Epub 2008/08/02. doi: 10.1177/021849230801600421. PubMed PMID: 18670035.

776. Omeroglu SN, Erdogan HB, Kirali K, Omeroglu A, Toker ME, Kayalar N, et al. Combined coronary artery bypass grafting and lung surgery. Asian Cardiovasc Thorac Ann. 2004;12(3):260-2. Epub 2004/09/09. doi: 10.1177/021849230401200318. PubMed PMID: 15353469.

777. Gemmell MA. Hydatidosis and cysticercosis. 2. Distribution of Cysticercus ovis in sheep. Aust Vet J. 1970;46(1):22-4. Epub 1970/01/01. doi: 10.1111/j.1751-0813.1970.tb14877.x. PubMed PMID: 5433299.

778. Sahpaz A, Irez A, Gulbeyaz H, Sener MT, Kok AN. Non-thrombotic Pulmonary Embolism Due to Liver Hydatic Cyst: A Case Report. Balkan Med J. 2017;34(3):275-7. Epub 2017/04/27. doi: 10.4274/balkanmedj.2016.0391. PubMed PMID: 28443563; PubMed Central PMCID: PMCPMC5450869.

779. Magistri P, Guerrini GP, Ballarin R, Assirati G, Tarantino G, Di Benedetto F. Improving Outcomes Defending Patient Safety: The Learning Journey in Robotic Liver Resections. Biomed Res Int. 2019;2019:1835085. Epub 2019/05/14. doi: 10.1155/2019/1835085. PubMed PMID: 31080809; PubMed Central PMCID: PMCPMC6476155.

780. Conde MP, Rodriguez MA, Lopez JM, Gonzalez-Porras JR. Thrombosis secondary to acute hypernatraemia after liver hydatid cyst surgery. Blood Coagul Fibrinolysis. 2015;26(6):695-8. Epub 2015/07/15. doi: 10.1097/mbc.0000000000000328. PubMed PMID: 26154611.

781. Kobryn K, Paluszkiewicz R, Dudek K, Oldakowska-Jedynak U, Korba M, Raszeja-Wyszomirska J, et al. Good outcome following liver transplantation using pericardial-peritoneum window for hepato-atrial anastomosis to overcome advanced hepatic alveolar echinococcosis and secondary Budd-Chiari Syndrome - a case report. BMC Surg. 2017;17(1):5. Epub 2017/01/15. doi: 10.1186/s12893-017-0205-2. PubMed PMID: 28086841; PubMed Central PMCID: PMCPMC5237181.

782. Hiremath B, Subramaniam N, Boggavarapu M. Primary pancreatic hydatid cyst: an unexpected differential diagnosis. BMJ Case Rep. 2015;2015. Epub 2015/09/04. doi: 10.1136/bcr-2015-211377. PubMed PMID: 26336187; PubMed Central PMCID: PMCPMC4567774.

783. Juodeikis Z, Poskus T, Seinin D, Strupas K. Echinococcus multilocularis infection of the liver presenting as abdominal wall fistula. BMJ Case Rep. 2014;2014. Epub 2014/05/09. doi: 10.1136/bcr-2014-203769. PubMed PMID: 24810453; PubMed Central PMCID: PMCPMC3992557.

784. Kunal S, Pilaniya V, Shah A. Middle lobe syndrome: a singularly rare presentation of complicated pulmonary hydatid disease. BMJ Case Rep. 2016;2016. Epub 2016/04/06. doi: 10.1136/bcr-2016-214670. PubMed PMID: 27045051; PubMed Central PMCID: PMCPMC4840639.

785. Patel J, Ali M. Hydatid cyst: hepatitis B and D coinfection accelerating the course of the disease. BMJ Case Rep. 2017;2017. Epub 2017/03/08. doi: 10.1136/bcr-2017-219569. PubMed PMID: 28264808; PubMed Central PMCID: PMCPMC5353532.

786. Saeed MY, Ahmed AH, Elhassan NB, Elhassan AM. Concomitant tuberculosis and hydatid cyst in a solitary pulmonary nodule of left lower lobe. BMJ Case Rep. 2009;2009. Epub 2009/01/01. doi: 10.1136/bcr.04.2009.1738. PubMed PMID: 21897836; PubMed Central PMCID: PMCPMC3027308.

787. Ciociola G, Cafaro L, Campanale G, Scarcia A, Scattaglia VF, Saliani R. [Echocardiographic findings in 2 cases of extracardiac mass]. Boll Soc Ital Cardiol. 1980;25(4):421-30. Epub 1980/01/01. PubMed PMID: 7284146.

788. Eren B, Turkmen N, Fedakar R. Allergic myocardial infarction due to hydatid cyst: an autopsy case. Bratisl Lek Listy. 2010;111(6):351-2. Epub 2010/07/20. PubMed PMID: 20635682.

789. Kankilic N, Aydin MS, Günendi T, Göz M. Unusual Hydatid Cysts: Cardiac and Pelvic-Ilio femoral Hydatid Cyst Case Reports and Literature Review. Braz J Cardiovasc Surg. 2020;35(4):565-72. Epub 20200801. doi: 10.21470/1678-9741-2019-0153. PubMed PMID: 32865381; PubMed Central PMCID: PMCPMC7454634.

790. Ozarmagan S, Erbil Y, Barbaros U, Salmaslioglu A, Bozbora A. Primary hydatid disease in the adrenal gland: a case report. Braz J Infect Dis. 2006;10(5):362-3. Epub 2007/02/13. PubMed PMID: 17293927.

791. Salem CB, Schneegans F, Chollet JY. [Study of lesional aspects of hydatid echinococcosis in man in Mauritania: fertility, histology of hydatid cysts and protoscolex viability]. Bull Soc Pathol Exot. 2011;104(1):1-5. Epub 2010/12/29. doi: 10.1007/s13149-010-0099-z. PubMed PMID: 21188568.

792. Aldahmashi M, Alassal M, Kasb I, Elrakhawy H. Conservative Surgical Management for Pulmonary Hydatid Cyst: Analysis and Outcome of 148 Cases. Can Respir J. 2016;2016:8473070. Epub 2016/09/20. doi: 10.1155/2016/8473070. PubMed PMID: 27642249; PubMed Central PMCID: PMCPMC5013219.

793. Mezgar Z, Khrouf M, Ben Soltane H, Mahjoub M, Ben Fredj S, Amara A, et al. Case of Massive Hydatid Pulmonary Embolism Incidentally Discovered in a 56-Year-Old Woman with Posttraumatic Abdominal Pain. Case Rep Pulmonol. 2018;2018:7831910. Epub 2018/06/05. doi: 10.1155/2018/7831910. PubMed PMID: 29862109; PubMed Central PMCID: PMCPMC5971327.

794. Mrabet FZ, Achrane J, Sabri Y, El Hassani FE, Hammi S, Bourkadi JE. Contribution of Imaging in Diagnosis of Primitive Cyst Hydatid in Unusual Localization: Pleura-A Report of Two Cases. Case Rep Radiol. 2018;2018:6242379. Epub 2018/08/17. doi: 10.1155/2018/6242379. PubMed PMID: 30112245; PubMed Central PMCID: PMCPMC6077536.

795. Patel AM, Trastek VF, Coles DT. Gossypibomas mimicking echinococcal cyst disease of the lung. Chest. 1994;105(1):284-5. Epub 1994/01/01. doi: 10.1378/chest.105.1.284. PubMed PMID: 8275748.

796. Chen ZY, Yan LN, Zeng Y, Wen TF, Li B, Zhao JC, et al. Transdiaphragmatic exposure for direct atrioatrial anastomosis in liver transplantation. Chin Med J (Engl). 2010;123(24):3515-8. Epub 2011/12/15. PubMed PMID: 22166622.

797. Savulescu F, Iordache, II, Hristea R, Dumitru C, Sandru AM, Balasa G, et al. Primary hydatid cyst with an unusual location--a case report. Chirurgia (Bucur). 2010;105(3):419-22. Epub 2010/08/24. PubMed PMID: 20726313.

798. Robles P, Rubio JA, Jimenez JJ. Right atrial and ventricular compression by a hepatic hydatic cyst. Clin Cardiol. 2009;32(6):E99. Epub 2007/07/10. doi: 10.1002/clc.20008. PubMed PMID: 17618474; PubMed Central PMCID: PMCPMC6653680.

799. Ozkan HS, Sahin B. Primary hydatid disease of subcutaneous tissue in the leg. Clin Exp Dermatol. 2010;35(8):915-6. Epub 2010/05/12. doi: 10.1111/j.1365-2230.2010.03843.x. PubMed PMID: 20456405.

800. Zippi M, Siliquini F, Fierro A, Aloisio P, Corbi S, Scocchera F, et al. Diffuse abdominal hydatidosis: role of magnetic resonance imaging. Clin Ter. 2007;158(3):231-3. Epub 2007/07/07. PubMed PMID: 17612283.

801. Paksoy Y, Ozbek O, Gumus S, Koc O, Nayman A, Kerimoglu U. Application of first-pass contrast bolus tracking sequence for the assessment of morphology and flow dynamics in cardiac MRI. Diagn Interv Radiol. 2013;19(1):3-14. Epub 2012/06/23. doi: 10.4261/1305-3825.Dir.5318-11.2. PubMed PMID: 22723088.

802. Ozcan OU, Er HA, Turhan S, Gulec S, Sogut G, Kozluca V, et al. Marked narrowing of right heart chambers due to compression by giant hepatic hydatid cyst. Echocardiography. 2013;30(10):E310-1. Epub 2013/08/03. doi: 10.1111/echo.12335. PubMed PMID: 23906310.

803. Emam Hadi MA, Najari F, Soleimani L. Sudden Death due to Hydatid Cyst Emboli; a Case Report. Emerg (Tehran). 2018;6(1):e20. Epub 2018/07/17. PubMed PMID: 30009222; PubMed Central PMCID: PMCPMC6036530.

804. Akgun V, Battal B, Karaman B, Ors F, Deniz O, Daku A. Pulmonary artery embolism due to a ruptured hepatic hydatid cyst: clinical and radiologic imaging findings. Emerg Radiol. 2011;18(5):437-9. Epub 2011/04/16. doi: 10.1007/s10140-011-0953-8. PubMed PMID: 21494880.

805. Charokopos N, Antonitsis P, Rouska E, Toumbouras M. Calcified aneurysm of the left ventricle mimicking hydatid disease of the lung. Eur J Cardiothorac Surg. 2008;33(5):925. Epub 2008/03/18. doi: 10.1016/j.ejcts.2008.01.068. PubMed PMID: 18343147.

806. Gerazounis M, Athanassiadi K, Metaxas E, Athanassiou M, Kalantzi N. Bronchobiliary fistulae due to echinococcosis. Eur J Cardiothorac Surg. 2002;22(2):306-8. Epub 2002/07/27. doi: 10.1016/s1010-7940(02)00257-9. PubMed PMID: 12142204.

807. Ravis E, Theron A, Lecomte B, Gariboldi V. Pulmonary cyst embolism: a rare complication of hydatidosis. Eur J Cardiothorac Surg. 2018;53(1):286-7. Epub 2017/10/05. doi: 10.1093/ejcts/ezx286. PubMed PMID: 28977397.

808. Erol B, Tetik C, Altun E, Soysal A, Bakir M. Hydatid cyst presenting as a soft-tissue calf mass in a child. Eur J Pediatr Surg. 2007;17(1):55-8. Epub 2007/04/05. doi: 10.1055/s-2007-964949. PubMed PMID: 17407023.

809. Rosenberg T, Panayiotopoulos YP, Bastounis E, Papalambros E, Balas P. Acute abdominal aorta embolism caused by primary cardiac echinococcus cyst. Eur J Vasc Surg. 1993;7(5):582-5. Epub 1993/09/01. PubMed PMID: 8405507.

810. Yague D, Lozano MP, Lample C, Nunez ME, Sanchez F. Bilateral hydatid cyst of pulmonary arteries: MR and CT findings. Eur Radiol. 1998;8(7):1170-2. Epub 1998/09/02. doi: 10.1007/s003300050528. PubMed PMID: 9724432.

811. Patakas D, Pitsiou G, Philippou D, Georgopoulos D, Mavrofridis E. Reversible platypnoea and orthodeoxia after surgical removal of an hydatid cyst from the liver. Eur Respir J. 1999;14(3):725-7. Epub 1999/10/30. doi: 10.1034/j.1399-3003.1999.14c39.x. PubMed PMID: 10543301.

812. Ertekin A, Öcalan D, Öcalan K, Gencer A. Analysis of patients requiring urgent thoracotomy. Eur Rev Med Pharmacol Sci. 2021;25(12):4345-50. doi: 10.26355/eurrev_202106_26143. PubMed PMID: 34227069.

813. Polat P, Atamanalp SS. Hepatic hydatid disease: radiographics findings. Eurasian J Med. 2009;41(1):49-55. Epub 2009/04/01. PubMed PMID: 25610064; PubMed Central PMCID: PMCPMC4261660.

814. Nikmanesh B, Mirhendi H, Mahmoudi S, Rokni MB. Multilocus sequence analysis of Echinococcus granulosus strains isolated from humans and animals in Iran. Exp Parasitol. 2017;183:50-5. Epub 2017/10/13. doi: 10.1016/j.exppara.2017.10.002. PubMed PMID: 29024693.

815. Salamone G, Licari L, Randisi B, Falco N, Tutino R, Vaglica A, et al. Uncommon localizations of hydatid cyst. Review of the literature. G Chir. 2016;37(4):180-5. Epub 2016/12/13. PubMed PMID: 27938537; PubMed Central PMCID: PMCPMC5161223.

816. Martin-Herrero F, Cruz I, Munoz L. Hepatic hydatid cyst rupturing into pericardial cavity. Heart. 2006;92(10):1536. Epub 2006/09/16. doi: 10.1136/hrt.2006.090456. PubMed PMID: 16973812; PubMed Central PMCID: PMCPMC1861029.

817. Herman VS, Hurwitz SS, Conlan AA, Krige LP. Pulmonary hydatid disease: a four-year experience in an urban black hospital. Heart Lung. 1983;12(6):597-600. Epub 1983/11/01. PubMed PMID: 6556172.

818. Turk F, Yuncu G, Atinkaya C, Semerkant T, Ekinci Y, Ozturk G. Hydatid cyst, an unusual cause of spontaneous hemothorax and diagnostic thoracoscopy: case report. Heart Lung. 2012;41(2):192-5. Epub 2011/03/23. doi: 10.1016/j.hrtlng.2011.01.007. PubMed PMID: 21419489.

819. El Hammoumi MM, Achir A, Ouchen F, Lamboni D, Oyali M, Bouchikh M, et al. Historical unilateral pulmonary hydatidosis. Heart Lung Circ. 2013;22(10):877. Epub 2013/02/21. doi: 10.1016/j.hlc.2012.12.018. PubMed PMID: 23422499.

820. Sersar SI, Elnahas HA, Saleh AB, Moussa SA, Ghafar WA. Pulmonary parasitosis: applied clinical and therapeutic issues. Heart Lung Circ. 2006;15(1):24-9. Epub 2006/02/14. doi: 10.1016/j.hlc.2005.04.004. PubMed PMID: 16473787.

821. Shehatha J, Alizzi A, Alward M, Konstantinov I. Thoracic hydatid disease; a review of 763 cases. Heart Lung Circ. 2008;17(6):502-4. Epub 2008/08/05. doi: 10.1016/j.hlc.2008.04.001. PubMed PMID: 18676200.

822. Ziyade S, Soysal O, Ugurlucan M, Yediyildiz S. Pancoast hydatid cyst leading to horner syndrome: thoracic hydatidosis. Heart Lung Circ. 2009;18(5):363-4. Epub 2009/09/22. doi: 10.1016/j.hlc.2008.04.002. PubMed PMID: 19765540.

823. Kanko M, Akbas H, Liman T, Berki KT. Persistent pleural effusion after open heart surgery: giant hydatid cyst of the liver and its demonstrative images. A case report. Heart Surg Forum. 2005;8(5):E378-9. Epub 2005/09/09. doi: 10.1532/hsf98.20051021. PubMed PMID: 16146836.

824. Sabbagh A, Sonon P, Sadissou I, Mendes-Junior CT, Garcia A, Donadi EA, et al. The role of HLA-G in parasitic diseases. Hla. 2018;91(4):255-70. Epub 2018/01/26. doi: 10.1111/tan.13196. PubMed PMID: 29368453.

825. Patankar T, Chotai N, Prasad S, Chowdhry S, Goel A. Intracerebral hydatid cyst in a child with atrial septal defect. Indian Pediatr. 1999;36(4):406-8. Epub 2000/03/16. PubMed PMID: 10717704.

826. Kaminstein D, Heller T, Tamarozzi F. Sound Around the World: Ultrasound for Tropical Diseases. Infect Dis Clin North Am. 2019;33(1):169-95. Epub 2019/02/05. doi: 10.1016/j.idc.2018.10.008. PubMed PMID: 30712760.

827. Scarlata F, Giordano S, Saporito L, Marasa L, Li Pani G, Odierna A, et al. Cystic hydatidosis: a rare case of spine localization. Infez Med. 2011;19(1):39-41. Epub 2011/04/08. PubMed PMID: 21471745.

828. Durhan G, Tan AA, Düzgün SA, Akkaya S, Arıyürek OM. Radiological manifestations of thoracic hydatid cysts: pulmonary and extrapulmonary findings. Insights Imaging. 2020;11(1):116. Epub 20201111. doi: 10.1186/s13244-020-00916-0. PubMed PMID: 33175295; PubMed Central PMCID: PMCPMC7658283.

829. Chazov E, Akchurin R, Lepilin M, Agapov A, Belyaev A, Partigulov S, et al. Giant aneurysm of the coronary artery. Int Angiol. 1991;10(2):106-11. Epub 1991/04/01. PubMed PMID: 1861086.

830. Erkilic S, Ozsarac C, Kocer NE, Bayazit YA. Hydatid cyst of the thyroid gland in a child. Int J Pediatr Otorhinolaryngol. 2004;68(3):369-71. Epub 2004/05/08. doi: 10.1016/j.ijporl.2003.11.002. PubMed PMID: 15129950.

831. Abetti A, Lyazidi S, Qechchar Z, Habbal R, Ettaoumi Y. Hepato-pericardial fistula revealed by a massive pericardial effusion: A case report of an exceptional complication of the hydatid liver cyst. Int J Surg Case Rep. 2020;73:199-202. Epub 20200716. doi: 10.1016/j.ijscr.2020.07.036. PubMed PMID: 32693235; PubMed Central PMCID: PMCPMC7372154.

832. Ben Ismail I, Sghaier M, Boujmil K, Rebii S, Zoghlami A. Hydatid cyst of the liver fistulized into the inferior vena cava. Int J Surg Case Rep. 2022;94:107060. Epub 20220409. doi: 10.1016/j.ijscr.2022.107060. PubMed PMID: 35413675; PubMed Central PMCID: PMCPMC9018139.

833. Messaoudi H, Zayène B, Ben Ismail I, Lajmi M, Lahdhili H, Hachicha S, et al. Bilateral pulmonary hydatidosis associated with uncommon muscular localization. Int J Surg Case Rep. 2020;76:130-3. Epub 20200916. doi: 10.1016/j.ijscr.2020.09.070. PubMed PMID: 33035955; PubMed Central PMCID: PMCPMC7548401.

834. Salih AM, Abdulla ZY, Mohammed DA, Jwamer VI, Ali PG, Hamasaeed AG, et al. Hydatid cyst of thyroid gland, a rare case report with a literature review. Int J Surg Case Rep. 2020;67:267-70. Epub 20200211. doi: 10.1016/j.ijscr.2020.02.019. PubMed PMID: 32097785; PubMed Central PMCID: PMCPMC7036693.

835. Tosya A, Uymaz B, Celebi S, Aybek T. A rare presentation of cystic echinococcosis: aortic involvement. Interact Cardiovasc Thorac Surg. 2015;21(4):548-9. Epub 2015/07/17. doi: 10.1093/icvts/ivv153. PubMed PMID: 26180090.

836. Geramizadeh B. Unusual locations of the hydatid cyst: a review from iran. Iran J Med Sci. 2013;38(1):2-14. Epub 2013/05/07. PubMed PMID: 23645952; PubMed Central PMCID: PMCPMC3642939.

837. Mowlavi G, Shirani S, Askari Z, Dupouy-Camet J, Kacki S, Fasihi Harandi M, et al. Dual-Source Dual-Energy CT-Scan Confirms the Diagnosis of Ancient Hydatid Cysts Recovered from a Late Roman Burial in Amiens, France. Iran J Parasitol. 2022;17(2):194-201. doi: 10.18502/ijpa.v17i2.9536. PubMed PMID: 36032750; PubMed Central PMCID: PMCPMC9363259.

838. Özkaçmaz S. Computed Tomography Findings of Ruptured Hepatic Hydatid Cyst into the Pericardial Space: A Case Report. Iran J Parasitol. 2019;14(4):674-8. PubMed PMID: 32099573; PubMed Central PMCID: PMCPMC7028240.

839. Sabzi F, Faraji R. Multiple Complications by Hydatid Cyst-induced Budd Chiary Syndrome: A Case Report. Iran J Parasitol. 2017;12(1):148-51. Epub 2017/08/02. PubMed PMID: 28761473; PubMed Central PMCID: PMCPMC5522693.

840. Senturk A, Er M, Karalezli A, Yakut ZI, Soyturk AN, Cetin H, et al. A case of pulmonary artery hydatid cyst observed on endobronchial ultrasound. Iran J Radiol. 2015;12(1):e15995. Epub 2015/03/21. doi: 10.5812/iranjradiol.15995. PubMed PMID: 25793087; PubMed Central PMCID: PMCPMC4349105.

841. Azzarelli S, Galassi AR, Centamore G, Carini V. [Electrocardiographic picture of a previous myocardial infarction in a patient with liver echinococcosis cyst]. Ital Heart J Suppl. 2003;4(10):866-8. Epub 2003/12/11. PubMed PMID: 14664299.

842. Abad-Torrent A, Sueiras-Gil A, Martinez-Vilalta M, Vallet-Fernandez J, Guisasola-Rabes M. Monitoring of the intraoperative analgesia by pupillometry during laparoscopic splenectomy for splenic hydatid cyst. J Clin Anesth. 2017;36:94-7. Epub 2017/02/12. doi: 10.1016/j.jclinane.2016.10.021. PubMed PMID: 28183584.

843. Gupta A, Bhardwaj N, Sarkar S, Kanojia RP. Endoscopic excision of hydatid cyst: A case of delayed anaphylaxis in postoperative period - "The risk is not over until it is over". J Clin Anesth. 2019;52:63-4. Epub 2018/09/15. doi: 10.1016/j.jclinane.2018.09.019. PubMed PMID: 30216926.

844. Zhou M, Xu D, Zhang W, Wang Y, Zuo M, Wang S, et al. The hepato-cardiac disorders in Tibetan residents with hepatic echinococcosis: A case-control echocardiography study. J Clin Ultrasound. 2022;50(9):1251-9. Epub 20220808. doi: 10.1002/jcu.23287. PubMed PMID: 36353905.

845. Demircan A, Keles A, Kahveci FO, Tulmac M, Ozsarac M. Cardiac tamponade via a fistula to the pericardium from a hydatid cyst: case report and review of the literature. J Emerg Med. 2010;38(5):582-6. Epub 2007/12/11. doi: 10.1016/j.jemermed.2007.07.017. PubMed PMID: 18065186.

846. Byard RW. An analysis of possible mechanisms of unexpected death occurring in hydatid disease (echinococcosis). J Forensic Sci. 2009;54(4):919-22. Epub 2009/05/27. doi: 10.1111/j.1556-4029.2009.01065.x. PubMed PMID: 19467137.

847. Sobrino JM, Pulpon LA, Crespo MG, Silva L, Segovia J, Serrano-Fiz S, et al. Heart transplantation in a patient with liver hydatidosis. J Heart Lung Transplant. 1993;12(3):531-3. Epub 1993/05/01. PubMed PMID: 8329434.

848. Hizem A, M'Rad S, Oudni-M'rad M, Mestiri S, Hammedi F, Mezhoud H, et al. Molecular genotyping of Echinococcus granulosus using formalin-fixed paraffin-embedded preparations from human isolates in unusual tissue sites. J Helminthol. 2016;90(4):417-21. Epub 2015/07/21. doi: 10.1017/s0022149x15000516. PubMed PMID: 26190231.

849. Katkhouda N, Mavor E, Gugenheim J, Mouiel J. Laparoscopic management of benign cystic lesions of the liver. J Hepatobiliary Pancreat Surg. 2000;7(2):212-7. Epub 2000/09/12. doi: 10.1007/s005340000070212.534. PubMed PMID: 10982616.

850. Capoglu I, Unuvar N, Erdogan F, Yilmaz O, Caydere M. A hydatid cyst of the thyroid gland. J Int Med Res. 2002;30(2):206-9. Epub 2002/05/25. doi: 10.1177/147323000203000216. PubMed PMID: 12025531.

851. Menassa-Moussa L, Braidy C, Riachy M, Tabet G, Smayra T, Haddad-Zebouni S, et al. [Hydatid disease diagnosed following a pulmonary embolism]. J Mal Vasc. 2009;34(5):354-7. Epub 2009/07/21. doi: 10.1016/j.jmv.2009.04.004. PubMed PMID: 19615835.

852. Farcas CP, Radulescu A, Dinu M, Madan V, Bratu O, Spinu D, et al. Echinococcal cyst of the left vas deferens - a case report and literature review. J Med Life. 2014;7 Spec No. 2:54-7. Epub 2014/01/01. PubMed PMID: 25870674; PubMed Central PMCID: PMCPMC4391357.

853. Aala F, Badali H, Hashemi Fesharaki S, Boroumand M, Sotoudeh Anvari M, Davari H, et al. Coexistence of aspergilloma and pulmonary hydatid cyst in an immunocompetent individual. J Mycol Med. 2017;27(3):396-9. Epub 2017/05/21. doi: 10.1016/j.mycmed.2017.04.006. PubMed PMID: 28526521.

854. Cheraghipour K, Masoori L, Zivdari M, Beiranvand M, Malekara V, Yarahmadi V, et al. A systematic appraisal of the use of carvacrol-rich plants to treat hydatid cysts. J Parasit Dis. 2022;46(3):916-22. Epub 20220116. doi: 10.1007/s12639-021-01461-3. PubMed PMID: 36091288; PubMed Central PMCID: PMCPMC9458781.

855. Shukla AK, Peter A, Arya V, Dwivedi V, Gupta MK, Rai N, et al. A rare case of hydatid cyst of the neck with concurrent pulmonary hydatid disease. J Parasit Dis. 2022;46(4):941-4. Epub 20220712. doi: 10.1007/s12639-022-01516-z. PubMed PMID: 36457772; PubMed Central PMCID: PMCPMC9606158.

856. Cakir M, Calikoglu C, Yilmaz A. A Very Rare Complication of Cerebral Hydatid Cyst Surgery: Cortical Collapse. J Pediatr Neurosci. 2017;12(4):346-8. Epub 2018/04/21. doi: 10.4103/jpn.JPN_82_17. PubMed PMID: 29675074; PubMed Central PMCID: PMCPMC5890555.

857. Abu-Eshy SA. Some rare presentations of hydatid cyst (Echinococcus granulosus). J R Coll Surg Edinb. 1998;43(5):347-52. Epub 1998/11/06. PubMed PMID: 9803111.

858. Laissy JP, Fernandez P, Mousseaux E, Dacher JN, Crochet D. [Cardiac tumors]. J Radiol. 2004;85(4 Pt 1):363-9. Epub 2004/06/24. PubMed PMID: 15213645.

859. Kahyaoglu M, Gecmen C, Izgi IA. Hydatid Cyst Mimicking a Pericardial Cyst. J Tehran Heart Cent. 2017;12(4):192-3. Epub 2018/03/27. PubMed PMID: 29576789; PubMed Central PMCID: PMCPMC5849594.

860. Camporrotondo M, Vrancic M, Piccinini F, Navia D. Surgical treatment of pulmonary hypertension caused by echinococcosis disease. J Thorac Cardiovasc Surg. 2014;147(3):e15-6. Epub 2013/11/23. doi: 10.1016/j.jtcvs.2013.10.015. PubMed PMID: 24263009.

861. Vasquez JC, DeLaRosa J, Montesinos E, Rojas L, Peralta J, Leon JJ. Severe mitral regurgitation and hepatopulmonary hydatid cysts: what should be treated first? J Trop Pediatr. 2008;54(6):420-1. Epub 2008/07/10. doi: 10.1093/tropej/fmn055. PubMed PMID: 18611958.

862. Basnet P, Chapagain S, Neupane R, Thapa A. Rare Encounter of Renal Hydatid Cyst: A Case Report. JNMA J Nepal Med Assoc. 2021;59(239):716-8. Epub 20210730. doi: 10.31729/jnma.6994. PubMed PMID: 34508491; PubMed Central PMCID: PMCPMC9107858.

863. Lee KT, Lai WT, Yen HW, Voon WC, Hwang CH, Lu YH, et al. Cystic left atrium myxoma--a rare case report. Kaohsiung J Med Sci. 2001;17(11):579-81. Epub 2002/02/21. PubMed PMID: 11852466.

864. Rezaei R, Soroush N, Rezaee K, Zehi V. Perforated lung hydatid cyst presenting with tension pneumothorax and cardiac arrest. Kardiochir Torakochirurgia Pol. 2020;17(3):165-7. Epub 20200923. doi: 10.5114/kitp.2020.99082. PubMed PMID: 33014094; PubMed Central PMCID: PMCPMC7526484.

865. McDonald A, McDonald L, Resnekov L, Robinson M, Ross D. Homograft replacement of the aortic valve. Immediate results and follow-up. Lancet. 1968;2(7566):469-74. Epub 1968/08/31. doi: 10.1016/s0140-6736(68)90645-4. PubMed PMID: 4174503.

866. Linquette M, Vandecasteele J, Swyngedauw J, Mesmacque R, Gasnault JP. [Large hydatid cyst of the liver revealing congenital cardiopathy: postoperative results]. Lille Med. 1968;13(5):563-7. Epub 1968/05/01. PubMed PMID: 5738903.

867. Li F, Yang M, Li B, Yan L, Zen Y, Wen T, et al. Initial clinical results of orthotopic liver transplantation for hepatic alveolar echinococcosis. Liver Transpl. 2007;13(6):924-6. Epub 2007/06/01. doi: 10.1002/lt.21187. PubMed PMID: 17538987.

868. Sarkar M, Pathania R, Jhobta A, Thakur BR, Chopra R. Cystic pulmonary hydatidosis. Lung India. 2016;33(2):179-91. Epub 2016/04/07. doi: 10.4103/0970-2113.177449. PubMed PMID: 27051107; PubMed Central PMCID: PMCPMC4797438.

869. Sokouti M, Sokouti B, Shokouhi B, Rahimi-Rad MH. Multi-vesicular pulmonary hydatid cyst, the potent underestimated factor in the formation of daughter cysts of pulmonary hydatid disease. Lung India. 2015;32(4):375-7. Epub 2015/07/17. doi: 10.4103/0970-2113.159583. PubMed PMID: 26180389; PubMed Central PMCID: PMCPMC4502204.

870. Solano Remirez M, Urbieta Echezarreta MA, Alvarez Frias MT, Gonzalez Arencibia C, Llorente Diaz B. [Cardiac tamponade caused by hydatid pericarditis]. An Med Interna. 2005;22(7):326-8. Epub 2005/11/18. PubMed PMID: 16288577.

871. Varro J, Mathew L, Athyal RP, Khafagy AH. Percutaneous alcohol sclerotherapy of a hepatic hydatid cyst after balloon occlusion of a large biliary communication. Med Princ Pract. 2011;20(5):477-9. Epub 2011/07/16. doi: 10.1159/000328424. PubMed PMID: 21757940.

872. Majdandzic J, Kremer G, Langer KH, Stein G. [Multilocular echinococcosis]. Med Welt. 1981;32(26):1060-4. Epub 1981/06/26. PubMed PMID: 7253912.

873. Leone JL, Fernandez E, Krausz JP, Rios C, Tursi A. [Dilated cardiomyopathy and hypereosinophilia in a young female patient]. Medicina (B Aires). 1993;53(1):50-3. Epub 1993/01/01. PubMed PMID: 8246731.

874. Eken H, Isik A, Balci G, Firat D, Cimen O, Soyturk M. A Rare Case of Isolated Cystic Hydatid of Thyroid Gland. Medicine (Baltimore). 2016;95(10):e2929. Epub 2016/03/11. doi: 10.1097/md.0000000000002929. PubMed PMID: 26962790; PubMed Central PMCID: PMCPMC4998871.

875. Zhao Q, Luo J, Zhang Q, Leng T, Yang L. Laparoscopic surgery for primary ovarian and retroperitoneal hydatid disease: A case report. Medicine (Baltimore). 2018;97(3):e9667. Epub 2018/03/06. doi: 10.1097/md.0000000000009667. PubMed PMID: 29505009; PubMed Central PMCID: PMCPMC5779778.

876. Weinzierl M, Bull U, Kruis W, Eisenburg J. [Embolization of the lung in echinococcosis (author's transl)]. MMW Munch Med Wochenschr. 1977;119(44):1419-22. Epub 1977/11/04. PubMed PMID: 412081.

877. Hall FM. Images in clinical medicine. Echinococcal cyst of the pericardium. N Engl J Med. 1995;333(3):165. Epub 1995/07/20. doi: 10.1056/nejm199507203330306. PubMed PMID: 7791819.

878. Kanj AH, Fares YH, Yehya RR, Hamzeh FF. Unusual appearance of a cerebral hydatid cyst as a hemorrhagic infarct. Neurosciences (Riyadh). 2010;15(4):275-6. Epub 2010/10/20. PubMed PMID: 20956927.

879. Bharati S, Pal M. Primary hydatid cyst in gastrocnemius muscle. Niger J Surg. 2012;18(1):19-21. Epub 2012/01/01. doi: 10.4103/1117-6806.95479. PubMed PMID: 24027387; PubMed Central PMCID: PMCPMC3716238.

880. Reza HAM, Rreza G, Nastaran B, Mousa M. Renal hydatid cyst; a rare infectious disease. Oxf Med Case Reports. 2019;2019(3):omz011. Epub 2019/04/06. doi: 10.1093/omcr/omz011. PubMed PMID: 30949349; PubMed Central PMCID: PMCPMC6440275.

881. Fenane H, Maidi el M, Bouchikh M, Lamboni D, Achir A, Ouchen F, et al. [Pericardial hydatidosis]. Pan Afr Med J. 2015;20:375. Epub 2015/07/18. doi: 10.11604/pamj.2015.20.375.3124. PubMed PMID: 26185566; PubMed Central PMCID: PMCPMC4499234.

882. Ouzzad O, Kechna H, Moudden MK, Chkoura K, Hanafi SM. [Cardiac arrest during liver hydatid cyst surgery]. Pan Afr Med J. 2015;22:32. Epub 2015/12/15. doi: 10.11604/pamj.2015.22.32.6909. PubMed PMID: 26664533; PubMed Central PMCID: PMCPMC4662535.

883. Mezioug D, Touil-Boukoffa C. [Cytokine profile in human hydatidosis: possible role in the immunosurveillance of patients infected with Echinococcus granulosus]. Parasite. 2009;16(1):57-64. Epub 2009/04/10. doi: 10.1051/parasite/2009161057. PubMed PMID: 19353953.

884. Andronikou S, Welman CJ, Kader E. Classic and unusual appearances of hydatid disease in children. Pediatr Radiol. 2002;32(11):817-28. Epub 2002/10/22. doi: 10.1007/s00247-002-0785-5. PubMed PMID: 12389111.

885. Choudhury SR, Chadha R, Mishra A, Kumar V, Singh V, Dubey NK. Lung resections in children for congenital and acquired lesions. Pediatr Surg Int. 2007;23(9):851-9. Epub 2007/08/03. doi: 10.1007/s00383-007-1940-8. PubMed PMID: 17671788.

886. Monge-Maillo B, Olmedo Samperio M, Perez-Molina JA, Norman F, Mejia CR, Tojeiro SC, et al. Osseous cystic echinococcosis: A case series study at a referral unit in Spain. PLoS Negl Trop Dis. 2019;13(2):e0007006. Epub 2019/02/20. doi: 10.1371/journal.pntd.0007006. PubMed PMID: 30779741; PubMed Central PMCID: PMCPMC6396934.

887. Sors C, Debesse B, Heintz C, Bousser MG, Meurice D, Thomeret G. [Metastatic pulmonary echinococcosis of hepatic origin]. Presse Med. 1971;79(55):2521-5. Epub 1971/12/25. PubMed PMID: 5157910.

888. Ramdani H, Benelhosni K, Billah NM, Nassar I. An unusual cause of pulmonary hypertension: Mediastinal hydatid cyst. Radiol Case Rep. 2022;17(1):86-90. Epub 20211101. doi: 10.1016/j.radcr.2021.09.066. PubMed PMID: 34765067; PubMed Central PMCID: PMCPMC8571532.

889. Lyske J, Hutchinson C, Manolea F, Patel VH, Low G. Case 275: Multiple Hepatic Hydatid Cysts. Radiology. 2020;294(3):716-9. doi: 10.1148/radiol.2019180104. PubMed PMID: 32069185.

890. Kotoulas S, Grapatsas K, Leivaditis V, Panagiotou I, Spiridakis E, Le UT, et al. Massive pulmonary embolism due to hydatid cysts: A rare postoperative complication of liver echinococcosis. Respir Med Case Rep. 2020;30:101054. Epub 20200420. doi: 10.1016/j.rmcr.2020.101054. PubMed PMID: 32373452; PubMed Central PMCID: PMCPMC7193314.

891. Marchiori E, Zanetti G, Hochhegger B. Mediastinal hydatic cysts: an uncommon cause of mediastinal enlargement. Respiration. 2013;85(2):154-5. Epub 2012/12/12. doi: 10.1159/000345217. PubMed PMID: 23220888.

892. Osorio Aira S, López Pedreira MR. Hepatothoracic hydatid transit with septic pulmonary embolism. Rev Clin Esp (Barc). 2021;221(2):127. Epub 20191205. doi: 10.1016/j.rceng.2019.05.017. PubMed PMID: 33998489.

893. Garcia-Velasco P, Garcia C, Parramon F, Villalonga A, Beltran de Heredia B. [Gas embolism secondary to intraoperative use of hydrogen peroxide]. Rev Esp Anestesiol Reanim. 1997;44(3):124-6. Epub 1997/03/01. PubMed PMID: 9229733.

894. Calleja Rosas F, Iriarte Perez-Pons A, Salas Millan J, Jimenez Cavadas J, Gomez-Angulo Giner JM, Martinez Calzon JL, et al. [Calcified solitary cardiac hydatid cyst fistulating into the right auricle]. Rev Esp Cardiol. 1986;39(5):390-3. Epub 1986/09/01. PubMed PMID: 3786908.

895. Beji M, Louzir B, el Mekki F, Jouini S, Mahouachi R, Daghfous J. [Post hydatid chronic cor pulmonale]. Rev Mal Respir. 1997;14(2):129-31. Epub 1997/04/01. PubMed PMID: 9198835.

896. Serraj M, Smahi M, Kamaoui I, El Houari A, Sahnoune F, Ouadnouni Y, et al. [Hydatic pulmonary embolism: a rare complication of hepatic hydatid cyst]. Rev Mal Respir. 2013;30(3):215-21. Epub 2013/03/19. doi: 10.1016/j.rmr.2012.06.004. PubMed PMID: 23497931.

897. Figueredo E, Liporace V, Mourglia-Ettlin G, Avila HG, Da Rosa D, Rosenzvit M. First genetic characterization of human cystic echinococcosis in Uruguay. Rev Panam Salud Publica. 2022;46:e177. Epub 20221010. doi: 10.26633/rpsp.2022.177. PubMed PMID: 36245908; PubMed Central PMCID: PMCPMC9553022.

898. Aydin Y, Ogul H, Eroglu A. Heart-Shaped lung hydatid cyst in a chest radiograph. Rev Soc Bras Med Trop. 2022;55:e0099. Epub 20220606. doi: 10.1590/0037-8682-0099-2022. PubMed PMID: 35674556; PubMed Central PMCID: PMCPMC9176721.

899. Aletras HA. Embolism of the popliteal artery by a ruptured hydatid cyst of the heart. Scand J Thorac Cardiovasc Surg. 1967;1(3):188-90. Epub 1967/01/01. PubMed PMID: 5601690.

900. Kambam JR, Dymond R, Krestow M, Handte RE. Efficacy of histamine H1 and H2 receptor blockers in the anesthetic management during operation for hydatid cysts of liver and lungs. South Med J. 1988;81(8):1013-5. Epub 1988/08/01. doi: 10.1097/00007611-198808000-00018. PubMed PMID: 2900555.

901. Ozkan F, Yesilkaya Y, Tokur M, Ozcan N, Inci MF. Embolization of Ruptured Hepatic Hydatid Cyst to Pulmonary Artery in an Elderly Patient: Multidetector computed tomography findings. Sultan Qaboos Univ Med J. 2013;13(1):165-8. Epub 2013/04/11. PubMed PMID: 23573401; PubMed Central PMCID: PMCPMC3616786.

902. Giuliante F, D'Acapito F, Vellone M, Giovannini I, Nuzzo G. Risk for laparoscopic fenestration of liver cysts. Surg Endosc. 2003;17(11):1735-8. Epub 2003/06/13. doi: 10.1007/s00464-002-9106-1. PubMed PMID: 12802647.

903. Delay D, Lardi C, Jaussi A, von Segesser LK. Hepato-atrial anastomosis, the "other Senning operation" for treatment of Budd-Chiari syndrome. Swiss Med Wkly. 2005;135(15-16):235-7. Epub 2005/06/23. doi: 2005/15/smw-11002. PubMed PMID: 15971116.

904. Erdogan A. Recurrent primary vascular echinococcosis. Tex Heart Inst J. 2005;32(1):113. Epub 2005/05/21. PubMed PMID: 15902840; PubMed Central PMCID: PMCPMC555842.

905. Oz N, Turkay C, Golbasi I, Dertsiz L, Sarper A, Demircan A, et al. Primary vascular echinococcosis: an uncommon cause of chronic iliofemoral arterial occlusion. Tex Heart Inst J. 2000;27(2):209-11. Epub 2000/08/06. PubMed PMID: 10928511; PubMed Central PMCID: PMCPMC101058.

906. Usluer O, Ceylan KC, Kaya S, Sevinc S, Gursoy S. Surgical management of pulmonary hydatid cysts: is size an important prognostic indicator? Tex Heart Inst J. 2010;37(4):429-34. Epub 2010/09/17. PubMed PMID: 20844615; PubMed Central PMCID: PMCPMC2929855.

907. Xia D, Yan LN, Li B, Zeng Y, Cheng NS, Wen TF, et al. Orthotopic liver transplantation for incurable alveolar echinococcosis: report of five cases from west China. Transplant Proc. 2005;37(5):2181-4. Epub 2005/06/21. doi: 10.1016/j.transproceed.2005.03.111. PubMed PMID: 15964373.

908. Gupta BB, Schangole S, Rnandagawali V, Kjadhav A. Spontaneous cutaneous fistulization, eventration of right hemidiaphragm and invasion of the pericardial cavity by a liver hydatid cyst. Trop Gastroenterol. 2013;34(4):287-9. Epub 2014/07/23. PubMed PMID: 25046901.

909. Nag C, Ghosh M, Ghosh T, Dey S, Maji P. Hydatid cyst in rectus abdominis muscle in a child: An unusual occurrence. Trop Parasitol. 2011;1(2):135-7. Epub 2011/07/01. doi: 10.4103/2229-5070.86967. PubMed PMID: 23508209; PubMed Central PMCID: PMCPMC3593496.

910. Bulut B, Aydın C, Bayhan G, Akın ME, Birincioğlu CL. [A liver cycst hydatid case with widespread embolies to vena cava inferior]. Tuberk Toraks. 2020;68(3):346-50. doi: 10.5578/tt.69499. PubMed PMID: 33295735.

911. Bellil S, Limaiem F, Bellil K, Chelly I, Mekni A, Haouet S, et al. [Descriptive epidemiology of extrapulmonary hydatid cysts: a report of 265 Tunisian cases]. Tunis Med. 2009;87(2):123-6. Epub 2009/06/16. PubMed PMID: 19522445.

912. Mokaddem A, Sdiri W, Selmi K, Bachraoui K, Makni H, Dargouth B, et al. [Hydatid pulmonary embolism]. Tunis Med. 2003;81(11):894-7. Epub 2004/02/28. PubMed PMID: 14986547.

913. Bavullu EN, Aksoy E, Abdullayev R, Gogus N, Dede D. Comparison of Dexmedetomidine and Midazolam in Sedation for Percutaneous Drainage of Hepatic Hydatid Cysts. Turk J Anaesthesiol Reanim. 2013;41(6):195-9. Epub 2013/12/01. doi: 10.5152/tjar.2013.40. PubMed PMID: 27366371; PubMed Central PMCID: PMCPMC4894208.

914. Erdem MR, Akbas A, Onol FF, Tanidir Y, Onol SY. An unusual retroperitoneal sero-negative hydatid cyst presenting with lower urinary tract symptoms. Turkiye Parazitol Derg. 2009;33(1):82-4. Epub 2009/04/16. PubMed PMID: 19367553.

915. El Farhaoui H, El Houmaidi A, Mhanna T, Aynaou M, Jdaini A, Barki A. Giant hydatid cyst of the right kidney discovered in a subicterus table. Urol Case Rep. 2020;32:101210. Epub 20200419. doi: 10.1016/j.eucr.2020.101210. PubMed PMID: 32346516; PubMed Central PMCID: PMCPMC7183154.

916. Malakzai HA, Baset Z, Ibrahimkhil AS, Rahimi MS, Khan J, Hanifi AN. Primary hydatid cyst of the urinary bladder with associated eosinophilic cystitis: Report of a unique case. Urol Case Rep. 2023;46:102296. Epub 20221130. doi: 10.1016/j.eucr.2022.102296. PubMed PMID: 36506796; PubMed Central PMCID: PMCPMC9731857.

917. Saeedan MB, Aljohani IM, Alghofaily KA, Loutfi S, Ghosh S. Thoracic hydatid disease: A radiologic review of unusual cases. World J Clin Cases. 2020;8(7):1203-12. doi: 10.12998/wjcc.v8.i7.1203. PubMed PMID: 32337194; PubMed Central PMCID: PMCPMC7176618.

918. Dziri C, Haouet K, Fingerhut A, Zaouche A. Management of cystic echinococcosis complications and dissemination: where is the evidence? World J Surg. 2009;33(6):1266-73. Epub 2009/04/08. doi: 10.1007/s00268-009-9982-9. PubMed PMID: 19350321.

919. Prousalidis J, Tzardinoglou K, Sgouradis L, Katsohis C, Aletras H. Uncommon sites of hydatid disease. World J Surg. 1998;22(1):17-22. Epub 1998/02/18. doi: 10.1007/s002689900343. PubMed PMID: 9465756.

920. Ramos G, Orduna A, Garcia-Yuste M. Hydatid cyst of the lung: diagnosis and treatment. World J Surg. 2001;25(1):46-57. Epub 2001/02/24. doi: 10.1007/s002680020007. PubMed PMID: 11213156.

921. Kotoulas CS, Foroulis C, Letsas K, Kostikas K, Konstantinou M. Bilious pericardial effusion at initial presentation in a patient with lung cancer. World J Surg Oncol. 2003;1(1):24. Epub 2003/11/14. doi: 10.1186/1477-7819-1-24. PubMed PMID: 14613553; PubMed Central PMCID: PMCPMC280706.

922. Sadashiva N, Shukla D, Devi BI. Rupture of Intraventricular Hydatid Cyst: Camalote Sign. World Neurosurg. 2018;110:115-6. Epub 2017/11/18. doi: 10.1016/j.wneu.2017.11.019. PubMed PMID: 29146436.

923. Kosak M, Obrez I, Jezernik J, Kosin M. [Echinococcosis of the heart]. Acta Chir Iugosl. 1981;28(2):277-80. Epub 1981/01/01. PubMed PMID: 7340357.

924. Maksimovic R, Seferovic PM, Ristic AD, Opincal TS, Dunjic MK, Markovic Z, et al. [Diagnosis of pericardial cysts: significance of cardiac magnetic resonance and literature review]. Acta Chir Iugosl. 2007;54(3):53-7. Epub 2007/11/09. PubMed PMID: 17988031.

925. Nalbanski B, Popivanova P, Lachev V, Ivanov S, Tsekova K. [A case of pregnancy with fetal malformation and an hepatic hydatid cyst]. Akush Ginekol (Sofiia). 2001;42(2):29-31. Epub 2002/01/22. doi: 10.3201/eid0801.010017. PubMed PMID: 11799753.

926. Manduz S, Katrancioglu N, Dogan K. [A cardiac hydatid cyst case seen after operation on pulmonary hydatid cyst]. Anadolu Kardiyol Derg. 2007;7(4):440-1. Epub 2007/12/11. PubMed PMID: 18065345.

927. Mese B, Uygur F, Erdogan MB, Asil R, Yamak B. [Surgical treatment of isolated cardiac echinococcosis, located in the right ventricular outflow tract]. Anadolu Kardiyol Derg. 2006;6(4):372-3. Epub 2006/12/13. PubMed PMID: 17162288.

928. Deliagina EM. [Primary echinococcosis of the heart]. Arkh Patol. 1969;31(5):78-9. Epub 1969/01/01. PubMed PMID: 5383744.

929. Golosovskaia MA. [Ossification in the myocardium]. Arkh Patol. 1966;28(12):67-9. Epub 1966/01/01. PubMed PMID: 4973159.

930. Zasetskii EE. [Primary alveolar echinococcus of the heart]. Arkh Patol. 1967;29(8):75-7. Epub 1967/01/01. PubMed PMID: 5601715.

931. de Oliveira SA, de Souza MR, Bittencourt D, Pileggi F, Macruz R, Zerbini EJ. [Hydatid cyst of the heart. Presentation of a case]. Arq Bras Cardiol. 1967;20(1):43-6. Epub 1967/02/01. PubMed PMID: 5609845.

932. Milei J, Fortunato MR, Mautner B, Storino RA, Gugliotta H. Hydatid cyst embolization to the left ventricle: a case report. Arq Bras Cardiol. 1988;50(3):189-91. Epub 1988/03/01. PubMed PMID: 3228369.

933. Anastasatu C, Burnea D, Braileanu A, Caracas P, Raiciulescu N, Niculescu-Zinca D. [Pulmonary scintigraphic changes in chronic cor pulmonale caused by tuberculosis and other non-tubercular diseases]. Ftiziologia. 1972;21(1):37-48. Epub 1972/01/01. PubMed PMID: 4664521.

934. Barcan F, Popescu P. [Thoracic calcifications]. Ftiziologia. 1974;23(1):13-24. Epub 1974/01/01. PubMed PMID: 4822456.

935. Biriukov Iu V, Gilevich M, Volkov Iu A, Golik AD. [Clinico-morphological bases of the choice of surgical method in echinococcosis of the organs of the thoracic cavity]. Grud Serdechnososudistaia Khir. 1990;(9):50-4. Epub 1990/01/01. PubMed PMID: 2261257.

936. Adil'gireeva L, Grents VG, Erenburg TA. [Complete atrioventricular block caused by single-chamber echinococcosis of the ventricular septum]. Grudn Khir. 1981;(6):78-9. Epub 1981/01/01. PubMed PMID: 7333516.

937. Altynkov PG. [Cases of echinococcosis of the heart]. Grudn Khir. 1966;8(6):85-6. Epub 1966/11/01. PubMed PMID: 6003777.

938. Astaf'ev VI, Zheltovskii Iu V, Kononenko VN, Noskov VS. [Echinococcal cyst of the left ventricle of the heart]. Grudn Khir. 1982;(4):83-5. Epub 1982/01/01. PubMed PMID: 7117895.

939. Avdalbekian S, Apoian VT, Khachatrian VL. [Echinococcosis of the heart]. Grudn Khir. 1980;4:25-8. Epub 1980/07/01. PubMed PMID: 7399301.

940. Chukhrienko DP, Mil'kov BO. [Resection of calcified echinococcal cysts and aneurysms of the heart left ventricle]. Grudn Khir. 1967;9(2):103. Epub 1967/03/01. PubMed PMID: 5603402.

941. Feofilov GL, Pak LA, Rychkova NA. [Echinococcosis of the heart]. Grudn Khir. 1986;(4):91-2. Epub 1986/07/01. PubMed PMID: 3758810.

942. Kalandadze ZF. [Simultaneous operation of removing echinococcal cysts of the lung and myocardium]. Grudn Khir. 1975;(3):115-6. Epub 1975/05/01. PubMed PMID: 1205242.

943. Paereli RS. [Remote results of the observations of a patient after resection of an extensive portion of the myocardium of the left ventricle]. Grudn Khir. 1967;9(2):105. Epub 1967/03/01. PubMed PMID: 5603403.

944. Behar S, Kreisler B, Kariv I. [Echinococcosis of the heart with ventricular tachycardia]. Harefuah. 1971;81(2):68-71. Epub 1971/07/15. PubMed PMID: 5160504.

945. Siniawski H, Lehmkuhl H, Pasic M, Weng Y, Hetzer R. [Echinococcus cyst in the left ventricle. The role of echocardiography]. Kardiol Pol. 2006;64(9):1015-7. Epub 2006/10/21. PubMed PMID: 17054036.

946. Tiurin EI, Davydenko VA, Kozlov GK. [On the radiodiagnosis of echinococcus of the heart]. Kardiologiia. 1967;7(6):106-7. Epub 1967/06/01. PubMed PMID: 5604291.

947. Aliev VM, Kushnarevich RL, Seksenbaev DS. [Isolated echinococcosis of the pericardium]. Khirurgiia (Mosk). 1968;44(12):110-2. Epub 1968/12/01. PubMed PMID: 5711613.

948. Andrianov VE. [Primary echinococcosis of the pericardium]. Khirurgiia (Mosk). 1978;(5):118. Epub 1978/05/01. PubMed PMID: 661101.

949. Dzhanashvili MI, Ioseliani GO, Tabidze FN. [Echinococcosis of the heart]. Khirurgiia (Mosk). 1981;(2):111. Epub 1981/02/01. PubMed PMID: 7206515.

950. Il'inov VN, Kozlov BN, Kuznetsov MS, Panfilov DS, Nasrashvili GG, Lelik EV, et al. [Surgical treatment of patient with echinococcus cyst of left ventricle apex]. Khirurgiia (Mosk). 2014;(11):70-2. Epub 2015/01/16. PubMed PMID: 25589189.

951. Ivanov VA, Shevelev, II, Nechaenko MA, Kuznetsova LM. [Surgical treatment of heart echinococcosis]. Khirurgiia (Mosk). 1999;(1):35-8. Epub 1999/03/02. PubMed PMID: 10050510.

952. Korolev BA, Korepanova IV. [Surgical treatment of benign tumors and cysts of the mediastinum]. Khirurgiia (Mosk). 1967;43(8):50-6. Epub 1967/08/01. PubMed PMID: 5632751.

953. Malikova MS, Frolova Iu V, Raskin VV, Dzemeshkevich AS, Voronina TS, Parshin VD, et al. [The simultaneous surgery of heart and echinococcosis under artificial blood circulation]. Khirurgiia (Mosk). 2012;(2):79-82. Epub 2012/06/09. PubMed PMID: 22678482.

954. Musaev GK, Sharipov RK, Khalilov AK, Bekshokov AS, Buchulaeva NA. [Cardiac hydatid cyst]. Khirurgiia (Mosk). 2022;(4):101-4. doi: 10.17116/hirurgia2022041101. PubMed PMID: 35477209.

955. Nazyrov FG, Abdumadzhidov Kh A, Buranov Kh D, Akbarov MM, Aliev Sh M, Mukaddirov MM. [Surgical treatment of the combined heart, lung and liver hydatid disease]. Khirurgiia (Mosk). 2009;(5):23-6. Epub 2009/06/06. PubMed PMID: 19491763.

956. Novozhilov AV, Movsisyan MO, Grigoriev SE, Magolina OV, Kleimenova NS. [Combined left hemihepatectomy for multiple organ alveococcosis]. Khirurgiia (Mosk). 2019;(9):52-7. doi: 10.17116/hirurgia201909152. PubMed PMID: 31532167.

957. Serebriakova NI. [On rare complications of unicamerate echinococcosis of the liver]. Khirurgiia (Mosk). 1965;41(12):123-5. Epub 1965/12/01. PubMed PMID: 5878875.

958. Shevchenko Iu L, Musaev G, Borisov IA, Popov LV, Magomedov AG, Akhmedov IG. [Echinococcosis of the heart]. Khirurgiia (Mosk). 2006;(1):11-6. Epub 2006/02/17. PubMed PMID: 16482052.

959. Stojanovic VK. [Tumors of the heart and pericardium]. Khirurgiia (Mosk). 1978;(6):48-54. Epub 1978/06/01. PubMed PMID: 672062.

960. Tarichko Iu V, Karazeev GL, Cherkasov I, Gradoboev MI, Rodionova LV, Maksimkin DA. [The hydatid cyst of the heart]. Khirurgiia (Mosk). 2008;(10):70-2. Epub 2008/12/02. PubMed PMID: 19039873.

961. Usmanov NU, Garipov MK, Tiuriaev AA, Abduzhabbarov AA, Babasaidov SI. [Diagnosis and treatment of echinococcosis in an unusual location]. Khirurgiia (Mosk). 1989;(6):86-90. Epub 1989/06/01. PubMed PMID: 2779133.

962. Chalukov P. [A case of echinosoccosis of the heart]. Khirurgiia (Sofiia). 1973;26(5):432-5. Epub 1973/01/01. PubMed PMID: 4781081.

963. Cherveniakov P, Dervishian M, Ianakiev D, Kolarov I. [Echinococcosis of the heart and pericardium]. Khirurgiia (Sofiia). 1984;37(6):458-63. Epub 1984/01/01. PubMed PMID: 6527488.

964. Dimitrov D, Daskalov E. [Primary multiple, simultaneous echinococcosis of the heart, pericardium and anterior and posterior mediastinum]. Khirurgiia (Sofiia). 1981;34(3):281-5. Epub 1981/01/01. PubMed PMID: 7278047.

965. Dimitrov D, Dragoichev C. [Echinococcosis of the heart]. Khirurgiia (Sofiia). 1981;34(4):313-9. Epub 1981/01/01. PubMed PMID: 7278052.

966. Dobrev I, Milev M, Viiachki I. [Primary multiple echinococcosis of the heart]. Khirurgiia (Sofiia). 1973;26(4):318-22. Epub 1973/01/01. PubMed PMID: 4768172.

967. Lazarov Z, Topalov I, Krustinov G. [Echinococcosis of the heart]. Khirurgiia (Sofiia). 1990;43(4):28-32. Epub 1990/01/01. PubMed PMID: 2097419.

968. Petkov I, Kunchev V. [Sudden death in echinococcosis of the heart with a case report]. Khirurgiia (Sofiia). 1983;36(5):478-80. Epub 1983/01/01. PubMed PMID: 6668855.

969. Stoianov G, Iarumov N, Damianov N, Ilieva I. [Liver cirrhosis caused by liver echinococcosis]. Khirurgiia (Sofiia). 2006;(3):20-2. Epub 2008/09/13. PubMed PMID: 18785433.

970. Stoianov G, Ignatov D. [Fatal outcome of anaphylactic shock following surgery of hepatic echinococcosis]. Khirurgiia (Sofiia). 1988;41(2):69-70. Epub 1988/01/01. PubMed PMID: 3411887.

971. Stoianov K, Stanchev P. [On echinococcosis of the heart and pericardium]. Khirurgiia (Sofiia). 1965;18(2):156-61. Epub 1965/01/01. PubMed PMID: 5841876.

972. Garipov MK, Rasulov RR. [Simultaneous mitral commissurotomy and surgical treatment of echinococcosis of the left lung]. Klin Khir. 1990;(10):64. Epub 1990/01/01. PubMed PMID: 2280554.

973. Borisenko VV, Shukin Iu D, Kugoev AI. [Ischemic infarction in a patient with echinococcosis of the heart and brain]. Klin Med (Mosk). 1990;68(4):122-4. Epub 1990/04/01. PubMed PMID: 2370772.

974. Shevtsova BB. [Pulmonary echinococcosis with cardiac metastasis]. Klin Med (Mosk). 1965;43(10):122-3. Epub 1965/10/01. PubMed PMID: 5872466.

975. Trisvetova EL. [Clinical diagnostics of papillary fibroelastoma]. Klin Med (Mosk). 2007;85(12):7-11. Epub 2008/03/06. PubMed PMID: 18318158.

976. Vishnevskii AA, Mazhorov VA, Kopeiko IP, Rubetskoi LS, Savvina TV. [Pulmonary artery embolism from echinococcal cysts]. Klin Med (Mosk). 1979;57(12):76-80. Epub 1979/12/01. PubMed PMID: 533987.

977. Jovic A, Dujella J, Nekic-Borcilo M, Troskot R, Nekic D. [Fatal rupture of an echinococcal cyst into the right atrium]. Lijec Vjesn. 1994;116(11-12):295-8. Epub 1994/11/01. PubMed PMID: 7715409.

978. Kraljevic L, Kostic D, Parpura D, Milovanovic M, Zoltner D. [Pericardial echinococcosis]. Lijec Vjesn. 1973;95(10):566-9. Epub 1973/11/01. PubMed PMID: 4761409.

979. Obradovic Z, Zerem E, Beslagic Z, Susic A. [Echinococcosis in Bosnia and Herzegovina]. Med Arh. 2006;60(4):259-62. Epub 2006/06/10. PubMed PMID: 16761523.

980. Deliagina EM, Deliagin VM. [Unusual localizations of echinococcosis in man]. Med Parazitol (Mosk). 1973;42(2):229-31. Epub 1973/03/01. PubMed PMID: 4805426.

981. Kazachkov EL, Egorov ON. [Primary echinococcosis of the heart in pulmonary tuberculosis]. Med Parazitol (Mosk). 2003;(1):28-30. Epub 2003/03/26. PubMed PMID: 12652846.

982. Ozeretskovskaia NN. [Organ pathology in chronic tissue-dwelling helminthic infections: role of blood and tissue eosinophilia, immunoglobulinemia E, G4, and immune response-inducing factors]. Med Parazitol (Mosk). 2000;(4):9-14. Epub 2001/02/24. PubMed PMID: 11210422.

983. Streliaeva AV, Akhmedov Iu M, Gasparian ER, Lazareva NB, Samylina IA, Chebyshev NV, et al. [Amino acid exchange in paeci lomycosis-complicated echinococcosis]. Med Parazitol (Mosk). 2011;(4):19-23. Epub 2012/02/09. PubMed PMID: 22308707.

984. Streliaeva AV, Sagieva AT, Abdiev FT, Sadykov RV, Sadykov VM, Gabchenko AK, et al. [Cardiac involvement in adults with echinococcosis]. Med Parazitol (Mosk). 2012;(4):40-2. Epub 2013/02/27. PubMed PMID: 23437723.

985. Cesur S, Ciftci A, Sozen TH, Tekeli E. [Case report: a brain hydatid cyst]. Mikrobiyol Bul. 2002;36(2):215-7. Epub 2003/03/26. PubMed PMID: 12652875.

986. Mesic J, Podnar T. [Pseudotumorous formations in the mediastinum]. Nar Zdrav. 1976;32(7-8):341-5. Epub 1976/07/01. PubMed PMID: 1029799.

987. Toda G, Yano K. [Parasitic pericarditis]. Nihon Rinsho. 2007;Suppl 5 Pt 2:362-4. Epub 2007/10/24. PubMed PMID: 17953020.

988. Marton G. [Echinococcal cyst in the thoracic cavity based on 16 cases]. Orv Hetil. 1974;115(23):1332-4. Epub 1974/06/09. PubMed PMID: 4840694.

989. Kocialkowski K. [ECHINOCOCCUS OF THE PERICARDIUM]. Pol Przegl Chir. 1965;37:175-7. Epub 1965/02/01. PubMed PMID: 14321674.

990. Bacalbasa N, Nichiteanu C. [Fatal anaphylactic shock (rupture of hydatid cyst) in the course of general anesthesia]. Rev Chir Oncol Radiol O R L Oftalmol Stomatol Chir. 1980;29(6):467-8. Epub 1980/11/01. PubMed PMID: 6454185.

991. Ionescu SD, Ouatu A, Manea P, Tanase DM. [Malignant hydatid disease--clinical case]. Rev Med Chir Soc Med Nat Iasi. 2011;115(4):1107-12. Epub 2012/01/27. PubMed PMID: 22276455.

992. Rautu F, Manolache C, Nistor S. [Pericardial hydatidosis. A clinical case]. Rev Med Chir Soc Med Nat Iasi. 1993;97(1):489. Epub 1993/01/01. PubMed PMID: 8153482.

993. Ispas R, Ruicanescu I, Marosin C. [Pericardial hydatid cyst and acute myocardial infarct]. Rev Med Interna Neurol Psihiatr Neurochir Dermatovenerol Med Interna. 1984;36(3):283-7. Epub 1984/05/01. PubMed PMID: 6151737.

994. Bordalo AD, Ferreira D, Bordalo e Sa AL, Tuna JL, Correia MJ, Pais F, et al. [A case of incessant junctional tachycardia in a female patient with aneurysm of the interauricular septum]. Rev Port Cardiol. 1992;11(6):561-81. Epub 1992/06/01. PubMed PMID: 1503789.

995. Barnard PM, MacGregor LA, Weich HF. [Primary echinococcus cyst of the heart. A case report]. S Afr Med J. 1989;76(6):275-7. Epub 1989/09/16. PubMed PMID: 2781427.

996. Shargorodskaia AM, Geller AN, Litvinov VA. [A case of echinococcosis of the heart]. Sov Med. 1967;30(7):138. Epub 1967/07/01. PubMed PMID: 5607867.

997. Ivanovic-Krstic B, Kalimanovska-Ostric D, Vujisic-Tesic B, Jovanovic D, Petrovic P, Svetkovic-Matic D. [Cardiac echinococcosis]. Srp Arh Celok Lek. 2002;130(5-6):217-21. Epub 2002/10/25. doi: 10.2298/sarh0206217i. PubMed PMID: 12395448.

998. Jancic-Zguricas M, Isvaneski M. [Parasitic diseases of the heart]. Srp Arh Celok Lek. 1976;104(10):751-60. Epub 1976/10/01. PubMed PMID: 1030523.

999. Markovic Z, Dobricanin S. [Echinococcus of the heart]. Srp Arh Celok Lek. 1977;105(2):217-23. Epub 1977/02/01. PubMed PMID: 905888.

1000. Chazov EI, Akchurin RS, Lepilin MG, Agapov AA, Beliaev AA, Partigulov SA, et al. [A case of a giant aneurysm of the coronary artery]. Ter Arkh. 1990;62(1):112-4. Epub 1990/01/01. PubMed PMID: 2333603.

1001. Strelyaeva AV, Svistunov AA, Dzhomaa RA, Sapozhnikov SA, Chebyshev NV, Ashurov AA, et al. [Pulmonary paecilomycosis: Diagnosis and treatment]. Ter Arkh. 2015;87(3):53-8. Epub 2015/06/02. doi: 10.17116/terarkh201587353-58. PubMed PMID: 26027241.

1002. Ayhan E, Akkaya E, Ekmekci A, Cicek G. [Case images: hydatid cyst of the interventricular septum]. Turk Kardiyol Dern Ars. 2010;38(8):593. Epub 2011/01/21. PubMed PMID: 21248467.

1003. Cakici M, Atilgan ZA, Davutoglu V. [A cystic mass in the interventricular septum: echocardiography and magnetic resonance imaging findings]. Turk Kardiyol Dern Ars. 2010;38(7):521; author reply -2. Epub 2011/01/06. PubMed PMID: 21206215.

1004. Kandemir C, Sahin T, Kilic T, Kanko M. [Hydatid cyst in the left ventricle]. Turk Kardiyol Dern Ars. 2009;37(6):438. Epub 2009/12/19. PubMed PMID: 20019464.

1005. Berktin K, Baygin R, Kamuni A. [Case of echinococcosis with rare multiple localizations]. Turk Tip Cemiy Mecm. 1967;33(10):577-86. Epub 1967/10/01. PubMed PMID: 5601630.

1006. Cakin O, Ustun C, Akcay S, Inci MF, Altinsoy HB. [Primary disseminated pulmonary hydatid cyst manifesting with right-side cardiac failure: case report]. Turkiye Parazitol Derg. 2011;35(4):230-3. Epub 2011/12/27. doi: 10.5152/tpd.2011.59. PubMed PMID: 22198927.

1007. Rahman A, Yucel A, Yilmaz M. [A case of secondary localized pericardial cyst hydatic disease and determination of cyst hydatic scoleces and hooks with various stains]. Turkiye Parazitol Derg. 2008;32(1):31-4. Epub 2008/03/21. PubMed PMID: 18351548.

1008. Borisov VA, Khvalev SV, Zinatullina FM, Nemov AB, Ignatova TN, Chepelevich LS. [Pericardial echinococcosis]. Vestn Khir Im I I Grek. 1999;158(4):84-5. Epub 1999/10/26. PubMed PMID: 10533233.

1009. Gilevich M, Boshno GL, Urusov E. [Diagnosis and surgical treatment of echinococcosis of the heart and pericardium]. Vestn Khir Im I I Grek. 1982;129(9):52-7. Epub 1982/09/01. PubMed PMID: 7147596.

1010. Kolesnikov SA, Kharin V, Bershadenko DD. [Primary benign neoplasms of the heart and their surgical treatment]. Vestn Khir Im I I Grek. 1966;97(11):43-8. Epub 1966/11/01. PubMed PMID: 6002110.

1011. Nazarevskii NG. [On the surgical treatment of echinococcosis]. Vestn Khir Im I I Grek. 1966;97(8):36-8. Epub 1966/08/01. PubMed PMID: 4234683.

1012. Pavlov RK, Demkin VP. [Complications of pericardial echinococci]. Vestn Khir Im I I Grek. 1971;106(2):138-9. Epub 1971/02/01. PubMed PMID: 5117951.

1013. Voronov AA, Vasil'ev VN. [Resection of the anterior wall of the left ventricle because of echinococcosis]. Vestn Khir Im I I Grek. 1967;99(9):113-4. Epub 1967/09/01. PubMed PMID: 5617479.

1014. Didenko VI. [Alveolar echinococcus of the liver with involvement of the inferior vena cava and right auricle of the heart]. Vrach Delo. 1965;9:137-8. Epub 1965/09/01. PubMed PMID: 5871470.

1015. Ianakiev B, Silianov M. [Case of myocardial echinococcosis of the right ventricle]. Vutr Boles. 1978;17(6):106-9. Epub 1978/01/01. PubMed PMID: 735024.

1016. Petrov S. [Echinococcosis of the heart and parasitic embolism of a cerebral artery]. Vutr Boles. 1987;26(3):86-8. Epub 1987/01/01. PubMed PMID: 3617711.

1017. Potapov AA, Goriainov SA, Okhlopkov VA, Kravchuk AD, Zakharova NE, Podoprigora AE, et al. [Multiple echinococcosis of brain, heart and kidneys]. Zh Vopr Neirokhir Im N N Burdenko. 2011;75(1):57-65; discussion Epub 2011/06/28. PubMed PMID: 21698925.

1018. Jin P, Huang YC, Chen AN. [One case of heart cystic echinococcosis in ventricular septum]. Zhongguo Ji Sheng Chong Xue Yu Ji Sheng Chong Bing Za Zhi. 2012;30(2):inside front cover. Epub 2012/08/23. PubMed PMID: 22908801.

1019. Tuersun K, Huang Y. [Changes of blood vessel active substances in acute pulmonary embolism induced by echinococus granulous cyst in rabbits]. Zhonghua Jie He He Hu Xi Za Zhi. 2002;25(10):598-602. Epub 2002/12/20. PubMed PMID: 12490126.

1020. Qian ZX. [Hydatid cyst of the heart: report of 2 cases]. Zhonghua Wai Ke Za Zhi. 1983;21(10):626-7. Epub 1983/10/01. PubMed PMID: 6676002.

1021. Guo XX, Zhang Y, Cheng KA. [Cardiac hydatid disease: a case report]. Zhonghua Xin Xue Guan Bing Za Zhi. 2012;40(1):74-5. Epub 2012/04/12. PubMed PMID: 22490639.

1022. Sun XR. [Cardiac echinococcosis]. Zhonghua Xin Xue Guan Bing Za Zhi. 1983;11(4):289-90. Epub 1983/12/01. PubMed PMID: 6673948.

1023. Huo Q, Yan F, Murat, Ma SF, Qiao J, Zhu T. [Surgical treatment of cardiac echinococcosis]. Zhonghua Yi Xue Za Zhi. 2012;92(40):2855-8. Epub 2013/01/08. PubMed PMID: 23290217.

1024. Polo M, Boero G, Orru L. [Brief considerations on complications of local anesthetics. (Apropos of 3 treated cases)]. Acta Anaesthesiol. 1968;19:Suppl 4:245+. Epub 1968/01/01. PubMed PMID: 4308239.

1025. Giti R, Hosseinzadeh M. Efficacy of Bronchial Washing and Brushing Cytology in the Diagnosis of Non-Neoplastic Lung Diseases. Acta Med Iran. 2017;55(10):636-41. Epub 2017/12/13. PubMed PMID: 29228529.

1026. Turan AA, Karayel F, Akyildiz EU, Ozdes T, Yilmaz E, Pakis I. Sudden death due to eosinophilic endomyocardial diseases: three case reports. Am J Forensic Med Pathol. 2008;29(4):354-7. Epub 2009/03/05. doi: 10.1097/PAF.0b013e3181859fe3. PubMed PMID: 19259026.

1027. Szarfman A, Khoury EL, Cossio PM, Arana RM, Kagan IG. Investigation of the EVI antibody in parasitic diseases other than American trypanosomiasis. An anti-skeletal muscle antibody in leishmaniasis. Am J Trop Med Hyg. 1975;24(1):19-24. Epub 1975/01/01. doi: 10.4269/ajtmh.1975.24.19. PubMed PMID: 1089366.

1028. Christian DJ, Khithani A, Jeyarajah DR. Making liver transection even safer: a novel use of microwave technology. Am Surg. 2011;77(4):417-21. Epub 2011/06/18. PubMed PMID: 21679548.

1029. Velibey Y, Satilmis S, Cagdas M, Altay S, Guzelburc O, Satilmis D, et al. Intramyocardial fissure. Anadolu Kardiyol Derg. 2011;11(3):E11. Epub 2011/04/20. doi: 10.5152/akd.2011.070. PubMed PMID: 21501988.

1030. Monies-Chass I, Wajsbort E, Zveibil FR. Massive aspiration during surgery for a hydatid cyst of the lung. Anaesthesist. 1975;24(4):177-9. Epub 1975/04/01. PubMed PMID: 1138564.

1031. Nardi F, Derenzini M. [Cardiac aneurysm probably of a rheumatic nature]. Ann Anat Pathol (Paris). 1971;16(2):197-204. Epub 1971/04/01. PubMed PMID: 5126044.

1032. Beck-Schimmer B, Breitenstein S, Urech S, De Conno E, Wittlinger M, Puhan M, et al. A randomized controlled trial on pharmacological preconditioning in liver surgery using a volatile anesthetic. Ann Surg. 2008;248(6):909-18. Epub 2008/12/19. doi: 10.1097/SLA.0b013e31818f3dda. PubMed PMID: 19092335.

1033. Katkhouda N, Hurwitz M, Gugenheim J, Mavor E, Mason RJ, Waldrep DJ, et al. Laparoscopic management of benign solid and cystic lesions of the liver. Ann Surg. 1999;229(4):460-6. Epub 1999/04/15. doi: 10.1097/00000658-199904000-00003. PubMed PMID: 10203077; PubMed Central PMCID: PMCPMC1191730.

1034. Kahlbau H, Gomes I, Pinto F, Fragata JI. Uncommon Multicystic Lesion of the Interventricular Septum in a 7-Year-Old Boy: Unusual Presentation of an Intracardiac Teratoma. Ann Thorac Surg. 2016;101(6):2375-7. Epub 2016/05/24. doi: 10.1016/j.athoracsur.2015.09.014. PubMed PMID: 27211950.

1035. Oomman A, Ramachandran P, Santhosham R, Sridhar LF, Ramesh B, Jayaraman S. Cardiac varix in relation to right atrial free wall presenting as a mass compressing the right atrium and mimicking a pericardial cyst. Ann Thorac Surg. 2004;78(6):e96-7. Epub 2004/11/25. doi: 10.1016/j.athoracsur.2003.12.070. PubMed PMID: 15560995.

1036. Faraj W, Deborah Mukherji D, Fakih H, Majzoub N, Khalife M. Liver transplantation in Lebanon: A hard lesson to learn. Ann Transplant. 2010;15(3):25-9. Epub 2010/09/30. PubMed PMID: 20877263.

1037. Singh AV, Walsh JT, Birdi IS. Right atrial thrombus masquerading as intracardiac cyst: a case report. Asian Cardiovasc Thorac Ann. 2008;16(4):e35-6. Epub 2008/08/02. doi: 10.1177/021849230801600424. PubMed PMID: 18670013.

1038. Ortiz B, Hernández C, Barajas NC. A radiological and clinical description of metastatic angiosarcoma simulating a hydatid cyst. Biomedica. 2019;39(3):440-7. Epub 20190901. doi: 10.7705/biomedica.4335. PubMed PMID: 31584759; PubMed Central PMCID: PMCPMC7357370.

1039. Kokulu S, Sivaci RG, Oz G, Baki ED, Senay H, Ela Y. Thoracic epidural anesthesia in a geriatric patient with cardiac risk: a case report. Braz J Anesthesiol. 2014;64(3):215-6. Epub 2014/06/09. doi: 10.1016/j.bjane.2013.06.010. PubMed PMID: 24907884.

1040. Hica L, Ene V, Cristoloveanou D, Papilian VV. [Coelomic cysts of the mediastinum. 12 cases]. Bronches. 1972;22(5):267-81. Epub 1972/09/01. PubMed PMID: 4662513.

1041. Sery Z, Holusa R. The bronchological indications of pulmonary resections in children. Bronches. 1969;19(1):34-57. Epub 1969/01/01. PubMed PMID: 5808019.

1042. Meligrana F, Sugar L, Casella L, Birnbaum PL, Salerno T. Successful removal of an unusual cystic mass of the heart. Can J Cardiol. 1994;10(5):555-8. Epub 1994/06/01. PubMed PMID: 8012886.

1043. Ozlu MF, Ozcan F, Tufekcioglu O. Left ventricular apical cystic thrombus mimicking a hydatid cyst. Can J Cardiol. 2009;25(7):e266. Epub 2009/07/09. doi: 10.1016/s0828-282x(09)70520-3. PubMed PMID: 19584987; PubMed Central PMCID: PMCPMC2723041.

1044. Reddy S, Polito A, Staveski S, Dalton H. A process for academic societies to develop scientific statements and white papers: experience of the Pediatric Cardiac Intensive Care Society. Cardiol Young. 2019;29(2):174-7. Epub 20181204. doi: 10.1017/s1047951118002019. PubMed PMID: 30511598; PubMed Central PMCID: PMCPMC7433703.

1045. Groth KA, Hoyer S, Klaaborg KE, Kim WY, Andersen NH. Get closer to the diagnosis in a flash. Circ Cardiovasc Imaging. 2012;5(2):280-2. Epub 2012/03/23. doi: 10.1161/circimaging.112.972919. PubMed PMID: 22438426.

1046. Madisson-Bernardo M, Bernardo D, Trad HS, Meneghelli U, Villanova M, Schmidt A. Intense Pericardial Involvement in Polycystic Echinococcosis Submitted to Successful Medical Treatment. Circ Cardiovasc Imaging. 2019;12(12):e009826. Epub 20191209. doi: 10.1161/circimaging.119.009826. PubMed PMID: 31813272.

1047. Abouliatim I, Corbineau H, Rugierri VG, Flecher E, Bellouin A, Leguerrier A. Images in cardiovascular medicine. Mitral valve varix. Circulation. 2009;119(19):e529-30. Epub 2009/05/20. doi: 10.1161/circulationaha.108.820175. PubMed PMID: 19451358.

1048. D'Alessandro A, Rausch RL. New aspects of neotropical polycystic (Echinococcus vogeli) and unicystic (Echinococcus oligarthrus) echinococcosis. Clin Microbiol Rev. 2008;21(2):380-401, table of contents. Epub 2008/04/11. doi: 10.1128/cmr.00050-07. PubMed PMID: 18400802; PubMed Central PMCID: PMCPMC2292577.

1049. Walpot J, Shivalkar B, Pasteuning WH, Hokken R. Staphylococcus aureus infective endocarditis mimicking a hydatid cyst. Echocardiography. 2010;27(8):E80-2. Epub 2010/09/21. doi: 10.1111/j.1540-8175.2010.01160.x. PubMed PMID: 20849473.

1050. Basso C, Valente M, Poletti A, Casarotto D, Thiene G. Surgical pathology of primary cardiac and pericardial tumors. Eur J Cardiothorac Surg. 1997;12(5):730-7; discussion 7-8. Epub 1998/02/11. doi: 10.1016/s1010-7940(97)00246-7. PubMed PMID: 9458144.

1051. Ibanez B, Marcos-Alberca P, Rey M, de Rabago R, Orejas M, Renedo G, et al. Multicavitated left atrial myxoma mimicking a hydatid cyst. Eur J Echocardiogr. 2005;6(3):231-3. Epub 2005/05/17. doi: 10.1016/j.euje.2004.09.006. PubMed PMID: 15894245.

1052. Topdagi O, Okcu N, Bilen N. The frequency of complications and the etiology of disease in patients with liver cirrhosis in erzurum. Eurasian J Med. 2014;46(2):110-4. Epub 2015/01/23. doi: 10.5152/eajm.2014.25. PubMed PMID: 25610308; PubMed Central PMCID: PMCPMC4261449.

1053. Janik M, Straka L, Krajcovic J, Hejna P, Hamzik J, Novomesky F. Non-traumatic and spontaneous hemothorax in the setting of forensic medical examination: a systematic literature survey. Forensic Sci Int. 2014;236:22-9. Epub 2014/02/18. doi: 10.1016/j.forsciint.2013.12.013. PubMed PMID: 24529771.

1054. Barton A. [The radiological recognition of calcification of the atrium]. Fortschr Geb Rontgenstr Nuklearmed. 1967;106(2):241-6. Epub 1967/02/01. PubMed PMID: 5626315.

1055. Wang Z, Liang X, Lu Y, Jiang T, Aji T, Aimulajiang K, et al. Insomnia Promotes Hepatic Steatosis in Rats Possibly by Mediating Sympathetic Overactivation. Front Physiol. 2021;12:734009. Epub 20210924. doi: 10.3389/fphys.2021.734009. PubMed PMID: 34630154; PubMed Central PMCID: PMCPMC8497715.

1056. Narayanapillai J, Madhavan S, Shankaragouda BH. Cystic mediastinal mass. Heart Asia. 2018;10(2):e011071. Epub 2018/08/18. doi: 10.1136/heartasia-2018-011071. PubMed PMID: 30116306; PubMed Central PMCID: PMCPMC6088339.

1057. Cunha BA, Pherez FM, Katz DS. Fever of unknown origin (FUO) due to a solitary cavitary lung lesion: the deadly ferritin-laced doughnut. Heart Lung. 2010;39(4):340-4. Epub 2010/06/22. doi: 10.1016/j.hrtlng.2009.07.010. PubMed PMID: 20561835.

1058. Boutayeb A, Marmade L, Laaroussi M, Bensouda A, Moughil S. Textiloma mimicking a pericardial hydatid cyst: a case report. Heart Lung Circ. 2012;21(5):278-80. Epub 2012/03/06. doi: 10.1016/j.hlc.2012.01.005. PubMed PMID: 22386613.

1059. Marcos-Garces V, Santas E, Pellicer M, Ruiz-Granell R, Chorro FJ. Unusual Hydatid Cyst-Like Images Caused by Staphylococcus Lugdunensis Infective Endocarditis. Heart Lung Circ. 2019;28(2):e16-e8. Epub 2018/08/22. doi: 10.1016/j.hlc.2018.07.008. PubMed PMID: 30126792.

1060. Roy PM, Khanna S, Mehta Y, Khan AZ. Aspergilloma of the Lung: Strategy to Prevent Endobronchial Spillage. Innovations (Phila). 2016;11(5):373-5. Epub 2016/11/10. doi: 10.1097/imi.0000000000000304. PubMed PMID: 27828805.

1061. Mahdavi Gorabi A, Sadat Ravari M, Sanaei MJ, Davaran S, Kesharwani P, Sahebkar A. Immune checkpoint blockade in melanoma: Advantages, shortcomings and emerging roles of the nanoparticles. Int Immunopharmacol. 2022;113(Pt A):109300. Epub 20221017. doi: 10.1016/j.intimp.2022.109300. PubMed PMID: 36252486.

1062. Li Z, Tan J, Liu X, Zhang W, Meng Q, Zhou M, et al. Tibetan patients with hepatic hydatidosis can tolerate hypoxic environment without incident increase of pulmonary hypertension: an echocardiography study. Int J Cardiovasc Imaging. 2020;36(11):2139-44. Epub 20200806. doi: 10.1007/s10554-020-01922-5. PubMed PMID: 32767023.

1063. Truong U, Patel S, Kheyfets V, Dunning J, Fonseca B, Barker AJ, et al. Non-invasive determination by cardiovascular magnetic resonance of right ventricular-vascular coupling in children and adolescents with pulmonary hypertension. J Cardiovasc Magn Reson. 2015;17:81. Epub 2015/09/18. doi: 10.1186/s12968-015-0186-1. PubMed PMID: 26376972; PubMed Central PMCID: PMCPMC4574020.

1064. Zia ur R, Alvi AR, Bibi S. Hepatic vein and inferior vena caval thrombus extending into the right atrium: a rare complication of amoebic liver abscess. J Coll Physicians Surg Pak. 2010;20(1):57-9. Epub 2010/02/10. doi: 01.2010/jcpsp.5759. PubMed PMID: 20141696.

1065. Rawat S, Kumar R, Raja J, Singh RS, Thingnam SKS. Pulmonary hydatid cyst: Review of literature. J Family Med Prim Care. 2019;8(9):2774-8. Epub 20190930. doi: 10.4103/jfmpc.jfmpc_624_19. PubMed PMID: 31681642; PubMed Central PMCID: PMCPMC6820383.

1066. Chachques JC, Grandjean PA, Pfeffer TA, Perier P, Dreyfus G, Jebara V, et al. Cardiac assistance by atrial or ventricular cardiomyoplasty. J Heart Transplant. 1990;9(3 Pt 1):239-51. Epub 1990/05/01. PubMed PMID: 2355276.

1067. Nakra T, Chandrashekhara SH, Rajashekar P, Ray R, Arava S. Intracardiac Teratoma in a Child: A Rare Site of a Common Tumor. J Pediatr Hematol Oncol. 2021;43(5):e697-e701. doi: 10.1097/mph.0000000000001929. PubMed PMID: 32925407.

1068. Gupta A, Gulati GS, Hote MP, Ray R, Bahl VK, Sharma S. Cavitating atrial myxoma mimicking hydatid cyst on echocardiography: utility of cardiac magnetic resonance imaging and computed tomography for diagnosis and preoperative evaluation. J Thorac Imaging. 2010;25(3):W85-8. Epub 2010/05/07. doi: 10.1097/RTI.0b013e3181c1ad24. PubMed PMID: 20445463.

1069. Bauer H, Pichlmaier H, Ott E, Klovekorn WP, Sunder-Plassmann L, Messmer K. [Autotransfusion through acute, preoperative hemodilution --1st clinical experiences]. Langenbecks Arch Chir. 1974;Suppl:185-9. Epub 1974/01/01. PubMed PMID: 4464408.

1070. Kim E, Choi SW, Min D, Kim SH, Yang WI, Moon JY, et al. A case of a resected benign myxoma-like hemorrhagic cyst, which later recurred as undifferentiated pleomorphic sarcoma in the left atrium. Medicine (Baltimore). 2017;96(16):e6353. Epub 2017/04/20. doi: 10.1097/md.0000000000006353. PubMed PMID: 28422827; PubMed Central PMCID: PMCPMC5406043.

1071. Tuxun T, Apaer S, Yao G, Wang Z, Gu S, Zeng Q, et al. Atrial reconstruction, distal gastrectomy with Ante-situm liver resection and autotransplantation for hepatocellular carcinoma with atrial tumor thrombus: A case report. Medicine (Baltimore). 2021;100(19):e25780. doi: 10.1097/md.0000000000025780. PubMed PMID: 34106611; PubMed Central PMCID: PMCPMC8133267.

1072. Eberly MD, Soh EK, Bannister SP, Tavaf-Motamen H, Scott JS. Isolated cardiac cysticercosis in an adolescent. Pediatr Infect Dis J. 2008;27(4):369-71. Epub 2008/03/05. doi: 10.1097/INF.0b013e318163d316. PubMed PMID: 18316979.

1073. Mercier O, Fadel E, Mussot S, Fabre D, Ladurie FL, Angel C, et al. [Surgical treatment of chronic thromboembolic pulmonary hypertension]. Presse Med. 2014;43(9):994-1007. Epub 2014/08/27. doi: 10.1016/j.lpm.2014.07.007. PubMed PMID: 25154908.

1074. Caruso S, Marrone G, Gentile G. Case 305: Loeffler Endocarditis. Radiology. 2022;304(3):736-42. doi: 10.1148/radiol.210453. PubMed PMID: 35994399.

1075. Caruso S, Marrone G, Gentile G. Case 305. Radiology. 2022;303(2):477-9. doi: 10.1148/radiol.210452. PubMed PMID: 35468018.

1076. Arcas Bellas JJ, Gracia Martinez JL, Cuarental Garcia A, Fajardo Perez MI. [Intrathoracic gossypiboma: a case report]. Rev Esp Anestesiol Reanim. 2009;56(1):54-6. Epub 2009/03/17. PubMed PMID: 19284133.

1077. Lainez B, Ruiz V, Berjon J, Lezaun R. Purulent pericarditis complicated by cardiac tamponade secondary to a hydatid cyst-associated hepatic abscess. Rev Esp Cardiol. 2009;62(8):948-9. Epub 2009/08/27. PubMed PMID: 19706257.

1078. Dartevelle P, Fadel E, Mussot S, Le Roy Ladurie F. [Surgical treatment of pulmonary arterial hypertension]. Rev Prat. 2008;58(18):2031-5. Epub 2009/01/16. PubMed PMID: 19143275.

1079. Bersohn I, Purves LR, Geddes EW. Liver-function tests in primary cancer of the liver in the Bantu. S Afr Med J. 1969;43(40):1219-25. Epub 1969/10/04. PubMed PMID: 4310889.

1080. Yu XY, Wang Y, Zhong H, Dou QL, Song YL, Wen H. Diagnostic value of serum procalcitonin in solid organ transplant recipients: a systematic review and meta-analysis. Transplant Proc. 2014;46(1):26-32. Epub 2014/02/11. doi: 10.1016/j.transproceed.2013.07.074. PubMed PMID: 24507021.

1081. Azad S, Dutta N, Roy Chowdhuri K, Ramman TR, Chandra N, Radhakrishnan S, et al. Atypical Left Ventricular Myxoma: Unusual Echocardiographic and Histopathological Features. World J Pediatr Congenit Heart Surg. 2018:2150135117742626. Epub 2018/03/07. doi: 10.1177/2150135117742626. PubMed PMID: 29506452.

1082. Azad S, Dutta N, Roy Chowdhuri K, Ramman TR, Chandra N, Radhakrishnan S, et al. Atypical Left Ventricular Myxoma: Unusual Echocardiographic and Histopathological Features. World J Pediatr Congenit Heart Surg. 2020;11(4):Np129-np31. Epub 20180305. doi: 10.1177/2150135117742626. PubMed PMID: 29506452.

1083. Boner A, Siegenthaler W. [Correlation between Na2-35SO4 metabolism, age and defined disease pictures]. Z Klin Chem Klin Biochem. 1969;7(3):299-300. Epub 1969/05/01. PubMed PMID: 5356497.

1084. Boner A, Siegenthaler W. [Simultaneous determination of different body fluid spaces]. Z Klin Chem Klin Biochem. 1969;7(3):293-8. Epub 1969/05/01. PubMed PMID: 4901729.

1085. Cabrera PA, Irabedra P, Orlando D, Rista L, Haran G, Vinals G, et al. National prevalence of larval echinococcosis in sheep in slaughtering plants Ovis aries as an indicator in control programmes in Uruguay. Acta Trop. 2003;85(2):281-5. Epub 2003/02/28. PubMed PMID: 12606107.

1086. Getaw A, Beyene D, Ayana D, Megersa B, Abunna F. Hydatidosis: prevalence and its economic importance in ruminants slaughtered at Adama municipal abattoir, Central Oromia, Ethiopia. Acta Trop. 2010;113(3):221-5. Epub 2009/11/04. doi: 10.1016/j.actatropica.2009.10.019. PubMed PMID: 19883622.

1087. D'Alessandro A, Rausch RL, Morales GA, Collet S, Angel D. Echinococcus infections in Colombian animals. Am J Trop Med Hyg. 1981;30(6):1263-76. Epub 1981/11/01. doi: 10.4269/ajtmh.1981.30.1263. PubMed PMID: 7325284.

1088. Inceboz T, Mavi A, Capa Kaya G, Korkmaz M, Goktay Y, Yilmaz O, et al. The ability of 67Ga scintigraphy to detect the lesions of Echinococcus multilocularis infection: preliminary results. Ann Nucl Med. 2006;20(5):345-8. Epub 2006/08/02. PubMed PMID: 16878706.

1089. Alsulami M. Prevalence and histopathological study on cystic hydatidosis in heart and spleen of goat slaughtered at Makkah, Saudi Arabia. Ann Parasitol. 2019;65(3):225-36. doi: 10.17420/ap6503.204. PubMed PMID: 31578846.

1090. Tabatabai M, Ismaili MH, Sami M, Fardin R, Kadivar R. Effect of ovine hydatid cyst fluid on the cardiovascular and respiratory systems in sheep. Ann Parasitol Hum Comp. 1975;50(1):7-15. Epub 1975/01/01. PubMed PMID: 1099969.

1091. Gusbi AM, Awan MA, Beesley WN. Echinococcosis in Libya. II. Prevalence of hydatidosis (Echinococcus granulosus) in sheep. Ann Trop Med Parasitol. 1987;81(1):35-41. Epub 1987/02/01. PubMed PMID: 3675041.

1092. Gusbi AM, Awan MA, Beesley WN. Experimental infection of Libyan sheep with Echinococcus granulosus. Ann Trop Med Parasitol. 1991;85(4):433-7. Epub 1991/08/01. doi: 10.1080/00034983.1991.11812588. PubMed PMID: 1796885.

1093. Gregory GG, McConnell JD. The toxicity and efficiency of arecoline hydrobromide in the Tasmanian hydatid control program. Aust Vet J. 1978;54(4):193-5. Epub 1978/04/01. doi: 10.1111/j.1751-0813.1978.tb02449.x. PubMed PMID: 687277.

1094. Ai Erken MH, Zhao JM, Guan XY, Wen H, Wang YH. Immune responses on allograft heart transplantation in inbred rats infected with Echinococcosis multilocularis. Chin Med J (Engl). 2012;125(24):4412-7. Epub 2012/12/21. PubMed PMID: 23253711.

1095. Tabatabai M, Boroomand K, Gettner S, Nazarian I. Respiratory and cardiovascular responses resulting from intravenous injection of sheep hydatid cyst fluid to dogs. Exp Parasitol. 1973;34(1):12-21. Epub 1973/08/01. doi: 10.1016/0014-4894(73)90057-x. PubMed PMID: 4578948.

1096. Khan J, Basharat N, Khan S, Jamal SM, Rahman SU, Shah AA, et al. Prevalence and Molecular Characterization of Cystic Echinococcosis in Livestock Population of the Malakand Division, Khyber Pakhtunkhwa, Pakistan. Front Vet Sci. 2021;8:757800. Epub 20211021. doi: 10.3389/fvets.2021.757800. PubMed PMID: 34746289; PubMed Central PMCID: PMCPMC8567086.

1097. Islam AW. The prevalence of hydatid cysts in slaughtered cattle in Bangladesh. J Helminthol. 1982;56(3):247-50. Epub 1982/09/01. doi: 10.1017/s0022149x00034611. PubMed PMID: 7175164.

1098. Macpherson CN, Else JE, Suleman M. Experimental infection of the baboon (Papio cynocephalus) with Echinococcus granulosus of camel, cattle, sheep and goat origin from Kenya. J Helminthol. 1986;60(3):213-7. Epub 1986/09/01. doi: 10.1017/s0022149x00026122. PubMed PMID: 3745875.

1099. Molla D, Nazir S, Mohammed A, Tintagu T. Parasitic infections as major cause of abattoir condemnations in cattle slaughtered at an Ethiopian abattoir: 10-year retrospective study. J Helminthol. 2019:1-7. Epub 2019/02/08. doi: 10.1017/s0022149x1900004x. PubMed PMID: 30729895.

1100. Singh BP, Deorani VP, Srivastava VK. Prevalence of hydatid in buffaloes in India and report of a severe liver infection. J Helminthol. 1988;62(2):124-6. Epub 1988/06/01. doi: 10.1017/s0022149x00011354. PubMed PMID: 3397513.

1101. Modabbernia G, Meshgi B, Eslami A. Diversity and burden of helminthiasis in wild ruminants in Iran. J Parasit Dis. 2021;45(2):394-9. Epub 20201115. doi: 10.1007/s12639-020-01314-5. PubMed PMID: 34295038; PubMed Central PMCID: PMCPMC8254833.

1102. Guduro GG, Desta AH. Cyst Viability and Economic Significance of Hydatidosis in Southern Ethiopia. J Parasitol Res. 2019;2019:2038628. Epub 20190801. doi: 10.1155/2019/2038628. PubMed PMID: 31467689; PubMed Central PMCID: PMCPMC6699347.

1103. Mitchell JR. An abattoir survey of helminths in cattle in Swaziland. J S Afr Vet Assoc. 1977;48(1):53-4. Epub 1977/03/01. PubMed PMID: 874949.

1104. Chiou MT, Wang FI, Chang PH, Liu CH, Jeng CR, Cheng CH, et al. Hydatidosis in a Chapman's zebra (Equus burchelli antiquorum). J Vet Diagn Invest. 2001;13(6):534-7. Epub 2001/11/29. doi: 10.1177/104063870101300615. PubMed PMID: 11724148.

1105. Umur S. Prevalence and economic importance of cystic echinococcosis in slaughtered ruminants in Burdur, Turkey. J Vet Med B Infect Dis Vet Public Health. 2003;50(5):247-52. Epub 2003/07/17. PubMed PMID: 12864901.

1106. Tharwat M. Ultrasonography of the liver in healthy and diseased camels (Camelus dromedaries). J Vet Med Sci. 2020;82(4):399-407. Epub 20200226. doi: 10.1292/jvms.19-0690. PubMed PMID: 32101826; PubMed Central PMCID: PMCPMC7192722.

1107. Morales GA, Guzman VH, Wells EA, Angel D. Polycystic echinococcosis in Colombia: the larval cestodes in infected rodents. J Wildl Dis. 1979;15(3):421-8. Epub 1979/07/01. doi: 10.7589/0090-3558-15.3.421. PubMed PMID: 501848.

1108. Orihara M, Obayashi M. A case of cardiac multilocular echinococcosis in a gerbil. Jpn J Vet Res. 1967;15(2):56-7. Epub 1967/06/01. PubMed PMID: 5299529.

1109. Assefa H, Mulate B, Nazir S, Alemayehu A. Cystic echinococcosis amongst small ruminants and humans in central Ethiopia. Onderstepoort J Vet Res. 2015;82(1):E1-7. Epub 2015/08/26. doi: 10.4102/ojvr.v82i1.949. PubMed PMID: 26304166; PubMed Central PMCID: PMCPMC6238683.

1110. Tolosa T, Tigre W, Teka G, Dorny P. Prevalence of bovine cysticercosis and hydatidosis in Jimma municipal abattoir, South West Ethiopia. Onderstepoort J Vet Res. 2009;76(3):323-6. Epub 2009/09/01. PubMed PMID: 21105600.

1111. Tabatabai M, Farrokhsiar M, Cohanim N. Effects of ovine hydatid cyst fluid in sheep before and after treatment with hydrocortisone, antihistamines and atropine. Pahlavi Med J. 1978;9(3):260-76. Epub 1978/07/01. PubMed PMID: 30932.

1112. Beyene T, Hiko A. Zoonotic metacestodes and associated financial loss from cattle slaughtered at Yabello municipal abattoir, Borana-Oromia, Ethiopia. Parasite Epidemiol Control. 2019;5:e00096. Epub 20190223. doi: 10.1016/j.parepi.2019.e00096. PubMed PMID: 30891507; PubMed Central PMCID: PMCPMC6406629.

1113. Atayi Z, Borji H, Moazeni M, Saboor Darbandi M, Heidarpour M. Zataria multiflora would attenuate the hepatotoxicity of long-term albendazole treatment in mice with cystic echinococcosis. Parasitol Int. 2018;67(2):184-7. Epub 2017/12/03. doi: 10.1016/j.parint.2017.11.007. PubMed PMID: 29196023.

1114. Wilson CS, Jenkins DJ, Brookes VJ, Barnes TS. An eight-year retrospective study of hydatid disease (Echinococcus granulosus sensu stricto) in beef cattle slaughtered at an Australian abattoir. Prev Vet Med. 2019;173:104806. Epub 20191019. doi: 10.1016/j.prevetmed.2019.104806. PubMed PMID: 31704561.

1115. Yibar A, Selcuk O, Senlik B. Major causes of organ/carcass condemnation and financial loss estimation in animals slaughtered at two abattoirs in Bursa Province, Turkey. Prev Vet Med. 2015;118(1):28-35. Epub 2014/12/08. doi: 10.1016/j.prevetmed.2014.11.012. PubMed PMID: 25481623.

1116. Panziera W, Bianchi MV, Vielmo A, Bianchi RM, Pavarini SP, Sonne L, et al. Atypical parasitic lesions in slaughtered cattle in Southern Brazil. Rev Bras Parasitol Vet. 2020;29(3):e001720. Epub 20200710. doi: 10.1590/s1984-29612020043. PubMed PMID: 32667499.

1117. Sidler X, Eichhorn J, Geiser V, Burgi E, Schupbach G, Overesch G, et al. [Lung and pleural lesions before and after implementation of a national eradication program against enzootic pneumonia and actinobacillosis as well as changes of slaughter carcass organs in slaughter pigs in Switzerland]. Schweiz Arch Tierheilkd. 2015;157(12):665-73. Epub 2016/02/20. doi: 10.17236/sat00044. PubMed PMID: 26891572.

1118. Negash K, Beyene D, Kumsa B. Cystic echinococcosis in cattle slaughtered at Shashemanne Municipal Abattoir, south central Oromia, Ethiopia: prevalence, cyst distribution and fertility. Trans R Soc Trop Med Hyg. 2013;107(4):229-34. Epub 2013/02/15. doi: 10.1093/trstmh/trt003. PubMed PMID: 23407344.

1119. Al-Kitani F, Baqir S, Hussain MH, Roberts D. Cystic hydatidosis in slaughtered goats from various municipal abattoirs in Oman. Trop Anim Health Prod. 2014;46(8):1357-62. Epub 2014/08/07. doi: 10.1007/s11250-014-0646-x. PubMed PMID: 25096054.

1120. Berhe G. Abattoir survey on cattle hydatidosis in Tigray Region of Ethiopia. Trop Anim Health Prod. 2009;41(7):1347-52. Epub 2009/03/03. doi: 10.1007/s11250-009-9320-0. PubMed PMID: 19252999.

1121. Kebede N, Mitiku A, Tilahun G. Hydatidosis of slaughtered animals in Bahir Dar Abattoir, Northwestern Ethiopia. Trop Anim Health Prod. 2009;41(1):43-50. Epub 2008/12/05. doi: 10.1007/s11250-008-9152-3. PubMed PMID: 19052901.

1122. Kebede W, Hagos A, Girma Z, Lobago F. Echinococcosis/hydatidosis: its prevalence, economic and public health significance in Tigray region, North Ethiopia. Trop Anim Health Prod. 2009;41(6):865-71. Epub 2008/11/13. doi: 10.1007/s11250-008-9264-9. PubMed PMID: 19002598.

1123. Nonga HE, Karimuribo ED. A retrospective survey of hydatidosis in livestock in Arusha, Tanzania, based on abattoir data during 2005-2007. Trop Anim Health Prod. 2009;41(7):1253-7. Epub 2009/02/03. doi: 10.1007/s11250-009-9308-9. PubMed PMID: 19184507.

1124. Regassa F, Molla A, Bekele J. Study on the prevalence of cystic hydatidosis and its economic significance in cattle slaughtered at Hawassa Municipal abattoir, Ethiopia. Trop Anim Health Prod. 2010;42(5):977-84. Epub 2010/01/30. doi: 10.1007/s11250-009-9517-2. PubMed PMID: 20111997.

1125. Sinha BP, Verma BB, Ray SK. Multiple hydatids in the heart of an Indian buffalo (Bubalus bubalus). Trop Anim Health Prod. 1977;9(1):18. Epub 1977/02/01. doi: 10.1007/bf02297383. PubMed PMID: 906084.

1126. Sissay MM, Uggla A, Waller PJ. Prevalence and seasonal incidence of larval and adult cestode infections of sheep and goats in eastern Ethiopia. Trop Anim Health Prod. 2008;40(6):387-94. Epub 2008/06/26. doi: 10.1007/s11250-007-9096-z. PubMed PMID: 18575964.

1127. Alsaadawi MA, Al-Safar AH, Al-Yasari AM, Hussein HM, Allawi AH, Alsalih NJ, et al. Hematological and histopathological changes of rat's hearts experimentally infected with protoscoleces. Trop Biomed. 2022;39(1):117-25. doi: 10.47665/tb.39.1.014. PubMed PMID: 35507934.

1128. Oryan A, Goorgipour S, Moazeni M, Shirian S. Abattoir prevalence, organ distribution, public health and economic importance of major metacestodes in sheep, goats and cattle in Fars, southern Iran. Trop Biomed. 2012;29(3):349-59. Epub 2012/09/29. PubMed PMID: 23018497.

1129. Kose M, Sevimli FK. Prevalence of cystic echinococcosis in slaughtered cattle in Afyonkarahisar. Turkiye Parazitol Derg. 2008;32(1):27-30. Epub 2008/03/21. PubMed PMID: 18351547.

1130. Balbinotti H, Santos GB, Badaraco J, Arend AC, Graichen DA, Haag KL, et al. Echinococcus ortleppi (G5) and Echinococcus granulosus sensu stricto (G1) loads in cattle from Southern Brazil. Vet Parasitol. 2012;188(3-4):255-60. Epub 2012/05/11. doi: 10.1016/j.vetpar.2012.04.007. PubMed PMID: 22571833.

1131. Simsek S, Balkaya I, Koroglu E. Epidemiological survey and molecular characterization of Echinococcus granulosus in cattle in an endemic area of eastern Turkey. Vet Parasitol. 2010;172(3-4):347-9. Epub 2010/06/15. doi: 10.1016/j.vetpar.2010.05.016. PubMed PMID: 20542381.

1132. Kiresi DA, Karabacakoglu A, Odev K, Karakose S. Uncommon locations of hydatid cysts. Acta Radiol. 2003;44(6):622-36. Epub 2003/11/18. PubMed PMID: 14616207.

1133. Mayer DA, Fried B. Aspects of human parasites in which surgical intervention may be important. Adv Parasitol. 2002;51:1-94. Epub 2002/09/20. PubMed PMID: 12238889.

1134. Beggs I. The radiology of hydatid disease. AJR Am J Roentgenol. 1985;145(3):639-48. Epub 1985/09/01. doi: 10.2214/ajr.145.3.639. PubMed PMID: 3895873.

1135. Dursun M, Terzibasioglu E, Yilmaz R, Cekrezi B, Olgar S, Nisli K, et al. Cardiac hydatid disease: CT and MRI findings. AJR Am J Roentgenol. 2008;190(1):226-32. Epub 2007/12/21. doi: 10.2214/ajr.07.2035. PubMed PMID: 18094316.

1136. Akar R, Eryilmaz S, Yazicioglu L, Eren NT, Durdu S, Uysalel A, et al. Surgery for cardiac hydatid disease: an Anatolian experience. Anadolu Kardiyol Derg. 2003;3(3):238-44. Epub 2003/09/12. PubMed PMID: 12967892.

1137. Khiari A, Fabre JM, Mzali R, Domergue J, Beyrouti MI. [Unusual locations of hydatid cysts]. Ann Gastroenterol Hepatol (Paris). 1995;31(5):295-305. Epub 1995/10/01. PubMed PMID: 8572565.

1138. Vicente E, Quijano Y, Ielpo B, De Luca GM, Prestera A, Duran H, et al. Cavoatrial thrombectomy without the use of cardiopulmonary bypass for abdominal tumors. Our experience and state of the art. Ann Vasc Surg. 2015;29(5):1020.e1-5. Epub 2015/03/17. doi: 10.1016/j.avsg.2015.01.021. PubMed PMID: 25770387.

1139. Ciampi Roti A. [Primary echinococcosis of the myocardium]. Arch De Vecchi Anat Patol. 1979;63(3):435-80. Epub 1979/03/01. PubMed PMID: 162435.

1140. Cacoub P, Chapoutot L, Du-Boutin LT, Derbel A, Gandjbakhch I, Sal R, et al. [Hydatid cyst of the interventricular septum. Contribution of magnetic resonance imaging]. Arch Mal Coeur Vaiss. 1991;84(12):1857-60. Epub 1991/12/01. PubMed PMID: 1793324.

1141. Abhishek V, Avinash V. Cardiac hydatid disease: literature review. Asian Cardiovasc Thorac Ann. 2012;20(6):747-50. doi: 10.1177/0218492312460774. PubMed PMID: 23284134.

1142. Emlik D, Kiresi D, Sunam GS, Kivrak AS, Ceran S, Odev K. Intrathoracic extrapulmonary hydatid disease: radiologic manifestations. Can Assoc Radiol J. 2010;61(3):170-6. Epub 2010/01/30. doi: 10.1016/j.carj.2009.12.002. PubMed PMID: 20110156.

1143. Turgut M, Bayulkem K. Cerebrovascular occlusive disease: hydatidosis. Childs Nerv Syst. 1998;14(12):697-9. Epub 1999/01/09. doi: 10.1007/s003810050300. PubMed PMID: 9881621.

1144. Franco-Paredes C, Rouphael N, Mendez J, Folch E, Rodriguez-Morales AJ, Santos JI, et al. Cardiac manifestations of parasitic infections part 3: pericardial and miscellaneous cardiopulmonary manifestations. Clin Cardiol. 2007;30(6):277-80. Epub 2007/06/07. doi: 10.1002/clc.20092. PubMed PMID: 17551959; PubMed Central PMCID: PMCPMC6653132.

1145. Freundlich IM, Lind TA. Calcification of the heart and great vessels. CRC Crit Rev Clin Radiol Nucl Med. 1975;6(2):171-216. Epub 1975/04/01. PubMed PMID: 238789.

1146. Taiana JA. Thoracic hydatid echinococcosis; diagnosis and treatment. Dis Chest. 1966;49(1):8-14. Epub 1966/01/01. doi: 10.1378/chest.49.1.8. PubMed PMID: 5907808.

1147. Kahlfuss S, Flieger RR, Roepke TK, Yilmaz K. Diagnosis and treatment of cardiac echinococcosis. Heart. 2016;102(17):1348-53. Epub 2016/05/21. doi: 10.1136/heartjnl-2016-309350. PubMed PMID: 27199228.

1148. Nunes MC, Guimaraes Junior MH, Diamantino AC, Gelape CL, Ferrari TC. Cardiac manifestations of parasitic diseases. Heart. 2017;103(9):651-8. Epub 2017/03/13. doi: 10.1136/heartjnl-2016-309870. PubMed PMID: 28285268.

1149. Fennira S, Kamoun S, Besbes B, Ben Mrad I, Zairi I, Ben Moussa F, et al. Cardiac hydatid cyst in the interventricular septum: A literature review. Int J Infect Dis. 2019;88:120-6. Epub 20190906. doi: 10.1016/j.ijid.2019.09.004. PubMed PMID: 31499211.

1150. Munzer D. New perspectives in the diagnosis of Echinococcus disease. J Clin Gastroenterol. 1991;13(4):415-23. Epub 1991/08/01. doi: 10.1097/00004836-199108000-00011. PubMed PMID: 1918847.

1151. Imani F, Ajana A, Dafiri R, Benamour H, Zakari S, Fathi K, et al. [Pulmonary hydatidosis. Contribution of x-ray computed tomography]. J Radiol. 1988;69(8-9):489-94. Epub 1988/08/01. PubMed PMID: 3057182.

1152. Kilic D, Tercan F, Sahin E, Bilen A, Hatipoglu A. Unusual radiologic manifestations of the echinococcus infection in the thorax. J Thorac Imaging. 2006;21(1):32-6. Epub 2006/03/16. doi: 10.1097/01.rti.0000186994.92705.2d. PubMed PMID: 16538153.

1153. Yuksel M, Demirpolat G, Sever A, Bakaris S, Bulbuloglu E, Elmas N. Hydatid disease involving some rare locations in the body: a pictorial essay. Korean J Radiol. 2007;8(6):531-40. Epub 2007/12/12. doi: 10.3348/kjr.2007.8.6.531. PubMed PMID: 18071284; PubMed Central PMCID: PMCPMC2627456.

1154. Valdovinos Mahave MC, Lapuente Gonzalez MP, Munoz Marco J, Aliacar Munoz M. [Anaphylaxis due to the rupture of a hydatid cyst in the right ventricle]. Med Clin (Barc). 1997;108(6):238-9. Epub 1997/02/15. PubMed PMID: 9102497.

1155. Slim MS, Akel SR. Hydatidosis in childhood. Prog Pediatr Surg. 1982;15:119-29. Epub 1982/01/01. PubMed PMID: 7146431.

1156. Zalaquett E, Menias C, Garrido F, Vargas M, Olivares JF, Campos D, et al. Imaging of Hydatid Disease with a Focus on Extrahepatic Involvement. Radiographics. 2017;37(3):901-23. Epub 2017/05/12. doi: 10.1148/rg.2017160172. PubMed PMID: 28493801.

1157. Hilger HH, Carstens V, Behrenbeck DW. [The differential diagnosis of heart failure (author's transl)]. Radiologe. 1979;19(2):43-50. Epub 1979/02/01. PubMed PMID: 368878.

1158. Ruiz-Nodar JM, Iturralde E, Aguilar R, Caniego JL, Martinez de la Concha L, Martinez Elbal L. [The rupture of a cardiac hydatid cyst located in the right ventricle]. Rev Esp Cardiol. 1995;48(8):563-5. Epub 1995/08/01. PubMed PMID: 7644812.

1159. Elbiaze M. [Thoracic hydatid cysts: news]. Rev Mal Respir. 2006;23(4 Pt 2):10s80-10s82. Epub 2006/11/28. PubMed PMID: 17127973.

1160. Thameur H, Chenik S, Abdelmoulah S, Bey M, Hachicha S, Chemingui M, et al. [Thoracic hydatidosis. A review of 1619 cases]. Rev Pneumol Clin. 2000;56(1):7-15. Epub 2000/03/31. PubMed PMID: 10740109.

1161. Thameur H, Abdelmoula S, Chenik S, Bey M, Ziadi M, Mestiri T, et al. Cardiopericardial hydatid cysts. World J Surg. 2001;25(1):58-67. Epub 2001/02/24. doi: 10.1007/s002680020008. PubMed PMID: 11213157.

1162. Arvinti PC, Rosca G. [Multiple echinococcosis with predominant cardiac localization as the cause of death in sudden deaths]. Acta Med Leg Soc (Liege). 1986;36(1):1-9. Epub 1986/01/01. PubMed PMID: 2979138.

1163. McManus D. Hydatid disease. Am J Trop Med Hyg. 1995;53(6):575-6. Epub 1995/12/01. PubMed PMID: 8561255.

1164. Wiwanitkit V. Cardiac hydatid cyst: a comment/ Cardiac hydatid cyst case recovered with medical treatment. Anadolu Kardiyol Derg. 2012;12(4):368; author reply Epub 2012/04/14. doi: 10.5152/akd.2012.107. PubMed PMID: 22498864.

1165. Alami B, Boujraf S, Alaoui-Lamrani Y, Boubbou M, Maaroufi M. Hydatid cyst in interventricular septum. Ann Card Anaesth. 2019;22(3):343-4. doi: 10.4103/aca.ACA_183_18. PubMed PMID: 31274505; PubMed Central PMCID: PMCPMC6639872.

1166. Losanoff JE, Richman BW, Jones JW. Cardiac hydatid cysts. Ann Thorac Surg. 2002;73(2):699-700. Epub 2002/02/16. doi: 10.1016/s0003-4975(01)03352-5. PubMed PMID: 11845918.

1167. Ozyazicioglu A, Kocak H, Becit N. Cardiac and pericardiac echinococcosis. Ann Thorac Surg. 2000;70(3):1002-4. Epub 2000/10/04. doi: 10.1016/s0003-4975(00)01730-6. PubMed PMID: 11016362.

1168. Umut S, Tosun CA, Mihmanh A. Hydatidosis with pericardial involvement. Chest. 1992;102(6):1916-7. Epub 1992/12/01. doi: 10.1378/chest.102.6.1916d. PubMed PMID: 1446531.

1169. Sirikci A, Kervancioglu R, Bayram A, Ozkur A, Kervancioglu S, Bayram M. A giant cardiac hydatid cyst located in the left ventricle of the heart: MR imaging features. Eur Radiol. 2000;10(12):1993-4. Epub 2001/04/18. doi: 10.1007/pl00022809. PubMed PMID: 11305585.

1170. Karuppiah S, Dharmalingam S, Sahajanandan R, George G. Role of transoesophageal echocardiography in peri-operative management of cardiac hydatid cyst. Indian J Anaesth. 2015;59(11):753-4. Epub 2016/01/13. doi: 10.4103/0019-5049.170039. PubMed PMID: 26755844; PubMed Central PMCID: PMCPMC4697251.

1171. Mestrovic T, Sviben M. eComment. New horizons in diagnosing and managing cardiac echinococcosis. Interact Cardiovasc Thorac Surg. 2018;26(2):359. Epub 2018/01/24. doi: 10.1093/icvts/ivx359. PubMed PMID: 29361169.

1172. Dost B, Kartal C, Baris S, Karakaya D. Cardiac Hydatid Cyst Diagnosed Incidentally by Transesophageal Echocardiography After Cardiac Arrest. J Cardiothorac Vasc Anesth. 2022;36(1):344. Epub 20210514. doi: 10.1053/j.jvca.2021.05.009. PubMed PMID: 34103215.

1173. Saidi F. Images in clinical medicine: echinococcal cyst of the pericardium. N Engl J Med. 1995;333(23):1572. Epub 1995/12/07. doi: 10.1056/nejm199512073332317. PubMed PMID: 7477190.

1174. Cosma E, Georgescu T, Chirileanu T, Ghibu M, Georgescu R. [Letter: Embolism of the aortic bifurcation by 2 mobilized cardiac hydatid cyst]. Nouv Presse Med. 1975;4(5):353. Epub 1975/02/01. PubMed PMID: 1129088.

1175. Saimot AG. [Other localizations of hydatidosis]. Rev Prat. 1978;28(37):2887-94. Epub 1978/08/01. PubMed PMID: 705198.

1176. Akar AR. Cardiac echinococcosis: A rare but challenging surgical entity. Turk Gogus Kalp Damar Cerrahisi Derg. 2019;27(3):401-2. Epub 20190629. doi: 10.5606/tgkdc.dergisi.2019.03002. PubMed PMID: 32082894; PubMed Central PMCID: PMCPMC7021429.

1177. Reuter S, Buck A, Grebe O, Nussle-Kugele K, Kern P, Manfras BJ. Salvage treatment with amphotericin B in progressive human alveolar echinococcosis. Antimicrob Agents Chemother. 2003;47(11):3586-91. Epub 2003/10/25. doi: 10.1128/aac.47.11.3586-3591.2003. PubMed PMID: 14576122; PubMed Central PMCID: PMCPMC253803.

1178. Leinzinger EP. [Echinococcosis of the heart]. Beitr Gerichtl Med. 1985;43:393-5. Epub 1985/01/01. PubMed PMID: 4051988.

1179. Albayrak Y, Kargi A, Albayrak A, Gelincik I, Cakir YB. Liver alveolar echinococcosis metastasized to the breast. Breast Care (Basel). 2011;6(4):289-91. Epub 2011/12/03. doi: 10.1159/000331314. PubMed PMID: 22135627; PubMed Central PMCID: PMCPMC3225214.

1180. Zhang X, Wei X, Ran L, Tang H. A rare case of cardiac alveolar echinococcosis. Eur Heart J. 2020;41(28):2698. doi: 10.1093/eurheartj/ehaa511. PubMed PMID: 32702740.

1181. Bansal N, Vij V, Rastogi M, Wadhawan M, Kumar A. A report on three patients with Echinococcus multilocularis: Lessons learned. Indian J Gastroenterol. 2018;37(4):353-8. Epub 2018/08/20. doi: 10.1007/s12664-018-0860-y. PubMed PMID: 30121887.

1182. Chernyavskiy A, Alsov S, Guliaeva K, Porshennikov I. The first case of combined heart-liver transplantation in a patient with alveolar echinococcosis. J Card Surg. 2020;35(11):3199-201. Epub 20200813. doi: 10.1111/jocs.14932. PubMed PMID: 32789914.

1183. Etievent JP, Vuitton D, Allemand H, Weill F, Gandjbakhch I, Miguet JP. Pulmonary embolism from a parasitic cardiac clot secondary to hepatic alveolar echinococcosis. J Cardiovasc Surg (Torino). 1986;27(6):671-4. Epub 1986/11/01. PubMed PMID: 3782269.

1184. Grimm J, Krickl J, Beck A, Nell J, Bergmann M, Tappe D, et al. Establishing and evaluation of a polymerase chain reaction for the detection of Echinococcus multilocularis in human tissue. PLoS Negl Trop Dis. 2021;15(2):e0009155. Epub 20210225. doi: 10.1371/journal.pntd.0009155. PubMed PMID: 33630840; PubMed Central PMCID: PMCPMC7906421.

1185. Kantarci M, Bayraktutan U, Karabulut N, Aydinli B, Ogul H, Yuce I, et al. Alveolar echinococcosis: spectrum of findings at cross-sectional imaging. Radiographics. 2012;32(7):2053-70. Epub 2012/11/15. doi: 10.1148/rg.327125708. PubMed PMID: 23150858.

1186. Çelik D, Ödemiş I, Korkmaz M, Köse S. A rare case of cardiac alveolar echinococcosis without any complications for eight years. Rev Esp Quimioter. 2022;35(1):91-4. Epub 20211126. doi: 10.37201/req/082.2021. PubMed PMID: 34823349; PubMed Central PMCID: PMCPMC8790652.

1187. Gaultier JB, Hot A, Mauservey C, Dumortier J, Coppere B, Ninet J. [Granulomatous liver disease as the presenting feature of alveolar echinococcosis in an hepatitis C infected cardiac transplant patient]. Rev Med Interne. 2009;30(9):812-5. Epub 2009/02/06. doi: 10.1016/j.revmed.2008.11.007. PubMed PMID: 19193475.

1188. Guo H, Liu W, Wang J, Xing Y. Extrahepatic alveolar echinococcus on multi-slice computed tomography and magnetic resonance imaging. Sci Rep. 2021;11(1):9409. Epub 20210430. doi: 10.1038/s41598-021-89101-x. PubMed PMID: 33931712; PubMed Central PMCID: PMCPMC8087791.

1189. Yamamoto Y, Sakamoto Y, Kamiyama T, Nagatsu A, Asahi Y, Orimo T, et al. A case of alveolar echinococcosis in the liver that ruptured into the pericardium treated by a combination of hepatectomy and albendazole. Surg Case Rep. 2022;8(1):63. Epub 20220408. doi: 10.1186/s40792-022-01417-6. PubMed PMID: 35394220; PubMed Central PMCID: PMCPMC8993950.

1190. Ibanez Esquembre V, Sola R, Pelayo M. [Multiple hydatid embolism: acute ischemia of the right lower limb. Apropos of a case]. Angiologia. 1990;42(3):89-94. Epub 1990/05/01. PubMed PMID: 2393164.

1191. Delaye A, Metras D, Malmejac C. [Treatment of hydatid cyst of the heart]. Ann Chir Thorac Cardiovasc. 1973;12(3):307-9. Epub 1973/07/01. PubMed PMID: 4782979.

1192. Mechmeche R, Ben Cheikh M, Bousnina A, Gharbi HA, Ben Ismail M. Hydatid cyst of the heart in children. A series of nine cases. Ann Radiol (Paris). 1984;27(2-3):173-81. Epub 1984/02/01. PubMed PMID: 6721424.

1193. Aris A, Leon C, Bonnin JO, Serra C, Caralps JM. One-stage surgical treatment of cardiac and pulmonary echinococcosis. Ann Thorac Surg. 1981;31(6):564-8. Epub 1981/06/01. doi: 10.1016/s0003-4975(10)61350-1. PubMed PMID: 7247549.

1194. Aytac A, Yurdakul Y, Ikizler C, Olga R, Saylam A. Pulmonary hydatid disease: report of 100 patients. Ann Thorac Surg. 1977;23(2):145-51. Epub 1977/02/01. doi: 10.1016/s0003-4975(10)64088-x. PubMed PMID: 836103.

1195. Tellez G, Nojek C, Juffe A, Rufilanchas J, O'Connor F, Figuera D. Cardiac echinococcosis: report of 3 cases and review of the literature. Ann Thorac Surg. 1976;21(5):425-30. Epub 1976/05/01. doi: 10.1016/s0003-4975(10)63892-1. PubMed PMID: 131522.

1196. Shennak MM, Tarawneh MS, Amr SS, Al-Sheikh TM, Abu-Rajab MT, Grec SS. Patterns of hepatomegaly in Jordanians: a prospective study of 800 cases. Ann Trop Med Parasitol. 1985;79(4):443-8. Epub 1985/08/01. doi: 10.1080/00034983.1985.11811943. PubMed PMID: 4073996.

1197. Atias A, Pizarro D, Leiva A, Gomez G, Naquira N. [Intracardiac rupture of a hydatid cyst of the left ventricle, with arterial hydatidic emboli and gangrene of a leg]. Bol Chil Parasitol. 1966;21(4):124-6. Epub 1966/10/01. PubMed PMID: 5990164.

1198. Franquet T, Lecumberri F, Joly M. Hydatid heart disease. Br J Radiol. 1984;57(674):171-3. Epub 1984/02/01. doi: 10.1259/0007-1285-57-674-171. PubMed PMID: 6692090.

1199. Niel G, Pinon JM, Gentilini M. [Immunofluorescence applied to the serologic diagnosis of bilharziasis (apropos of a simple technic of preparation of slices for freezing)]. Bull Soc Pathol Exot Filiales. 1970;63(3):356-62. Epub 1970/05/01. PubMed PMID: 4932716.

1200. Hazan E, Leblanc J, Robillard M, Mathey J. [Hydatid cyst of the right ventricle revealed by an acute complication. Emergency exeresis with prosthetic replacement of the tricuspid valve]. Chirurgie. 1970;96(4):257-60. Epub 1970/01/01. PubMed PMID: 5497423.

1201. Malmejac C, Houel J, Metras D, Pons R. [Hydatid cyst of the heart (apropos of 7 cases)]. Chirurgie. 1970;96(4):261-7. Epub 1970/01/01. PubMed PMID: 5492524.

1202. McClatchie S, Rajpal SK. Sudden death in hydatid disease of the heart. East Afr Med J. 1965;42(12):723-4. Epub 1965/12/01. PubMed PMID: 5861307.

1203. Montoyo JV, Nogues-Antich FJ, Rivas L, Angel J, Soler-Soler J. Case report: left ventricular echinococcosis diagnosed by coronary cineangiography. Eur J Cardiol. 1979;10(3):215-9. Epub 1979/09/01. PubMed PMID: 499281.

1204. Loperfido F, Bonomo L, Bellocci F, Zeppilli P, Fanelli R, Ansalone G, et al. [3 cases of cardiac echinococcosis. Diagnostic considerations]. G Ital Cardiol. 1978;8(3):315-22. Epub 1978/01/01. PubMed PMID: 640314.

1205. Pome G, Quaini E, Vitali E, Panzeri E. Intrapericardial hydatid cyst. Report of a case successfully operated. G Ital Cardiol. 1985;15(10):998-1001. Epub 1985/10/01. PubMed PMID: 4092921.

1206. Victor S, Ravindran P, Joseph AD, Vijayasankar CS, Suniti S. Hydatid cyst of the right ventricle in association with rheumatic mitral stenosis. Indian Heart J. 1982;34(4):256-9. Epub 1982/07/01. PubMed PMID: 7141456.

1207. Balram A, Kaul U, Rama Rao BV, Rao IM, Rajani M, Gopinath N, et al. Echinococcosis of the heart presenting as ventricular tachycardia: recovery after surgical treatment. Indian J Chest Dis Allied Sci. 1983;25(4):286-9. Epub 1983/10/01. PubMed PMID: 6679497.

1208. Singh B, Logani KB. Primary hydatid cyst of the heart (a case report with review of literature). Indian J Pathol Microbiol. 1983;26(4):315-8. Epub 1983/10/01. PubMed PMID: 6674193.

1209. Chapedeiro E, Lopes ER, Mesquita PM. PRIMARY HYDATID CYSTS OF THE HEART. J Trop Med Hyg. 1965;68:9-11. Epub 1965/01/01. PubMed PMID: 14260874.

1210. Niehues B, Heuser L, Recht K, Tauchert M, Dalichau H. [Echinococcosis involvement of the heart]. Leber Magen Darm. 1983;13(1):27-32. Epub 1983/01/01. PubMed PMID: 6843270.

1211. Sala J, Pare C, Abad C, Sanz G, Navarro-Lopez F, Mulet J, et al. [Cardiac echinococcosis]. Med Clin (Barc). 1983;80(3):144-5. Epub 1983/02/05. PubMed PMID: 6834928.

1212. Chin DD, Rice JP, Lequesne DM. Hydatid cyst of the heart presenting as cerebral and cerebellar infarction. Med J Aust. 1981;2(10):556-7. Epub 1981/11/14. PubMed PMID: 7321957.

1213. Diaz P, Maillo A. [Middle cerebral artery occlusion after cerebral hydatic embolism in a pediatric patient: case report]. Neurocirugia (Astur). 2002;13(3):216-8. Epub 2002/08/01. PubMed PMID: 12148166.

1214. Al-Naaman YD, Samarrai AR, Al-Ani HR. Hydatid disease of the heart. Nihon Kyobu Geka Gakkai Zasshi. 1978;26(3):267-75. Epub 1978/03/01. PubMed PMID: 701864.

1215. Fawzy ME. Hydatid disease. Nurs Mirror Midwives J. 1977;144(18):48-9. Epub 1977/05/05. PubMed PMID: 585880.

1216. Vara Lopez R. [Hydatid cysts of the heart]. An R Acad Nac Med (Madr). 1972;89(4):685-706. Epub 1972/01/01. PubMed PMID: 4679033.

1217. Hueto Perez de Heredia J, Perez de las Casas M, Dominguez del Valle J, Vila Mayo E, Urquia Brana M, Gomez Dorronsoro M. [Thoracic hydatidosis. Our experience in the last 15 years]. Rev Clin Esp. 1999;199(1):13-7. Epub 1999/03/25. PubMed PMID: 10089771.

1218. Ramos G, Del Villar JL, Sainz JL, Del Busto EF, Gonzalez E, Ortega J. [Cardiac hydatidosis]. Rev Clin Esp. 1971;121(5):411-24. Epub 1971/06/15. PubMed PMID: 5095846.

1219. Urquia M, Garrido J, de los Arcos E, Aleu M, Suliman G, Frances M, et al. [Hydatidosis of the interventricular septum. Treatment of 9 cases]. Rev Clin Esp. 1975;138(6):521-7. Epub 1975/09/30. PubMed PMID: 1197820.

1220. Villacastin BP, Robledo AG, Perez Gil MA, Franco A, Miro C. [New gamut: cavitated pulmonary nodules and localized deformity of the cardiac region]. Rev Clin Esp. 1992;190(7):367-70. Epub 1992/04/01. PubMed PMID: 1620925.

1221. Arevalo Prieto J, Otaduy Larrea C, Iriarte Ezcurdia MM. [A case of intramyocardial hydatid cyst]. Rev Esp Cardiol. 1965;18(4):552-9. Epub 1965/10/01. PubMed PMID: 5864952.

1222. Armenta J, Duclos F, Zambrano A, Bohorquez A, Jimenez C. [Cardiac hydatidosis with cerebral metastasis. (clinical note)]. Rev Esp Cardiol. 1967;20(1):125-31. Epub 1967/01/01. PubMed PMID: 5598501.

1223. Munoz-Gil J, Chorro FJ, Insa L, Llacer A, Carbonell C, Martinez-Leon J, et al. [Cardiac hydatid cyst. Bidimensional echocardiographic study]. Rev Esp Cardiol. 1984;37(5):375-7. Epub 1984/09/01. PubMed PMID: 6494548.

1224. Bianchi C, Rodriguez R, Saavedra J, Reyes H. [Cardiac hydatidosis]. Rev Med Chil. 1972;100(1):46-8. Epub 1972/01/01. PubMed PMID: 5012092.

1225. Bahr R, Huzly A. [Rare intrapericardial localization of Echinococcus granulosus]. Rofo. 1984;141(1):107-8. Epub 1984/07/01. doi: 10.1055/s-2008-1053096. PubMed PMID: 6431507.

1226. Goebel N, Gander MP. [Echinococcus of the heart. Infundibular pulmonary stenosis due to a large solitary cyst of the ventricular septum (author's transl)]. Rofo. 1977;126(1):11-4. Epub 1977/01/01. doi: 10.1055/s-0029-1230525. PubMed PMID: 137861.

1227. Przybojewski JZ. Primary cardiac hydatid disease. A case report. S Afr Med J. 1984;65(11):438-42. Epub 1984/03/17. PubMed PMID: 6701705.

1228. Przybojewski JZ, Barnard PM, Van der Walt JJ, Botha JA. Unstable angina pectoris secondary to multiple calcified coronary artery masses. Successful treatment with coronary artery bypass surgery. S Afr Med J. 1986;69(11):694-9. Epub 1986/05/24. PubMed PMID: 3486488.

1229. Smedema E, Mayosi BM, Smedema JP. Hydatid disease - the 'water lily' sign. S Afr Med J. 2006;96(10):1042. Epub 2006/12/19. PubMed PMID: 17171836.

1230. Ghannad E, Abbou CB, Nottin R, Hourdebaigt-Larrusse P, Soulie J, Grivaux M. [Cardiac hydatidosis]. Sem Hop. 1983;59(19):1459-63. Epub 1983/05/12. PubMed PMID: 6310761.

1231. Hernigou A, Plainfosse MC, Merran S, Guermonprez JL. [Role of ultrasonics in the diagnosis of 5 hydatid cysts of the heart]. Sem Hop. 1984;60(15):1061-7. Epub 1984/04/05. PubMed PMID: 6326294.

1232. Cheng W. Hydatid cysts in the pericardium- a new case and review of the literature. Thorac Cardiovasc Surg. 1982;30(1):56-7. Epub 1982/02/01. doi: 10.1055/s-2007-1022210. PubMed PMID: 6179237.

1233. Gula G, Luisi VS, Machado F, Yacoub M. Hydatid cyst of the heart. Clinical and surgical implications. Thorac Cardiovasc Surg. 1979;27(6):393-6. Epub 1979/12/01. doi: 10.1055/s-0028-1096284. PubMed PMID: 542947.

1234. Madariaga I, de la Fuente A, Lezaun R, Imizcoz MA, Carmona JR, Urquia M, et al. Cardiac echinococcosis and systemic embolism. Report of a case. Thorac Cardiovasc Surg. 1984;32(1):57-9. Epub 1984/02/01. doi: 10.1055/s-2007-1023346. PubMed PMID: 6198777.

1235. Di Bello R. [Complications provoked by cystic rupture in 15 personal cases of cardiac echinococcosis]. Torax. 1965;14(3):182-3. Epub 1965/09/01. PubMed PMID: 5879714.

1236. Matteucci P, Rubio R, Fiandra O. [Double hydatidosis of the heart]. Torax. 1970;19(3):204-7. Epub 1970/09/01. PubMed PMID: 5502967.

1237. Purriel P, Tomalino D, Acosta Ferreira W, Canoso J. [Hydatid cyst of the right auricular wall, opening into the heart chamber]. Torax. 1965;14(3):187-95. Epub 1965/09/01. PubMed PMID: 5879716.

1238. Sanjines A, Abo JC, Rubio R, Zerbino V. [Cardiac echinococcosis. Our experience on 15 surgical cases]. Torax. 1965;14(3):163-81. Epub 1965/09/01. PubMed PMID: 5879713.

1239. Bousnina A, Bousnina S, Mechmech R, Ben Ismail M. [Hydatid cyst of the heart in children. Apropos of 6 cases]. Tunis Med. 1983;61(1):15-7. Epub 1983/01/01. PubMed PMID: 6680936.

1240. Bousnina S, Tabbane C, Bousnina A, Ben Ismail M. [Hydatid cyst of the heart in a 2-year-old girl]. Tunis Med. 1981;59(6):446-9. Epub 1981/11/01. PubMed PMID: 7348493.

1241. Zalila S, Slimane ML, Labidi S, Ben Naceur M. [Cardiac hydatidosis (apropos of 13 cases)]. Tunis Med. 1986;64(6-7):607-14. Epub 1986/06/01. PubMed PMID: 3787749.

1242. Bouraoui H, Trimeche B, Mahdhaoui A, Majdoub A, Zaaraoui J, Hajri Ernez S, et al. Echinococcosis of the heart: clinical and echocardiographic features in 12 patients. Acta Cardiol. 2005;60(1):39-41. Epub 2005/03/23. doi: 10.2143/ac.60.1.2005047. PubMed PMID: 15779850.

1243. Yilmazer MM, Devrim I, Tavli V. Cardiac hydatid cyst associated with multiple organ involvement. Acta Cardiol. 2011;66(1):87-8. Epub 2011/03/31. doi: 10.2143/ac.66.1.2064973. PubMed PMID: 21446387.

1244. Bashour TT, Alali AR, Mason DT, Saalouke M. Echinococcosis of the heart: clinical and echocardiographic features in 19 patients. Am Heart J. 1996;132(5):1028-30. Epub 1996/11/01. doi: 10.1016/s0002-8703(96)90017-4. PubMed PMID: 8892779.

1245. Koza Y, Tufekcioglu O, Birincioglu L, Okten S, Basar V. A cardiac cyst presented with subacute anterior myocardial infarction. Hydatid cyst. Anadolu Kardiyol Derg. 2013;13(8):820, 4. Epub 2013/11/30. doi: 10.5152/akd.2013.4813. PubMed PMID: 24287368.

1246. Simsek Z, Koza Y, Tas MH, Kaya U, Ates A, Kantarci M. A heart-like cystic image in the heart. Hydatid cyst. Anadolu Kardiyol Derg. 2013;13(5):509-10, 1. Epub 2013/06/04. doi: 10.5152/akd.2013.165. PubMed PMID: 23728270.

1247. Noaman H, Rawaf S, Majeed A, Salmasi AM. Hydatid Cyst of the Heart. Angiology. 2017;68(9):765-8. Epub 2017/02/02. doi: 10.1177/0003319717690093. PubMed PMID: 28142253.

1248. Bakkali A, Jaabari I, Bouhdadi H, Razine R, Bennani Mechita N, El Harrag J, et al. [Cardiac hydatid cyst about 17 operated cases]. Ann Cardiol Angeiol (Paris). 2018;67(2):67-73. Epub 2017/05/31. doi: 10.1016/j.ancard.2017.04.010. PubMed PMID: 28554702.

1249. Jerbi S, Romdhani N, Tarmiz A, Kortas C, Mlika S, Khelil N, et al. [Emboligenous hydatid cyst of the right heart]. Ann Cardiol Angeiol (Paris). 2008;57(1):62-5. Epub 2007/07/17. doi: 10.1016/j.ancard.2007.05.007. PubMed PMID: 17631269.

1250. Mesrati MA, Belhadj M, Aissaoui A, HajSalem N, Oualha D, Boughattas M, et al. [Sudden cardiovascular death in adults: Study of 361 autopsy cases]. Ann Cardiol Angeiol (Paris). 2017;66(1):7-14. Epub 2016/04/26. doi: 10.1016/j.ancard.2016.03.003. PubMed PMID: 27109042.

1251. Mrad Dali K, Tlili K, Ly M, Romdhani N, Bakir D, Gharbi H, et al. [Radioclinical profile of cardiopericardial hydatid: report of 17 cases]. Ann Cardiol Angeiol (Paris). 2000;49(7):414-22. Epub 2003/01/31. PubMed PMID: 12555496.

1252. Orhan G, Ozay B, Tartan Z, Kurc E, Ketenci B, Sargin M, et al. [Surgery of cardiac hydatid cysts. Experience of 39 years]. Ann Cardiol Angeiol (Paris). 2008;57(1):58-61. Epub 2007/06/15. doi: 10.1016/j.ancard.2007.05.003. PubMed PMID: 17568556.

1253. Sabouret P, Pavie A, Bors V, Gandjbakhch I. [Picture of the month. Hydatid cyst of cardiac localization]. Ann Cardiol Angeiol (Paris). 1998;47(10):767. Epub 1999/01/29. PubMed PMID: 9922857.

1254. Chuzel M, Grizard M, Gressier M, Guillot B, Michaud P, Termet H. [Surgical treatment of cardiac hydatidosis. Apropos of 4 left ventricular localizations]. Ann Chir. 1984;38(2):139-40. Epub 1984/03/01. PubMed PMID: 6732129.

1255. Delaye A, Metras D, Malmejac C. [Pathological anatomy and modes of development of hydatid cysts of the heart]. Ann Chir Thorac Cardiovasc. 1973;12(3):245-7. Epub 1973/07/01. PubMed PMID: 4782966.

1256. Delaye A, Metras D, Malmejac C. [Hydatid cyst of the heart; diagnostic and topographic aspects]. Ann Chir Thorac Cardiovasc. 1973;12(3):263-6. Epub 1973/07/01. PubMed PMID: 4782971.

1257. Eser I, Karabag H, Gunay S, Seker A, Cevik M, Ali Sak ZH, et al. Surgical approach for patients with unusually located hydatid cyst. Ann Ital Chir. 2014;85(1):50-5. Epub 2013/12/21. PubMed PMID: 24355801.

1258. Gozubuyuk A, Savasoz B, Gurkok S, Yucel O, Caylak H, Kavakli K, et al. Unusually located thoracic hydatid cysts. Ann Saudi Med. 2007;27(1):36-9. Epub 2007/06/23. doi: 10.5144/0256-4947.2007.36. PubMed PMID: 17582915; PubMed Central PMCID: PMCPMC6077021.

1259. Erkilinç A, Ermerak NO, Zengin A, Olgun Yildizeli Ş, Mutlu B, Karakoç AZ, et al. Is There Any Role of Pulmonary Endarterectomy in Pulmonary Arterial Hydatidosis? Ann Thorac Surg. 2022;114(6):2093-9. Epub 20211126. doi: 10.1016/j.athoracsur.2021.10.035. PubMed PMID: 34843694.

1260. Qian ZX. Thoracic hydatid cysts: a report of 842 cases treated over a thirty-year period. Ann Thorac Surg. 1988;46(3):342-6. Epub 1988/09/01. doi: 10.1016/s0003-4975(10)65941-3. PubMed PMID: 3046523.

1261. Ben-Ismail M, Fourati M, Bousnina A, Zouari F, Lacronique J. [Hydatid cyst of the heart. Apropos of 9 cases]. Arch Mal Coeur Vaiss. 1977;70(2):119-27. Epub 1977/02/01. PubMed PMID: 403888.

1262. Maillet-Vioud C, Bertrand B, Tribouilloy C, Messner-Pellenc P, Cohen A, Dobsak P, et al. [Transesophageal echocardiography in cardiac and paracardiac tumors. A multicenter study]. Arch Mal Coeur Vaiss. 1995;88(9):1307-13. Epub 1995/09/01. PubMed PMID: 8526711.

1263. Mechmeche R, Bousnina A, Ben Ismail M. [Use of coronary angiography in the diagnosis of hydatid cysts of the heart]. Arch Mal Coeur Vaiss. 1983;76(3):305-13. Epub 1983/03/01. PubMed PMID: 6409031.

1264. Trigano JA, Mourot F, Talmoudi T, Malmejac C, Torresani J, Houel J. [Symptomatology of hydatid cyst of the heart. Study of a continuous series of 13 cases and value of x-ray computed tomography]. Arch Mal Coeur Vaiss. 1985;78(13):1895-9. Epub 1985/12/01. PubMed PMID: 3938642.

1265. Birincioglu CL, Kervan U, Tufekcioglu O, Ozen A, Bardakci H, Kucuker SA, et al. Cardiac echinococcosis. Asian Cardiovasc Thorac Ann. 2013;21(5):558-65. Epub 2014/02/27. doi: 10.1177/0218492312463210. PubMed PMID: 24570558.

1266. Kabbani SS, Ramadan A, Kabbani L, Sandouk A, Nabhani F, Jamil H. Surgical experience with cardiac echinococcosis. Asian Cardiovasc Thorac Ann. 2007;15(5):422-6. Epub 2007/10/04. doi: 10.1177/021849230701500513. PubMed PMID: 17911072.

1267. Murat V, Qian Z, Guo S, Qiao J. Cardiac and pericardial echinococcosis: report of 15 cases. Asian Cardiovasc Thorac Ann. 2007;15(4):278-9. Epub 2007/08/01. doi: 10.1177/021849230701500402. PubMed PMID: 17664197.

1268. Duchosal PW, Goncalves J, Odier J. Electrical relations between the cell and the whole heart. Cardiology. 1971;56(1):336-8. Epub 1971/01/01. doi: 10.1159/000169379. PubMed PMID: 4261990.

1269. Shayingca T, Andronikou S, Truter R, Reid E. MRI finding of a papillary muscle cyst: a differential diagnosis. Cardiovasc J Afr. 2012;23(10):e5-6. Epub 2012/11/30. doi: 10.5830/cvja-2012-062. PubMed PMID: 23192289.

1270. Tanyeli O, Dereli Y, Mercan I, Gormus N, Yuksek T. New World's old disease: cardiac hydatid disease and surgical principles. Cardiovasc J Afr. 2017;28(5):304-8. Epub 2017/02/22. doi: 10.5830/cvja-2017-006. PubMed PMID: 28218769; PubMed Central PMCID: PMCPMC5756909.

1271. Shojaee S, Hutchins GM. Echinococcosis complicated by purulent pericarditis. Chest. 1978;73(4):512-4. Epub 1978/04/01. doi: 10.1378/chest.73.4.512. PubMed PMID: 630969.

1272. Gouliamos A, Andreou J, Steriotis J, Kalovidouris A, Vlahos L, Papavassiliou C. Detection of pericardial heart disease by computed tomography. Clin Radiol. 1984;35(5):397-400. Epub 1984/09/01. doi: 10.1016/s0009-9260(84)80202-0. PubMed PMID: 6467827.

1273. Andreou J, Leitman BS, McCauley DI, Gouliamos A, Pontifex G, Naidich DP. The use of computed tomography in the assessment of cardiac masses. Comput Radiol. 1983;7(6):355-9. Epub 1983/11/01. PubMed PMID: 6641204.

1274. Katz AS, Naidech HJ, Malhotra P. The air meniscus as a radiographic finding: a review of the literature and presentation of nine unusual cases. CRC Crit Rev Diagn Imaging. 1978;11(2):167-83. Epub 1978/01/01. PubMed PMID: 753577.

1275. Butt A, Khan J. The Maverick Disease: Cystic Echinococcosis in Unusual Locations: A Ten Year Experience from an Endemic Region. Cureus. 2019;11(10):e5939. Epub 20191018. doi: 10.7759/cureus.5939. PubMed PMID: 31799081; PubMed Central PMCID: PMCPMC6860736.

1276. Inan N, Akhun N, Akansel G, Arslan A, Ciftci E, Demirci A. Conventional and diffusion-weighted MRI of extrahepatic hydatid cysts. Diagn Interv Radiol. 2010;16(2):168-74. Epub 2010/02/09. doi: 10.4261/1305-3825.Dir.2892-09.1. PubMed PMID: 20140855.

1277. Kurkcuoglu IC, Eroglu A, Karaoglanoglu N, Polat P. Complications of albendazole treatment in hydatid disease of lung. Eur J Cardiothorac Surg. 2002;22(4):649-50. Epub 2002/09/26. doi: 10.1016/s1010-7940(02)00396-2. PubMed PMID: 12297195.

1278. Yan F, Huo Q, Abudureheman M, Qiao J, Ma S, Wen H. Surgical treatment and outcome of cardiac cystic echinococcosis. Eur J Cardiothorac Surg. 2015;47(6):1053-8. Epub 2014/09/07. doi: 10.1093/ejcts/ezu323. PubMed PMID: 25193952.

1279. Richter J, Esmann L, Lindner AK, Trebesch I, Equihua-Martinez G, Niebank M, et al. Cystic echinococcosis in unaccompanied minor refugees from Afghanistan and the Middle East to Germany, July 2016 through June 2017. Eur J Epidemiol. 2019;34(6):611-2. Epub 2019/02/11. doi: 10.1007/s10654-019-00492-8. PubMed PMID: 30739267.

1280. Vasile N, Nicoleau F, Mathieu D. CT features of cardio-pericardial masses. Eur J Radiol. 1986;6(1):21-3. Epub 1986/02/01. PubMed PMID: 3699033.

1281. Engin G, Acunas B, Rozanes I, Acunas G. Hydatid disease with unusual localization. Eur Radiol. 2000;10(12):1904-12. Epub 2001/04/18. doi: 10.1007/s003300000468. PubMed PMID: 11305568.

1282. Mancuso L. [Echocardiography in intracardiac masses: important but not always decisive]. G Ital Cardiol. 1991;21(10):1135-6. Epub 1991/10/01. PubMed PMID: 1804752.

1283. Guarini L, Fanelli G, Leggio A. [Cardio-pericardial echinococcosis]. G Ital Chir. 1965;21(4):485-507. Epub 1965/07/01. PubMed PMID: 5863348.

1284. Kraef C, Ramharter M. [Cardiac manifestations of tropical diseases]. Herz. 2019;44(1):83-91. Epub 2019/01/19. doi: 10.1007/s00059-018-4780-5. PubMed PMID: 30656388.

1285. Unal E, Karcaaltincaba M, Akpinar E, Ariyurek OM. The imaging appearances of various pericardial disorders. Insights Imaging. 2019;10(1):42. Epub 2019/03/31. doi: 10.1186/s13244-019-0728-4. PubMed PMID: 30927107; PubMed Central PMCID: PMCPMC6441059.

1286. Abid A, Khayati A, Zargouni N. Hydatid cyst of the heart and pericardium. Int J Cardiol. 1991;32(1):108-9. Epub 1991/07/01. doi: 10.1016/0167-5273(91)90051-p. PubMed PMID: 1864661.

1287. Skuhala T, Trkulja V, Runje M, Balen-Topić M, Vukelić D, Desnica B. Combined Albenazole-Praziquantel Treatment in Recurrent Brain Echinococcosis: Case Report. Iran J Parasitol. 2019;14(3):492-6. PubMed PMID: 31673271; PubMed Central PMCID: PMCPMC6815861.

1288. Kucukarslan N, Kirilmaz A, Ulusoy E, Baysan O, Yildirim V, Ozal E, et al. Eleven-year experience in diagnosis and surgical therapy of right atrial masses. J Card Surg. 2007;22(1):39-42. Epub 2007/01/24. doi: 10.1111/j.1540-8191.2007.00335.x. PubMed PMID: 17239209.

1289. Sultan FAT, Ahmed SW. Cardiac Magnetic Resonance Evaluation of Cardiac Masses in Patients with Suspicion of Cardiac Masses on Echo or Computed Tomography. J Clin Imaging Sci. 2020;10:57. Epub 20200914. doi: 10.25259/jcis_137_2020. PubMed PMID: 33024612; PubMed Central PMCID: PMCPMC7533085.

1290. Sanaei Dashti A, Kadivar MR, Alborzi A, Sadeghi E, Pouladfar GR, Bagherian N, et al. Analysis of hospital records of children with hydatid cyst in south of Iran. J Parasit Dis. 2017;41(4):1044-8. Epub 2017/11/09. doi: 10.1007/s12639-017-0932-4. PubMed PMID: 29114140; PubMed Central PMCID: PMCPMC5660032.

1291. Khannous M, Ferretti G, Ranchoup Y, Thony F, Robert F, Coulomb M. [Intrathoracic hydatid cyst. Contribution of tomodensitometry. Apropos of 25 cases]. J Radiol. 1993;74(11):541-8. Epub 1993/11/01. PubMed PMID: 8283408.

1292. Ben Jomaa S, Haj Salem N, Hmila I, Saadi S, Aissaoui A, Belhadj M, et al. Sudden death and hydatid cyst: A medicolegal study. Leg Med (Tokyo). 2019;40:17-21. Epub 20190705. doi: 10.1016/j.legalmed.2019.07.001. PubMed PMID: 31299424.

1293. Malmejac C, Paoli JM, Poggi L, Metras D, Serradimigni A, Houel J. [Note on cardiac hydatidosis]. Mars Med. 1969;106(5):393-7. Epub 1969/01/01. PubMed PMID: 5351029.

1294. Ekim H, Ozbay B, Kurnaz M, Tuncer M, Ekim M. Management of complicated giant thoracic hydatid disease. Med Sci Monit. 2009;15(12):Cr600-5. Epub 2009/12/01. PubMed PMID: 19946229.

1295. Shevchenko YL, Travin NO, Musaev GH, Morozov AV. Heart echinococcosis: current problems and surgical treatment. Multimed Man Cardiothorac Surg. 2006;2006(810):mmcts.2005.001115. Epub 2006/01/01. doi: 10.1510/mmcts.2005.001115. PubMed PMID: 24412935.

1296. Travin N, Shevchenko Y. Heart echinococcosis: Current problems and surgical treatment. Multimed Man Cardiothorac Surg. 2017;2017. Epub 2018/01/05. doi: 10.1510/mmcts.2017.017. PubMed PMID: 29300073.

1297. Vahedi MA, Vahedi ML. Demographics of patients with surgical and nonsurgical cystic echinococcosis in East Azerbaijan from 2001 to 2012. Pak J Biol Sci. 2012;15(4):186-91. Epub 2012/07/24. doi: 10.3923/pjbs.2012.186.191. PubMed PMID: 22816176.

1298. Dianka MB, El Hamdani T, Kaba I, Naja A, Ibahioin K, El Azhari A. [Multiple cerebral hydatid cysts of cardiac origin: report of a case]. Pan Afr Med J. 2015;22:15. Epub 2015/11/26. doi: 10.11604/pamj.2015.22.15.7743. PubMed PMID: 26600914; PubMed Central PMCID: PMCPMC4646436.

1299. Nozais JP, Danis M, Loisy M, Gentilini M. [Serologic diagnosis of hydatidosis. 235 cases]. Pathol Biol (Paris). 1985;33(4):238-42. Epub 1985/04/01. PubMed PMID: 3925424.

1300. Oueslati S, Said W, Saaidi I, Djebbi M, Charrada L, Rezgui L, et al. [Imaging cardiac hydatid cysts: 8 cases]. Presse Med. 2006;35(7-8):1162-6. Epub 2006/07/15. PubMed PMID: 16840892.

1301. Carcassonne M, Aubrespy P, Dor V, Choux M. Hydatid cysts in childhood. Prog Pediatr Surg. 1973;5:1-35. Epub 1973/01/01. PubMed PMID: 4736028.

1302. Garcia Ortiz E. [Heart cysts and tumors]. An R Acad Nac Med (Madr). 1971;88(2):203-25. Epub 1971/01/01. PubMed PMID: 5171275.

1303. Guhl L, Grawunder HJ, Arlart IP. [Magnetic resonance tomographic findings in cardiac tumors]. Radiologe. 1993;33(3):153-8. Epub 1993/03/01. PubMed PMID: 8480024.

1304. Baltaxe HA, Fleming RJ. The angiographic appearance of hydatid disease. Radiology. 1970;97(3):599-604. Epub 1970/12/01. doi: 10.1148/97.3.599. PubMed PMID: 5497505.

1305. Traibi A, El Hammoumi M, El Oueriachi F, Arsalane A, Kabiri EH. [Benign cysts of the mediastinum: series of 28 cases]. Rev Mal Respir. 2012;29(9):1111-5. Epub 2012/12/04. doi: 10.1016/j.rmr.2011.09.053. PubMed PMID: 23200583.

1306. Mahdhaoui A, Bouraoui H, Majdoub MA, Jeridi G, Hajri SE, Trimeche B, et al. [Cardiopericardial echinococcosis]. Rev Med Suisse Romande. 2003;123(3):169-73. Epub 2004/04/21. PubMed PMID: 15095703.

1307. Bousnina S, Racil H, Maghraoui O, Marniche K, Ben Mrad S, Ghedira H, et al. [Hydatid pulmonary embolisms. Seven cases]. Rev Pneumol Clin. 2005;61(1 Pt 1):31-6. Epub 2005/03/18. PubMed PMID: 15772577.

1308. Mzabi R, Dziri C. [Extrahepatic echinococcosis: diagnosis and treatment]. Rev Prat. 1990;40(3):220-4. Epub 1990/01/21. PubMed PMID: 2305187.

1309. Schratter M, Mayr H, Tscholakoff D, Kramer J, Glogar D, Imhof H. [MRT using Gd-DTPA in the diagnosis of tumor and pseudotumor intracardiac masses]. Rofo. 1990;152(1):16-22. Epub 1990/01/01. doi: 10.1055/s-2008-1046810. PubMed PMID: 2154003.

1310. Gursoy S, Ucvet A, Tozum H, Erbaycu AE, Kul C, Basok O. Primary intrathoracic extrapulmonary hydatid cysts: analysis of 14 patients with a rare clinical entity. Tex Heart Inst J. 2009;36(3):230-3. Epub 2009/07/02. PubMed PMID: 19568393; PubMed Central PMCID: PMCPMC2696497.

1311. Hoyer J, Malmejac C, Delaye A, Houel J. [Contribution to cardiac echinococcosis on the basis of 7 observations (author's transl)]. Thoraxchir Vask Chir. 1974;22(3):197-206. Epub 1974/06/01. doi: 10.1055/s-0028-1102762. PubMed PMID: 4547636.

1312. Drissa H, Zayani Z, Boussaada R, Zaouali RM. [Cardiac hydatid cyst (report of 8 cases)]. Tunis Med. 2001;79(11):633-7. Epub 2002/03/15. PubMed PMID: 11892434.

1313. Jerbi S, Kortas C, Dammak S, Hamida N, Aly F, Mlika S, et al. [Cardio-pericardial hydatid cyst. Report of 19 cases]. Tunis Med. 2004;82 Suppl 1:152-7. Epub 2004/05/07. PubMed PMID: 15127707.

1314. Aarons BJ. Thoracic surgery for hydatid disease. World J Surg. 1999;23(11):1105-9. Epub 1999/09/29. doi: 10.1007/s002689900631. PubMed PMID: 10501870.

1315. Dimitrov M, Jovev G. [X-ray findings in heart tumors and cysts]. Z Gesamte Inn Med. 1972;27(21):919-24. Epub 1972/11/01. PubMed PMID: 4662915.

1316. Unal M, Tuncer C, Serce K, Bostan M, Erem C, Gokce M. A cardiac giant hydatid cyst of the interventricular septum masquerading as ischemic heart disease: role of MR imaging. Acta Cardiol. 1995;50(4):323-6. Epub 1995/01/01. PubMed PMID: 8540274.

1317. Dodek A, DeMots H, Jr., Antonovic JA, Hodam RP. Echinococcus of the heart. An unusual tumor of the heart and liver. Am J Cardiol. 1972;30(3):293-7. Epub 1972/08/01. doi: 10.1016/0002-9149(72)90076-8. PubMed PMID: 5044490.

1318. Natarajan D, Bera M. Hydatid cyst of the heart. BMJ Case Rep. 2009;2009:bcr2006095869. Epub 2009/01/01. doi: 10.1136/bcr.2006.095869. PubMed PMID: 21687140; PubMed Central PMCID: PMCPMC3106030.

1319. Gavrilescu S, Gavrilescu M, Streian C, Luca C. Complete atrio-ventricular block due to cardiac echinococcosis. Cardiology. 1979;64(4):215-21. Epub 1979/01/01. doi: 10.1159/000170618. PubMed PMID: 476728.

1320. Busic Z, Bradaric N, Ledenko V, Pavlek G. Cystic echinococcosis of lung and heart coupled with repeated echinococcosis of brain--a case report. Coll Antropol. 2011;35(4):1311-5. Epub 2012/03/09. PubMed PMID: 22397279.

1321. Pawar PA, Deshmukh PP, Deshpande M, Pusate AA. Asymptomatic Presentation of Large Cardiac Ball. J Assoc Physicians India. 2016;64(12):96-7. Epub 2017/04/14. PubMed PMID: 28406002.

1322. Hela BJ, Abir B, Majdi G, Aiman D, Iheb S, Nizar E, et al. Interventricular septum hydatid cyst presenting with acute lower limb ischemia: a case report. Libyan J Med. 2015;10(1):28634. Epub 2015/01/01. doi: 10.3402/ljm.v10.28634. PubMed PMID: 28349810.

1323. Tetik O, Yilik L, Emrecan B, Ozbek C, Gurbuz A. Giant hydatid cyst in the interventricular septum of a pregnant woman. Tex Heart Inst J. 2002;29(4):333-5. Epub 2002/12/18. PubMed PMID: 12484621; PubMed Central PMCID: PMCPMC140299.

1324. Parvizi R, Namdar H, Bilehjani E, Bayat A, Sheikhalizadeh MA. Simultaneous operation of hydatid cyst of the heart and liver: a case report. J Cardiovasc Thorac Res. 2013;5(3):127-8. Epub 2013/11/21. doi: 10.5681/jcvtr.2013.027. PubMed PMID: 24252990; PubMed Central PMCID: PMCPMC3825392.
